# Synthesis of *N*-alkoxycarbonyl Pyrroles from *O*-Substituted Carbamates: A Synthetically Enabling Pyrrole Protection Strategy

**DOI:** 10.1021/acs.joc.3c01257

**Published:** 2023-09-20

**Authors:** Jodie L. Hann, Catherine L. Lyall, Gabriele Kociok-Köhn, Simon E. Lewis

**Affiliations:** † Department of Chemistry, University of Bath, Bath BA2 7AY, U.K.; ‡ Material and Chemical Characterization Facility (MC2), University of Bath, Bath BA2 7AY, U.K.

## Abstract

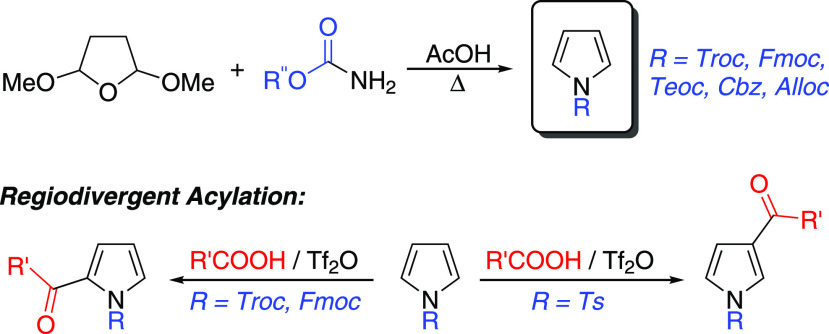

The condensation of readily available *O*-substituted carbamates with 2,5-dimethoxytetrahydrofuran gives *N*-alkoxycarbonyl pyrroles in a single step and in good yield. By this method, several common amine protecting groups can be introduced on the pyrrole nitrogen. With the exception of *N*-Boc, *N*-alkoxycarbonyl groups have seen only minimal use for protection of the pyrrole nitrogen to date. Here, we show that *N*-alkoxycarbonyl protection can endow pyrrole with distinct reactivity in comparison with *N*-sulfonyl protection, for example, in a pyrrole acylation protocol employing carboxylic acids with a sulfonic acid anhydride activator.

## Introduction

**Scheme 1:**
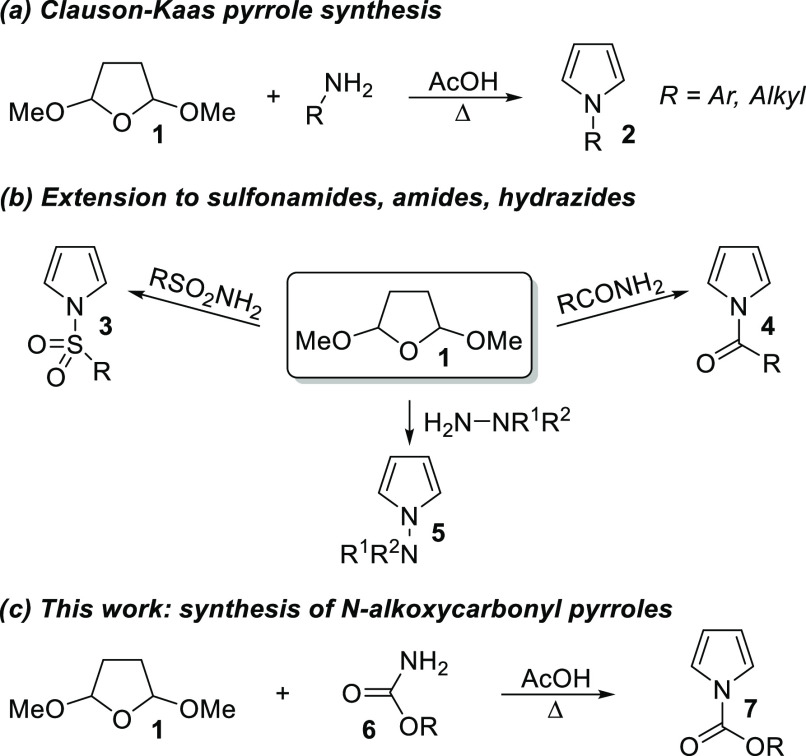
Pyrrole Synthesis Employing 2,5-Dimethoxytetrahydrofuran **1**

Pyrroles are commonplace in both natural products^1,2^ and drug substances.^3^ Accordingly, many methods have been reported for pyrrole synthesis^4,5^ and functionalization.^6^ The Clauson-Kaas pyrrole synthesis employs 2,5-dimethoxytetrahydrofuran **1** as a 1,4-dicarbonyl surrogate, which reacts with amines to afford pyrroles **2** as shown in Scheme 1a.^7^ Subsequently, this methodology has been expanded to encompass the reaction of nitrogen-containing reagents other than simple amines. For example, reaction of **1** with sulfonamides gives *N*-sulfonyl pyrroles **3** (Scheme 1b),^8^ reaction with amides gives *N*-acylpyrroles **4**,^9−11^ and reaction with hydrazines and hydrazides gives *N*-aminopyrroles **5**.^12,13^ The reaction of **1** with an *O*-substituted carbamate **6** to give an *N*-alkoxycarbonyl pyrrole **7** (Scheme 1c) has not previously been reported in the peer-reviewed literature to the best of our knowledge. In this letter, we report that such reactions proceed cleanly and in high yield to afford *N*-alkoxycarbonyl pyrroles.

The electron-rich nature of pyrrole often necessitates the introduction of a protecting group to render oxidative degradation less facile. Protection at nitrogen may also be required to block *N*-deprotonation. In this context, *N*-sulfonyl pyrroles **3** have often been employed, as have *N*-acyl and *N*-aminopyrroles (**4** and **5**), along with protecting group strategies such as *N-* silylation, allylation, and benzylation.^14^ In contrast, *N*-alkoxycarbonyl substituents are underexploited as protecting groups for pyrroles. While many pyrroles have been prepared starting from *N*-Boc pyrrole, other *N*-alkoxycarbonyl pyrroles have scarcely been explored. Indeed, even simple compounds such as *N*-Fmoc pyrrole and *N*-Troc pyrrole are unreported to date. An *N*-alkoxycarbonyl substituent is expected to exert an electron-withdrawing effect on the pyrrole ring, thus enhancing stability. However, distinct reactivity from that of *N*-sulfonyl pyrroles may be expected. A computational study comparing these two classes of *N*-substituted pyrroles concluded that an *N*-alkoxycarbonyl pyrrole possesses a *more* electron-deficient nitrogen than the analogous *N*-sulfonylpyrrole; this is due in part to a degree of pyramidalization at nitrogen in the latter case.^15^

## Results and Discussion

**Scheme 2:**
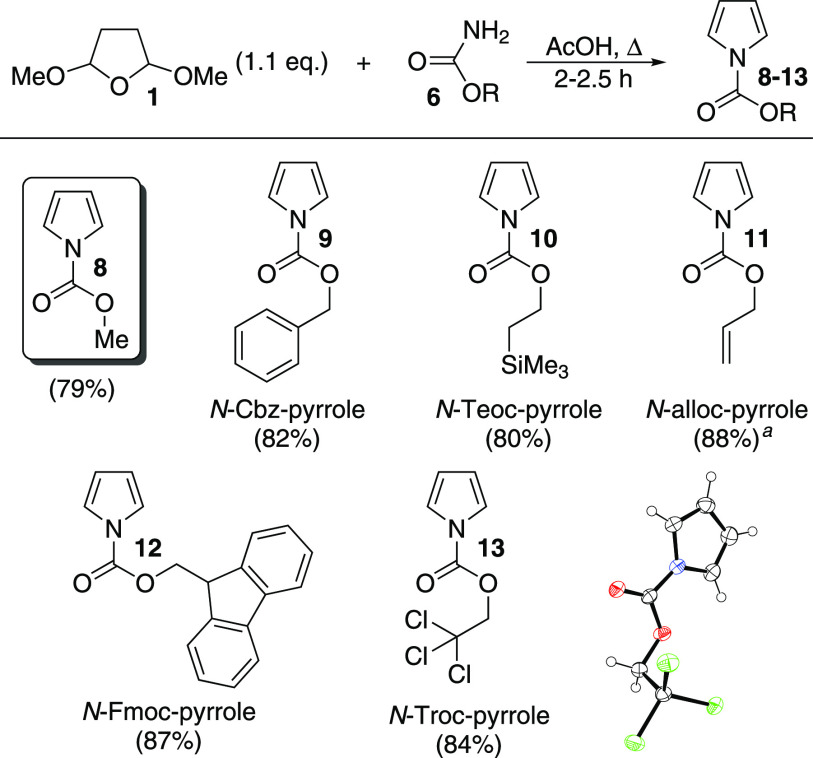
Synthesis of *N*-alkoxycarbonyl Pyrroles **8**–**13**^,^ ORTEP representation of the X-ray structure of **13** (CCDC #2241194) shows ellipsoids at 50% probability. Hydrogens are shown as spheres of arbitrary radius. Only one of two independent molecules in the unit cell is shown for clarity. Crystals of **13** were formed upon cooling to 0 °C for a prolonged period. 1.0 equiv of **1** used, as **11** was unstable upon prolonged drying.

We began our study by applying the reported Clauson-Kaas conditions (reflux in acetic acid) to **1** and the simplest *O*-substituted carbamate (**6**, R = Me). Gratifyingly, we found that no modification of the original reaction conditions was required to afford the product **8** in good yield (Scheme 2). We then carried out the reaction with a range of *O*-substituted carbamates that correspond to well-known protecting groups. These starting materials are either commercially available or easily prepared (see Supporting Information). Passing the crude *N*-alkoxycarbonyl pyrrole products through a plug of silica, then drying under high vacuum to remove any residual **1**, was sufficient to provide material of good purity.

**Scheme 3:**
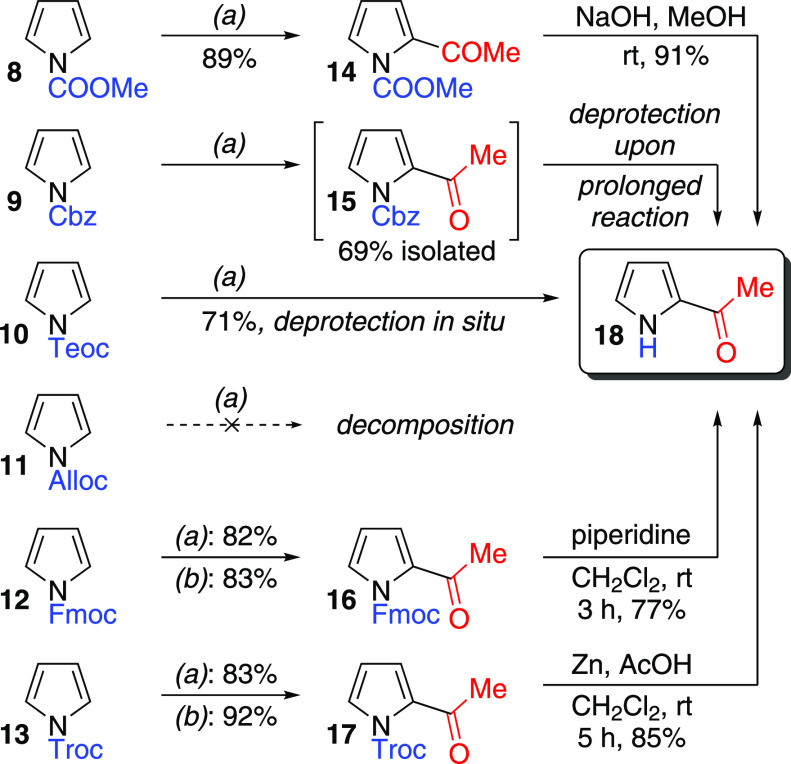
Acetylation of *N*-alkoxycarbonyl Pyrroles **8**–**13** *(a)* 3.0 equiv AcOH, 10 equiv TFAA, CH_2_Cl_2_, rt. *(b)* 1.0 equiv AcOH, 10 equiv Tf_2_O, CH_2_Cl_2_, 0 °C to rt.

We next sought to evaluate the usefulness of *N*-alkoxycarbonyl pyrroles in a representative synthetic transformation. We selected pyrrole acylation, as this process has been extensively studied and many synthetic methods have been reported.^16−28^ A key consideration is regioselectivity, with reported methods exhibiting differing selectivity for 2-acyl/3-acyl/diacyl products. Many methods employ carboxylic acid derivatives as reagents (acid chlorides, anhydrides, *etc*.). Knight et al. reported that *N*-tosylpyrroles could be effectively acylated using trifluoroacetic anhydride (TFAA) to activate carboxylic acids *in situ*.^29^ The reaction was proposed to proceed by formation of a mixed carboxylic acid anhydride, which acts as the acylating agent. An alternate proposal is that the mixed anhydride fragments to an acylium ion and that this is the actual acylating agent.^30^ We applied Knight’s conditions to *N*-alkoxycarbonyl pyrroles **8**–**13**, using acetic acid in an attempt to form 2-acetyl pyrroles (Scheme 3).

*N*-COOMe-pyrrole **8** gave the expected 2-acetyl derivative **14** in good yield. *N*-Cbz-pyrrole **9** also gave 2-acetyl derivative **15**, but then underwent rapid *N*-deprotection under the reaction conditions, giving 2-acetylpyrrole **18** directly. Careful monitoring of the reaction allowed isolation of **15** in 69% yield. The newly installed acyl group in **15** and the TFA byproduct may both play a role in the Cbz cleavage since *N*-Cbz-pyrrole **9** itself remains unchanged upon prolonged exposure to TFA. *N*-Teoc-pyrrole **10** underwent both 2-acetylation and deprotection in one pot; in this case, no acylated intermediate was observable and only 2-acetylpyrrole **18** was isolated. *N*-Alloc-pyrrole **11** decomposed upon attempted acetylation; the instability of such alkene-containing substrates under the acidic reaction conditions has been previously noted.^29^ Finally, *N*-Fmoc-pyrrole **12** and *N*-Troc-pyrrole **13** both gave the corresponding 2-acetyl derivatives **16** and **17** in good yield. It is notable that in every case the only products were the 2-acetylated pyrroles, with none of the corresponding 3-acetyl isomers (or any diacetyl isomers) formed. Deprotection of **14**, **16**, and **17** was then attempted. Basic hydrolysis of **14** gave 2-acetylpyrrole **18** in good yield. Similarly, 2-acetyl-*N*-Fmoc-pyrrole **16** was cleanly deprotected to **18** under classical conditions^31^ (piperidine/CH_2_Cl_2_). Finally, 2-acetyl-*N*-Troc-pyrrole **17** also gave **18** cleanly upon treatment with zinc in acetic acid.^32^

TFAA-mediated acetylations of *N*-alkoxycarbonyl pyrroles (conditions (*a*); Scheme 3) required long reaction times (16–24 h). We reasoned the reaction time could be reduced through the use of alternative reaction conditions to generate a more reactive electrophile *in situ*. Specifically, use of trifluoromethanesulfonic anhydride (Tf_2_O) instead of TFAA could afford significant rate accelerations. This modified approach is expected to proceed *via* formation of a mixed carboxylic sulfonic anhydride.^33^ In the first instance, we employed this alternative activating agent with *N*-Fmoc-pyrrole **12** and *N*-Troc-pyrrole **13**, since we judged these to be the most useful protecting groups in the context of pyrrole acylation (conditions (*b*); Scheme 3). In both cases, the acylated products were formed more quickly (<30 min) and with a modest yield increase. Another advantage of using Tf_2_O as the activator is that only one equivalent of the carboxylic acid was required.

**Scheme 4:**
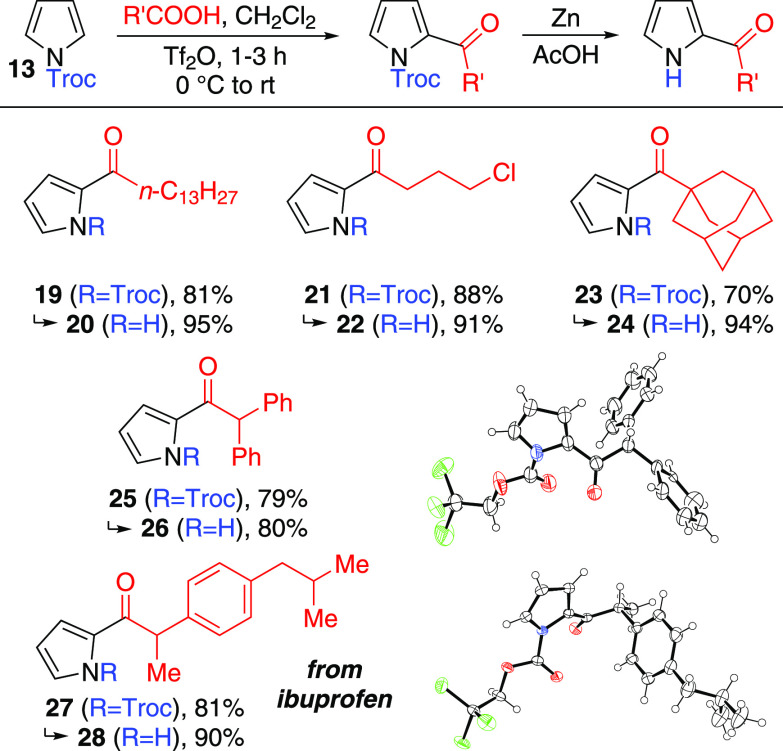
*N*-Troc-pyrrole Acylation and Deprotection ORTEP representations of the X-ray structures of **25** (CCDC #2241195) and **27** (CCDC #2241193) show ellipsoids at 50% probability. Hydrogens are shown as spheres of arbitrary radius. Disorder in the CCl_3_ group and a phenyl ring of **25**, and in the *iso*butyl group of **27**, is omitted for clarity. Crystals **25** formed upon prolonged standing of a solution of **25** in CDCl_3_ and crystals **27** formed by diffusion of hexane vapor into a CH_2_Cl_2_ solution of **27.**

Having demonstrated the applicability of these acylation conditions to *N*-alkoxycarbonyl pyrroles, we selected *N*-Troc-pyrrole **13** as a substrate to explore the scope of the acylation reaction (Scheme 4). This was due to the wide synthetic utility of the Troc group (due to its inertness to a wide variety of reaction conditions), as well as the ease of purification of the deprotected pyrrole **18** formed from **17** (simple filtration through a plug of celite). All the acylated *N*-Troc pyrroles in Scheme 4 formed in good yield within 1–3 h and could be cleanly deprotected to the corresponding *N*-H pyrroles also in good yield. In each case, once the starting material was consumed, the 2-acylated isomer was the sole product present (and the sole product isolated if the reaction was worked up at this point). Upon prolonged reaction (>18 h), a degree of isomerization to the 3-acylated isomer was observed for some substrates. No diacylation was ever observed, even when 3 equiv of acetic acid and a reaction time of 24 h were used.

**Scheme 5:**
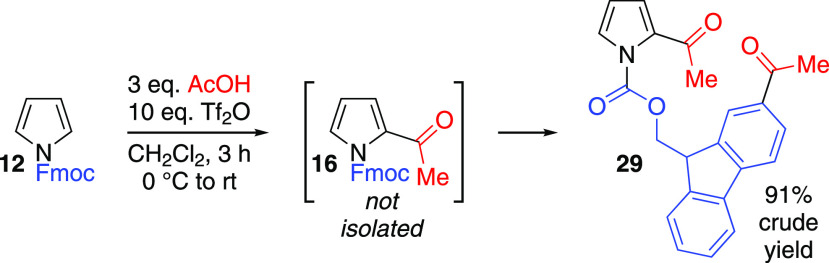
Overacetylation of *N*-Fmoc Pyrrole

The monoacylation of *N*-Troc-pyrrole **13** even in the presence of excess carboxylic acid is in contrast to the outcome with *N*-Fmoc pyrrole **12**. When 3 equiv of acetic acid were used in the acetylation of **12**, diacylated product **29** was isolated instead of **16** (Scheme 5). Compound **29** was unstable on silica and hence was characterized in crude form. Seemingly, the presence of both the *N*-alkoxycarbonyl group and the 2-acyl group in **16** render the pyrrole less reactive toward further acylation than the fluorenyl motif. In comparison, overacetylation of *N*-tosyl pyrrole **30** under the same reaction conditions (3 equiv AcOH/Tf_2_O) results in the introduction of a second acetyl group on the pyrrole ring (see Supporting Information Figures S198,S199).

**Scheme 6:**
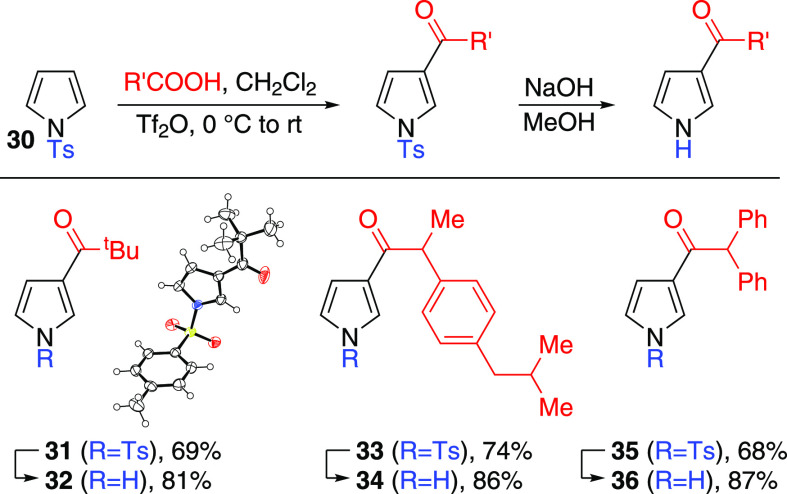
*N*-Ts-pyrrole Acylation and Deprotection ORTEP representation of the X-ray structure of **31** (CCDC #2241194) shows ellipsoids at 50% probability. Hydrogens are shown as spheres of arbitrary radius. Crystals of **31** were formed by diffusion of hexane vapor into a CH_2_Cl_2_ solution of **31**.

The exclusive formation of 2-acylated products from the *N*-alkoxycarbonyl pyrroles above is distinct from the outcome of some of the other acylation protocols reported for pyrroles with other *N*-substituents. Partial or total selectivity for 3-acylation has been achieved by various means.^34,35^ It is well established that 2-acylpyrroles may be isomerized to the corresponding 3-acyl isomers under Brønsted acid catalysis, with the ease of this process depending on factors including acid strength, acyl group, and *N*-substituent.^36−38^ The acylation methodology described here by us (scheme 4–5) results in the formation of a stoichiometric quantity of acid byproduct (either TFA or TfOH, depending on the activating agent used). However, isomerization to 3-acylated products occurs only very slowly, if at all (*vide supra*). As such, we reasoned the *N*-alkoxycarbonyl protecting group might particularly disfavor the isomerization. To determine if this is the case, we studied acylation of *N*-tosyl pyrrole **30** under the same reaction conditions (*i.e.*, Tf_2_O as an activator), to allow comparison with *N*-alkoxycarbonyl pyrroles (Scheme 6).

In each case shown, 3-acylated-*N*-tosylpyrroles were the only isomers isolated (Scheme 6). Identification of the products as the 3-acylated isomers was on the basis of x-ray crystallography (for **32**) as well as the spectroscopic inequivalence of the deprotected products (**34**, **36**) from their 2-acylated counterparts (**26**, **28**) and diagnostic 2D-NMR data (see Supporting Information). The clean formation of these 3-acyl isomers from an *N*-sulfonyl substrate under these conditions shows that there is good regiocomplementarity between the *N*-alkoxycarbonyl pyrroles we have described here and the already-established *N*-sulfonyl pyrroles. We propose that under the strongly acidic conditions, initial 2-acylation of *N*-tosyl pyrrole **30** is followed by isomerization (as opposed to direct acylation at the 3-position). In support of this proposition, NMR reaction monitoring of the acetylation of **30** with AcOH/Tf_2_O allows the formation and disappearance of peaks corresponding to the 2-acyl isomer to be observed as the reaction progresses to the endpoint of 3-acyl product formation. The finding that *N*-alkoxycarbonyl pyrroles are much more resistant to this isomerization under the same conditions is in keeping with the computational prediction that *N*-alkoxycarbonyl pyrroles are more electron-poor^15^ and hence less readily protonated.

## Conclusions

In conclusion, we have described a facile one-step synthesis of *N*-alkoxycarbonyl pyrroles, a class of compounds that have been scarcely exploited in synthesis to date (with the exception of *N*-Boc-pyrrole). We anticipate that this rapid access to pyrroles bearing previously unused protecting groups will lead to their utilization in diverse synthetic contexts. To showcase their potential utility, we have demonstrated the applicability of these *N*-alkoxycarbonyl pyrroles in a straightforward acylation protocol that employs electrophiles generated *in situ* from simple carboxylic acids upon activation with triflic anhydride. (Instability of *N*-Boc-protected pyrroles under acidic conditions would preclude their use in this process). We have further shown the outcome of the acylation to be regioselective depending on the protecting group used, with *N*-alkoxycarbonyl- and *N*-sulfonyl pyrroles giving regioisomeric products (after deprotection). We anticipate the versatility of the *N*-alkoxycarbonyl pyrrole formation may be further increased by the use of substituted variants of **1**, as has been demonstrated for the original Clauson-Kaas process.^7,39−49^

## Experimental Procedure

### General Procedure for *N*-alkoxycarbonyl Pyrrole Synthesis

Carbamate (4 mmol, 1.0 equiv) and 1,4-dimethoxytetrahydrofuran (0.63 mL, 4.4 mmol, 1.1 equiv, mixture of *cis* and *trans*) were added to a flask and purged with nitrogen. AcOH (2.2 mL) was added, and the reaction was heated to reflux (110 °C) using a heating mantle. The reaction was monitored by TLC, and upon completion was cooled to ambient temperature. (Vanillin TLC stain used for non-UV active substrates). CH_2_Cl_2_ (50 mL) was added, then washed with saturated Na_2_CO_3(aq)_ (50 mL × 2), then brine (50 mL). The organic layer was dried over MgSO_4_ and filtered, and then the filtrate was concentrated in *vacuo*. The crude product was purified by passage through a silica plug (elution with CH_2_Cl_2_), and any residual 1,4-dimethoxytetrahydrofuran starting material was removed under a vacuum.

### General Procedure for Acylation Reactions Using TFAA

To a nitrogen-purged flask of *N*-alkoxycarbonyl pyrrole (1.0 equiv), and carboxylic acid (3.0 equiv) in dry dichloromethane (*c* = 0.44 M), trifluoroacetic anhydride (10 equiv) was added dropwise at ambient temperature. The reaction was monitored by TLC, and upon completion, the reaction was diluted with CH_2_Cl_2_ and washed with 1 M Na_2_CO_3(aq)_. The organic layer was separated, and the aqueous layer was extracted with CH_2_Cl_2_ (×2). The combined organic solutions were then washed with brine, dried over MgSO_4_, and filtered, then the filtrate was concentrated in *vacuo*. The crude product was purified by column chromatography (SiO_2_, EtOAc–Pet Ether).

### General Procedure for Acylation Reactions Using Tf_2_O

To a nitrogen-purged flask of *N*-alkoxycarbonyl pyrrole (1 equiv), and carboxylic acid (1 equiv) in dry dichloromethane (*c* = 0.44 M), trifluoromethanesulfonic anhydride (10 equiv) was added dropwise at 0 °C. The reaction was stirred without further cooling and monitored by TLC, and upon completion, the reaction was diluted with CH_2_Cl_2_ and washed with 1 M Na_2_CO_3_ (aq). The organic layer was separated, and the aqueous layer extracted with CH_2_Cl_2_ (×2). The combined organic solutions were then washed with brine, dried over MgSO_4_, and filtered, then the filtrate was concentrated in *vacuo*. The crude product was purified by column chromatography (SiO_2_, EtOAc–Pet Ether).

### General Procedure for *N*-Troc Deprotection

To zinc dust (1.72 mmol) and the *N*-Troc-protected product (0.44 mmol), CH_2_Cl_2_ (3.2 mL) and AcOH (0.66 mL) were added. The reaction was stirred at ambient temperature and monitored by TLC until consumption of the starting material. The reaction mixture was diluted with acetone (30 mL) and filtered through celite, then concentrated under a vacuum to obtain an analytically pure product.

### General Procedure for *N*-Tosyl Deprotection

NaOH pellets (3 equiv) were crushed and added to a solution of *N*-Tosyl pyrrole product in (9:1) MeOH/H_2_O (0.81 M) and stirred overnight at ambient temperature. EtOAc was added, the phases were separated, and the aqueous phase was extracted with EtOAc. The combined organic extracts were washed with brine, dried over MgSO_4_, and filtered. The filtrate was evaporated to dryness to obtain analytically pure product.

## Data Availability

The data underlying this study are available in the published article, in its Supporting Information, and openly available in the University of Bath Research Data Archive at https://doi.org/10.15125/BATH-01255

## Supporting Information Available

Additional experimental procedures, tabulated compound characterization data, NMR spectra, IR spectra, X-ray crystallographic data (PDF)
