# Supplementary material for: Synthesis of N-alkoxycarbonyl Pyrroles from O-Substituted Carbamates: A Synthetically Enabling Pyrrole Protection Strategy
Source: J Org Chem. 2023 Sep 20;88(19):13584–9. doi: 10.1021/acs.joc.3c01257 (PMC10563134; doi:10.1021/acs.joc.3c01257)
Supplement: Supplementary file 1 — jo3c01257_si_001.pdf [file jo3c01257_si_001.pdf]

Synthesis of *N*-alkoxycarbonyl pyrroles from *O*-substituted carbamates – A synthetically enabling pyrrole protection strategy.

Jodie L. Hann,[a] Catherine L. Lyall[b], Gabriele Kociok-Köhn[b] and Simon E. Lewis*[a]

a. Department of Chemistry, University of Bath, Bath, BA2 7AY, UK. S.E.Lewis@bath.ac.uk

b. Material and Chemical Characterization Facility (MC²), University of Bath, Bath, BA2 7AY, UK.

ELECTRONIC SUPPORTING INFORMATION

General Experimental Details Page S2

Preparation of *O*-substituted carbamate starting materials Page S3

Preparation of *N*-Alkoxycarbonyl pyrroles Page S4

Preparation of Acetylated *N*-Alkoxycarbonyl pyrroles Page S7

Procedures for Deprotection of Acylated pyrroles Page S9

Preparation of Acylated *N*-Alkoxycarbonyl pyrroles from other carboxylic acids and their deprotection Page S10

Preparation of Acylated *N*-sulfonyl pyrroles and their deprotection Page S16

NMR and IR Spectra Page S20

X-ray Crystallographic Data Page S133

References Page S157

General Experimental Details Synthetic chemistry and compound characterisation.

Chemicals

Reagents were purchased from Fluorochem, Merck and Alfa Aesar.

Solvents

Reactions were carried out under an atmosphere of nitrogen, by purging the vessel with a flow of nitrogen. Dichloromethane, hexane and diethyl ether were dried and degassed by passing through anhydrous alumina columns using an Innovative Technology Inc. PS-400-7 solvent purification system. Pet Ether refers to petroleum ether, bp 40-60 °C. Solvents were removed using Büchi rotary evaporators and with high vacuum on a Schlenk line.

Melting Points

Capillary melting points were recorded on a Büchi 535 melting point apparatus and are uncorrected.

NMR Spectroscopy

^{1}H and ^{13}C NMR spectra were obtained using a 500 MHz Agilent ProPulse 500 or a 400 MHz Bruker NMR spectrometer, for which proton decoupling was active for ^{13}C NMR. Spectra were acquired at 298 K and were referenced to residual solvent peaks. Chemical shifts are reported in parts per million (ppm) relative to residual chloroform (δ = 7.26 ppm, ^{1}H; 77.16 ppm, ^{13}C), acetone (δ = 2.05 ppm, ^{1}H; 29.84, 206.26 ppm, ^{13}C) or dimethyl sulfoxide (δ = 2.50 ppm, ^{1}H; 39.52 ppm, ^{13}C). Coupling constants, *J*, reported in Hz, were calculated using MestreNova x64 to the nearest 0.1 Hz. ^{1}H and ^{13}C{^{1}H} assignments for novel compounds are corroborated though 2D NMR experiments (COSY, NOESY, HSQC, HMBC).

Infrared Spectroscopy

Infrared (IR) spectra were recorded on a Perkin-Elmer Spectrum 100 ATR-FTIR spectrometer with only selected absorbances quoted as ν in cm^{-1} .

Mass Spectroscopy

For mass spectrometry a microTOF electrospray time-of-flight (ESITOF) mass spectrometer (Bruker Daltonik GmbH, Bremen, Germany) was used. Data are reported in the form of *m/z*. The observed mass and isotope pattern matched the corresponding theoretical values as calculated from the expected molecular formula.

X-ray Crystallography

X-Ray crystallography was recorded on a Nonius Kappa CCD diffractometer with Mo-Kα radiation (λ=0.71074 Å). All structures were solved by direct methods and refined on all F2 data using SHELX-97 suite of programs.

Chromatography

Analytical thin-layer chromatography was performed on Merck silica gel 60 F254 aluminium-backed plates. Visualisation was accomplished with UV light (254 nm), and vanillin stain. Automated flash column chromatography (normal phase) was performed using a CombiFlash NextGen 300+ system equipped with UV and ELSD detectors, using 12 g silica columns.

Preparation of *O*-substituted carbamate starting materials.

Methyl carbamate, benzyl carbamate and (9H-fluoren-9-yl)methyl carbamate were purchased from commercial suppliers.

2,2,2-Trichloroethyl carbamate synthesised as according to literature.[1]

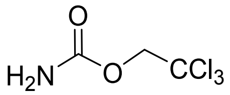

^1H NMR (400 MHz, $CDCl_3$) δ 4.90 (s, 2H), 4.74 (s, 2H).

Allyl carbamate – synthesised as according to literature.[2]

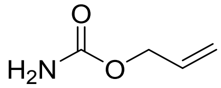

^1H NMR (400 MHz, $CDCl_3$) δ 5.97 – 5.86 (m, 1H), 5.35 – 5.28 (m, 1H), 5.22 (dt, *J* = 10.5, 1.3 Hz, 1H), 4.80 (s, 2H), 4.56 (ddd, *J* = 5.5, 1.3, 1.3 Hz, 2H).

2-(Trimethylsilyl)ethyl carbamate – synthesised as according to literature.[3]

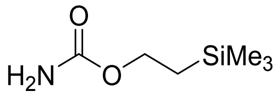

^1H NMR (400 MHz, $CDCl_3$) δ 5.17 – 4.78 (m, 2H), 4.28 – 3.96 (m, 2H), 1.10 – 0.80 (m, 2H), 0.01 (s, 9H).

Preparation of *N*-Alkoxycarbonyl pyrroles. The *General procedure for Synthesis of N-Alkoxycarbonyl pyrroles* in the main article was used to synthesize the following compounds:

Methyl 1*H*-pyrrole-1-carboxylate (8)[4]

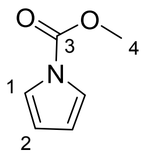

Reaction time: 2.5 h

Yellow oil, 0.38 g, 79%

^{1}H NMR (400 MHz, $CDCl_3$) δ 7.27 (dd (app t), *J* = 2.4, 2.4 Hz, 2H, H^1), 6.28-6.22 (m, 2H, H^2), 3.96 (s, 3H, H^4). HRMS (ESI+) m/z calcd. for ($C_8H_9NO_2$) $[M+H]^+$ 126.0550; found 126.0552. R_f 0.27 (2.5% EtOAc/97.5% Pet Ether)

Benzyl 1*H*-pyrrole-1-carboxylate (9)[5]

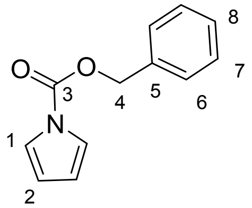

Reaction time: 2 h

Yellow oil, 0.66 g, 82%

^{1}H NMR (400 MHz, $CDCl_3$) δ 7.48 – 7.33 (m, 5H, H^{6-8}), 7.30 (dd (app t), *J* = 2.4,2.4 Hz, 2H, H^1), 6.25 (dd, *J* = 2.4, 1.8 Hz, 2H, H^2), 5.38 (s, 2H, H^4). HRMS (ESI+) m/z calcd. for ($C_{12}H_{11}NO_2$) $[M+H]^+$ 202.0865; found 202.0866. R_f 0.24 (2.5% EtOAc/97.5% Pet Ether)

2-(Trimethylsilyl)ethyl 1*H*-pyrrole-1-carboxylate (10)[6]

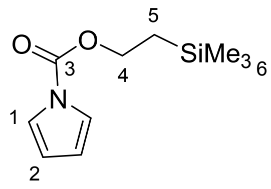

Reaction time: 2 h

Yellow oil, 0.68 g, 80%

^{1}H NMR (400 MHz, $CDCl_3$) δ 7.27 (dd, *J* = 2.7, 2.0 Hz, 2H, H^1), 6.24 (dd, *J* = 2.6, 2.0 Hz, 2H, H^2), 4.57–4.22 (m, 2H, H^4), 1.21–1.09 (m, 2H, H^5), 0.09 (s, 9H, H^6). $^{13}C\{^1H\}$ NMR (101 MHz, $CDCl_3$) δ 150.7 (C^3), 120.1 (C^1), 112.4 (C^2), 66.1 (C^4), 17.7 (C^5), -1.1 (C^6); IR (neat) 2988, 1717, 1454, 1407, 1394, 1250, 1075, 1056, 859, 700, 579 cm^{-1} HRMS (ESI+) m/z calcd. for ($C_{10}H_{17}NO_2Si$) $[M+Na]^+$ 234.0921; found 234.0942. R_f 0.25 (15% EtOAc/85% Pet Ether)

Allyl 1*H*-pyrrole-1-carboxylate (11)[7]

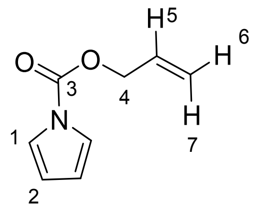

Reaction time: 2 h

Yellow oil, 0.53 g, 88%

^{1}H NMR (400 MHz, $CDCl_3$) δ 7.31 (dd, *J* = 2.7, 2.0 Hz, 2H, H^1), 6.26 (dd, *J* = 2.7, 2.0 Hz, 2H, H^2), 6.03 (ddt, *J* = 17.2, 10.4, 5.8 Hz, 1H, H^5), 5.43 (dt, *J* = 17.2, 1.5 Hz, 1H, H^7), 5.33 (dt, *J* = 10.4, 1.3 Hz, 1H, H^6), 4.84 (ddd, *J* = 5.8, 1.3, 1.3 Hz, 2H, H^4). $^{13}C\{^1H\}$ NMR (101 MHz, $CDCl_3$) δ 150.2 (C^3), 131.4 (C^5), 120.1 ($C^{6/7}$), 119.3 (C^1), 112.6 (C^2), 67.7 (C^4); IR (neat) 2970, 2950, 1741, 1649. 1572, 1272, 1335, 1307, 1229, 937, 877, 736 cm^{-1}. HRMS (ESI+) m/z calcd. for ($C_8H_9NO_2$) $[M+H]^+$ 152.0706; found 152.0712. R_f 0.30 (2.5% EtOAc/97.5% Pet Ether)

(9*H*-Fluoren-9-yl)methyl 1*H*-pyrrole-1-carboxylate (12)

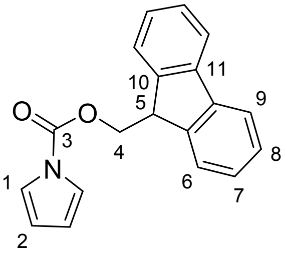

Reaction time: 2 h

Beige powder, 1.01 g, 87%, m.p. 87-88°C

^{1}H NMR (400 MHz, $CDCl_3$) δ 7.80 (dd, *J* = 7.5, 1.0 Hz, 1H, H^9), 7.61 (dd, *J* = 7.5, 1.0 Hz, 1H, H^6), 7.47-7.40 (m, 1H, H^8), 7.34 (ddd, *J* = 7.5, 7.5, 1.2 Hz, 1H, H^7), 7.30 (dd (app t), *J* = 2.4, 2.4Hz, 1H, H^1), 6.32–6.27 (m, 1H, H^2), 4.65 (d, *J* = 7.1 Hz, 2H, H^4), 4.37 (t, *J* = 7.0 Hz, 1H, ^{5}H). ^{13}C{^{1}H} NMR (101 MHz, $CDCl_3$) δ 150.7 (C^3), 143.3 (C^{11}), 141.5 (C^{10}), 128.2 (C^8), 127.4 (C^7), 125.1 (C^6), 120.3 (C^9), 120.2 (C^1), 112.9 (C^2), 69.2 (C^4), 46.9 (C^5). HRMS (ESI+) m/z calcd. for ($C_{19}H_{15}NO_2$) $[M+H]^+$290.1176; found 290.1175; IR (neat) 2954, 1741, 1709, 1473, 1450, 1382, 1334, 1169, 947, 886, 757, 621 cm^{-1}. R_f 0.19 (2.5% EtOAc/97.5% Pet Ether)

2,2,2-Trichloroethyl 1*H*-pyrrole-1-carboxylate (13)

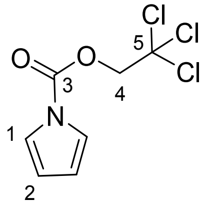

Reaction time: 2 h

Colourless crystals (stored at 0 °C) 0.81 g, 84%, m.p. 34-35 °C

^{1}H NMR (400 MHz, $CDCl_3$) δ 7.35 (dd, *J* = 2.7, 2.0 Hz, 2H, H^1), 6.31 (dd, *J*= 2.7, 2.1 Hz, 2H, H^2), 4.97 (s, 2H, H^4). ^{13}C{^{1}H} NMR (101 MHz, $CDCl_3$) δ 149.0 (C^3), 120.5 (C^1), 113.6 (C^2), 94.0 (C^5), 75.9 (C^4); IR (neat) 3127, 3158, 3015, 1781, 1756, 1548, 1433, 1471, 1378, 1281, 1032, 1062, 820. 755, 574 cm^{-1} HRMS (ESI+) m/z calcd. for ($C_7H_6Cl_3NO_2$) $[M+H]^+$ 241.9537; found 241.9516. R_f 0.20 (2.5% EtOAc/97.5% Pet Ether).

Preparation of acetylated *N*-Alkoxycarbonyl pyrroles. The *General Procedure for Acylation reactions using TFAA* and the *General Procedure for Acylation reactions using Tf_2O* in the main article were used to synthesize the following compounds:

Methyl 2-acetyl-1H-pyrrole-1-carboxylate (14)

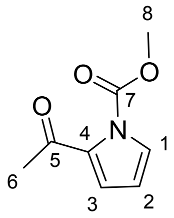

Reaction time: 18 h

Purified *via* column chromatography (silica gel, 0% to 5% EtOAc/Pet Ether)

Colourless oil, 85 mg, 89%

^{1}H NMR (400 MHz, $CDCl_3$) δ 7.35 (dd, *J* = 3.1, 1.6 Hz, 1H, H^1), 6.90 (dd, *J* = 3.6, 1.6 Hz, 1H, H^3), 6.20 (dd (app t), *J* = 3.3, 3.3 Hz, 1H, H^2), 3.96 (s, 3H,H^8), 2.46 (s, 3H, H^6). $^{13}C\{^1H\}$ NMR (101 MHz, $CDCl_3$) δ 188.4 (C^5), 151.2 (C^7), 134.5 (C^4), 128.1 (C^1) , 122.1 (C^3), 110.8 (C^2), 54.9 (C^8), 28.0 (C^6); IR (neat) 3135, 2921, 2852, 1767, 1671, 1545, 1344, 1263, 1061, 752, 591 cm^{-1}. HRMS (ESI+) m/z calcd. for ($C_8H_9NO_3$) $[M+H]^+$ 168.0652; found 168.0652. R_f 0.18 (5% EtOAc/95% Pet Ether)

Benzyl 2-acetyl-1*H*-pyrrole-1-carboxylate (15)

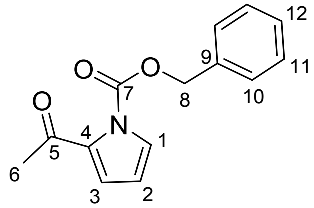

Reaction time: 7 h

Purified *via* column chromatography (silica gel, 5% to 10% EtOAc/Pet Ether)

Colourless oil, 80 mg, 69%

^{1}H NMR (400 MHz, $CDCl_3$) δ 7.45–7.37 (m, 5H, H^{10-12}), 7.37–7.35 (m, 1H, H^1), 6.90 (dd, *J* = 3.6, 1.6 Hz, 1H, H^3), 6.19 (dd, *J* = 3.6, 3.1 Hz, 1H, H^2), 5.37 (s, 2H, H8), 2.45 (s, 3H, H6). $^{13}C\{^1H\}$ NMR (101 MHz, $CDCl_3$) δ 188.5 (C^5), 150.5 (C^7), 134.7 (C^9), 134.6 (C^4), 129.0 (C^{12}), 128.86 ($C^{10/11}$), 128.84 ($C^{10/11}$), 128.1 (C^1), 122.1 (C^3), 110.8 (C^2), 70.1 (C^8), 28.9 (C^6); IR (neat) 3127, 2962, 1767, 1673, 1377, 1285, 1031. 741, 716, 575 cm^{-1}. HRMS (ESI+) m/z calcd. for ($C_{14}H_{13}NO_3$) $[M+Na]^+$ 266.0787; found 266.0792. R_f 0.34 (5% EtOAc/95% Pet Ether).

(9*H*-Fluoren-9-yl)methyl 2-acetyl-1*H*-pyrrole-1-carboxylate (16)

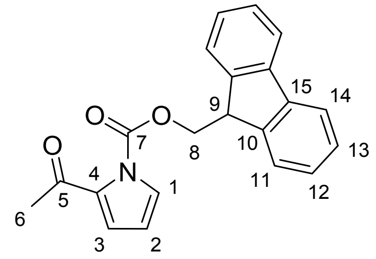

Purified *via* column chromatography (silica gel, 5% to 10% EtOAc/PetEther)

Reaction with TFAA: Reaction time: 18 h, white powder, 130 mg, 82%, m.p. 95-98 °C

Reaction with Tf_2O: Reaction time: 30 min, white powder, 132 mg, 83%

^{1}H NMR (400 MHz, $CDCl_3$) δ 7.78 (d, *J* = 7.6 Hz, 2H, H^{14}), 7.58 (d, *J* = 7.8 Hz, 2H, H^{11}), 7.42 (dd (app t), *J* = 7.5, 7.5 Hz, 2H, H^{12}), 7.32 (dd (app t), *J* = 7.5, 7.5 Hz, 2H, H^{13}), 7.23 (dd, *J* = 3.1, 1.6 Hz, 1H, H^1), 6.92 (dd, *J* = 3.7, 1.6 Hz, 1H, H^3), 6.21 (dd (app t), *J* = 3.6, 3.6 Hz, 1H, H^2), 4.70 (d, *J* = 6.7 Hz, 2H, H^8), 4.35 (t, *J* = 6.7 Hz, 1H, H^9), 2.45 (s, 3H, H^6). ^{13}C{^{1}H} NMR (101 MHz, $CDCl_3$) δ 188.4 (C^5), 150.4 (C^7), 143.3 (C^{10}), 141.5 (C^{15}), 134.7 (C^4), 128.2 (C^{13}), 128.0 (C^{12}), 127.4 (C^1), 125.1 (C^{11}), 122.2 (C^3), 120.3 (C^{14}), 110.9 (C^2), 69.9 (C^8), 46.8 (C^9), 28.0 (C^6); IR (neat) 3674, 2989, 2163, 1769, 1671, 1545, 1449, 1382, 1343, 1275, 1261, 880, 764, 750, 631, 557 cm^{-1} HRMS (ESI+) m/z calcd. for ($C_{21}H_{17}NO_3$) $[M+Na]^+$ 354.1100; found 354.1099. R_f 0.34 (5% EtOAc/95% Pet Ether).

2,2,2-Trichloroethyl 2-acetyl-1*H*-pyrrole-1-carboxylate (17)

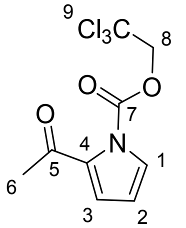

Purified *via* column chromatography (silica gel, 0% to 5% EtOAc/PetEther)

Reaction with TFAA - 18 h, 258 mg, 83%, yellow oil

Reaction with Tf_2O - 30 min, 76 mg, 92%, yellow oil

^{1}H NMR (400 MHz, $CDCl_3$) δ 7.45 (dd, *J* = 3.2, 1.6 Hz, 1H, H^1), 6.97 (dd, *J* = 3.6, 1.6 Hz, 1H, H^3), 6.27 (dd (app t), *J* = 3.4, 3.4 Hz, 1H, H^2), 4.96 (s, 2H, H^8), 2.49 (s, 3H, H^6). ^{13}C{^{1}H} NMR (101 MHz, $CDCl_3$) δ 188.2 (C^5), 148.8 (C^7), 134.9 (C^4), 128.2 (C^2), 122.9 (C^3), 111.5 (C^1), 94.1 (C^9), 76.5 (C^8), 28.0 (C^6); IR (neat) 3662, 3353, 2989, 2901, 1731, 1664, 1508, 1393, 1261, 1276, 1222, 1057, 897, 798, 751, 571 cm^{-1} HRMS (ESI+) m/z calcd. for ($C_9H_8Cl_3NO_3$) $[M+Na]^+$ 305.9462; found 305.9459. R_f 0.11 (5% EtOAc/95% Pet Ether)

Deprotections of Acetylated Pyrroles.

1-(1*H*-Pyrrol-2-yl)ethan-1-one (18)[8]

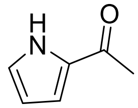

^1H NMR (400 MHz, $CDCl_3$) δ 7.04–6.99 (m, 1H), 6.94–6.89 (m, 1H), 6.30–6.26 (m, 1H), 2.43 (s, 3H).

Methoxy ester deprotection[9]

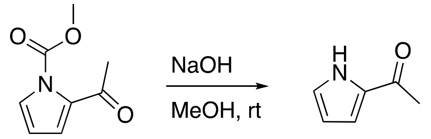

14 **18**

Scheme 1: Deprotection of compound **14**.

To a solution of **14** (150 mg, 0.90 mmol) in MeOH (6 mL) was added NaOH (179 mg, 4.5 mmol) and the reaction mixture was stirred at ambient temperature for 30 min. After that time, the reaction mixture was transferred to a separatory funnel and $NH_4Cl_{(sat)}$ solution was added until pH=7. The phases were separated, and the aqueous phase was extracted with CH_2Cl_2 (x 3). The combined organic phases were dried over $MgSO_4$ and filtered, then the filtrate was concentrated under vacuum to afford **18** as a colorless oil (89 mg, 91%).

Procedure for Fmoc deprotection[10]

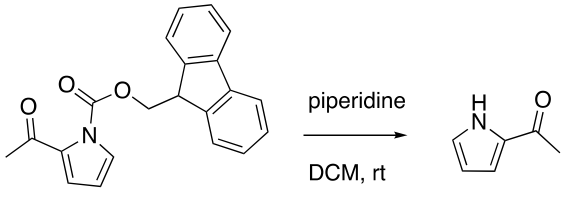

16 **18**

Scheme S2: Deprotection of compound **16**.

To a solution of compound **16** (173 mg, 0.75 mmol) in CH_2Cl_2 (3 mL) was added piperidine (580 mg, 6.8 mmol, 0.67 mL). The reaction mixture was stirred at ambient temperature and monitored by TLC until consumption of **16** (3 h). The reaction was diluted with H_2O (5 mL) and the organic phase was extracted with CH_2Cl_2 (20 mL). The combined organic phases were washed with brine (20 mL). The organic phase was dried over $MgSO_4$, filtered and the solvent was evaporated under vacuum. The mixture was purified by chromatography on silica (1% EtOAc/99% Pet Ether) to give **18** as a colourless oil (63 mg, 77%)

Preparation of Acylated *N*-Alkoxycarbonyl pyrroles from other carboxylic acids and their deprotection. The *General Procedure for Acylation reactions using Tf_2O* and the *General Procedure for N-Troc deprotection* in the main article were used to synthesize the following compounds:

2,2,2-Trichloroethyl 3-tetradecanoyl-1*H*-pyrrole-1-carboxylate (19)

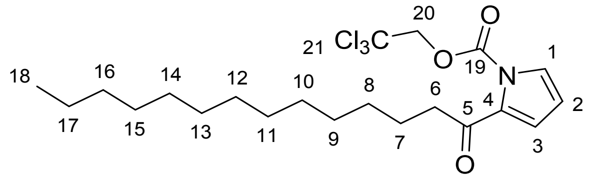

Reaction time: 1 h

Purified *via* column chromatography (silica gel, 0% to 1% EtOAc/99% Pet Ether)

White crystals, 88 mg, 81%, m.p. 66-68 °C

^{1}H NMR (400 MHz, $CDCl_3$) δ 7.44 (dd, *J* = 3.2, 1.5 Hz, 1H, H^1), 6.92 (dd, *J* = 3.6, 1.5 Hz, 1H, H^3), 6.26 (dd (app t), *J* = 3.4, 3.4 Hz, 1H, H^2), 4.95 (s, 2H, H^{20}), 2.78 (t, *J* = 7.5 Hz, 2H, H^6), 1.90 – 1.62 (m, 2H, H^7), 1.25 (m, 20H, H^{8-17}), 0.88 (t, *J* = 6.8 Hz, 3H, H^{18}). ^{13}C{^{1}H} NMR (101 MHz, $CDCl_3$) δ 191.5 (C^5), 148.8 (C^{19}), 134.8 (C^4), 127.8 (C^1), 121.8 (C^3), 111.3 (C^2), 94.0 (C^{21}), 76.4 (C^{20}), 40.4 (C^6), 31.9 (C^7), 29.78 (C^{8-17}), 29.77(C^{8-17}), 29.6 (C^{8-17}), 29.5 (C^{8-17}), 29.4 (C^{8-17}), 29.3 (C^{8-17}), 24.8 (C^{8-17}), 22.7(C^{8-17}), 14.1 (C^{18}); IR (neat) 3660, 3284, 3147, 3105, 2956, 2850, 1671, 1642, 1597, 1543, 1472, 1371, 1298, 1138, 1081. 917, 914, 670, 684 cm^{-1}. HRMS (ESI+) m/z calcd. for ($C_{21}H_{32}Cl_3NO_3$) $[M]^+$ 474.1340; found 474.1322. R_f 0.21 (1% EtOAc/99% Pet Ether)

1-(1*H*-Pyrrol-3-yl)tetradecan-1-one (20)

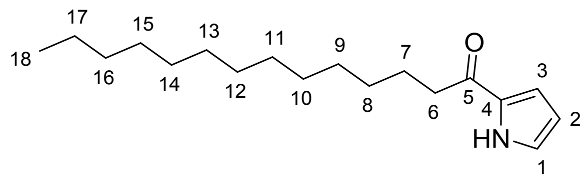

Reaction time: 3 h

White crystals, 25 mg, 95%, m.p. 64-67 °C

^{1}H NMR (400 MHz, $CDCl_3$) δ 9.35 (s, 1H, N-H), 7.01 (dd, *J* = 2.7, 1.3 Hz, 1H, H^1), 6.96 – 6.80 (m, 1H, H^3), 6.40 – 6.21 (m, 1H, H^2), 2.75 (t, *J* = 7.6 Hz, 2H, H^6), 1.77 – 1.66 (m, 2H, H7), 1.41 – 1.18 (m, 20H, H8-17), 0.88 (t, *J* = 6.7 Hz, 3H, H^{18}). ^{13}C{^{1}H} NMR (101 MHz, $CDCl_3$) δ 191.3 (C^5), 132.3 (C^4), 124.3 (C^3), 116.0 (C^1), 110.7 (C^2), 38.2 (C^6), 32.1 (C^{7-17}), 29.8 (C^{7-17}), 29.8 (C^{7-17}), 29.8 (C^{7-17}), 29.6 (C^{7-17}), 29.6 (C^{7-17}), 29.6 (C^{7-17}), 29.5 (C^{7-17}), 25.4 (C^{7-17}), 22.8 (C^{7-17}), 14.3 (C^{18}); IR (neat) 3283, 3150, 2954, 2916, 2850, 1671, 1641, 1597, 1546, 1404, 1314, 1087, 1135, 833, 761, 685, 585, 553 cm^{-1} HRMS (ESI+) m/z calcd. for ($C_{18}H_{31}NO$) $[M+H]^+$ 278.2479; found 278.2478. R_f 0.27 (1% EtOAc/99% Pet Ether)

2,2,2-Trichloroethyl 2-(4-chlorobutanoyl)-1*H*-pyrrole-1-carboxylate (21)

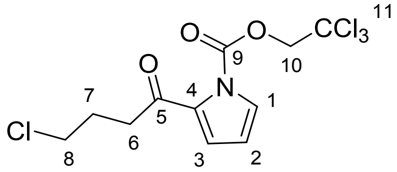

Reaction time: 2 h

Purified *via* column chromatography (silica gel, 0% to 5% EtOAc/Pet Ether)

White gum, 71 mg, 88%

^{1}H NMR (500 MHz, $CDCl_3$) δ 7.47 (dd, *J* = 3.2, 1.5 Hz, 1H, H^1), 7.00 (dd, *J* = 3.6, 1.5 Hz, 1H, H^3), 6.29 (dd (app t), *J* = 3.4, 3.4 Hz, 1H, H^2), 4.96 (s, 2H, H^{10}), 3.64 (t, *J* = 6.3 Hz, 2H, H^8), 3.01 (t, *J* = 7.0 Hz, 2H, H^6), 2.21 (m, 2H, H^7). $^{13}C\{^1H\}$ NMR (126 MHz, $CDCl_3$) δ 190.0 (C5), 148.8 (C9), 134.6 (C4), 128.3 (C1), 122.6 (C3), 111.7 (C2) , 94.1 (C11), 76.6 (C10), 44.6 (C8), 37.1 (C6), 27.2 (C7); IR (neat) 3289, 2974, 2162, 1742, 1706, 1636, 1471, 1402, 1293, 1257, 1007, 974, 739, 702 cm^{-1}. HRMS (ESI+) m/z calcd. for ($C_{11}H_{11}Cl_4NO_3$) $[M+H]^+$ 345.971; found 345.9563. R_f 0.14 (5% Et_2OAc/95% Pet Ether).

4-Chloro-1-(1*H*-pyrrol-2-yl)butan-1-one (22)

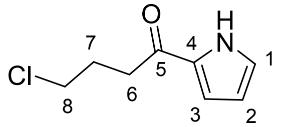

Reaction time: 3 h

Yellow oil, 33 mg, 91%

^{1}H NMR (400 MHz, $CDCl_3$) δ 9.77 (s, 1H, N-H), 7.05 (d, *J* = 2.7, 1.3 Hz, 1H, H1), 6.97 (dd, *J* = 3.8, 1.3 Hz, 1H, H3), 6.29 (dd, *J* = 3.9, 2.7 Hz, 1H, H2), 3.64 (t, *J* = 6.4 Hz, 2H, H^8), 2.98 (t, *J* = 7.2 Hz, 2H, H^6), 2.20 (tt, *J* = 7.1, 6.3 Hz, 2H, H7). $^{13}C\{^1H\}$ NMR (101 MHz, $CDCl_3$) δ 189.5 (C5), 131.9 (C4), 125.0 (C1), 116.7 (C3), 110.9 (C2), 44.8 (C^8), 34.8 (C^6), 27.6 (C7); IR (neat) 3289, 2974, 1742, 1706, 1636, 1471, 1402, 1293, 1257, 1007, 974, 739, 702 cm^{-1}. HRMS (ESI+) m/z calcd. for ($C_8H_{1o}ClNO$) $[M+H]^+$ 172.0524; found 172.0522. R_f 0.51 (10% EtOAc/90% Pet Ether).

^{1}H-NMR Data agree with previous literature reports.[11]

2,2,2-Trichloroethyl 2-(adamantane-1-carbonyl)-1*H*-pyrrole-1-carboxylate (23)

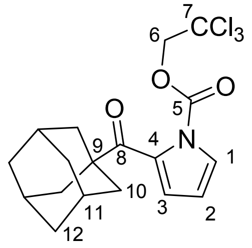

Reaction time: 3 h

Purified *via* column chromatography (silica gel, 0% to 2.5% EtOAc/Pet Ether)

Beige powder, 272 mg, 70%, m.p. 60-61.5 °C

^{1}H NMR (500 MHz, $CDCl_3$) δ 7.96 (dd (app t), *J* = 1.9, 1.9 Hz, 1H, H^1), 7.31-7.28 (m, 1H, H^3), 6.78 (dd, *J* = 3.4, 1.6 Hz, 1H, H^2), 5.00 (s, 2H, H^6), 2.22-2.06 (m, 3H, H^{11}), 2.02-1.93 (m, 6H, H^{10}), 1.90–1.70 (m, 6H, H^{12eq}+H^{12ax}). ^{13}C{^{1}H} NMR (126 MHz, $CDCl_3$) δ 201.5 (C^8), 148.8 (C^5), 126.7 (C^4), 124.1 (C^1), 120.3 (C^3), 114.5 (C^2), 109.4 (C^7), 76.2 (C^6), 46.8 (C^9), 39.5 (C^{10}), 36.9 (C^{12}), 28.4 (C^{11}). HRMS (ESI+) m/z calcd. for ($C_{18}H_{20}Cl_3NO_3$) $[M+H]^+$ 404.0582; found 404.0563; IR (neat) 3661, 3129, 2902, 1673, 1570, 1453, 1438, 1363, 1204, 1178, 1118, 1036, 840, 800, 754, 721 cm^{-1}. R_f 0.27 (5% EtOAc/95% Pet Ether).

(Adamantan-1-yl)(1*H*-pyrrol-2-yl)methanone (24)

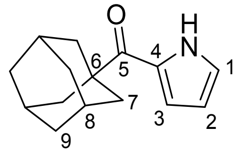

Reaction time: 2.5 h

Orange oil, 127 mg, 94%

^{1}H NMR (400 MHz, DMSO-d_6) δ 7.56 (dd, *J* = 3.4, 1.8 Hz, 1H, H^1), 6.80–6.72 (m, 1H, H^3), 6.63–6.44 (m, 1H, H^2), 2.05–2.00 (m, 3H, H^8), 2.00-1.95 (m, 6H, H^7), 1.79-1.66 (m, 6H, H^{9ax}+H^{9eq}). ^{13}C{^{1}H} NMR (101 MHz, DMSO-d_6) δ 200.4 (C^5), 124.2 (C^3), 122.2 (C^4), 118.9 (C^2), 109.9 (C^1), 45.8 (C^6), 40.0 (C^7), 36.8 (C^9), 28.3 (C^8); IR (neat) 3142, 3015, 2915, 2850, 1671, 1543, 1471, 1138, 1081, 804, 683 cm^{-1}. HRMS (ESI+) m/z calcd. for ($C_{15}H_{19}NO$) $[M+H]^+$ 230.1540; found 230.1545. R_f 0.27 (2.5% EtOAc/97.5% Pet Ether).

2,2,2-Trichloroethyl 2-(2,2-diphenylacetyl)-1*H*-pyrrole-1-carboxylate (25)

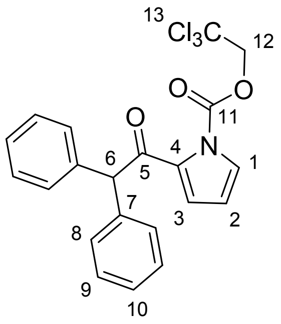

Reaction time: 2.5 h

Purified *via* column chromatography (silica gel, 0% to 2.5% EtOAc/Pet Ether)

White crystals, 83 mg, 79%, m.p. 74-77 °C

^{1}H NMR (400 MHz, $CDCl_3$) δ 7.37 (dd, *J* = 3.1, 1.5 Hz, 1H, H^1), 7.28–7.16 (m, 10H, H^{8-10}), 6.93 (dd, *J* = 3.7, 1.5 Hz, 1H, H^3), 6.17 (dd (app t), *J* = 3.4, 3.4 Hz, 1H, H^2), 5.66 (s, 1H, H^6), 4.88 (s, 2H, H^{12}). $^{13}C\{^1H\}$ NMR (126 MHz, $CDCl_3$) δ 188.6 (C^5), 149.1 (C^{11}), 139.0 (C^7), 134.5 (C^4), 129.3 ($C^{8/9}$), 128.78 ($C^{8/9}$), 128.77 (C^1), 127.3 (C^{10}), 123.0 (C^3), 111.4 (C^2), 94.2 (C^{13}), 76.7 (C^{12}), 60.8 (C^6); IR (neat) 2955, 1752, 1495, 1451, 1380, 1283, 1145, 1031, 745, 637 cm^{-1} HRMS (ESI+) m/z calcd. for ($C_{21}H_{16}Cl_3NO_3$) $[M+H]^+$ 436.0269; found 436.0274. R_f 0.17 (2.5% EtOAc/97.5% Pet Ether)

2,2-Diphenyl-1-(1*H*-pyrrol-2-yl)ethan-1-one (26)

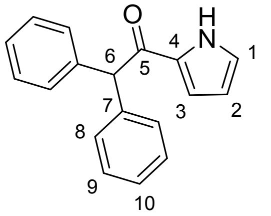

Reaction time: 2.5 h

White powder, 24 mg, 80%, m.p. 74-77 °C

^{1}H NMR (500 MHz, $CDCl_3$) δ 7.37–7.18 (m, 10H, H^{8-10}), 7.02 (dd, *J* = 2.5, 1.3 Hz, 1H, H^1), 6.97 (dd, *J* = 3.9, 1.4 Hz, 1H, H^3), 6.26 (dd, *J* = 3.9, 2.5 Hz, 1H, H^2), 5.76 (s, 1H, H^6). $^{13}C\{^1H\}$ NMR (126 MHz, $CDCl_3$) δ 188.5 (C^5), 139.5 (C^4), 132.1 (C^7), 129.1 ($C^{8/9}$), 128.7 ($C^{8/9}$), 127.2 (C^{10}), 125.1 (C^3), 117.1 (C^2), 111.1 (C^1), 58.9 (C^6); IR (neat) 2955, 1747, 1664, 1389, 1221, 1115, 714 cm^{-1} HRMS (ESI+) m/z calcd. for ($C_{18}H_{15}NO$) $[M+H]^+$ 262.1227; found 262.1233. R_f 0.21 (2.5% EtOAc/97.5% Pet Ether)

2,2,2-Trichloroethyl 2-(2-(4-isobutylphenyl)propanoyl)-1*H*-pyrrole-1-carboxylate (27)

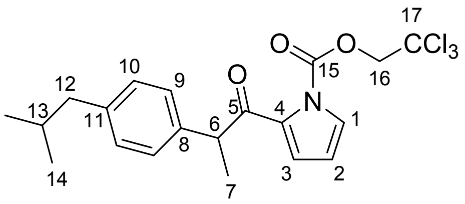

Reaction time: 1 h

Purified *via* column chromatography (silica gel, 0% to 10% EtOAc/Pet Ether)

White crystals, 167 mg, 81%, m.p. 86-89 °C

^{1}H NMR (400 MHz, $CDCl_3$) δ 7.31 (dd, *J* = 3.2, 1.5 Hz, 1H, H^1), 7.13 (d, *J* = 8.1 Hz, 2H, H^9), 6.99 (d, *J* = 8.1 Hz, 2H, H^{10}), 6.75 (dd, *J* = 3.6, 1.5 Hz, 1H, H^3), 6.11 (dd (app t), *J* = 3.4, 3.4 Hz, 1H, H^2), 4.89 (d, *J* = 11.8 Hz, 1H, H^{16a}), 4.84 (d, *J* = 11.8 Hz, 1H, H^{16b}), 4.25 (q, *J* = 6.9 Hz, 1H, H^6), 2.34 (d, *J* = 7.2 Hz, 2H, H^{12}), 1.81–1.67 (m, 1H, H^{13}), 1.44 (d, *J* = 7.0 Hz, 3H, H^7), 0.80 (d, *J* = 6.6 Hz, 6H, H^{14}). $^{13}C\{^1H\}$ NMR (101 MHz, $CDCl_3$) δ 191.5 (C^5), 149.0 (C^{15}), 140.6 (C^{11}), 138.4 (C^8), 134.3 (C^4), 129.7 (C^{10}), 128.0 (C^1), 127.7 (C^9), 122.4 (C^3), 111.5 (C^2), 94.1 (C^{17}), 76.6 (C^{16}), 49.6 (C^6), 45.2 (C^{12}), 30.3 (C^{13}), 22.5 (C^{14}), 18.8 (C^7); IR (neat) 3230, 2990, 2875, 1778, 1672, 1412, 1322, 1286, 1252, 1143, 922, 794, 662 cm^{-1} HRMS (ESI+) m/z calcd. for ($C_{20}H_{22}Cl_3NO_3$) $[M+H]^+$ 430.0738; found 430.0742. R_f 0.27 (5% EtOAc/95% Pet Ether).

2-(4-*iso*-Butylphenyl)-1-(1*H*-pyrrol-2-yl)propan-1-one (28)

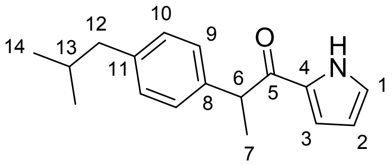

Reaction time: 2 h

White solid, 24 mg, 90%, m.p. 63-65 °C

^{1}H NMR (400 MHz, $CDCl_3$) δ 9.31 (s, 1H, N-H), 7.24 (d, *J* = 8.1 Hz, 2H, H^9), 7.07 (d, *J* = 8.1 Hz, 2H, H^{10}), 6.98 (dd, *J* = 2.7, 1.3 Hz, 1H, H^1), 6.91 (dd, *J* = 3.8, 1.3 Hz, 1H, H^2), 6.23 (dd, *J* = 3.9, 2.5 Hz, 1H, H^3), 4.39 (q, *J* = 7.0 Hz, 1H, H^6), 2.41 (d, *J* = 7.2 Hz, 2H, H^{12}), 1.88–1.73 (m, 1H, H^{13}), 1.52 (d, *J* = 7.0 Hz, 3H, H^7), 0.87 (d, *J* = 6.6 Hz, 6H, H^{14}). $^{13}C\{^1H\}$ NMR (101 MHz, $CDCl_3$) δ 191.3 (C^5), 140.4 (C^8), 139.0 (C^{11}), 131.5 (C^4), 129.6 (C^9), 127.5 (C^{10}), 124.6 (C^3), 116.6 (C^2), 110.8 (C^1), 47.3 (C^6), 45.2 (C^{12}), 30.2 (C^{13}), 22.55 (C^{14a}), 22.53 (C^{14b}) 18.8 (C^7). IR (neat) 3350, 2932, 1708, 1390, 1255, 1170, 1030, 714, 637 cm^{-1} HRMS (ESI+) m/z calcd. for ($C_{17}H_{21}NO$) $[M+H]^+$ 256.1705; found 256.1699. R_f 0.23 (5% EtOAc/95% Pet Ether).

(2-Acetyl-9*H*-fluoren-9-yl)methyl 2-acetyl-1*H*-pyrrole-1-carboxylate (29)

The *General Procedure for Acylation reactions using Tf_2O* was used.

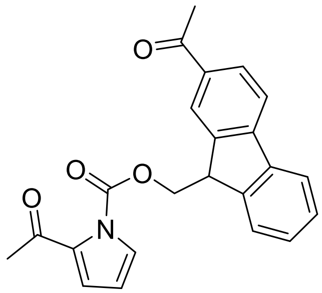

crude product

Reaction time: 4 h

Yellow oil, 91% crude yield

^{1}H NMR (500 MHz, d_6-acetone) δ 8.32 (s, 1H), 8.09–8.04 (m, 1H), 8.00–7.89 (m, 2H), 7.75 (d, *J* = 7.5 Hz, 1H), 7.45-7.44 (m, 1H), 7.43 (ddd, *J* = 7.4, 7.4, 1.4 Hz, 1H), 7.21 (dd, *J* = 3.3, 1.6 Hz, 1H), 7.02 (dd, *J* = 3.7, 1.6 Hz, 1H), 6.23 (dd (app t), *J* = 3.2 Hz, 1H), 4.89 (ddd, *J* = 10.7, 6.0, 2.3 Hz, 1H), 4.80 (dd, *J* = 10.7, 6.4 Hz, 1H), 4.50 (t, *J* = 6.1 Hz, 1H), 2.62 (s, 3H), 2.33 (s, 3H). ^{13}C{^{1}H} NMR (126 MHz, acetone-d_6) δ 197.6, 188.5, 151.2, 146.6, 145.6, 144.7, 141.1, 137.1, 129.4, 129.3, 129.0, 128.4, 126.1, 125.9, 124.7, 122.0, 120.8, 111.5, 69.8, 47.5, 27.7, 26.8; IR (neat) 3676, 3136, 2988, 2902, 1772, 1679, 1661, 1331, 1275, 1243, 791, 748 cm^{-1}. HRMS (ESI+) m/z calcd. for ($C_{23}H_{19}NO_4$) $[M+H]^+$ 374.1396; found 374.1393.

Preparation of Acylated *N*-sulfonyl pyrroles and their deprotection. The *General Procedure for Acylation reactions using Tf_2O* and the *General Procedure for N-Tosyl deprotection* in the main article were used to synthesize the following compounds:

2,2-Dimethyl-1-(1-tosyl-1*H*-pyrrol-3-yl)propan-1-one (31)

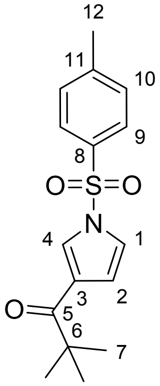

Reaction time: 6 h

Purified *via* column chromatography (silica gel, 0% to 2.5% EtOAc/ 96.5%Pet Ether)

Orange crystals, 205 mg, 69%, m.p. 86-89 °C

^{1}H NMR (400 MHz, $CDCl_3$) δ 7.79 (d, *J* = 8.4 Hz, 2H, H^9), 7.75 (dd (app t), *J* = 1.9, 1.9 Hz, 1H, H^1), 7.32 (d, *J* = 8.7 Hz, 2H, H^{10}), 7.10 (dd, *J* = 3.3, 2.2 Hz, 1H, H^4), 6.71 (dd, *J* = 3.3, 1.7 Hz, 1H, H^2), 2.41 (s, 3H, H^{12}), 1.28 (s, 9H, H^7). $^{13}C\{^1H\}$ NMR (101 MHz, $CDCl_3$) δ 201.2 (C^5), 145.9 (C^8), 135.3 (C^{11}), 130.4 (C^{10}), 127.2 (C^9), 126.2 (C^3), 124.4 (C^1), 120.5 (C^4), 114.2 (C^2), 43.9 (C^6), 27.8 (C^7), 21.7 (C^{12}); IR (neat) 3148, 2971, 1740, 1656, 1467, 1381, 1169, 810 cm^{-1}. HRMS (ESI+) m/z calcd. for ($C_{16}H_{19}NO_3S$) [M+H]$^+$; 306.1159, found 306.1168. R_f 0.31 (2.5% EtOAc/95% Pet Ether).

2,2-Dimethyl-1-(1*H*-pyrrol-3-yl)propan-1-one (32)

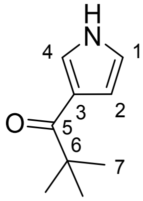

Reaction time: 20 h

Yellow oil, 145 mg, 81%

^{1}H NMR (400 MHz, $CDCl_3$) δ 7.47 (dd (app t), *J* = 1.8, 1.8 Hz, 1H, H^2), 6.75 (dd, *J* = 3.1, 2.0 Hz, 1H, H^1), 6.72 (dd, *J* = 3.1, 1.6 Hz, 1H, H^4), 1.34 (s, 9H, H^7). $^{13}C\{^1H\}$ NMR (101 MHz, $CDCl_3$) δ 202.6 (C^5), 123.8 (C^2), 123.0 (C^3), 118.3 (C^1), 110.4 (C^4), 43.8 (C^6), 28.3 (C^7); IR (neat) 3232, 2922, 1620, 1438, 1169, 1069, 755 cm^{-1}. HRMS (ESI+) m/z calcd. for ($C_9H_{14}NO$) [M+H]$^+$; 152.1075, found 152.1072. R_f 0.24 (2.5% EtOAc/95% Pet Ether).

2-(4-*iso*-Butylphenyl)-1-(1-tosyl-1*H*-pyrrol-3-yl)propan-1-one (33)

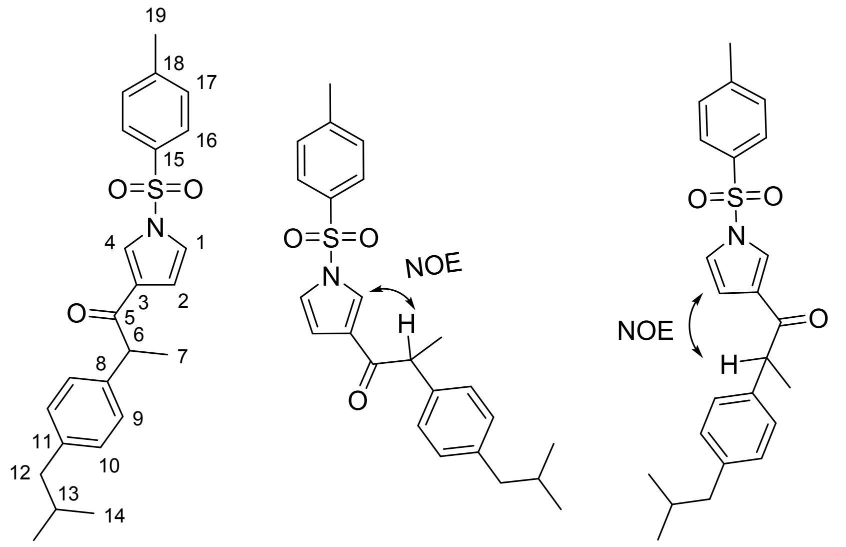

Reaction time: 6 h

Purified *via* column chromatography (silica gel, 0% to 2.5% EtOAc/ 97.5%Pet Ether)

Yellow powder, 294 mg, 74%, m.p. 98-102 °C

^{1}H NMR (400 MHz, $CDCl_3$) δ 7.68 (d, *J* = 8.4 Hz, 2H, H^{16}), 7.63 (dd (app t), *J* = 1.9, 1.9 Hz, 1H, H^{2}), 7.31–7.24 (m, 2H, H^{17}), 7.15 (d, *J* = 8.1 Hz, 2H, H^{9}), 7.06 (d, *J* = 8.1 Hz, 2H, H^{10}), 7.01 (dd, *J* = 3.3, 2.1 Hz, 1H, H^{1}), 6.61 (dd, *J* = 3.4, 1.6 Hz, 1H, H^{4}), 4.24 (q, *J* = 6.9 Hz, 1H, H^{6}), 2.42 (d, *J* = 7.1 Hz, 2H, H^{12}), 2.40 (s, 3H, H^{19}), 1.90–1.76 (m, 1H, H^{13}), 1.45 (d, *J* = 6.9 Hz, 3H, H^{7}), 0.89 (d, *J* = 6.6 Hz, 3H, H^{14a}), 0.87 (d, *J* = 6.6 Hz, 3H, H^{14b}). ^{13}C{^{1}H} NMR (101 MHz, $CDCl_3$) δ 195.9 (C^{5}), 145.9 (C^{15}), 140.6 (C^{11}), 138.7 (C^{8}), 135.3 (C^{18}), 130.4 (C^{17}), 129.8 (C^{10}), 128.4 (C^{3}), 127.6 (C^{9}), 127.3 (C^{16}), 125.0 (C^{2}), 121.3 (C^{1}), 113.2 (C^{4}), 49.5 (C^{6}), 45.2 (C^{12}), 30.3 (C^{13}), 22.54 (C^{14a}), 22.53 (C^{14b}), 21.8 (C^{19}), 19.0 (C^{7}); IR (neat) 3323, 2961. 2256, 1756, 1605, 1517, 1435, 1380, 1172, 1235, 907, 796, 724, 657cm^{-1}. HRMS (ESI+) m/z calcd. for ($C_{24}H_{27}NO_3S$) $[M+Na]^+$ 432.1604; found 432.1576. R_f 0.27 (2.5% Et_2OAc/95% Pet Ether).

2-(4-*iso*-Butylphenyl)-1-(1*H*-pyrrol-3-yl)propan-1-one (34)

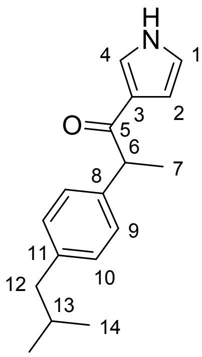

Reaction time: 18 h

White powder, 26 mg, 86%, m.p. 60-64°C

^{1}H NMR (400 MHz, $CDCl_3$) δ 7.37 (dd (app t), *J* = 1.7, 1.7 Hz, 1H, H^4), 7.22 (d, *J* = 8.2 Hz, 2H, H^{10}), 7.06 (d, *J* = 8.1 Hz, 2H, H^9), 6.70 (dd, *J* = 3.0, 1.8 Hz, 1H, H^2), 6.64 (dd, *J* = 3.1, 1.6 Hz, 1H, H^1), 4.33 (q, *J* = 6.9 Hz, 1H, H^6), 2.41 (d, *J* = 7.1 Hz, 2H, H^{12}), 1.70-1.89 (m, 1H, H^{13}), 1.51-1.45 (m, 3H, H^7), 0.87 (d, *J* = 6.6 Hz, 6H, H^{14}). ^{13}C{^{1}H} NMR (101 MHz, $CDCl_3$) δ 196.6 (C^5), 140.2 (C^8), 129.6 (C^9), 127.56 (C^{10}), 127.53 (C^{11}), 125.6 (C^3), 123.5 (C^4), 119.0 (C^2), 109.5 (C^1), 49.1 (C^6), 45.2 (C^{12}), 30.3 (C^{13}), 22.6 (C^{14a}), 22.5 (C^{14b}), 19.1 (C^7); IR (neat) 3147, 2966, 2926, 1726, 1659, 1596, 1494, 1470, 1371, 1171, 1070, 898, 811 cm^{-1}. HRMS (ESI+) m/z calcd. for ($C_{17}H_{21}NO$) $[M+H]^+$ 256.1696; found 256.1694. R_f 0.25 (5% EtOAc/95% Pet Ether)

2,2-Diphenyl-1-(1-tosyl-1*H*-pyrrol-3-yl)ethan-1-one (35)

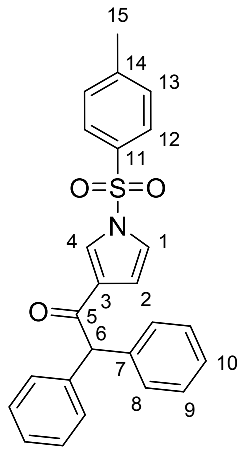

Reaction time: 16 h

Purified *via* column chromatography (silica gel, 0% to 5% EtOAc/ 95%Pet Ether)

Beige solid, 136 mg, 68%, m.p. 106-109 °C

^{1}H NMR (400 MHz, $CDCl_3$) δ 7.72–7.64 (m, 3H, H^4 and H^{12} or H^{13}), should this be 5H? 7.40–7.16 (m, 12H, $H^{8,9,10}$, and H^{12} or H^{13}), 7.04 (dd, *J* = 3.4, 2.2 Hz, 1H, H^1), 6.65 (dd, *J* = 3.4, 1.7 Hz, 1H, H^2), 5.61 (s, 1H, H^6), 2.42 (s, 3H, H^{15}). $^{13}C\{^1H\}$ NMR (101 MHz, $CDCl_3$) δ 193.2 (C^5), 145.9, 138.9, 135.0, 130.3, 129.1, 128.72, 128.68, 127.21 ($C^{12/C13}$), 127.16 ($C^{12/13}$), 125.1 (C^4), 121.4 (C^1), 113.1 (C^2), 61.1 (C^6), 21.7 (C^{15}). IR (neat) 3284, 3029, 1725, 1675, 1597, 1393, 1372, 1171, 1066, 822, 699, 670 cm^{-1}. HRMS (ESI+) m/z calcd. for ($C_{25}H_{21}NO_3S$) [M+H]$^+$ 416.1315; found 416.1324. R_f 0.50 (5% EtOAc/95% Pet Ether)

2,2-Diphenyl-1-(1H-pyrrol-3-yl)ethan-1-one (36)

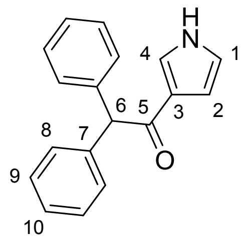

Reaction time: 20 h

White powder, 26 mg, 87%, m.p. 88-90 °C

^{1}H NMR (400 MHz, $CDCl_3$) δ 7.45–7.43 (m, 1H, H^4), 7.38–7.09 (m, 10H, $H^{8,9,10}$), 6.75–6.71 (m, 1H, H^2), 6.71–6.66 (m, 1H, H^1), 5.71 (s, 1H, H^6). $^{13}C\{^1H\}$ NMR (101 MHz, $CDCl_3$) δ 193.8 (C^5), 139.9, 129.3, 129.1, 128.7, 127.0, 124.0 (C^4), 119.4 (C^2), 109.8 (C^1), 60.9 (C^6). IR (neat) 3298, 2919, 2850, 1721, 1643, 1494, 1245, 1080, 729, 695 cm^{-1}. HRMS (ESI+) m/z calcd. for ($C_{18}H_{15}NO$) [M+H]$^+$ 262.1227; found 262.1228. R_f 0.23 (5% EtOAc/95% Pet Ether)

2,2,2-Trichloroethyl carbamate

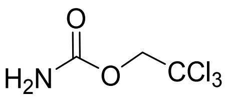

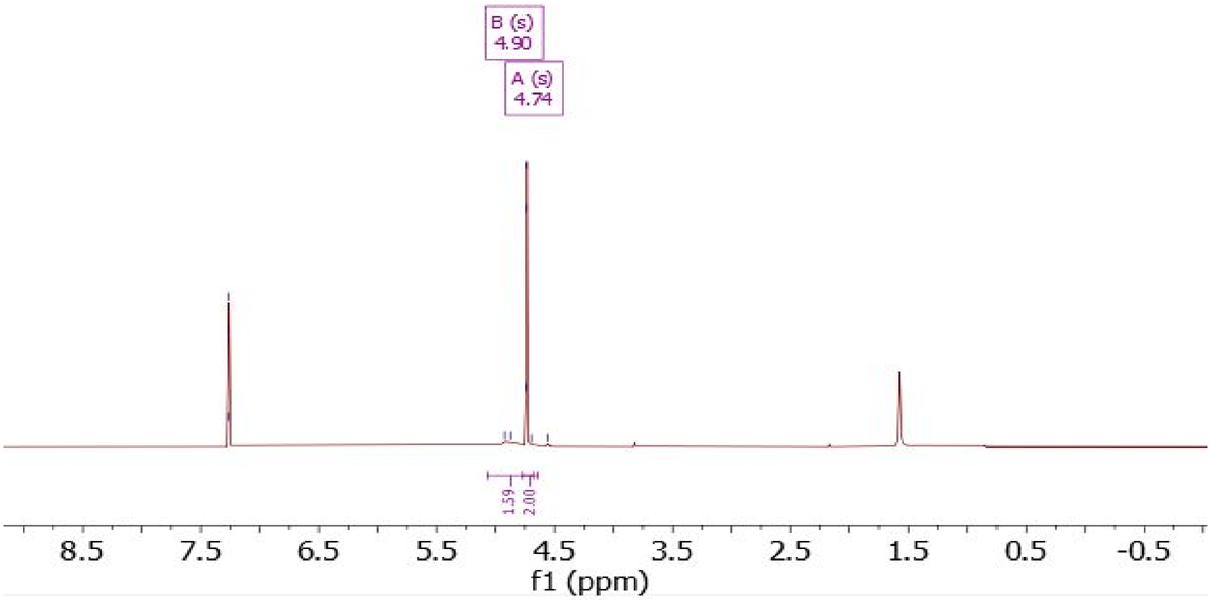

Fig S1: ^{1}H NMR (400 MHz) spectrum of 2,2,2-Trichloroethyl carbamate in $CDCl_3$

Allyl carbamate

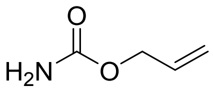

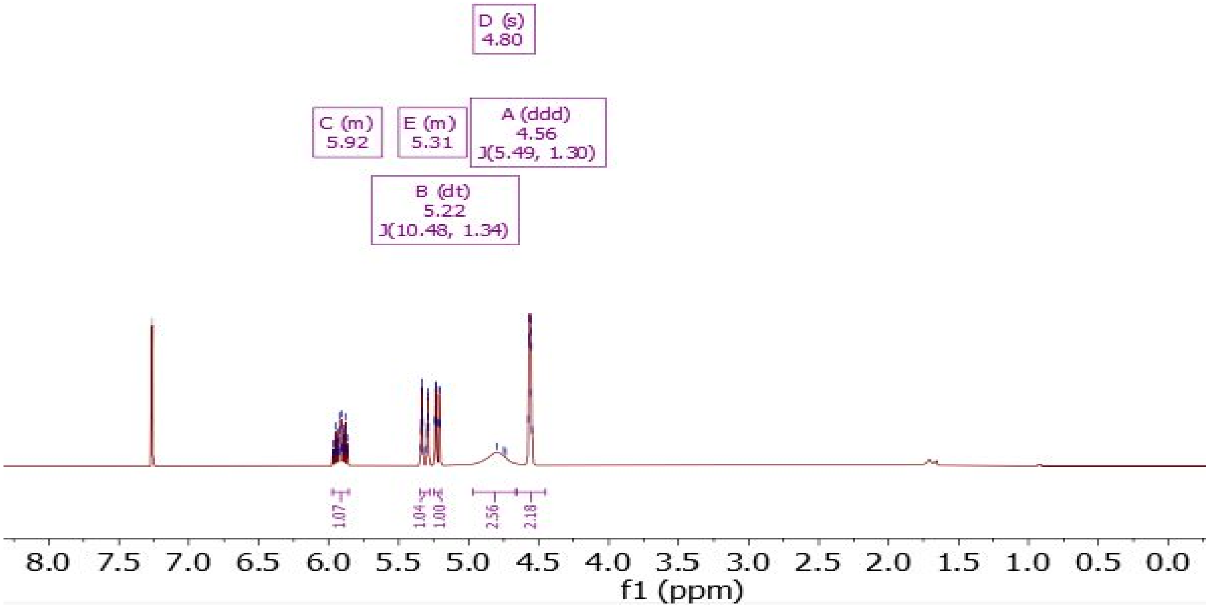

Fig S2: ^{1}H NMR (400 MHz) spectrum of allyl carbamate in $CDCl_3$

2-(Trimethylsilyl)ethyl carbamate

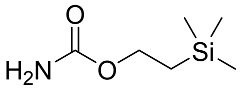

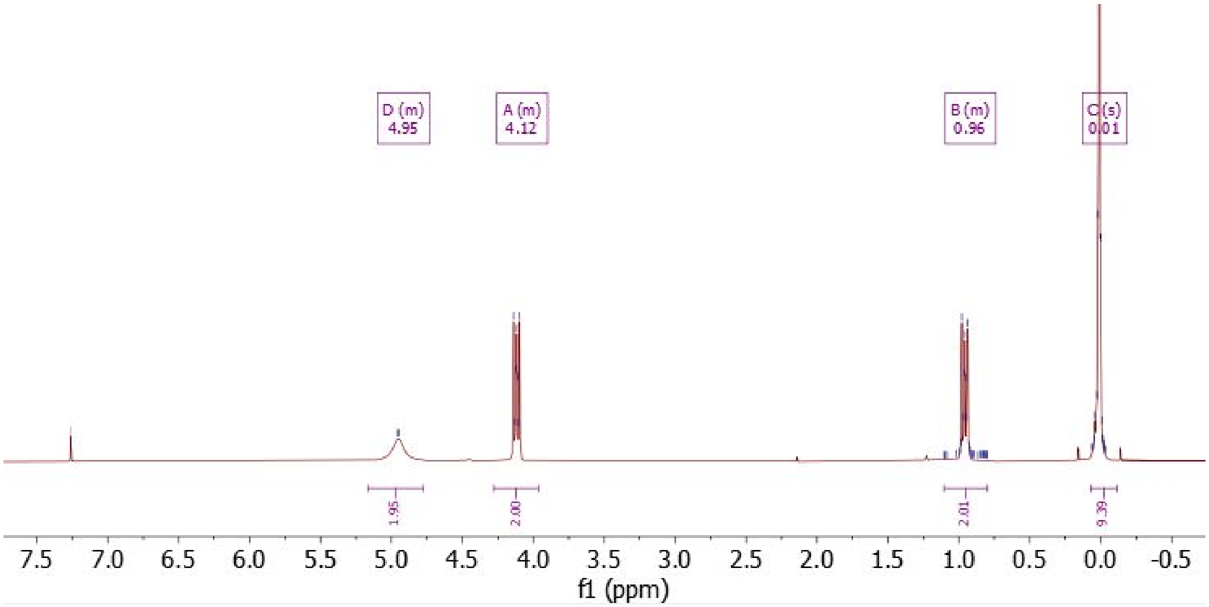

Fig S3: ^{1}H NMR (400 MHz) spectrum of 2-(trimethylsilyl)ethyl carbamate in $CDCl_3$

Methyl 1*H*-pyrrole-1-carboxylate (8)

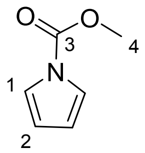

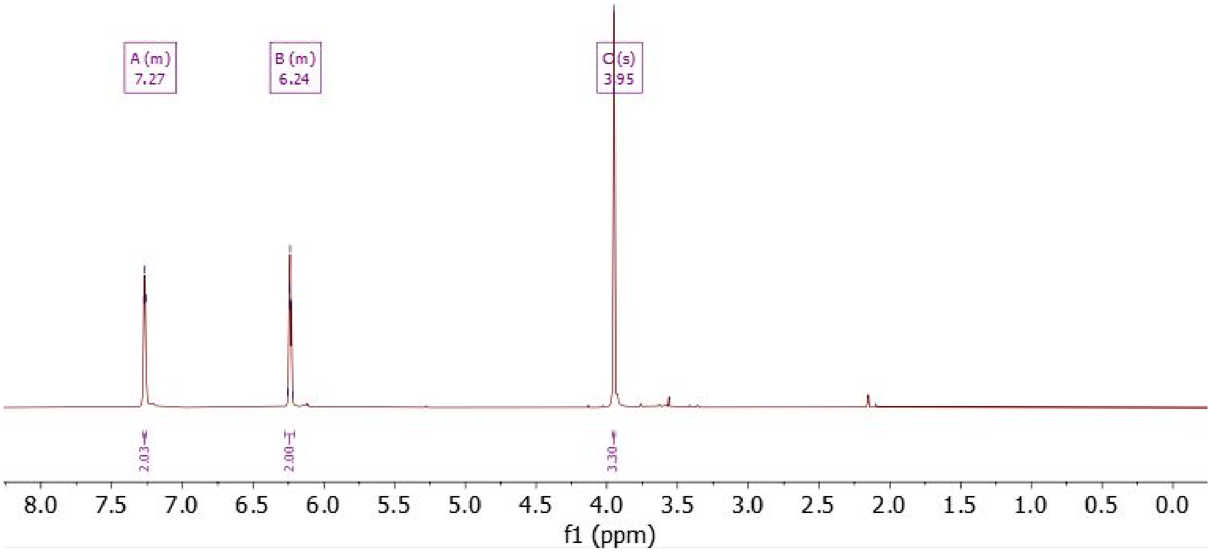

Fig S4: ^{1}H NMR spectrum of methyl 1*H*-pyrrole-1-carboxylate (**8**) in $CDCl_3$

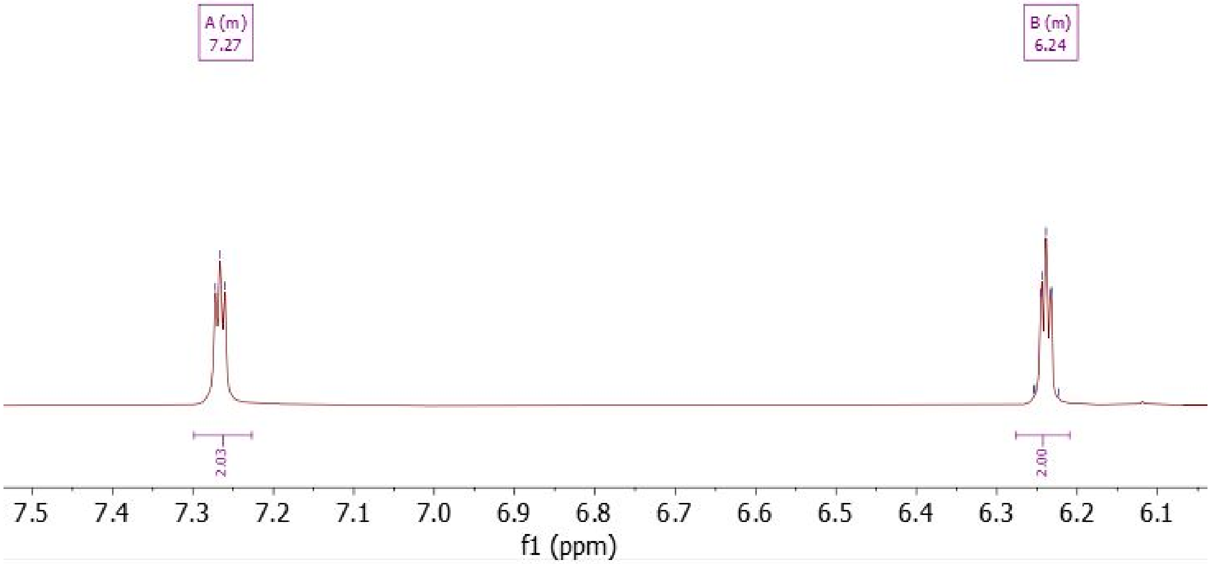

Fig S5: Zoomed region ^{1}H NMR (400 MHz) spectrum of methyl 1*H*-pyrrole-1-carboxylate (**8**) in $CDCl_3$

Benzyl 1*H*-pyrrole-1-carboxylate (9)

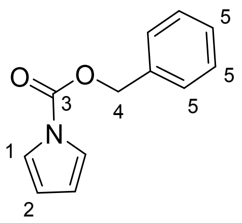

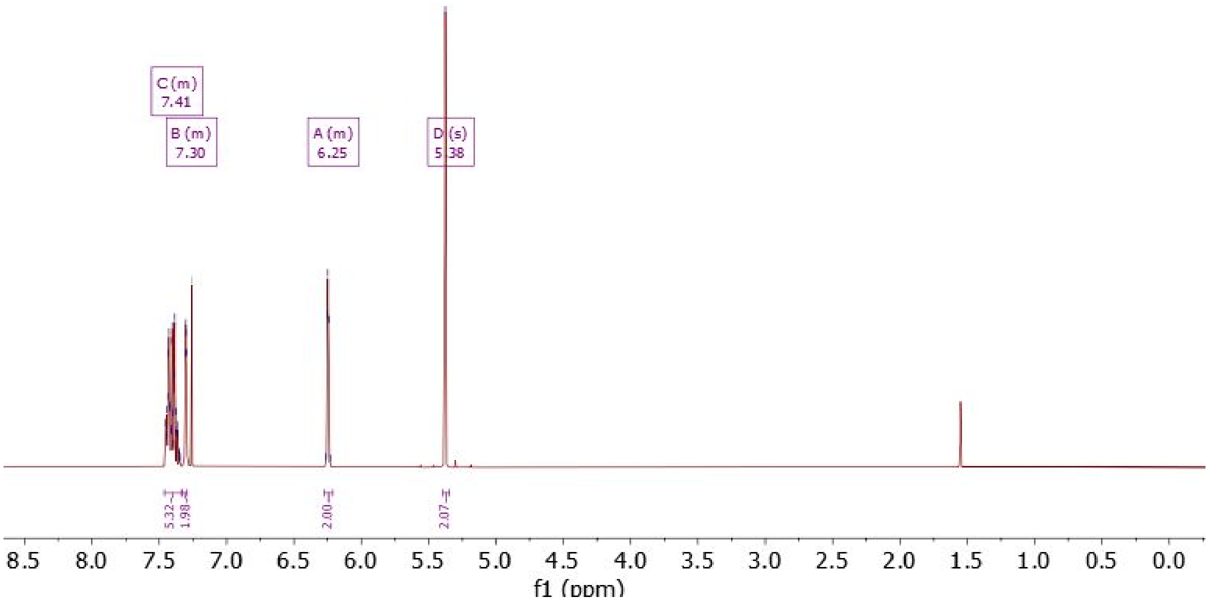

Fig S6: ^{1}H NMR (400 MHz) spectrum of benzyl 1*H*-pyrrole-1-carboxylate (**9**) in $CDCl_3$

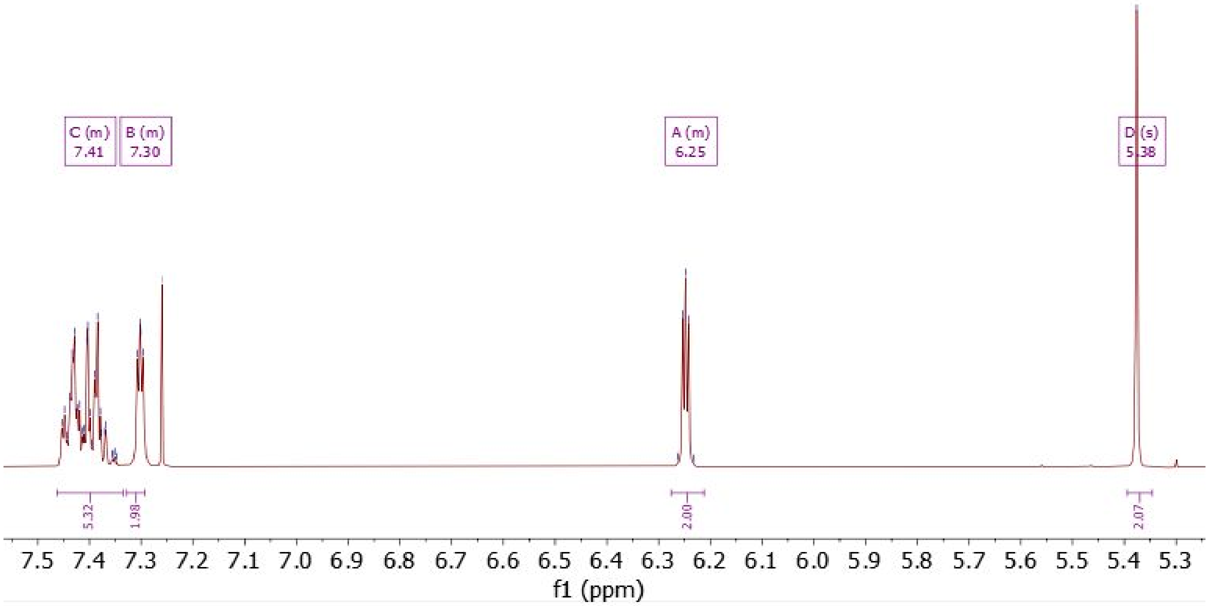

Fig S7: Zoomed region of ^{1}H NMR (400 MHz) spectrum of benzyl 1*H*-pyrrole-1-carboxylate (**9**) in $CDCl_3$

2-(Trimethylsilyl)ethyl 1*H*-pyrrole-1-carboxylate (10)

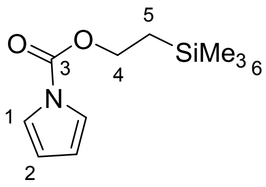

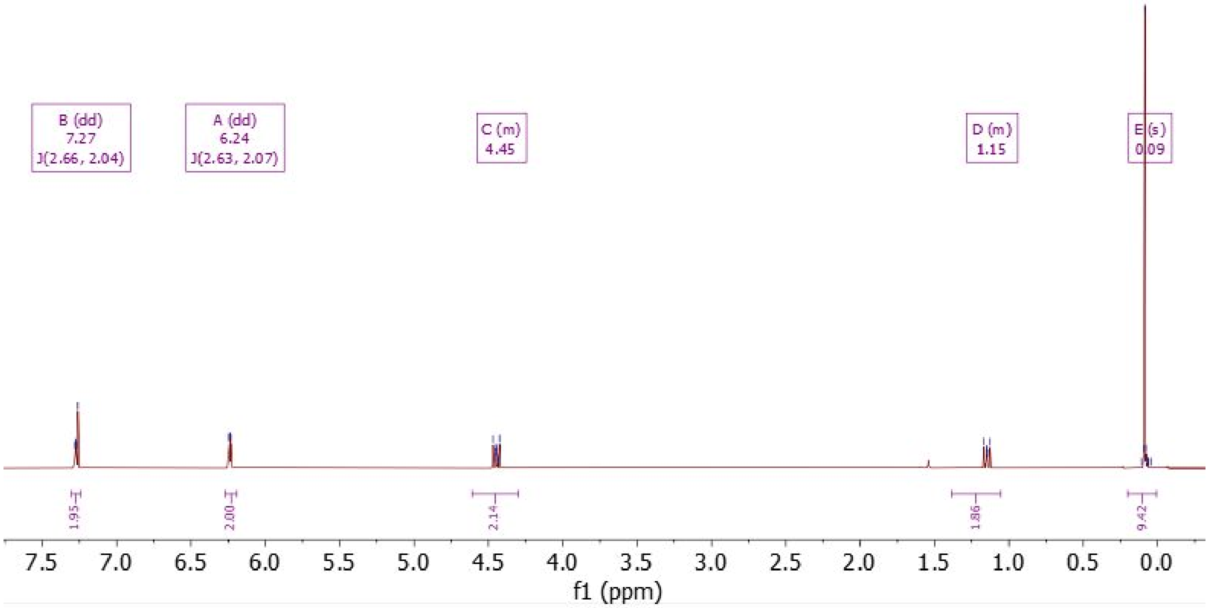

Fig S8: ^{1}H NMR (400 MHz) spectrum of 2-(trimethylsilyl)ethyl 1*H*-pyrrole-1-carboxylate (**10**) in $CDCl_3$

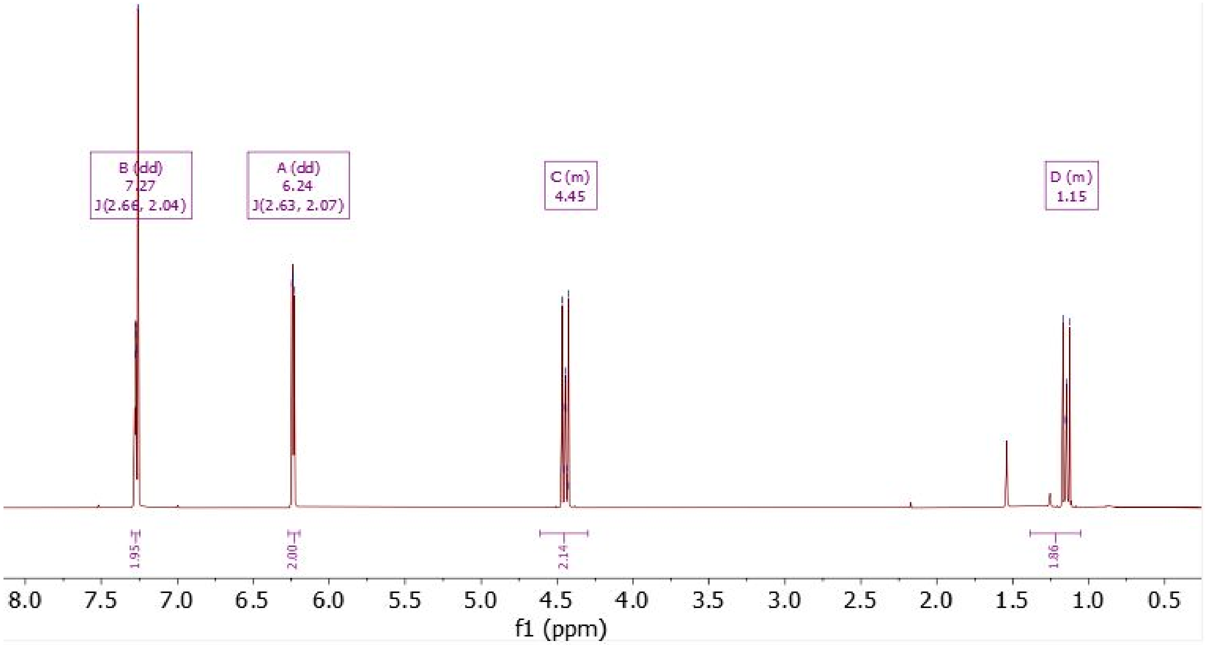

Fig S9: Zoomed region of ^{1}H NMR (400 MHz) spectrum of 2-(trimethylsilyl)ethyl 1*H*-pyrrole-1-carboxylate (**10**) in $CDCl_3$

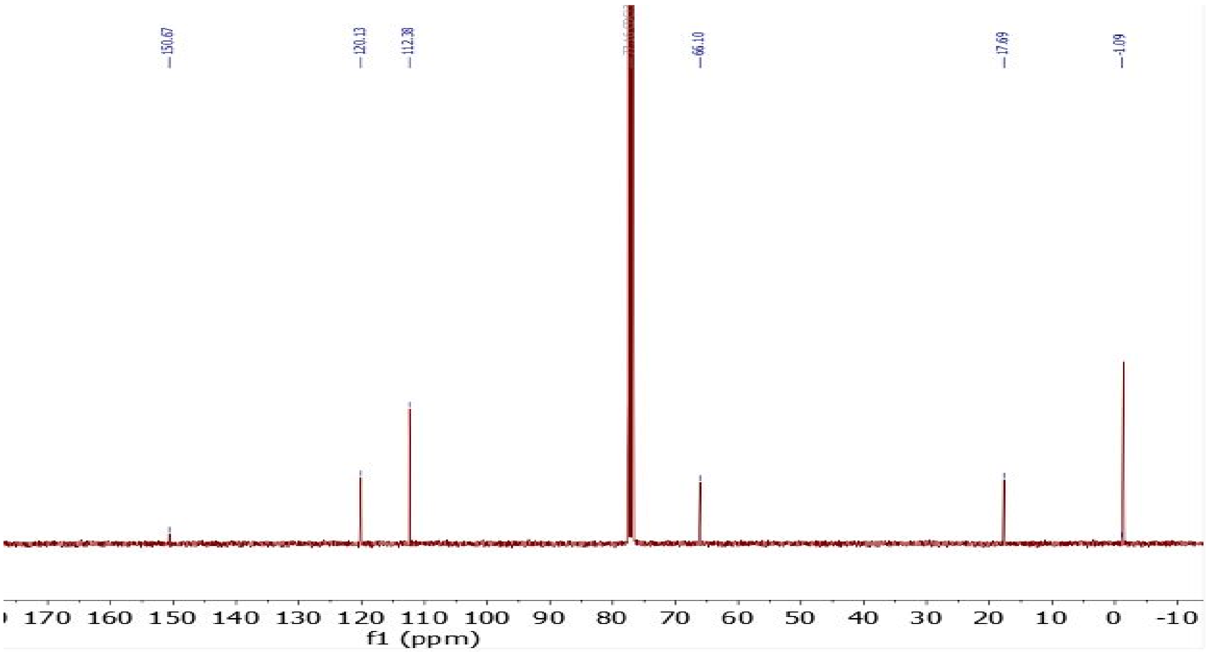

Fig S10: $^{13}C\{^{1}H\}$ NMR (101 MHz) spectrum of 2-(trimethylsilyl)ethyl 1*H*-pyrrole-1-carboxylate (**10**) in $CDCl_3$

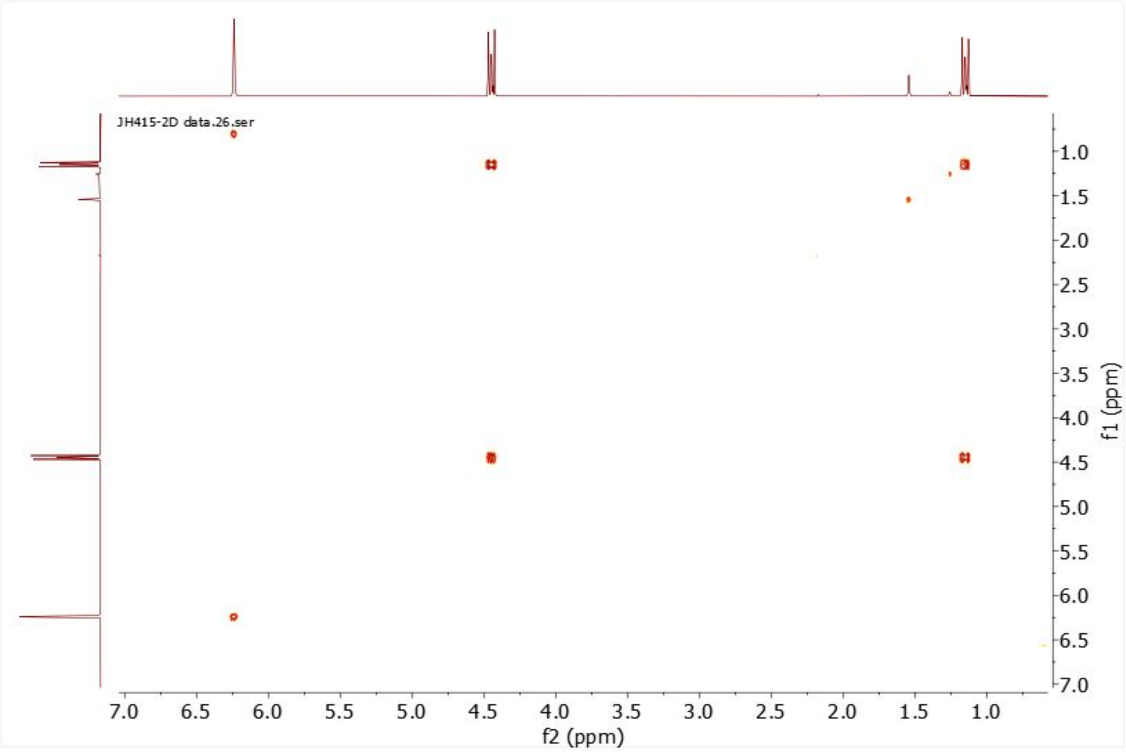

Fig S11: COSY NMR spectrum of 2-(trimethylsilyl)ethyl 1*H*-pyrrole-1-carboxylate (**10**) in $CDCl_3$

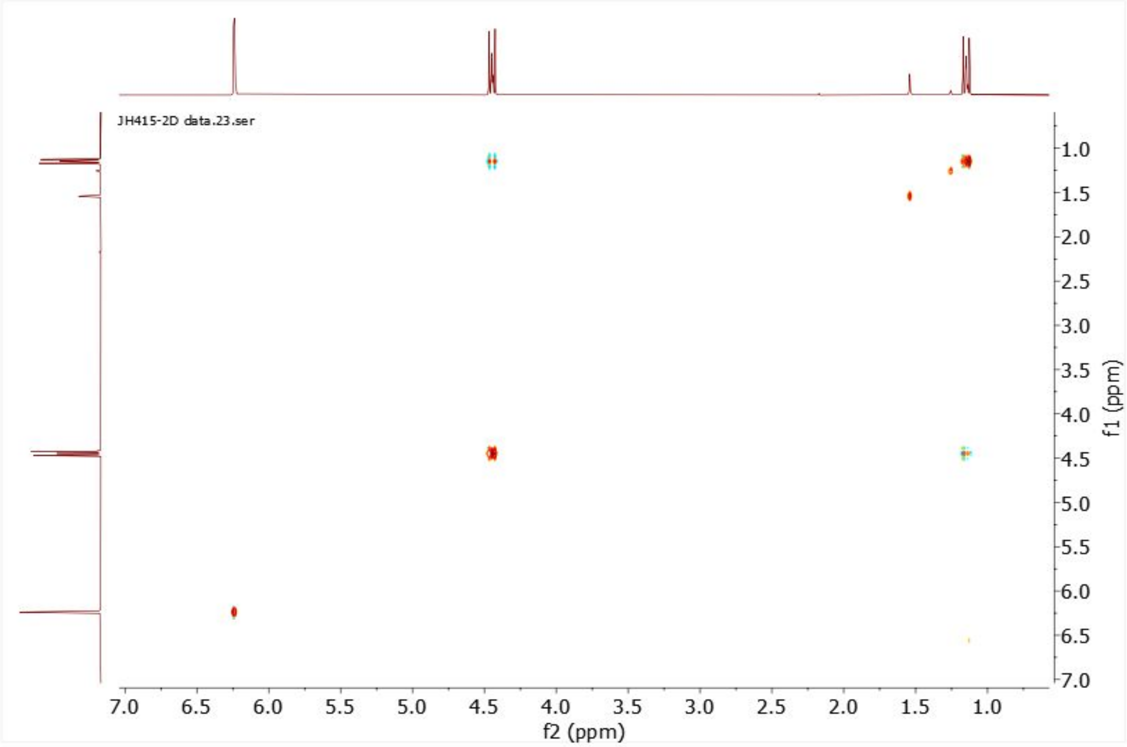

Fig S12: NOESY NMR spectrum of 2-(trimethylsilyl)ethyl 1*H*-pyrrole-1-carboxylate (**10**) in $CDCl_3$

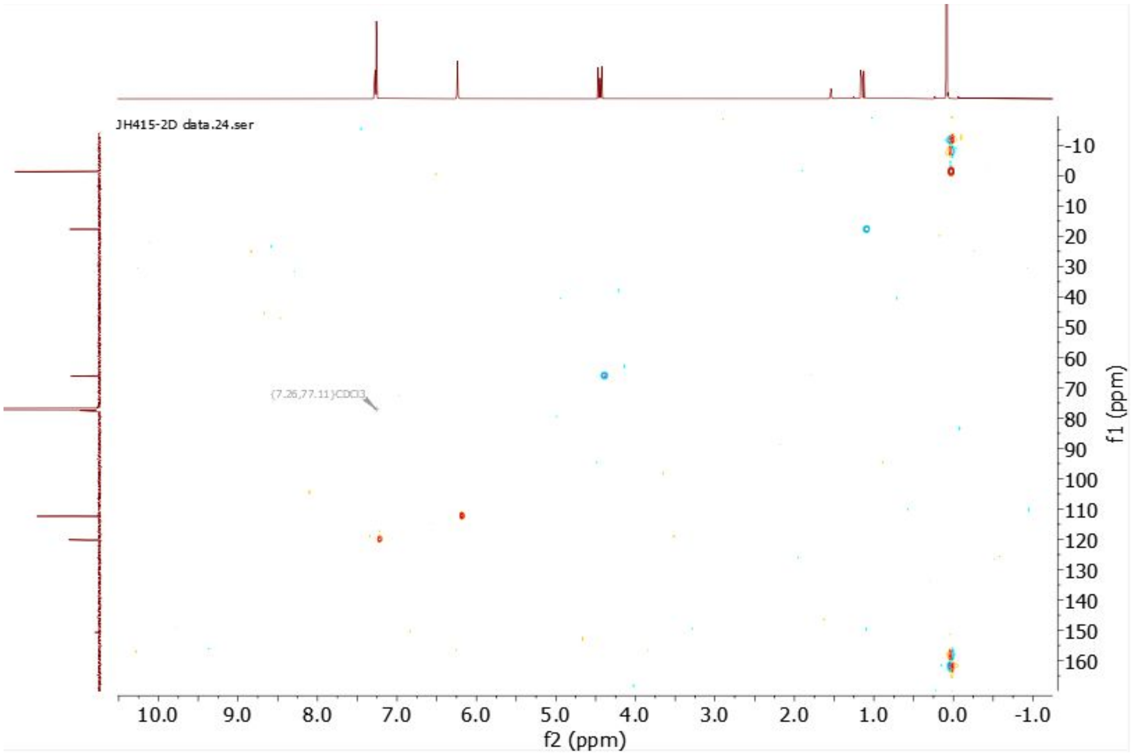

Fig S13: HSQC NMR spectrum of 2-(trimethylsilyl)ethyl 1*H*-pyrrole-1-carboxylate (**10**) in $CDCl_3$

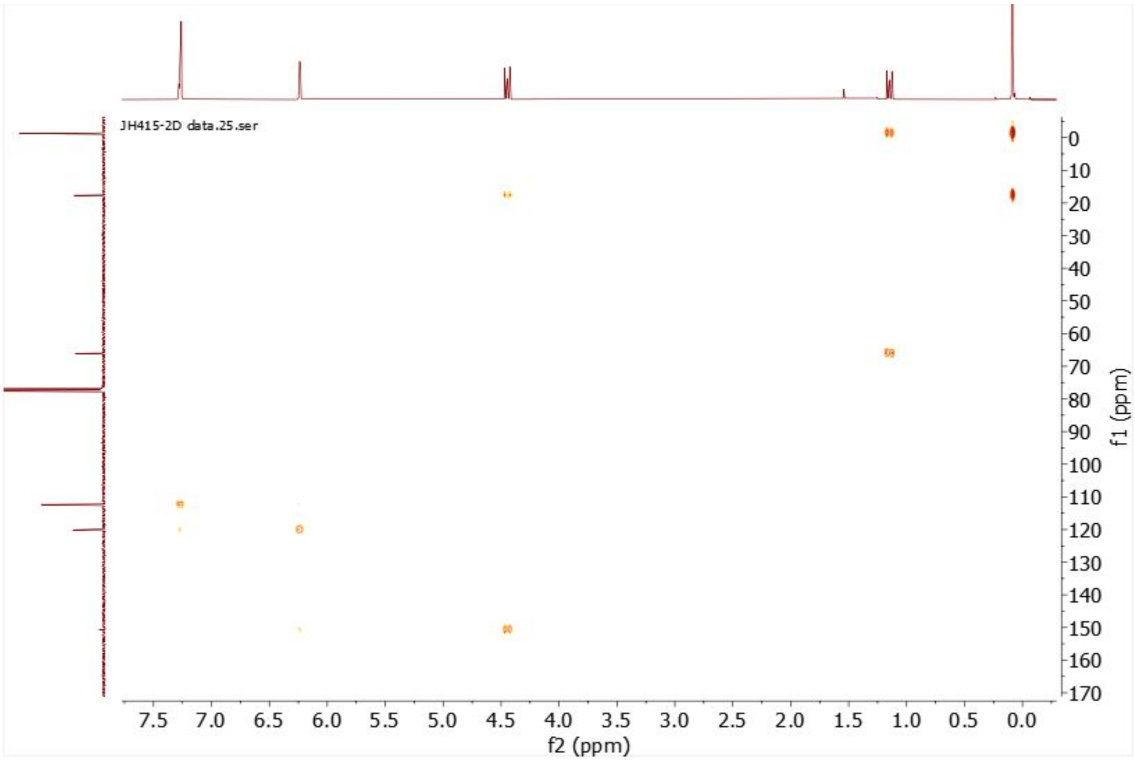

Fig S14: HMBC NMR spectrum of 2-(trimethylsilyl)ethyl 1*H*-pyrrole-1-carboxylate (**10**) in $CDCl_3$

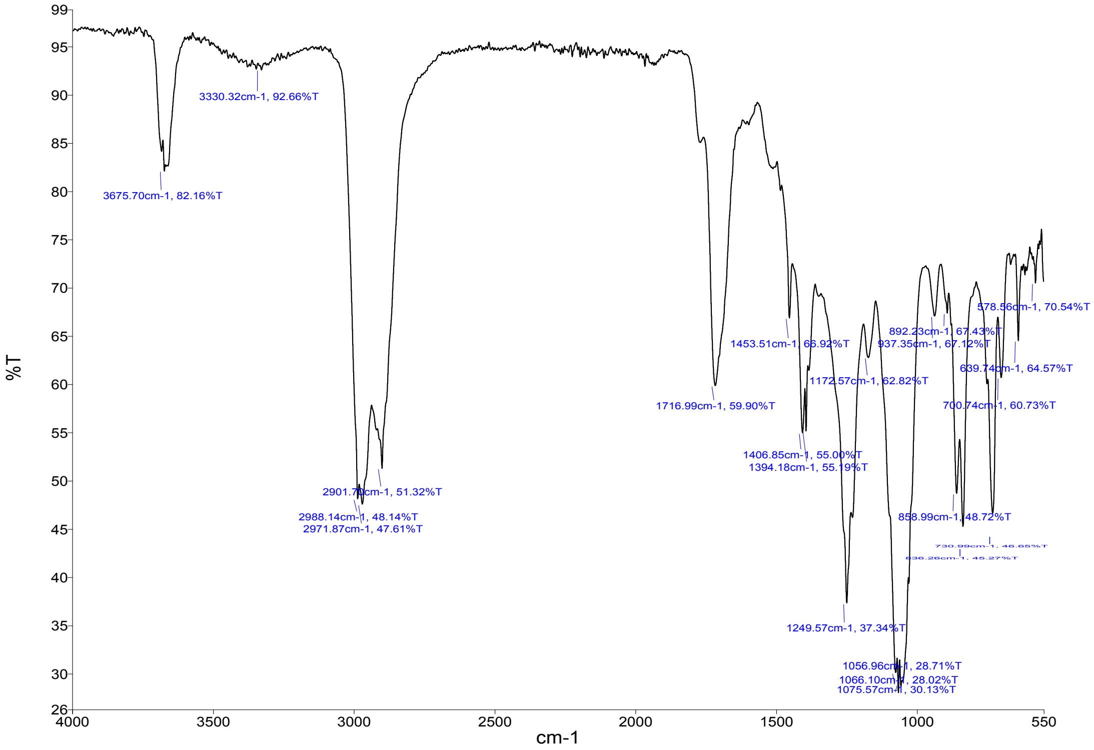

Fig S15. IR (neat) spectrum of 2-(trimethylsilyl)ethyl 1*H*-pyrrole-1-carboxylate (**10**)

Allyl 1*H*-pyrrole-1-carboxylate (11)

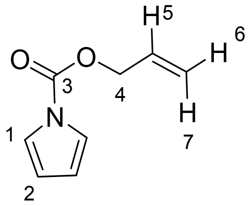

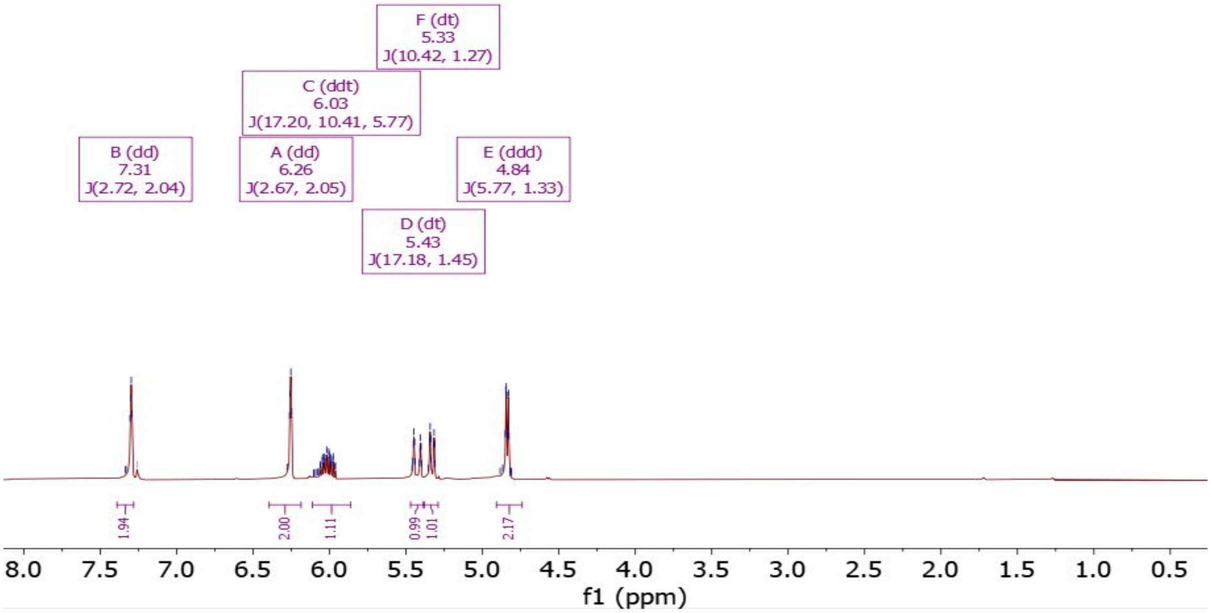

Fig S16: ^{1}H NMR (400 MHz) spectrum of allyl 1*H*-pyrrole-1-carboxylate (**11**) in $CDCl_3$

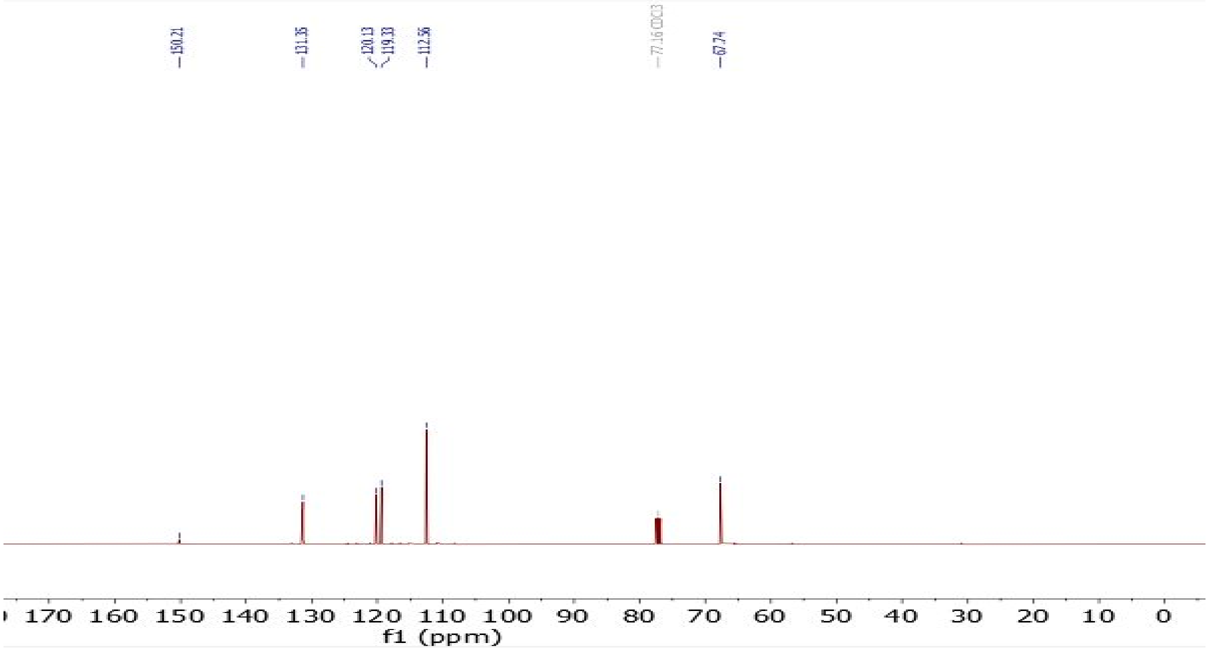

Fig S17: $^{13}C\{^{1}H\}$ NMR (101 MHz) spectrum of allyl 1*H*-pyrrole-1-carboxylate (**11**) in $CDCl_3$

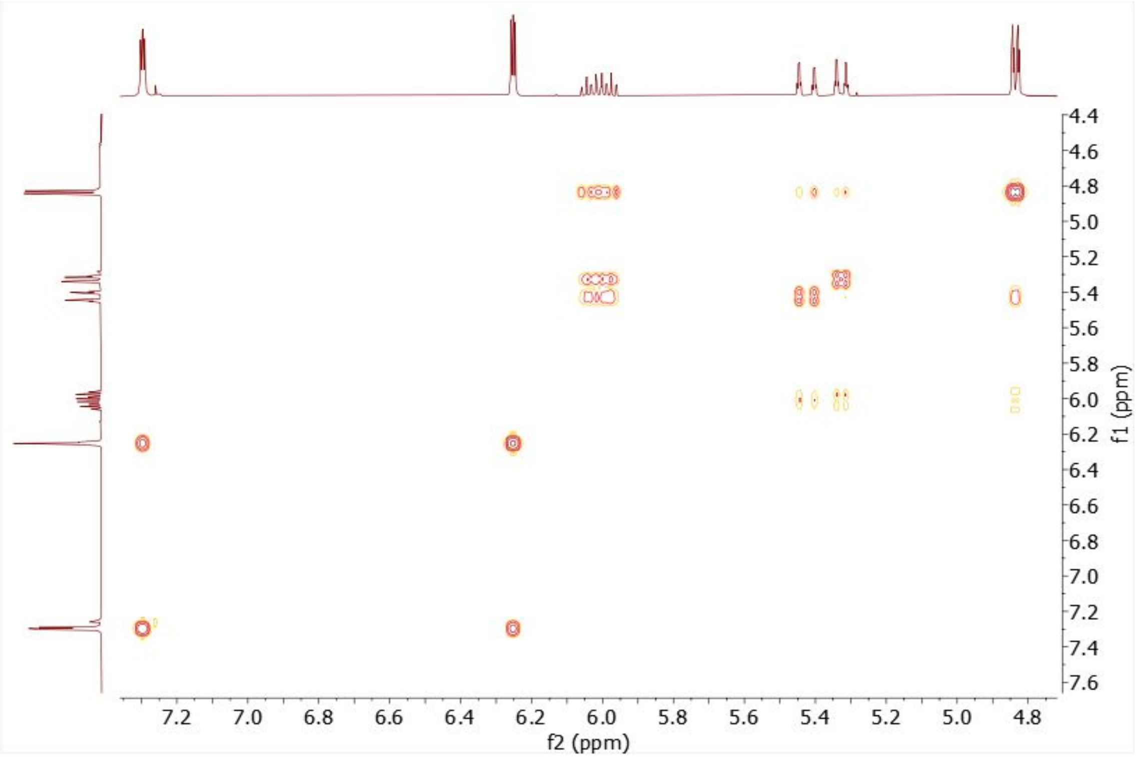

Fig S18: COSY NMR spectrum of allyl 1*H*-pyrrole-1-carboxylate (**11**) in $CDCl_3$

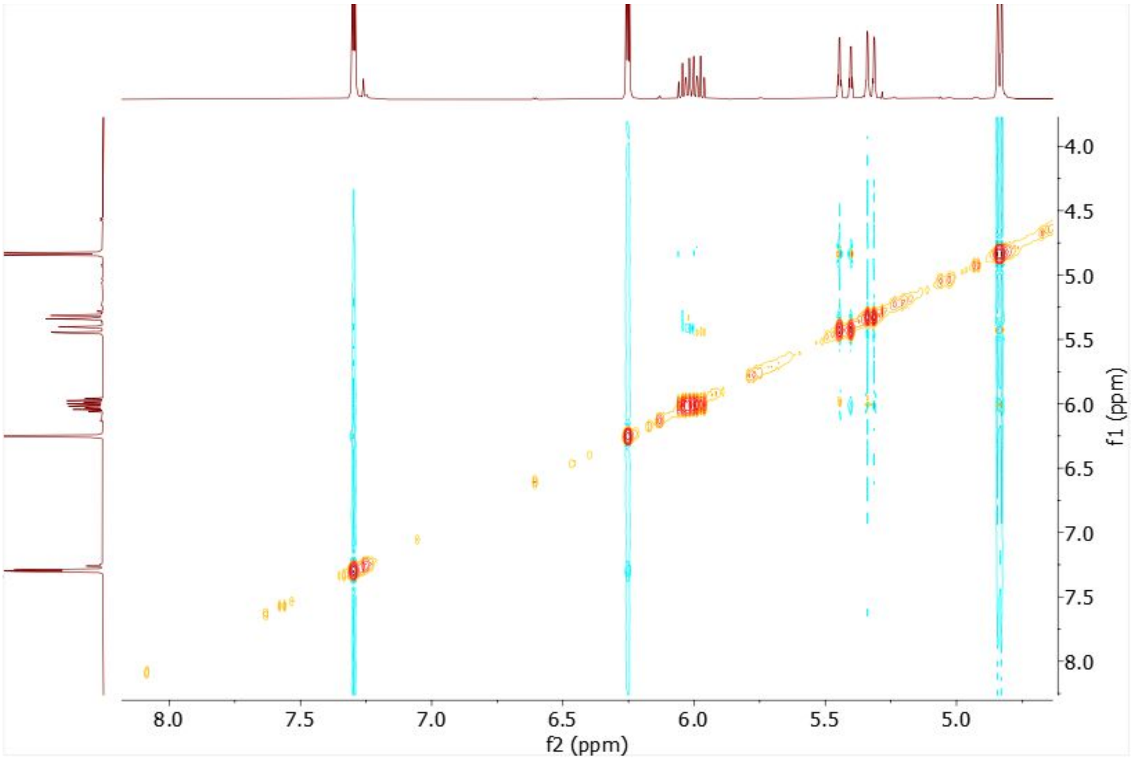

Fig S19: NOESY NMR spectrum of allyl 1*H*-pyrrole-1-carboxylate (**11**) in $CDCl_3$

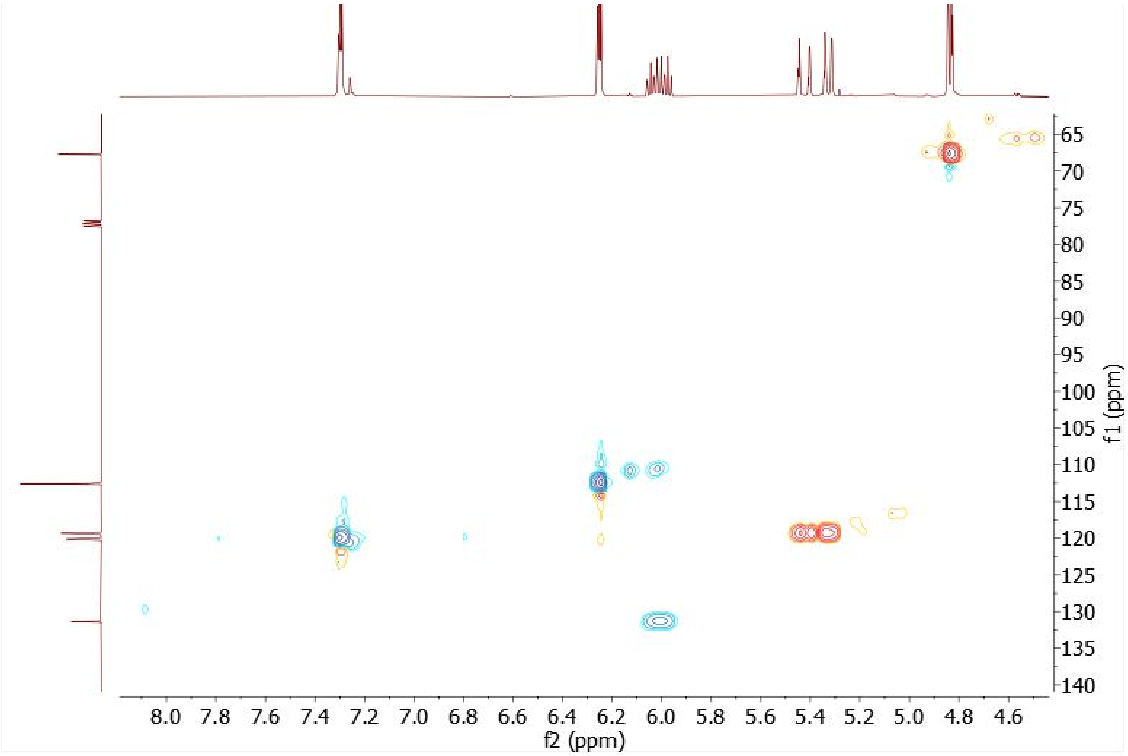

Fig S20: HSQC NMR spectrum of allyl 1*H*-pyrrole-1-carboxylate (**11**) in $CDCl_3$

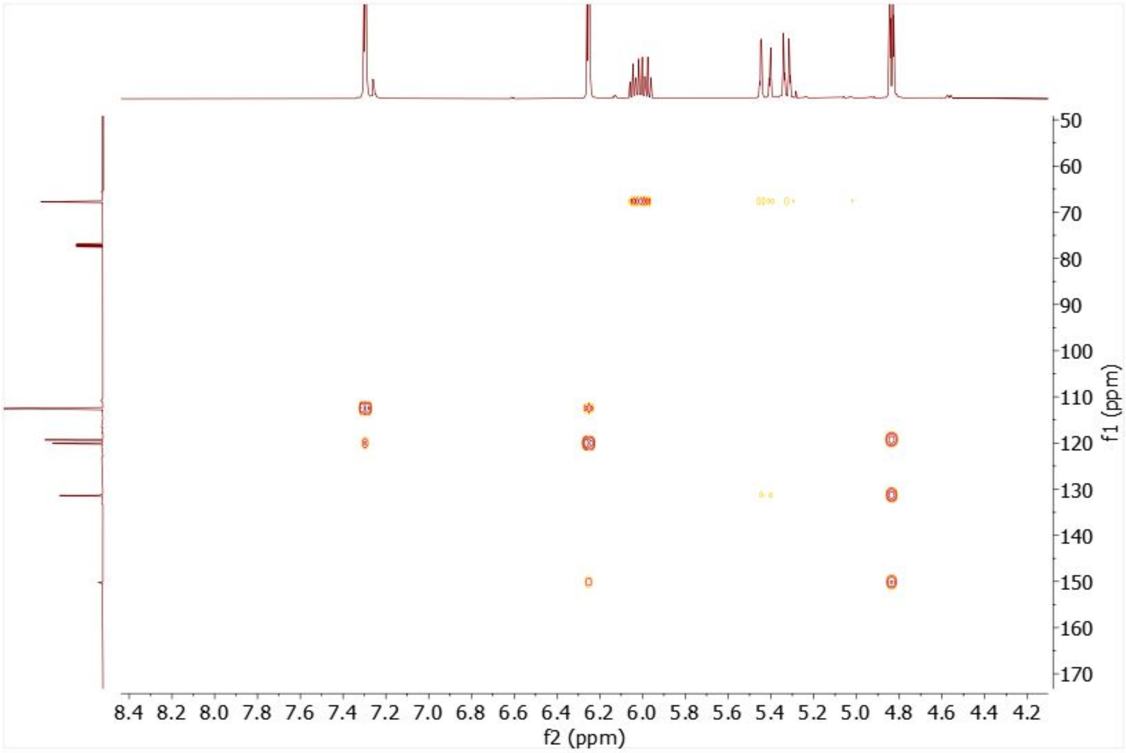

Fig S21: HMBC NMR spectrum of allyl 1*H*-pyrrole-1-carboxylate (**11**) in $CDCl_3$

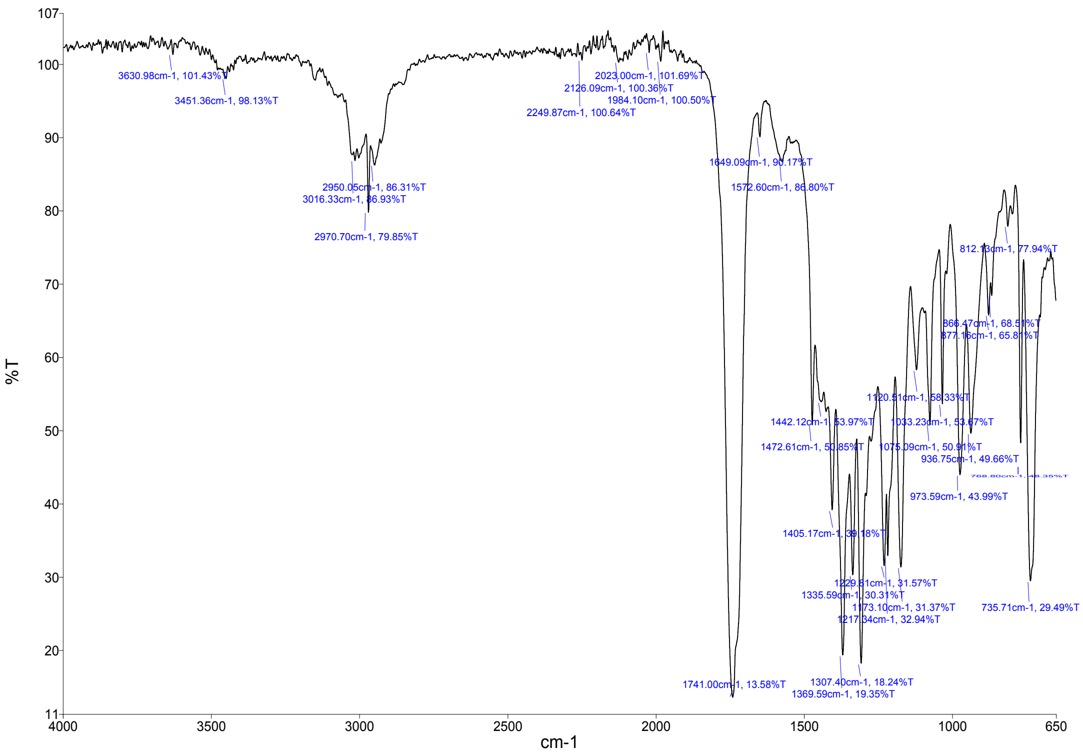

Fig S22: IR spectrum (neat) of allyl 1*H*-pyrrole-1-carboxylate (**11**)

(9*H*-Fluoren-9-yl)methyl 1*H*-pyrrole-1-carboxylate (12)

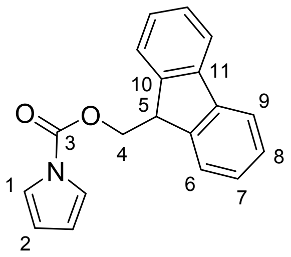

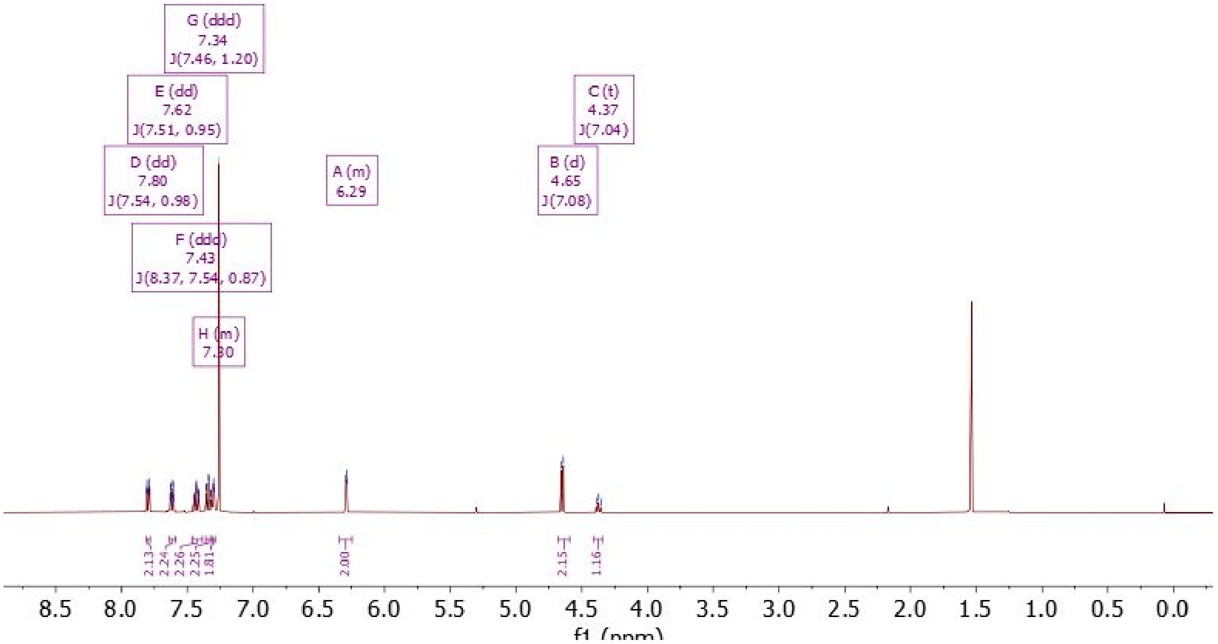

Fig S23: ^{1}H NMR (400 MHz) spectrum of (9*H*-fluoren-9-yl)methyl 1*H*-pyrrole-1-carboxylate (**12**) in $CDCl_3$

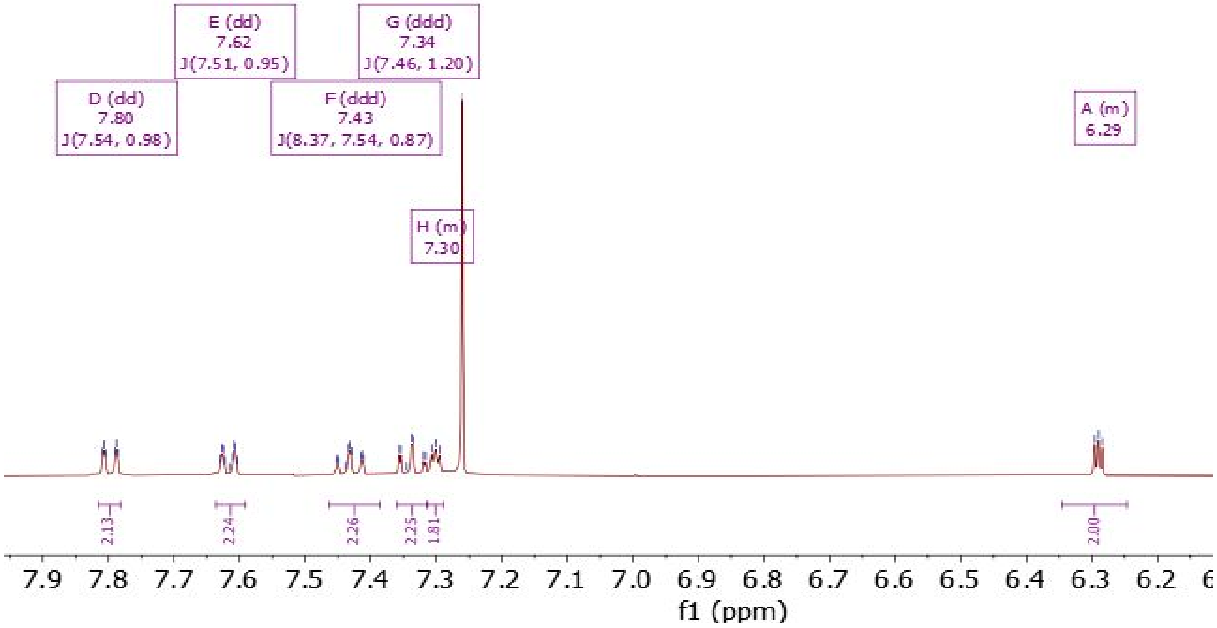

Fig S24: Zoomed region of ^{1}H NMR (400 MHz) spectrum of (9*H*-fluoren-9-yl)methyl 1*H*-pyrrole-1-carboxylate (**12**) in $CDCl_3$

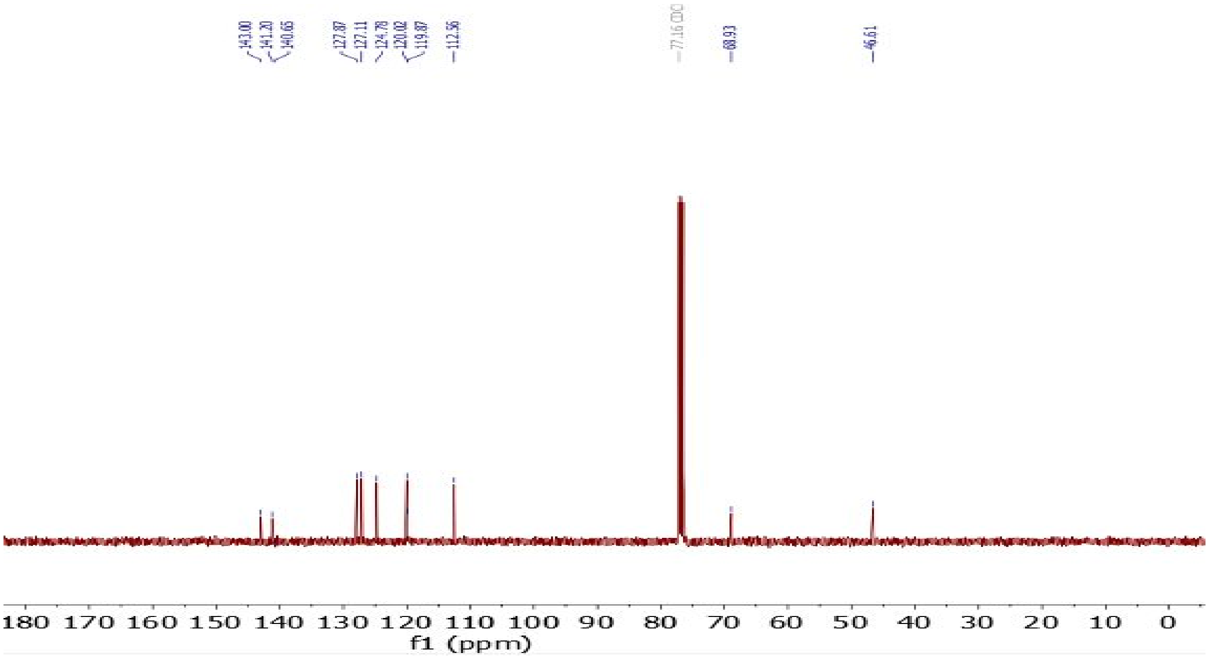

Fig S25: $^{13}C\{^{1}H\}$ NMR (101 MHz) spectrum of (9*H*-fluoren-9-yl)methyl 1*H*-pyrrole-1-carboxylate (**12**) in $CDCl_3$

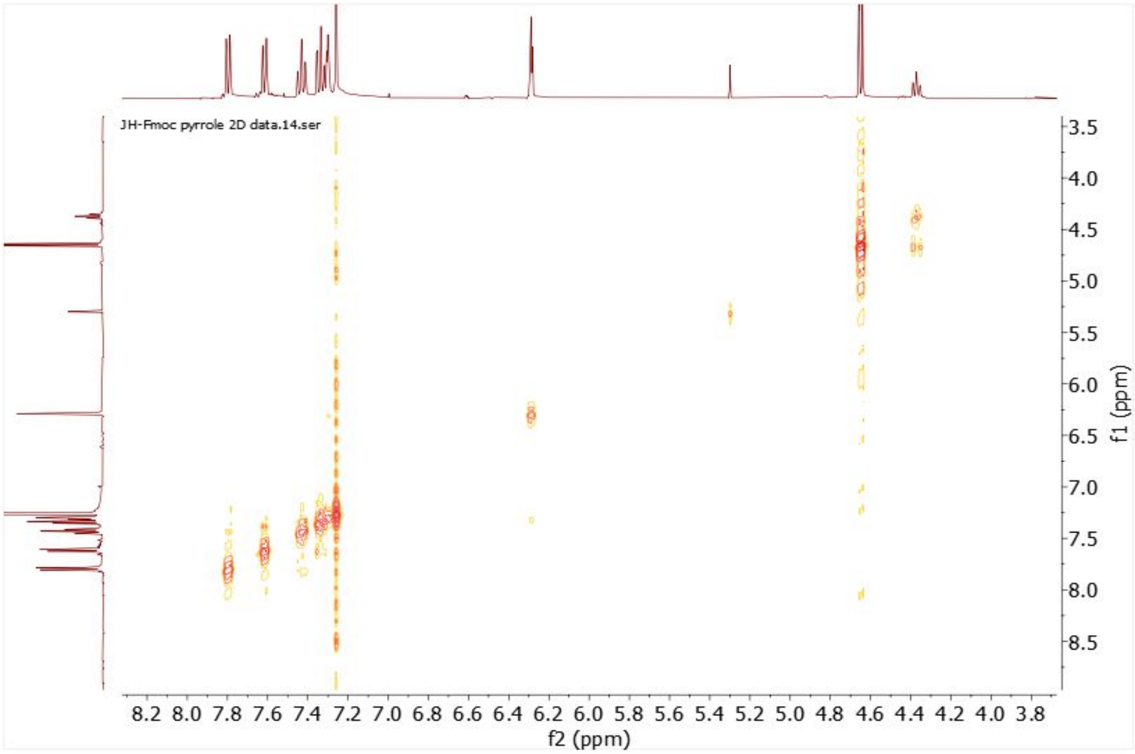

Fig S26: COSY NMR spectrum of (9*H*-fluoren-9-yl)methyl 1*H*-pyrrole-1-carboxylate in $CDCl_3$

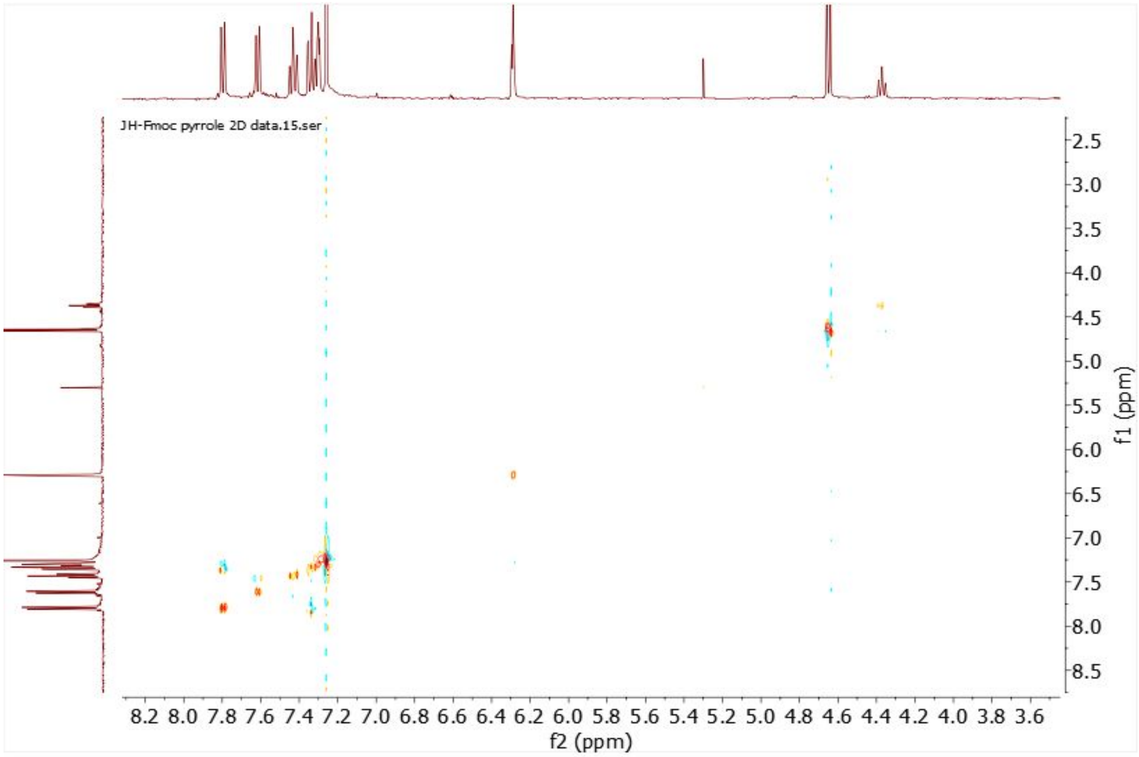

Fig S27: NOESY NMR spectrum of (9*H*-fluoren-9-yl)methyl 1*H*-pyrrole-1-carboxylate (**12**) in $CDCl_3$

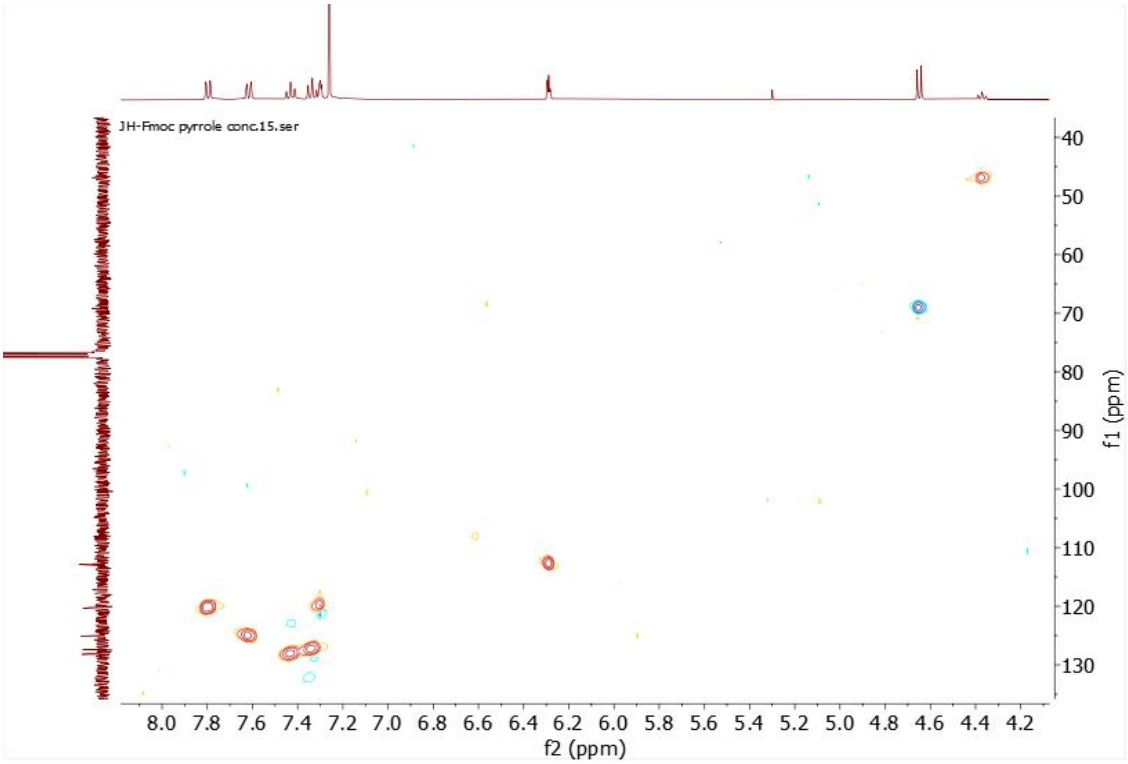

Fig S28: HSQC NMR spectrum of (9*H*-fluoren-9-yl)methyl 1*H*-pyrrole-1-carboxylate (**12**) in $CDCl_3$

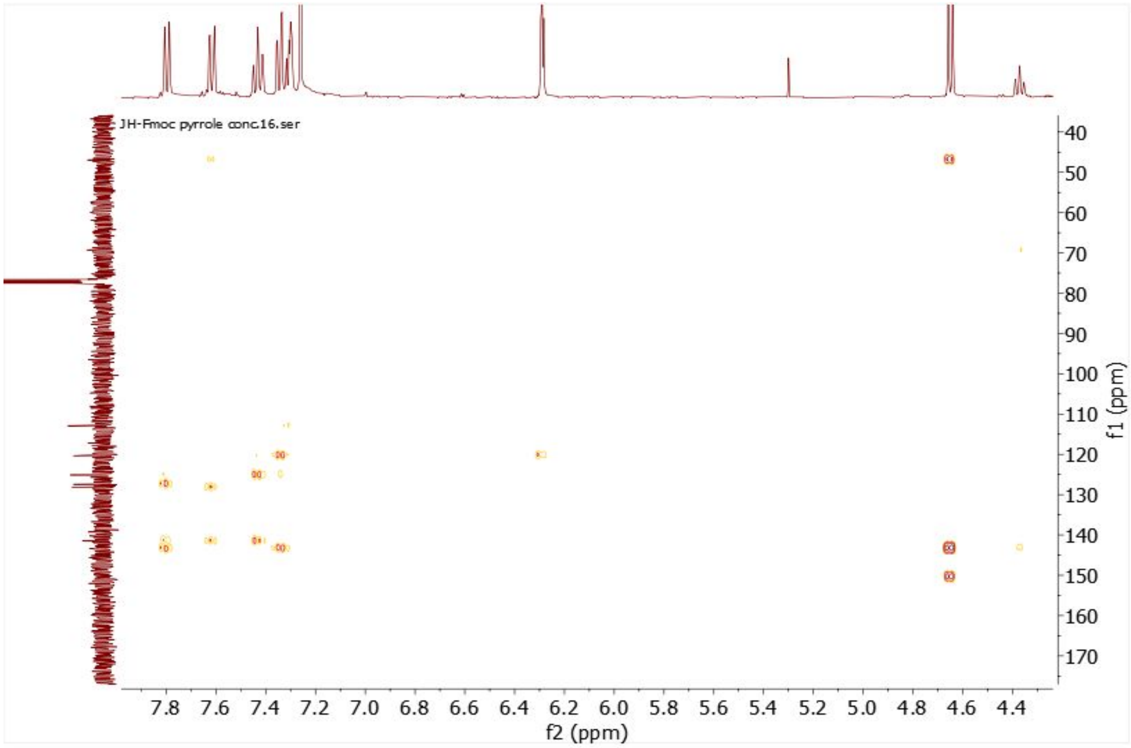

Fig S29: HMBC NMR spectrum of (9*H*-fluoren-9-yl)methyl 1*H*-pyrrole-1-carboxylate (**12**) in $CDCl_3$

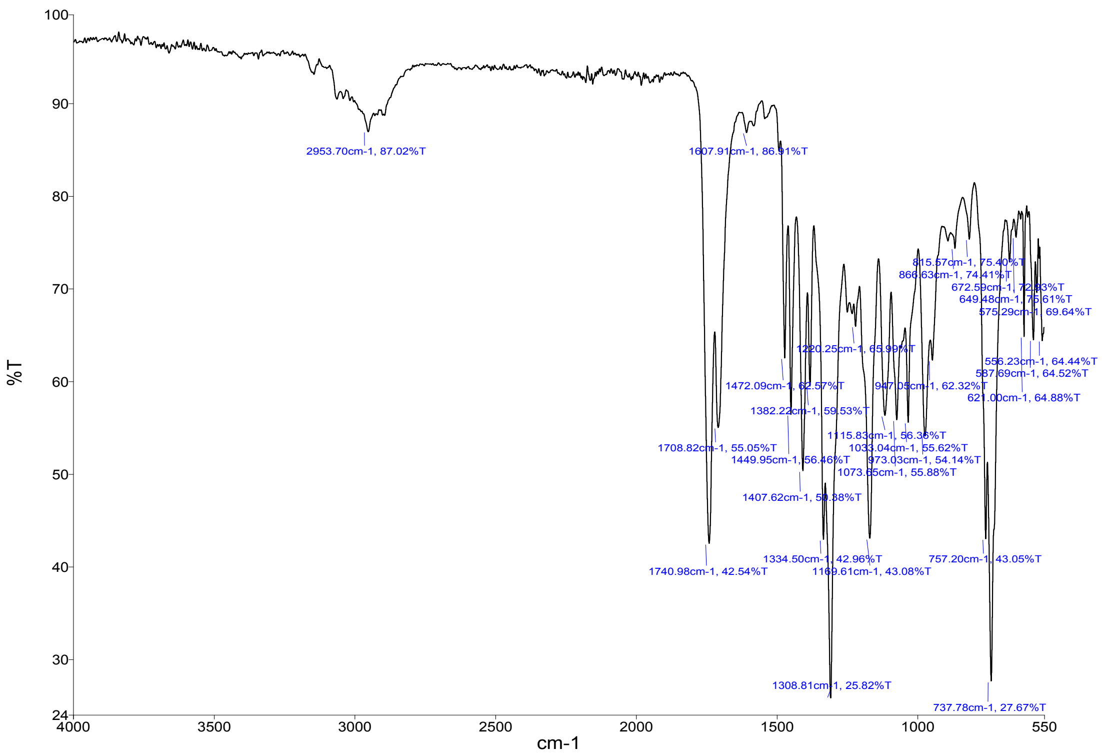

Fig S30: IR (neat) spectrum of (9*H*-fluoren-9-yl)methyl 1*H*-pyrrole-1-carboxylate (**12**)

2,2,2-Trichloroethyl 1*H*-pyrrole-1-carboxylate (13)

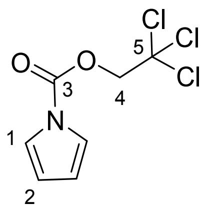

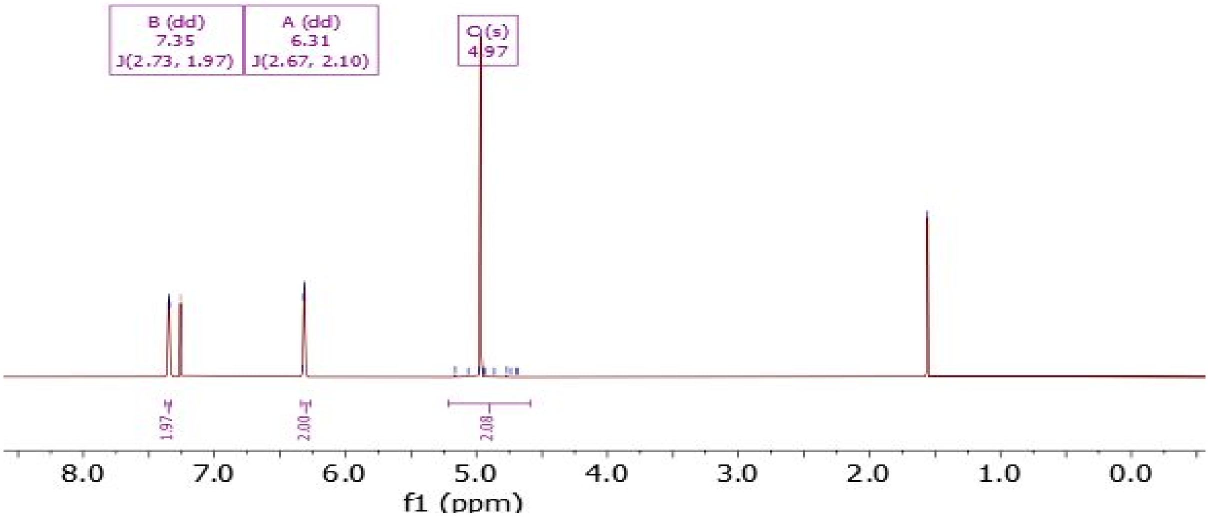

Fig S31: ^{1}H NMR (400 MHz) spectrum of 2,2,2-trichloroethyl 1*H*-pyrrole-1-carboxylate (**13**) in $CDCl_3$

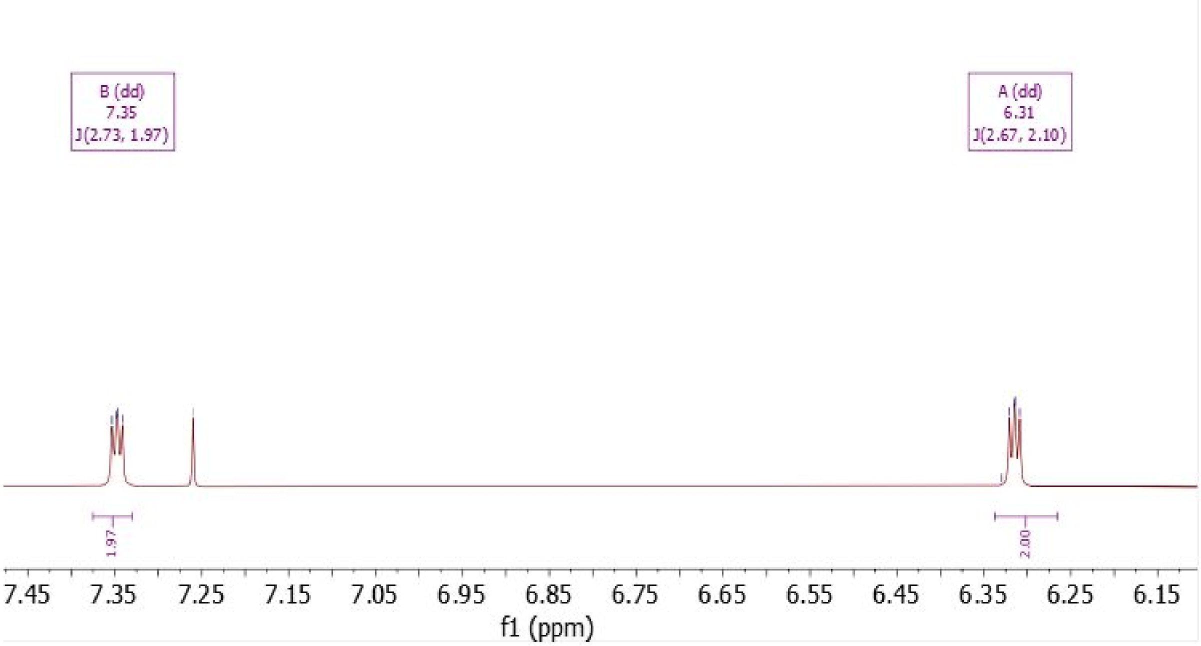

Fig S32: Zoomed region ^{1}H NMR (400 MHz) spectrum of 2,2,2-trichloroethyl 1*H*-pyrrole-1-carboxylate (**13**) in $CDCl_3$

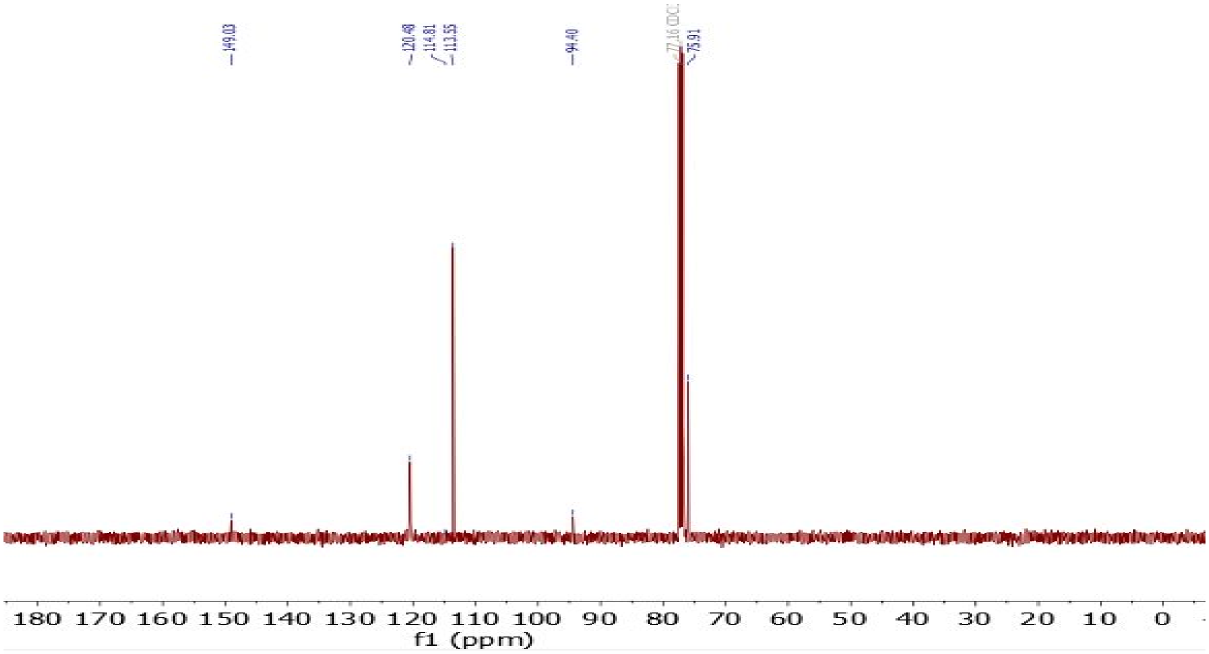

Fig S33: $^{13}C\{^{1}H\}$ NMR (101 MHz) spectrum of 2,2,2-trichloroethyl 1*H*-pyrrole-1-carboxylate (**13**) in $CDCl_3$

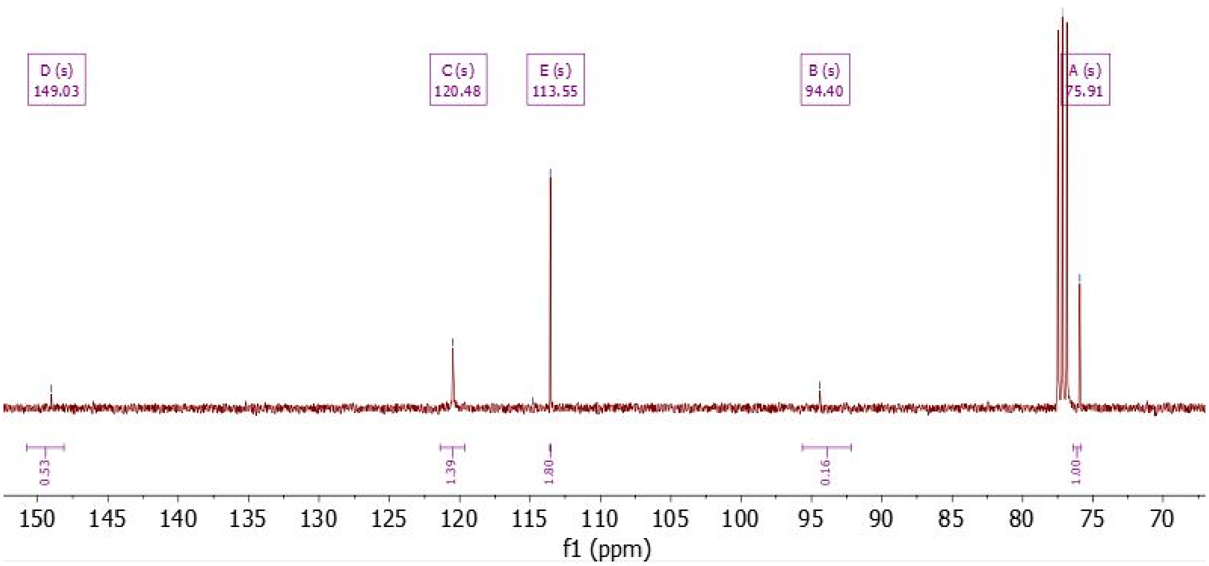

Fig S34: Zoomed region of $^{13}C\{^{1}H\}$ NMR (101 MHz) spectrum of 2,2,2-trichloroethyl 1*H*-pyrrole-1-carboxylate (**13**) in $CDCl_3$

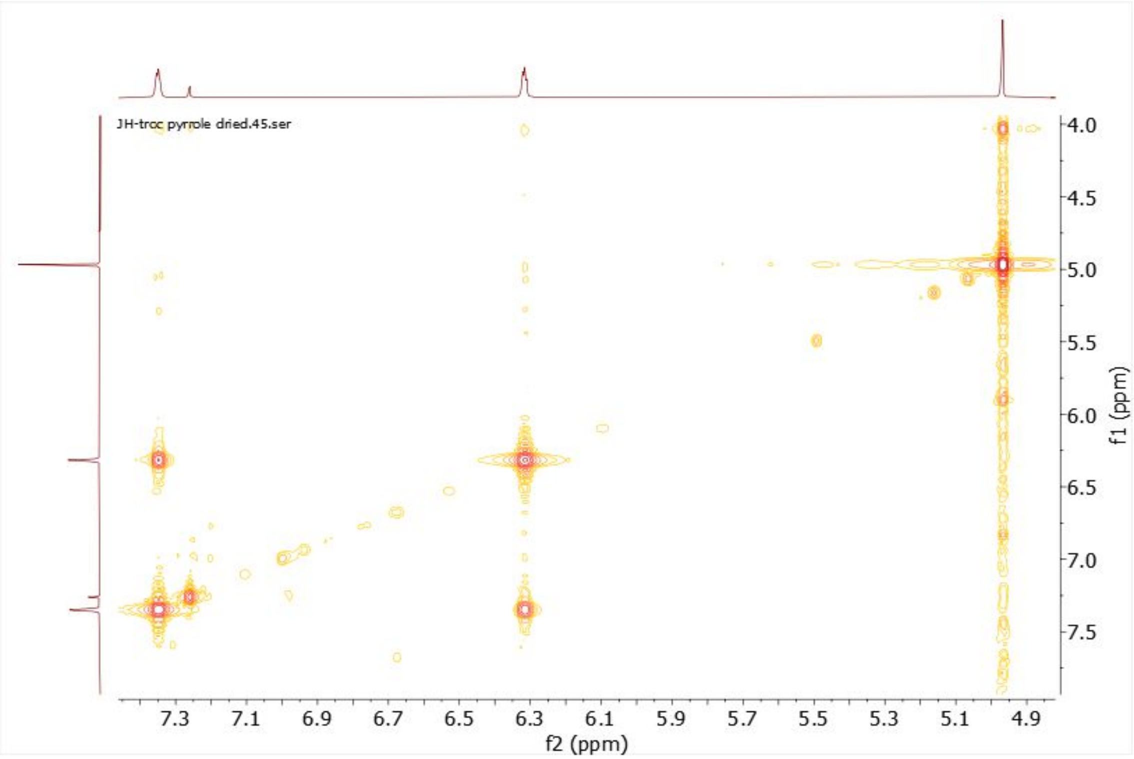

Fig S35: COSY NMR spectrum of 2,2,2-trichloroethyl 1*H*-pyrrole-1-carboxylate (**13**) in $CDCl_3$

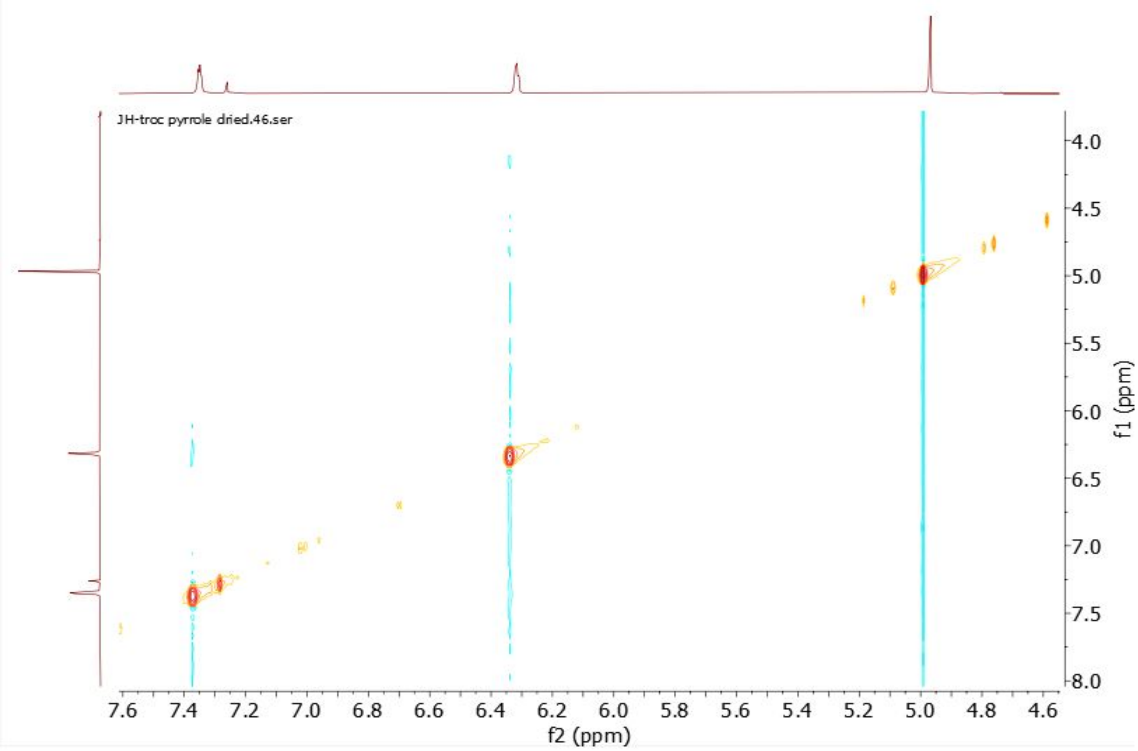

Fig S36: NOESY NMR spectrum of 2,2,2-trichloroethyl 1*H*-pyrrole-1-carboxylate (**13**) in $CDCl_3$

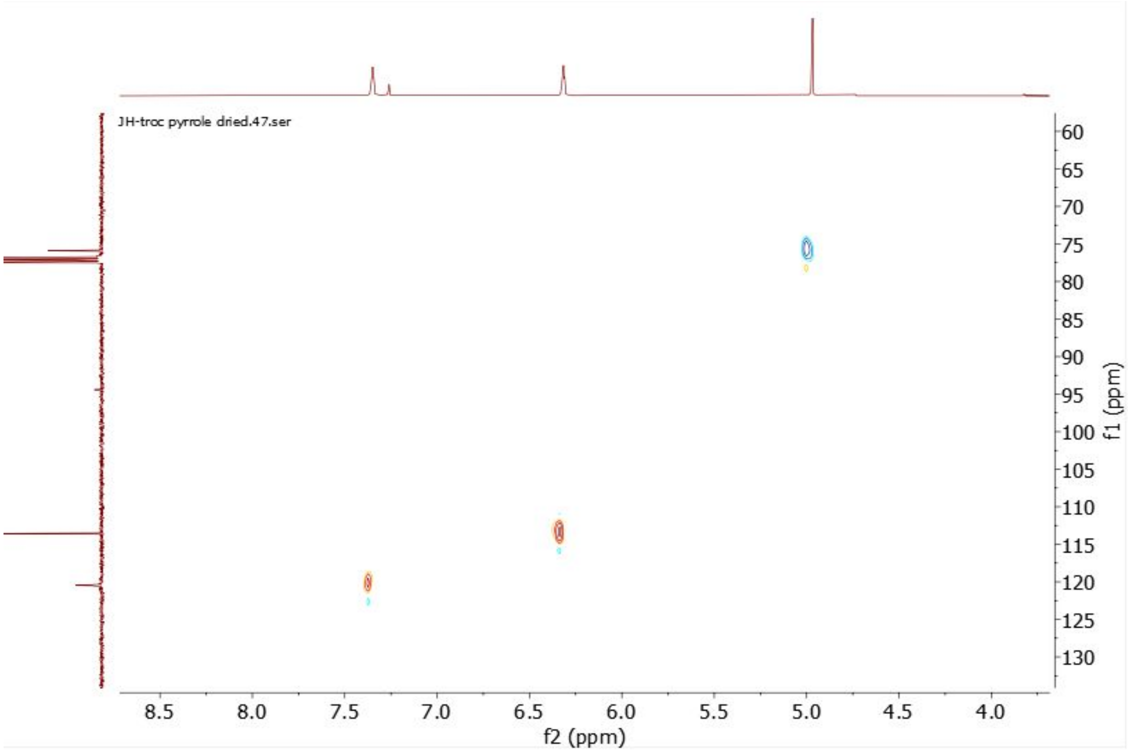

Fig S37: HSQC NMR spectrum of 2,2,2-trichloroethyl 1*H*-pyrrole-1-carboxylate (**13**) in $CDCl_3$

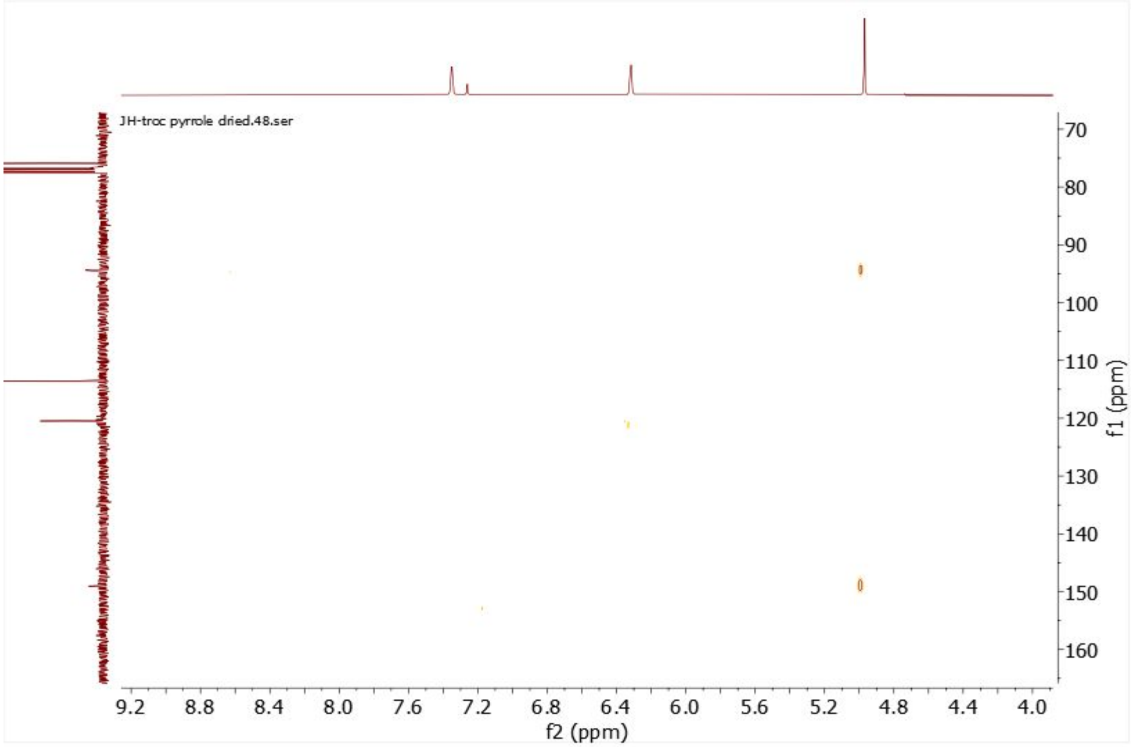

Fig S38: HMBC NMR spectrum of 2,2,2-trichloroethyl 1*H*-pyrrole-1-carboxylate (**13**) in $CDCl_3$

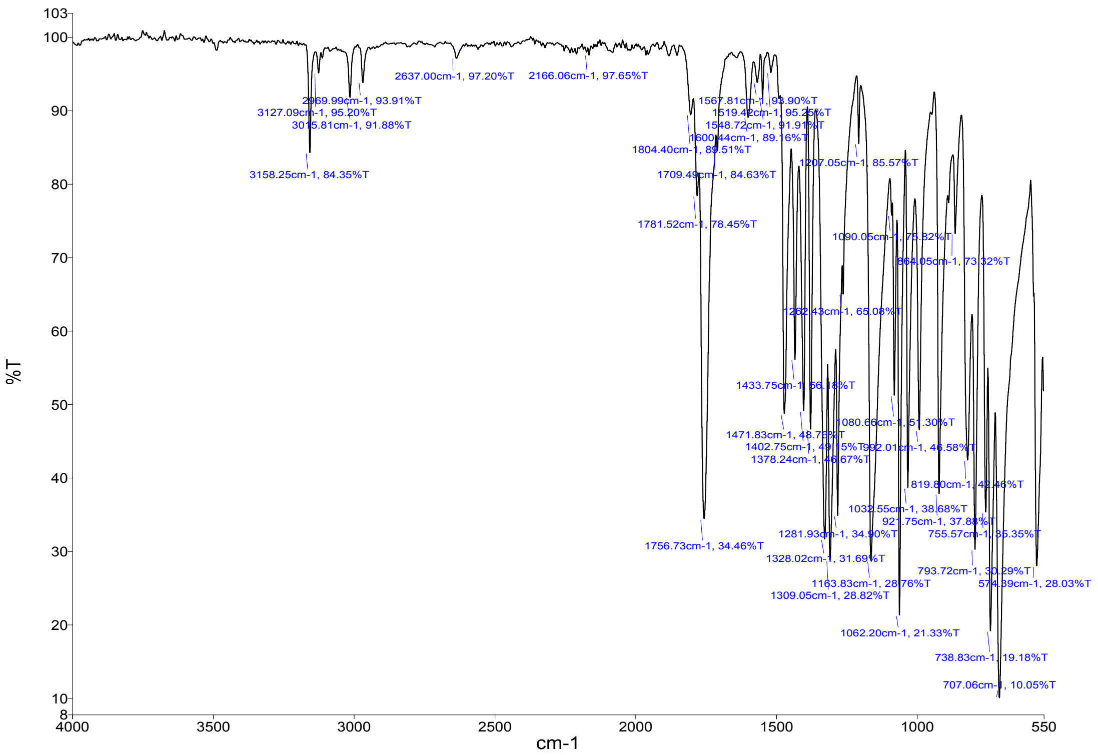

Fig S39: IR (neat) spectrum of 2,2,2-trichloroethyl 1*H*-pyrrole-1-carboxylate (**13**)

Methyl 2-acetyl-1*H*-pyrrole-1-carboxylate (14)

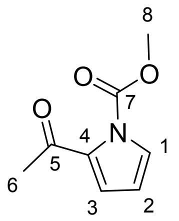

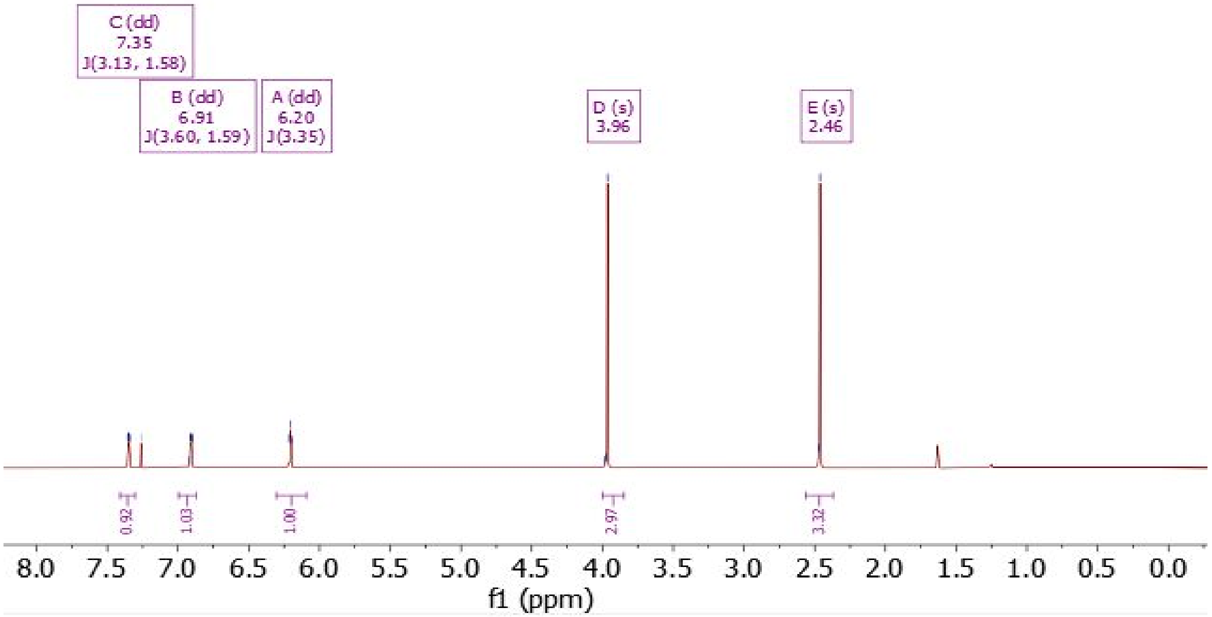

Fig S40: ^{1}H NMR (400 MHz) spectrum of methyl 2-acetyl-1*H*-pyrrole-1-carboxylate (**14**) in $CDCl_3$

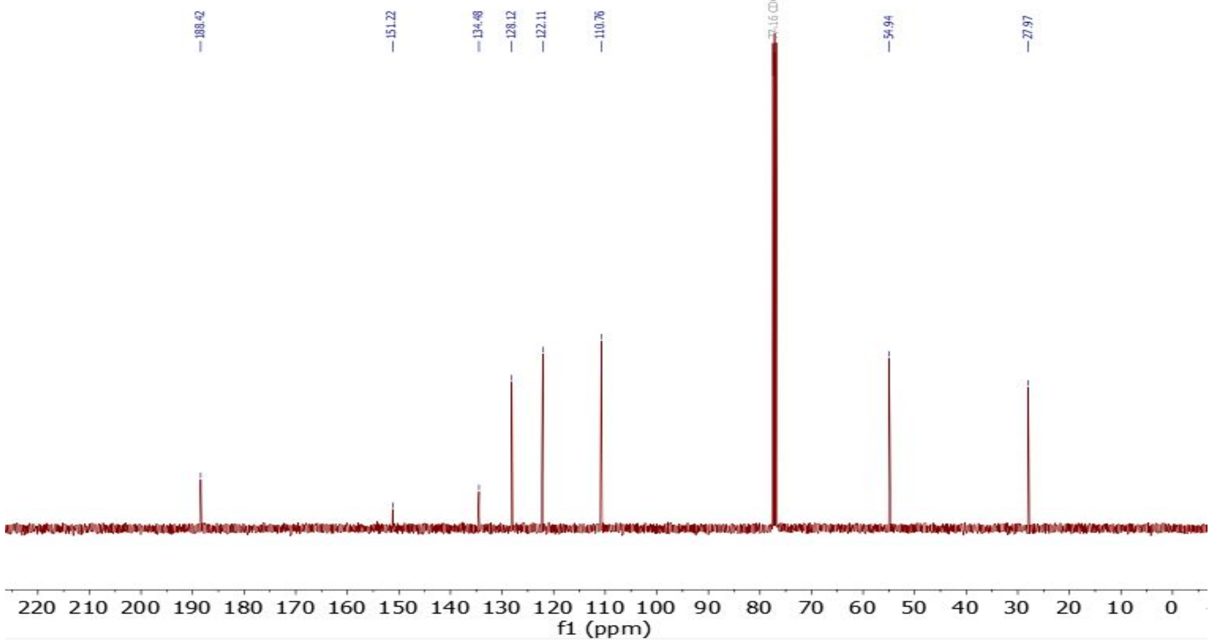

Fig S41: $^{13}C\{^{1}H\}$ NMR (101 MHz) spectrum of methyl 2-acetyl-1*H*-pyrrole-1-carboxylate (**14**) in $CDCl_3$

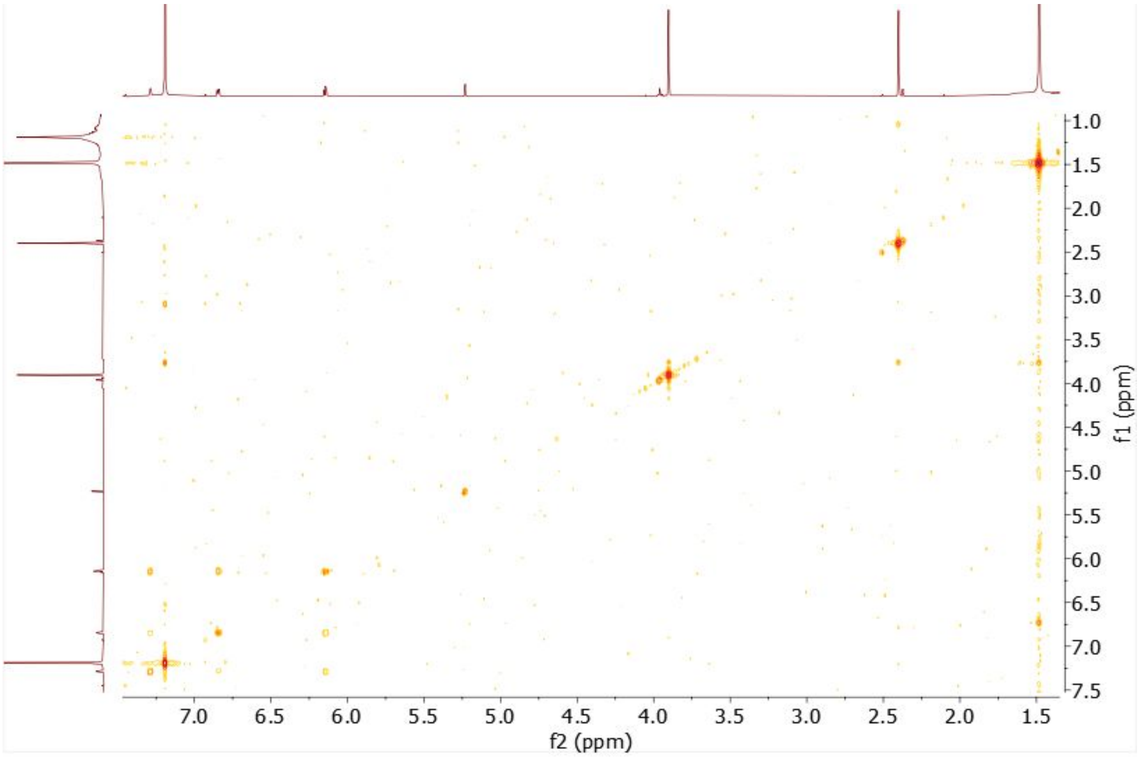

Fig S42: COSY NMR spectrum of methyl 2-acetyl-1*H*-pyrrole-1-carboxylate (**14**) in $CDCl_3$

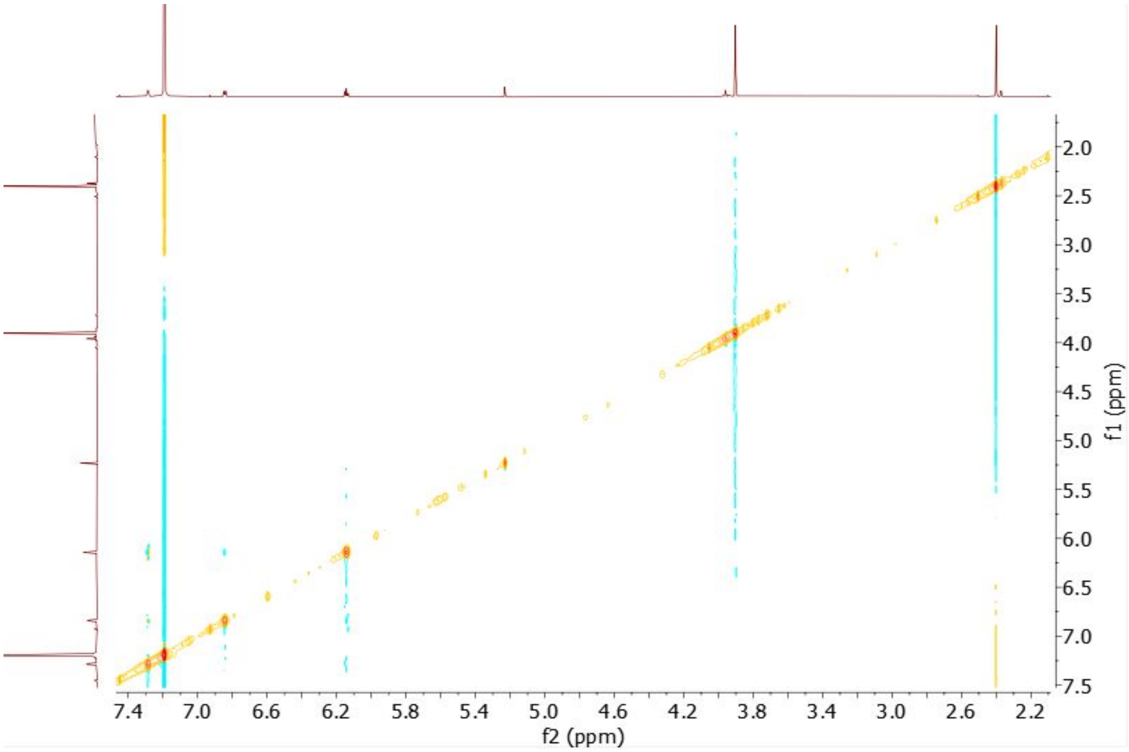

Fig S43: NOESY NMR spectrum of methyl 2-acetyl-1*H*-pyrrole-1-carboxylate (**14**) in $CDCl_3$

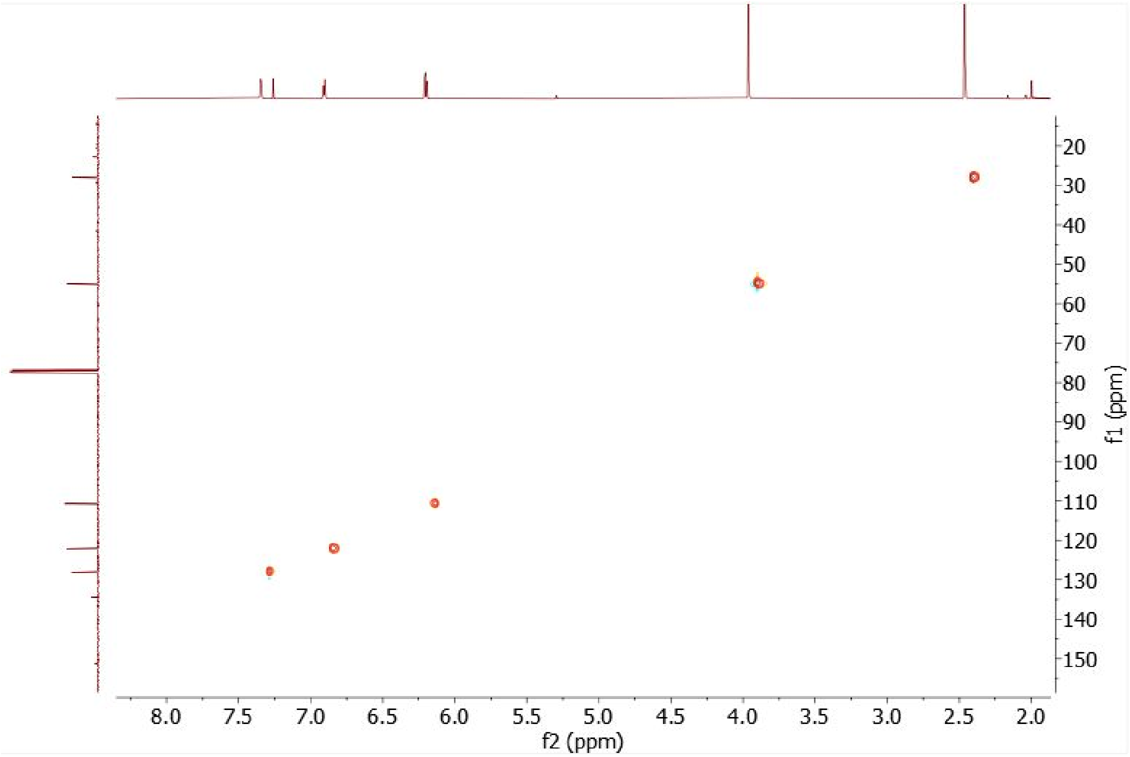

Fig S44: HSQC NMR spectrum of methyl 2-acetyl-1*H*-pyrrole-1-carboxylate (**14**) in $CDCl_3$

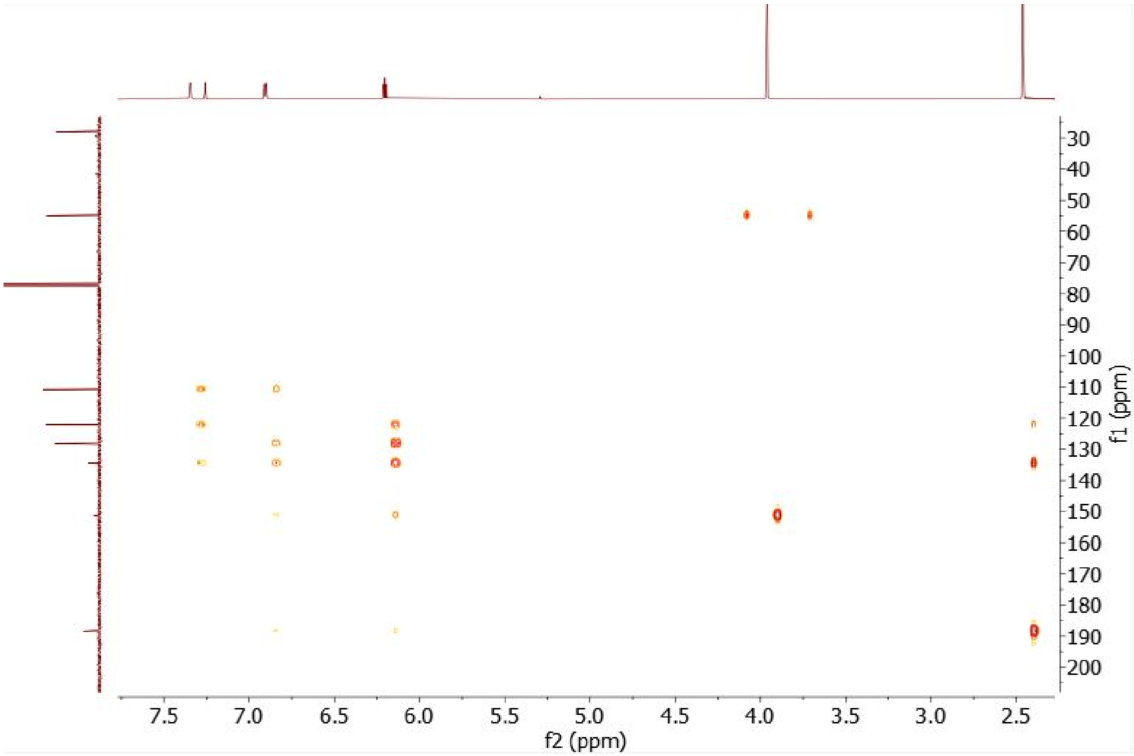

Fig S45: HMBC NMR spectrum of methyl 2-acetyl-1*H*-pyrrole-1-carboxylate (**14**) in $CDCl_3$

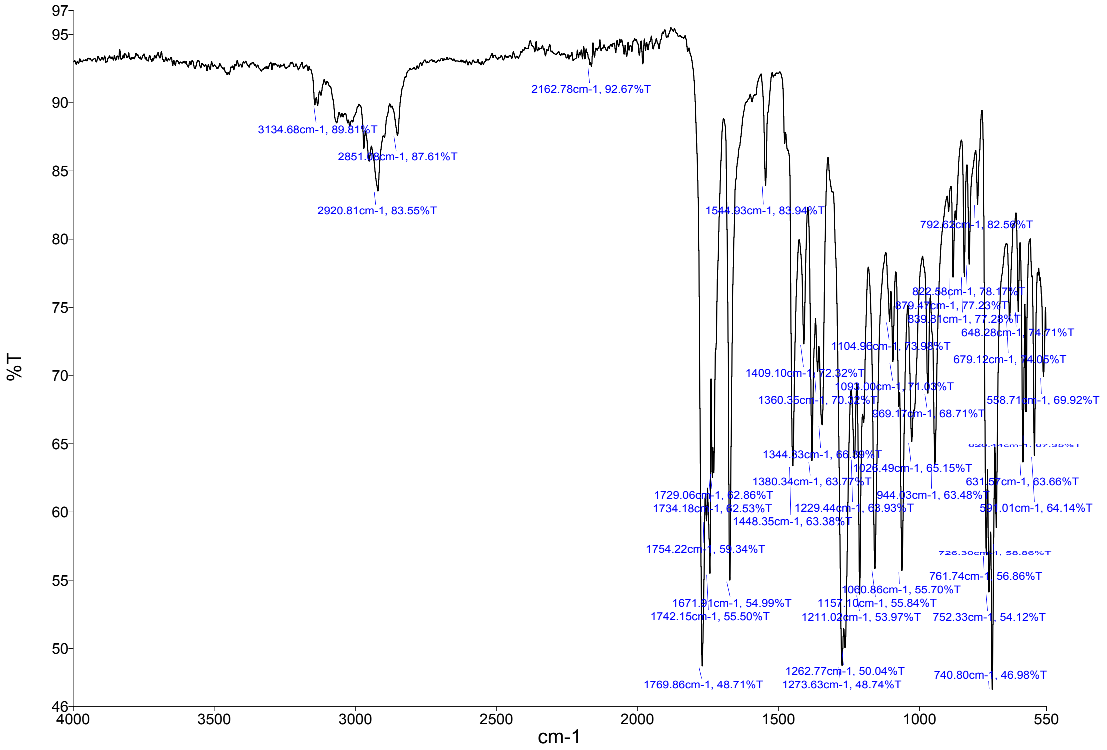

Fig S46: IR spectrum of methyl 2-acetyl-1*H*-pyrrole-1-carboxylate (**14**)

Benzyl 2-acetyl-1*H*-pyrrole-1-carboxylate (15)

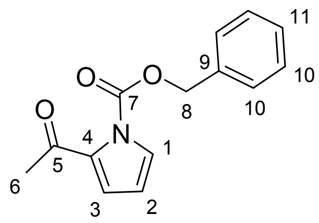

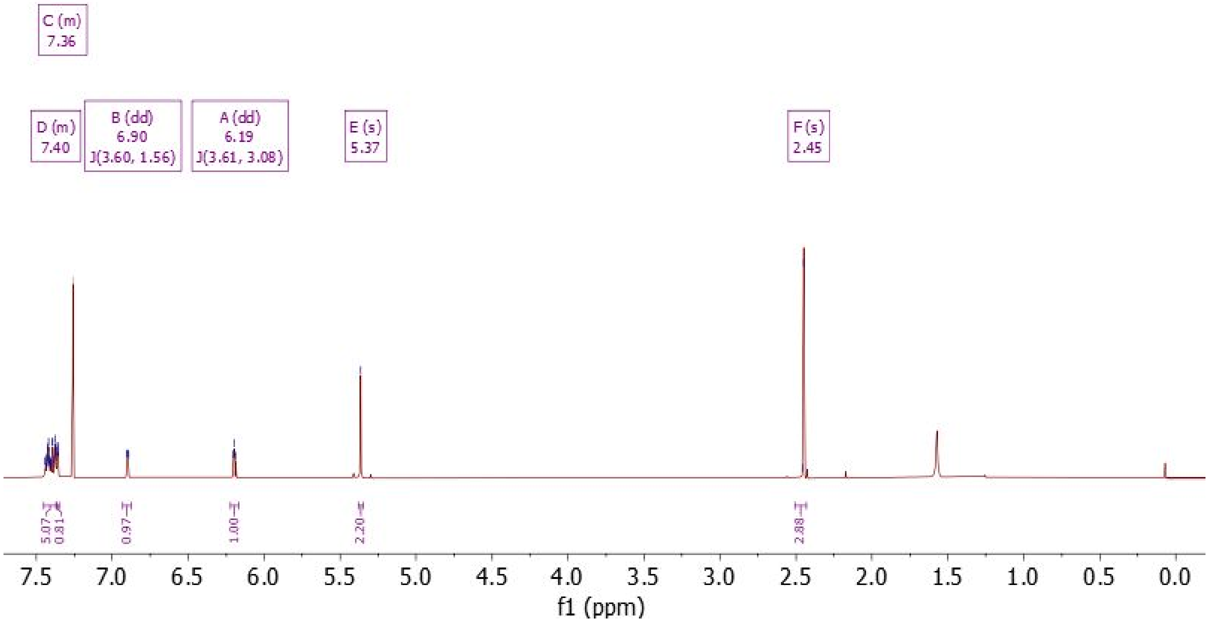

Fig S47: ^{1}H NMR (400 MHz) spectrum of benzyl 2-acetyl-1*H*-pyrrole-1-carboxylate (**15**) in $CDCl_3$

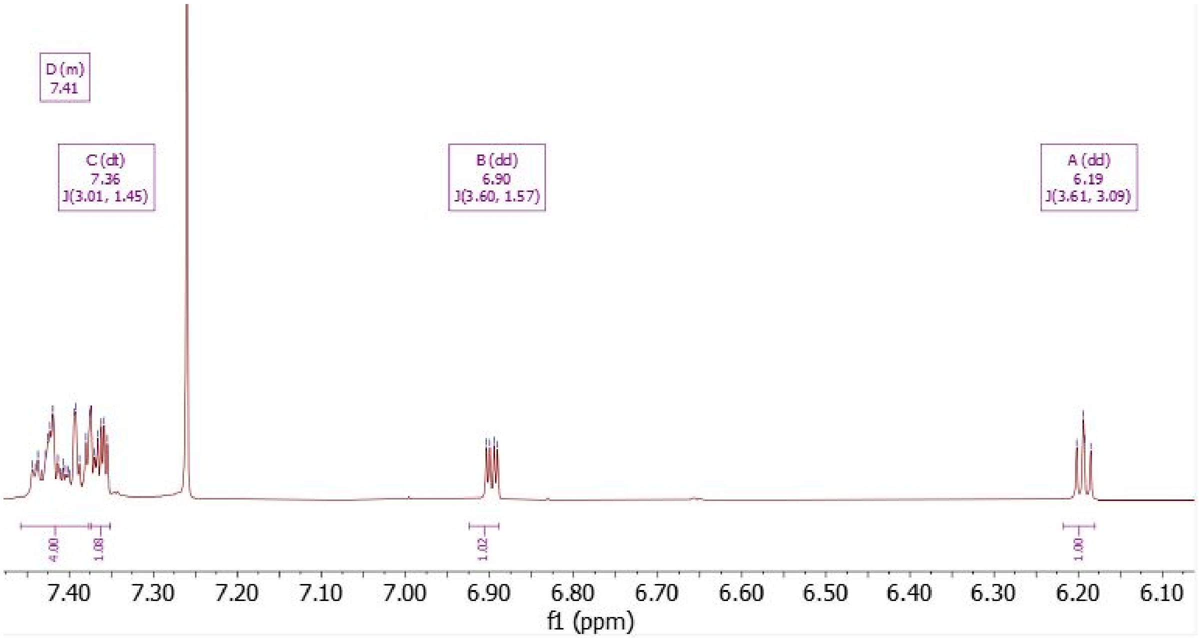

Fig S48: Zoomed region ^{1}H NMR (101 MHz) spectrum of benzyl 2-acetyl-1*H*-pyrrole-1-carboxylate (**15**) in $CDCl_3$

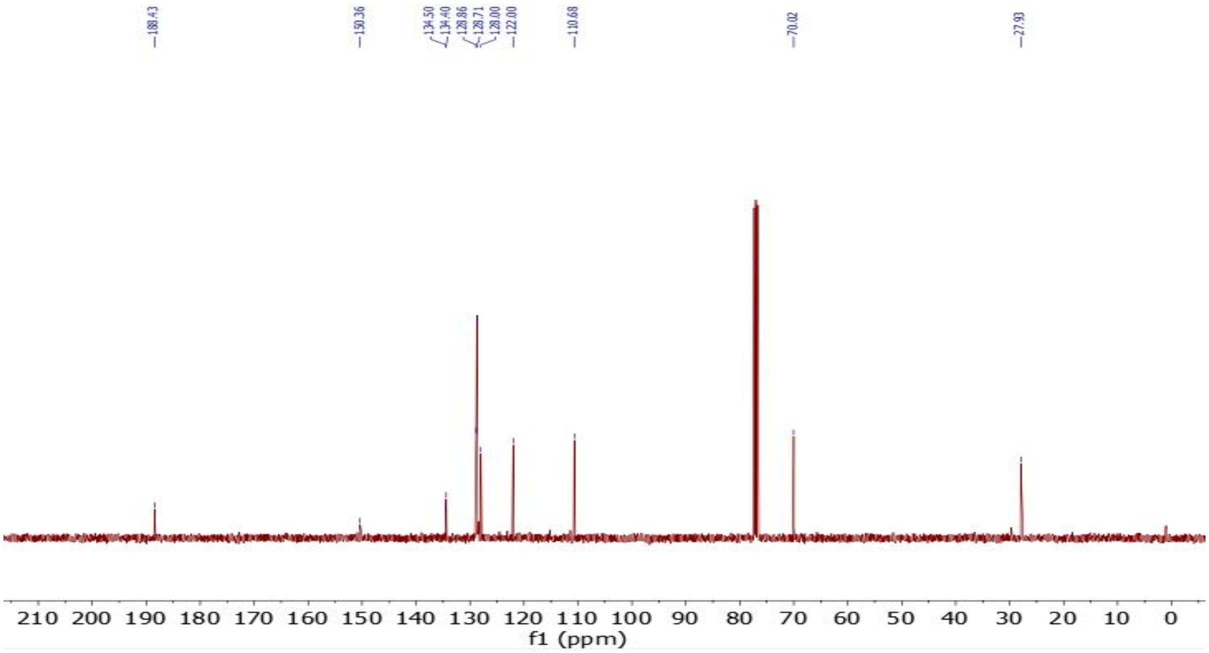

Fig S49: $^{13}C\{^{1}H\}$ NMR (101 MHz) spectrum of benzyl 2-acetyl-1*H*-pyrrole-1-carboxylate (**15**) in $CDCl_3$

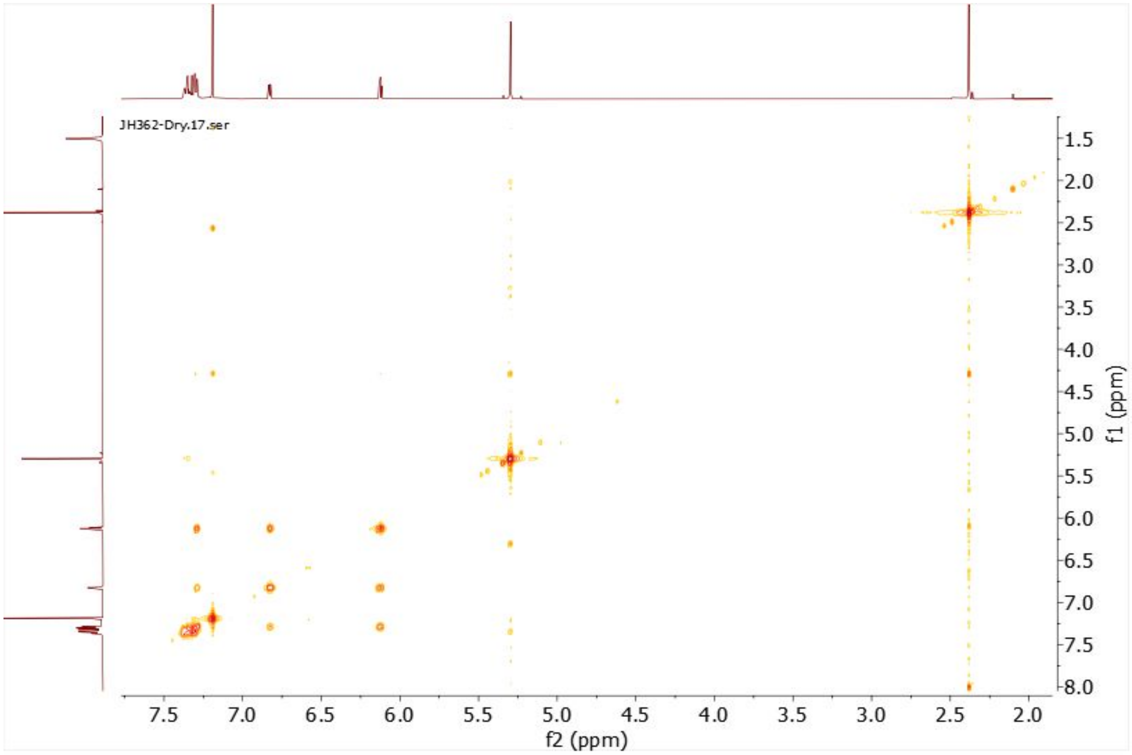

Fig S50: COSY NMR spectrum of benzyl 2-acetyl-1*H*-pyrrole-1-carboxylate (**15**) in $CDCl_3$

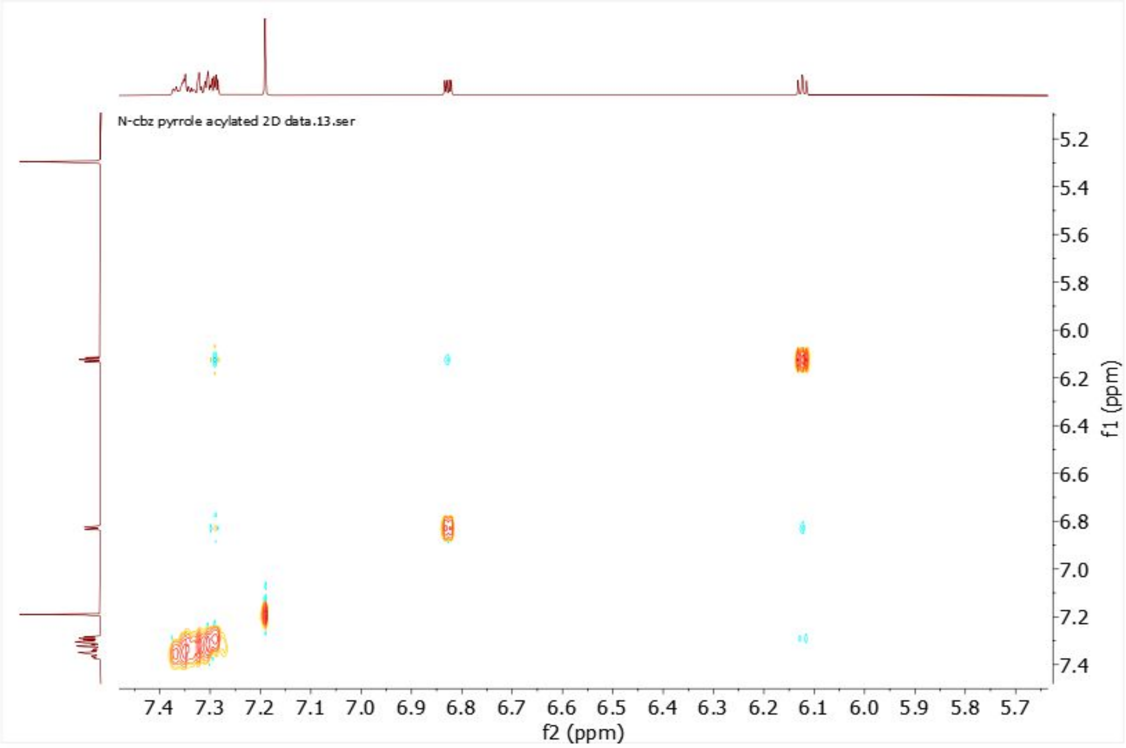

Fig S51: NOESY NMR spectrum of benzyl 2-acetyl-1*H*-pyrrole-1-carboxylate (**15**) in $CDCl_3$

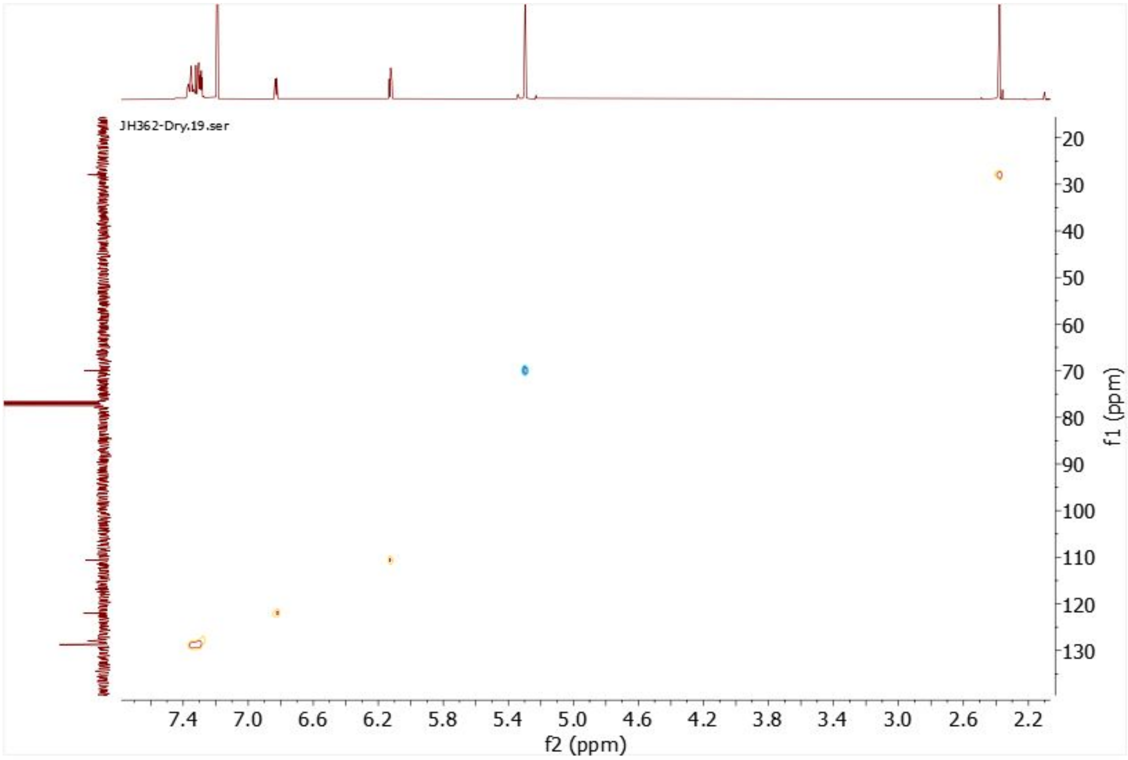

Fig S52: HSQC NMR spectrum of benzyl 2-acetyl-1*H*-pyrrole-1-carboxylate (**15**) in $CDCl_3$

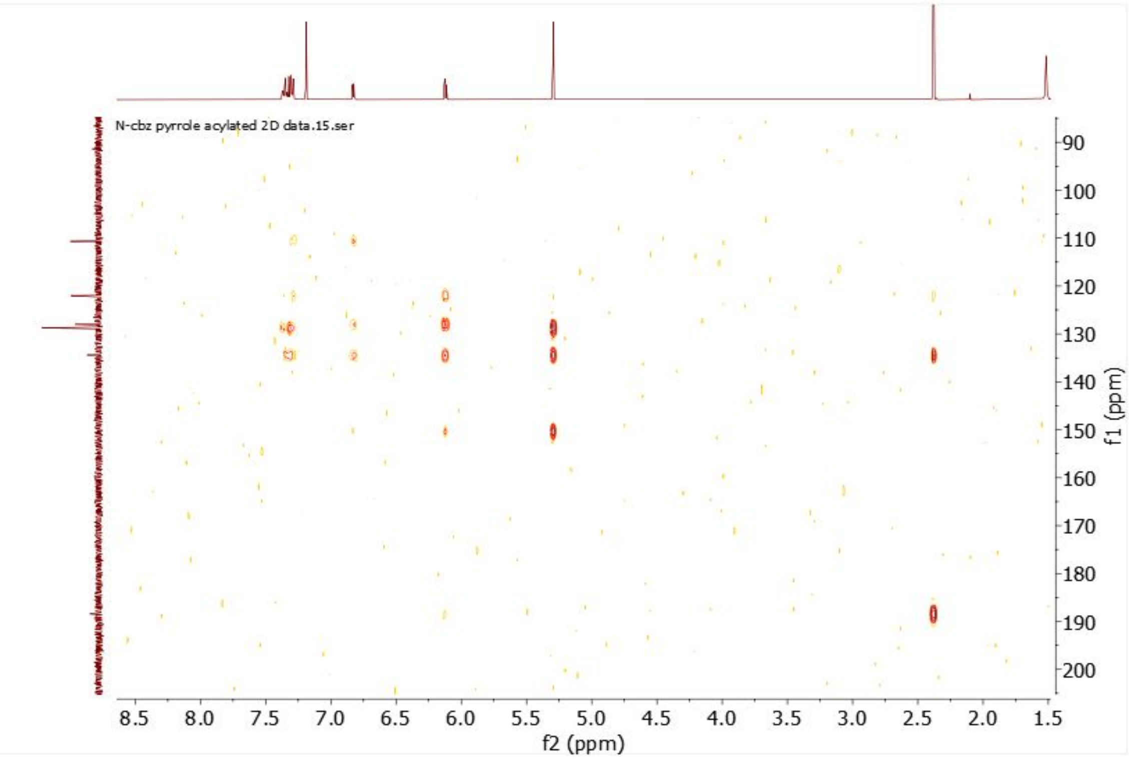

Fig S53: HMBC NMR spectrum of benzyl 2-acetyl-1*H*-pyrrole-1-carboxylate (**15**) in $CDCl_3$

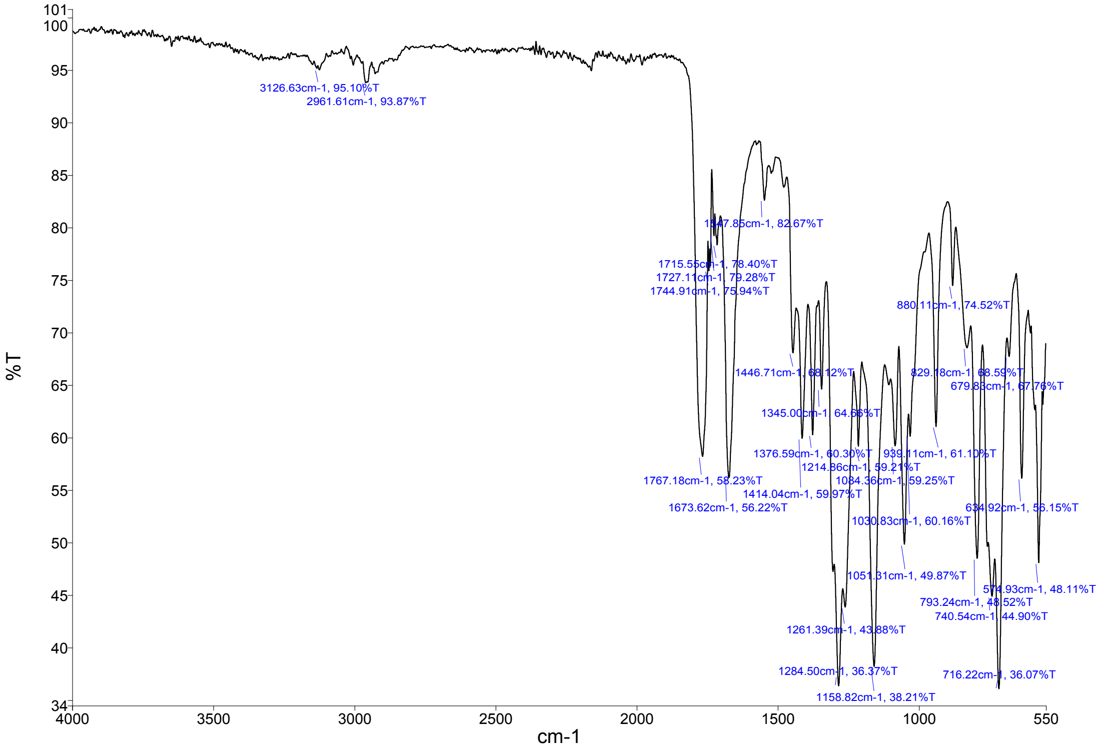

Fig S54: IR (neat) spectrum of benzyl 2-acetyl-1*H*-pyrrole-1-carboxylate (**15**)

(9*H*-Fluoren-9-yl)methyl 2-acetyl-1*H*-pyrrole-1-carboxylate (16)

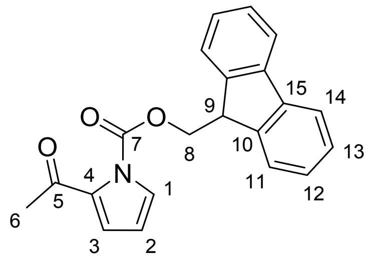

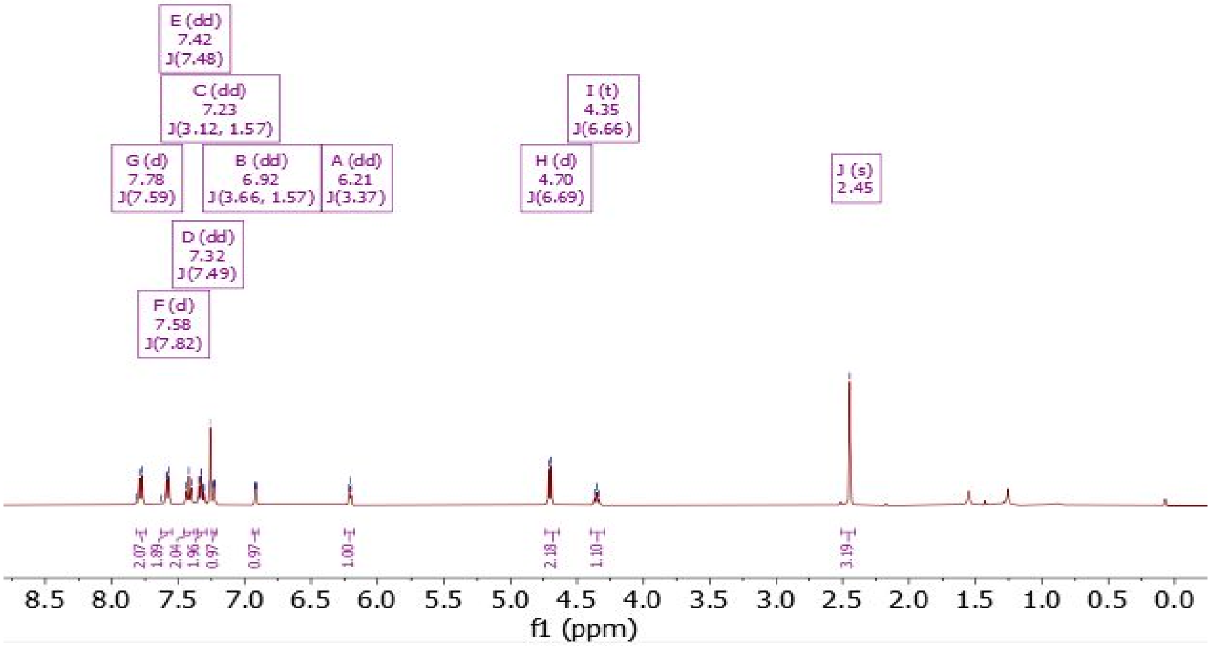

Fig S55: ^{1}H NMR (400 MHz) spectrum of (9*H*-fluoren-9-yl)methyl 2-acetyl-1*H*-pyrrole-1-carboxylate (**16**) in $CDCl_3$

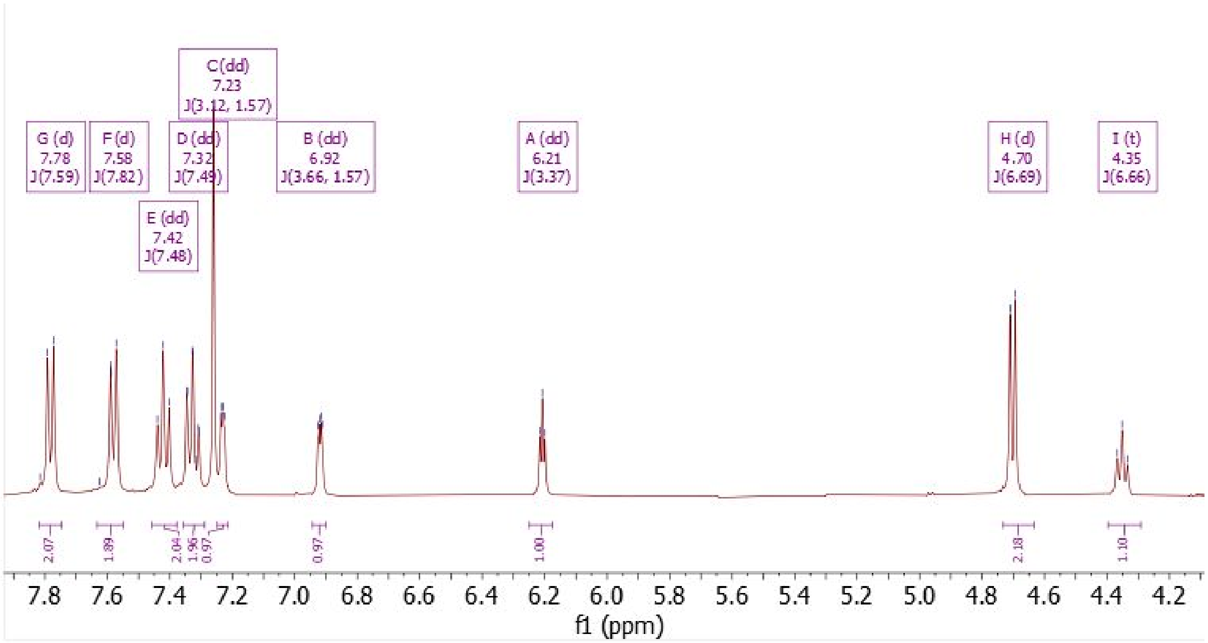

Fig S56: Zoomed region ^{1}H NMR (400 MHz) spectrum of (9*H*-fluoren-9-yl)methyl 2-acetyl-1*H*-pyrrole-1-carboxylate (**16**) in $CDCl_3$

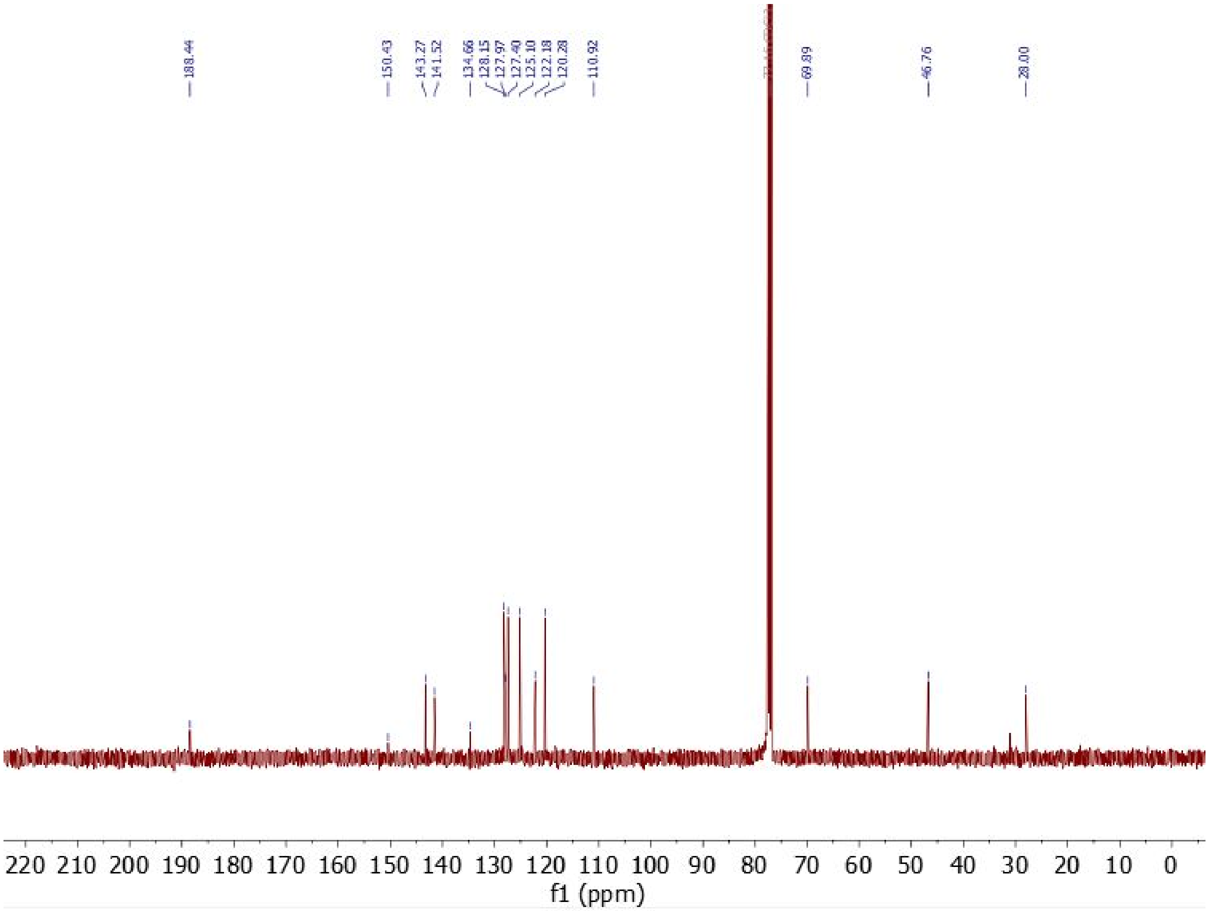

Fig S57: $^{13}C\{^{1}H\}$ NMR (101 MHz) spectrum of (9*H*-fluoren-9-yl)methyl 2-acetyl-1*H*-pyrrole-1-carboxylate (**16**) in $CDCl_3$

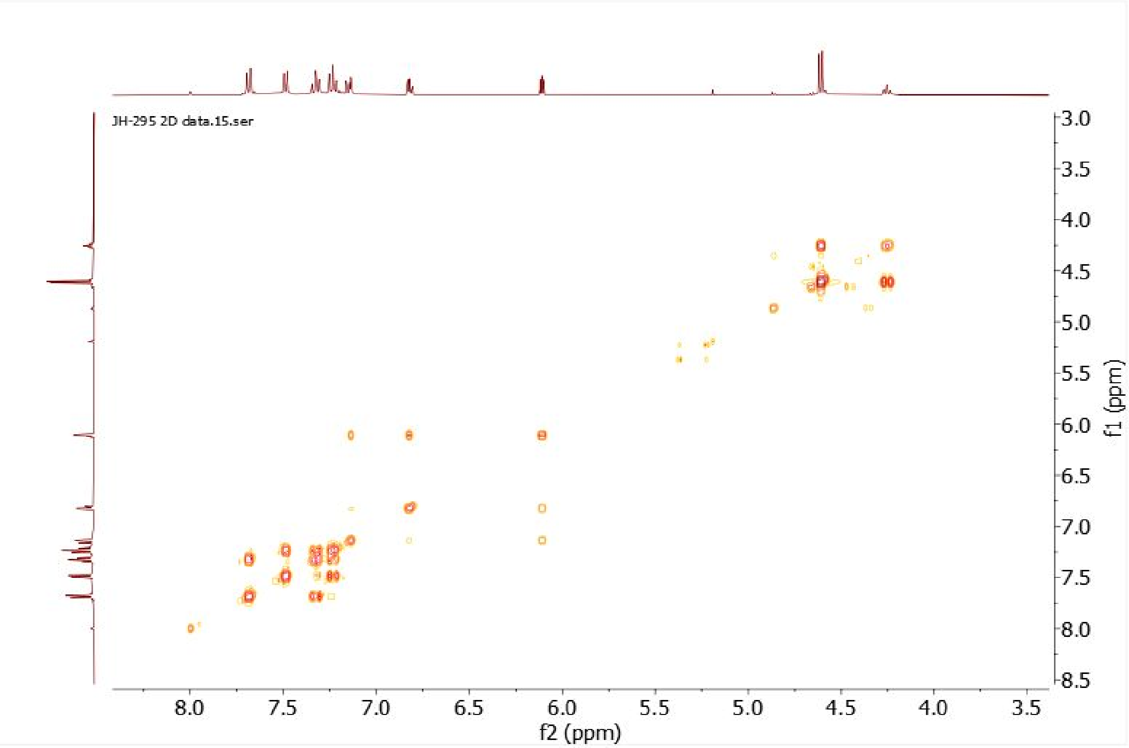

Fig S58: COSY NMR spectrum of (9*H*-fluoren-9-yl)methyl 2-acetyl-1*H*-pyrrole-1-carboxylate (**16**) in $CDCl_3$

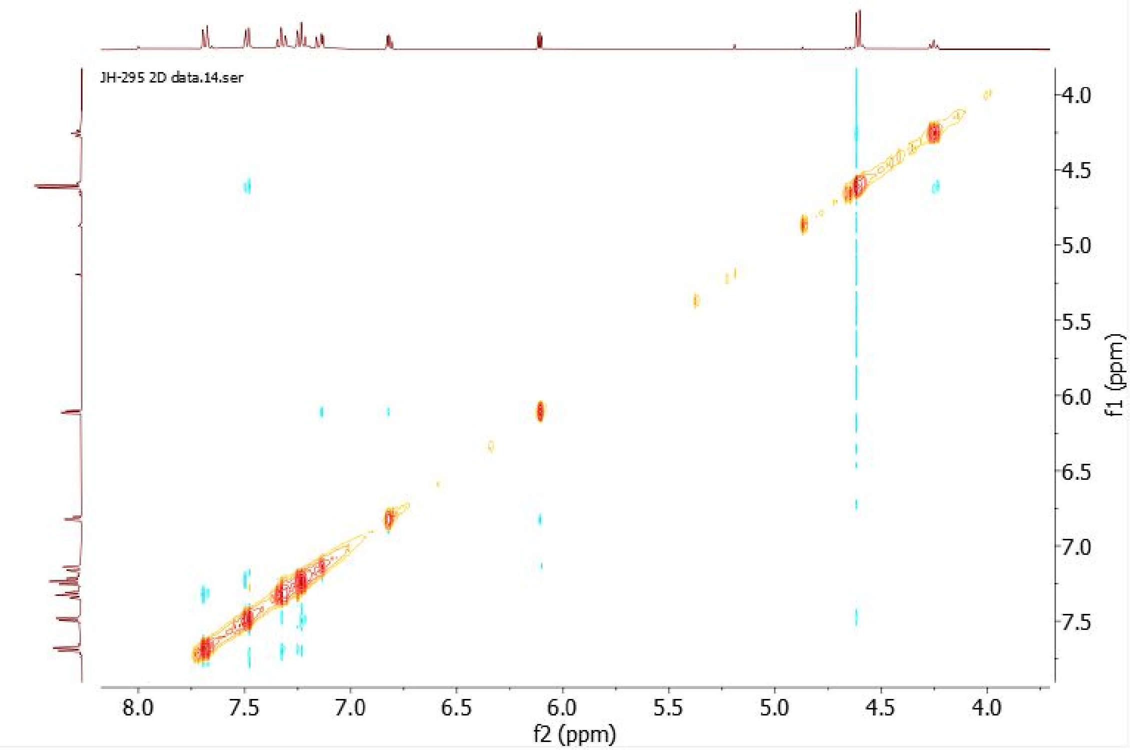

Fig S59: NOESY NMR spectrum of (9*H*-fluoren-9-yl)methyl 2-acetyl-1*H*-pyrrole-1-carboxylate (**16**) in $CDCl_3$

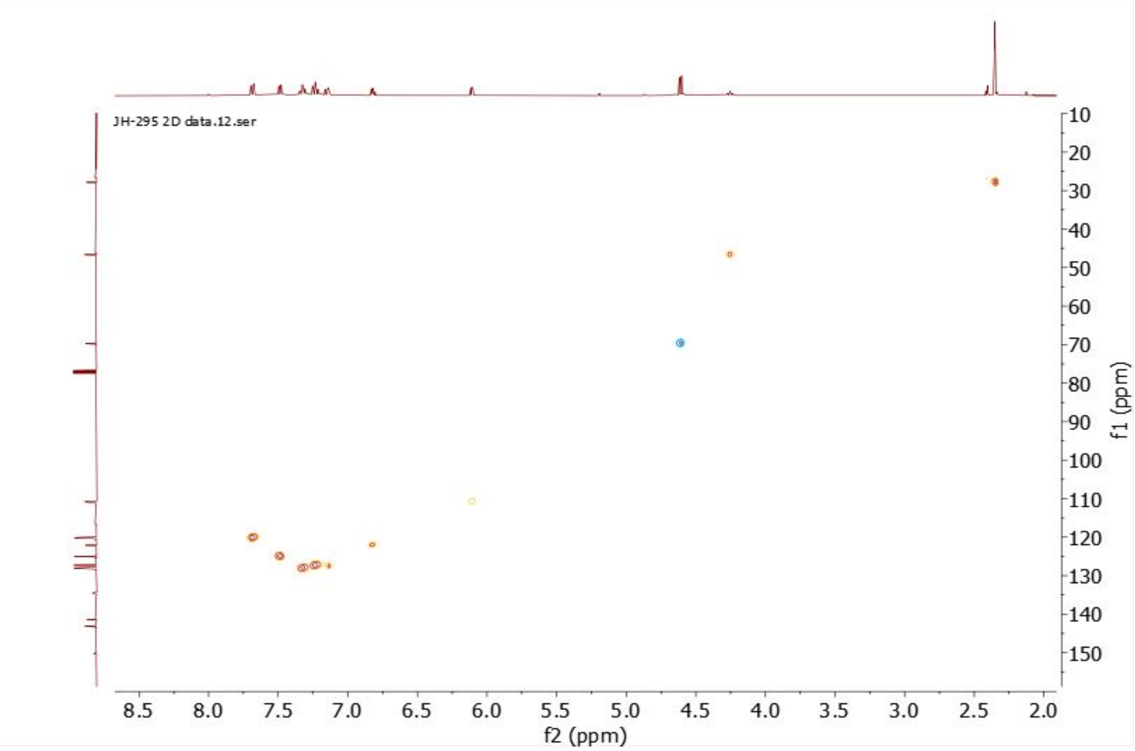

Fig S60: HSQC NMR spectrum of (9*H*-fluoren-9-yl)methyl 2-acetyl-1*H*-pyrrole-1-carboxylate (**16**) in $CDCl_3$

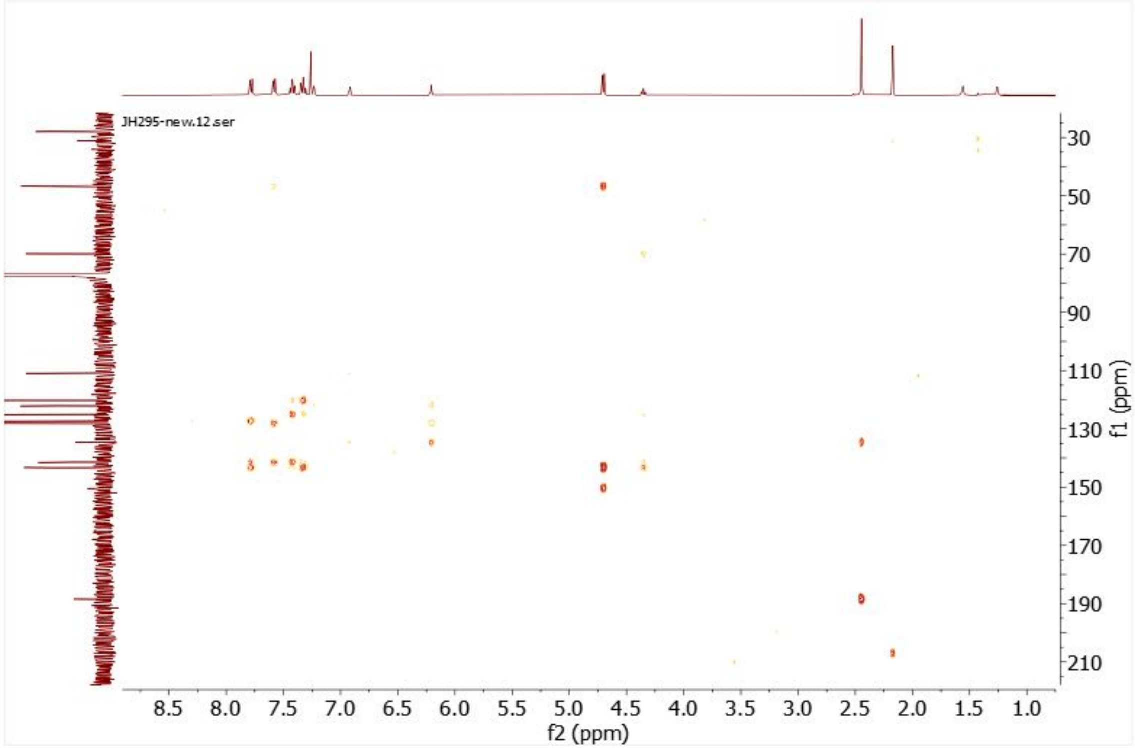

Fig S61: HMBC NMR spectrum of (9*H*-fluoren-9-yl)methyl 2-acetyl-1*H*-pyrrole-1-carboxylate (**16**) in $CDCl_3$

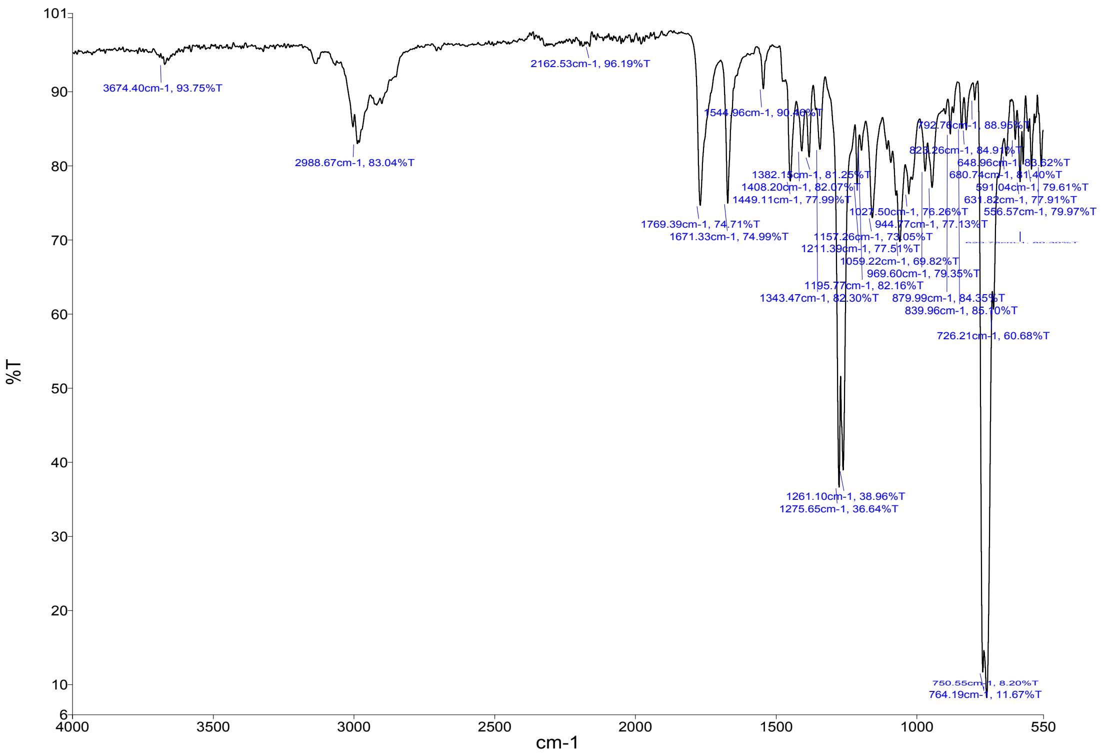

Fig S62: IR Spectrum of 9*H*-fluoren-9-yl)methyl 2-acetyl-1*H*-pyrrole-1-carboxylate (**16**) in $CDCl_3$

2,2,2-trichloroethyl 2-acetyl-1*H*-pyrrole-1-carboxylate (17)

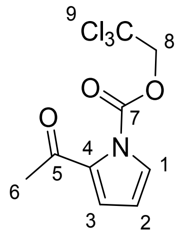

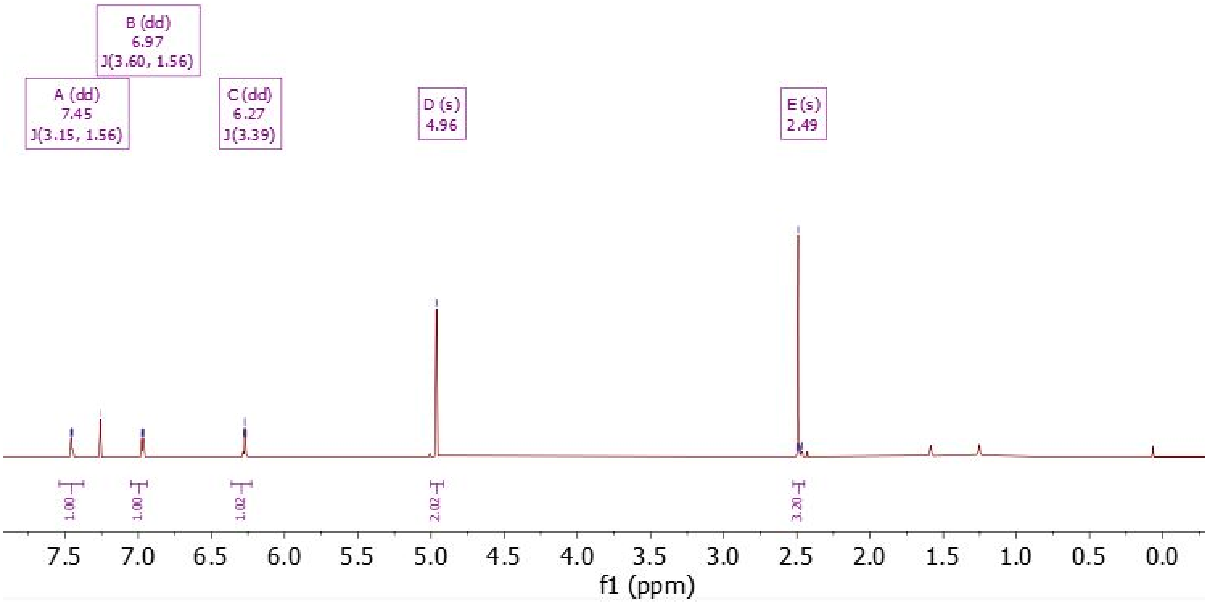

Fig S63: ^{1}H NMR (400 MHz) spectrum of 2,2,2-trichloroethyl 2-acetyl-1*H*-pyrrole-1-carboxylate (**17**) in $CDCl_3$

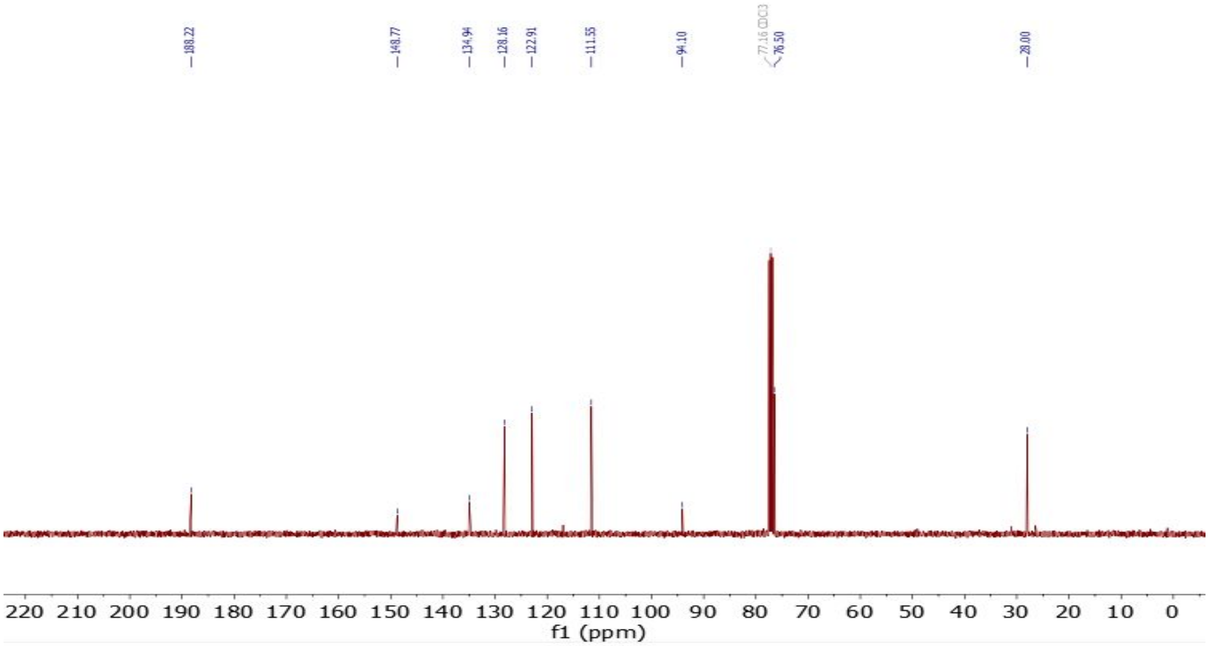

Fig S64: $^{13}C\{^{1}H\}$ NMR (101 MHz) spectrum of 2,2,2-trichloroethyl 2-acetyl-1*H*-pyrrole-1-carboxylate (**17**) in $CDCl_3$

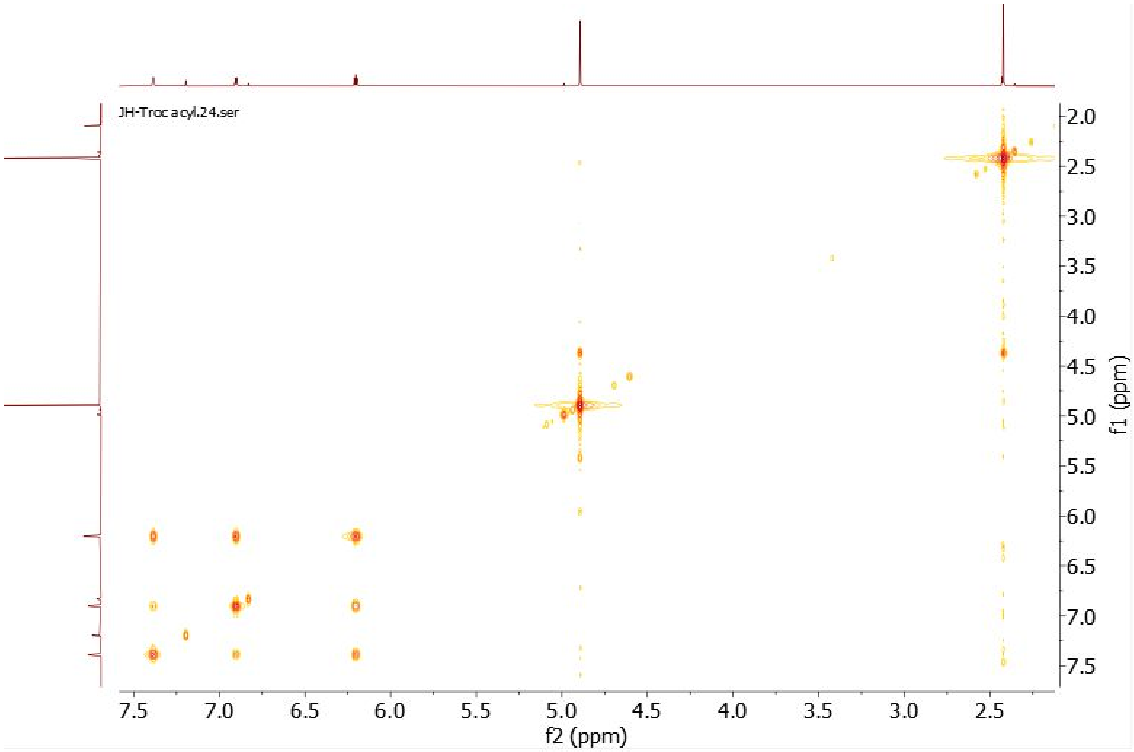

Fig S65: COSY NMR spectrum of 2,2,2-trichloroethyl 2-acetyl-1*H*-pyrrole-1-carboxylate (**17**) in $CDCl_3$

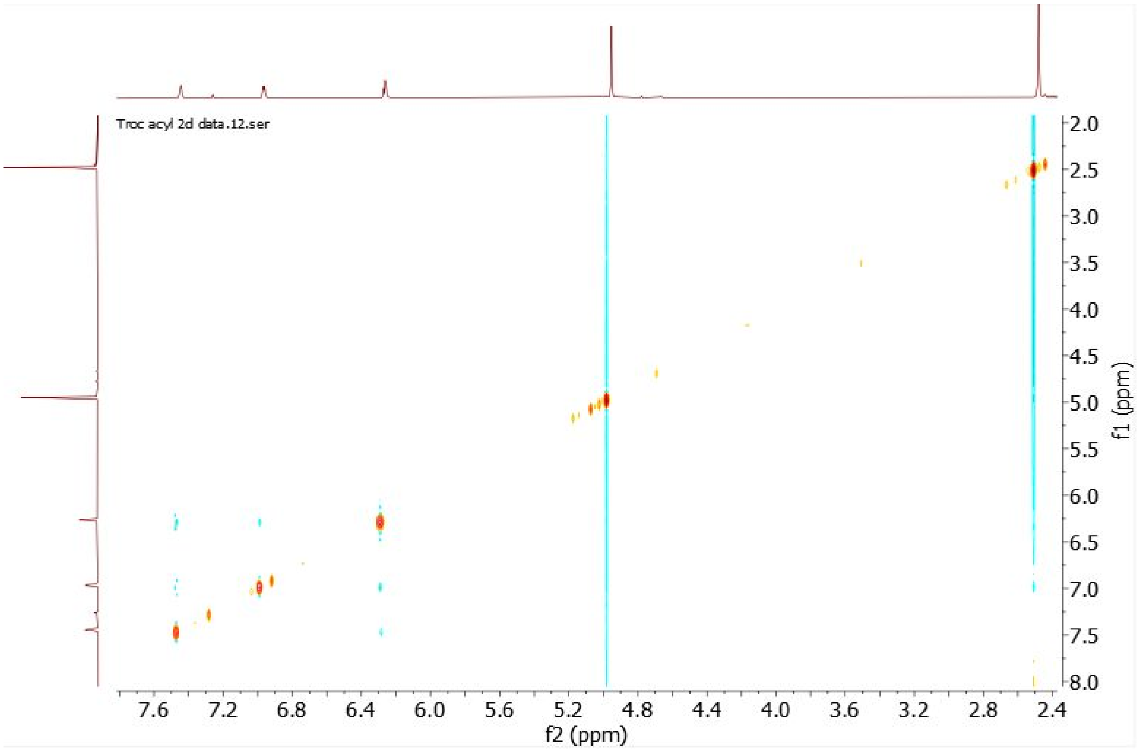

Fig S66: NOESY NMR spectrum of 2,2,2-trichloroethyl 2-acetyl-1*H*-pyrrole-1-carboxylate (**17**) in $CDCl_3$

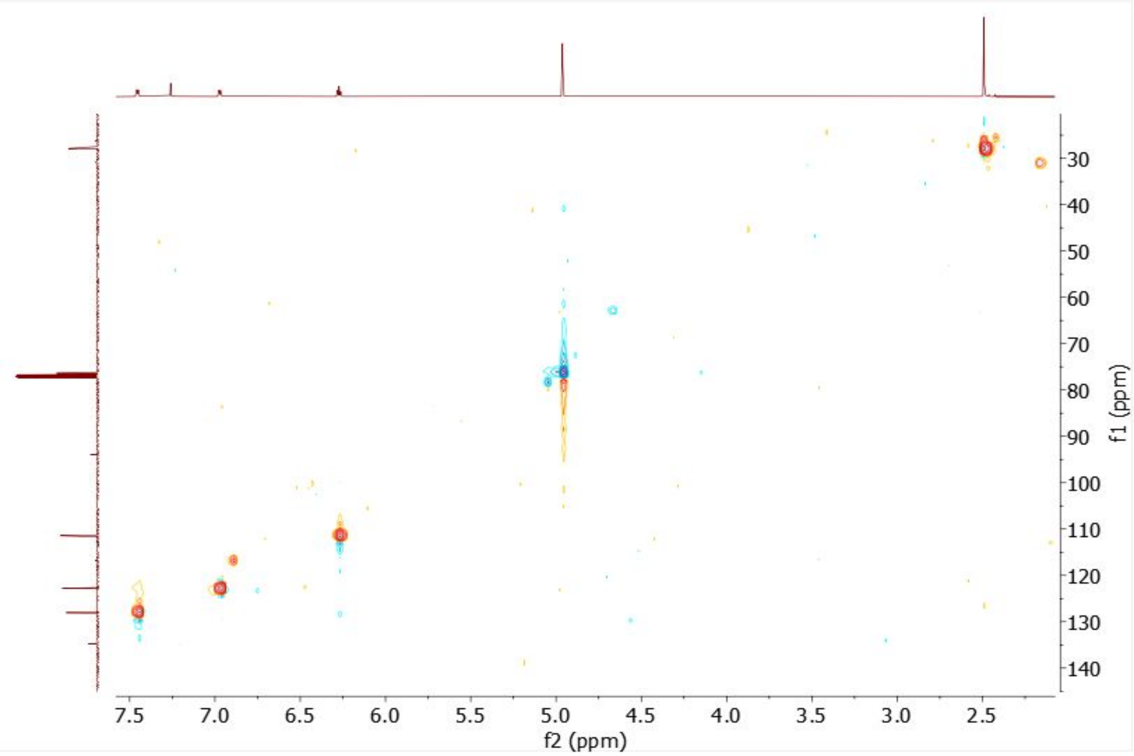

Fig S67: HSQC NMR spectrum of 2,2,2-trichloroethyl 2-acetyl-1*H*-pyrrole-1-carboxylate (**17**) in $CDCl_3$

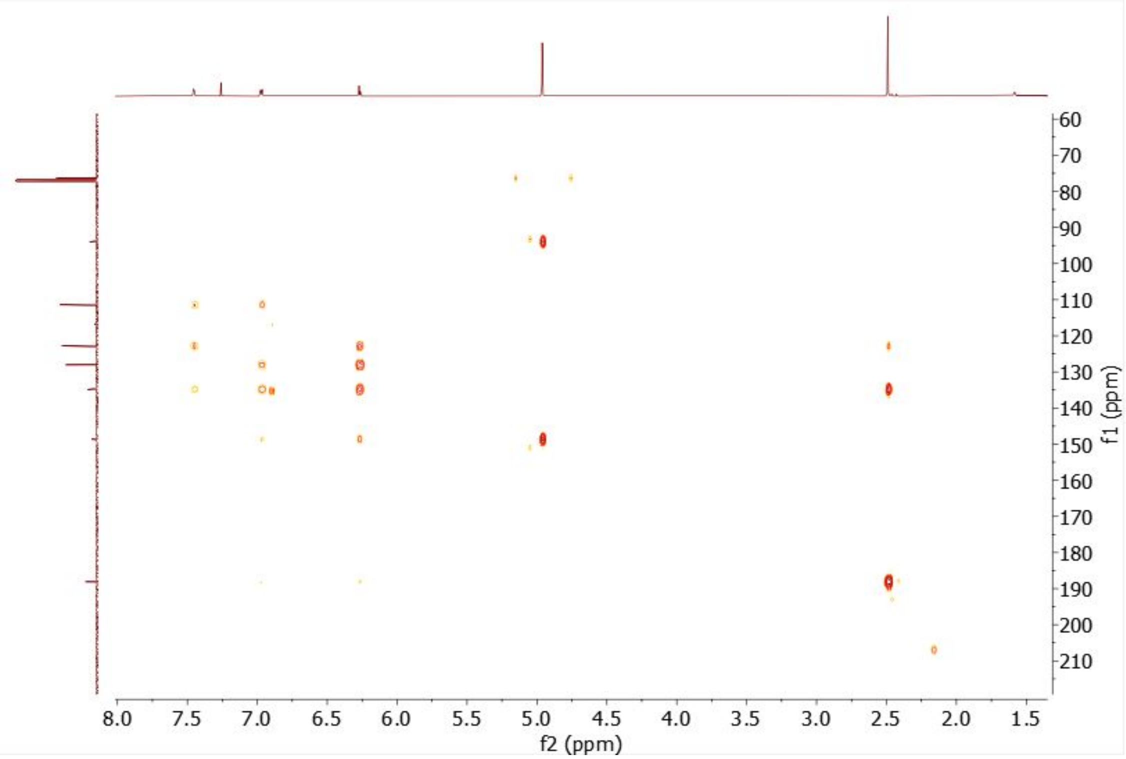

Fig S68: HMBC NMR spectrum of 2,2,2-trichloroethyl 2-acetyl-1*H*-pyrrole-1-carboxylate (**17**) in $CDCl_3$

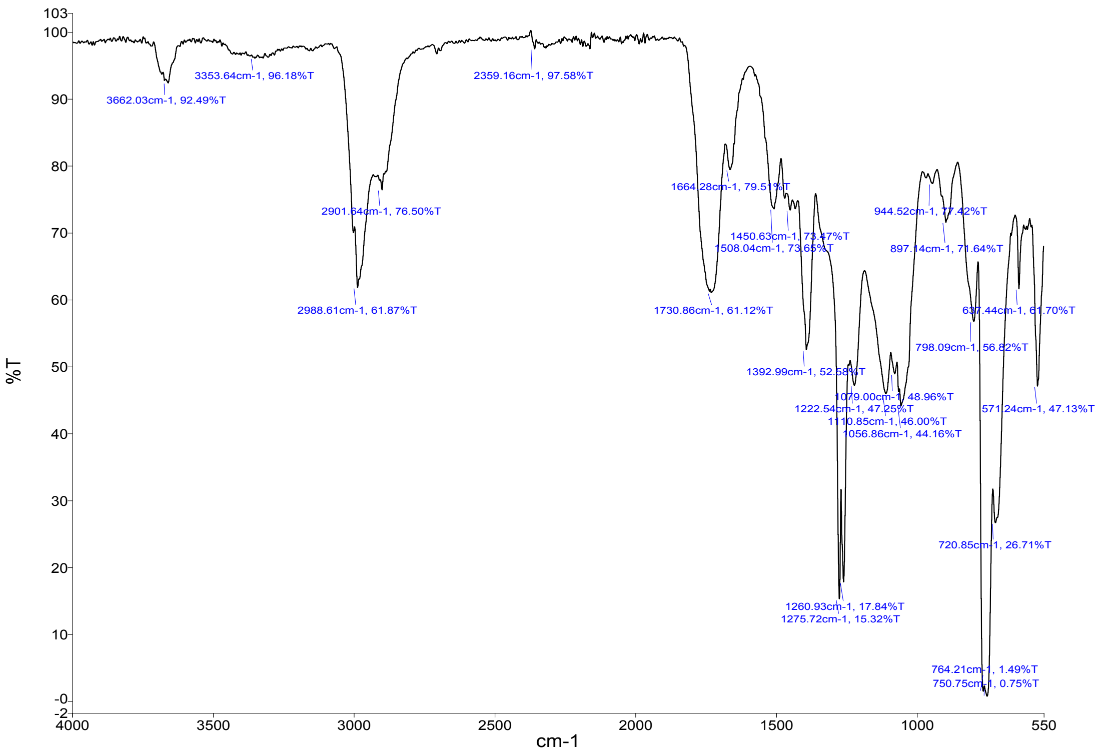

Fig S69: IR spectrum (neat) of 2,2,2-trichloroethyl 2-acetyl-1*H*-pyrrole-1-carboxylate (**17**).

1-(1*H*-Pyrrol-2-yl)ethan-1-one (18)

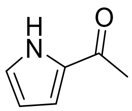

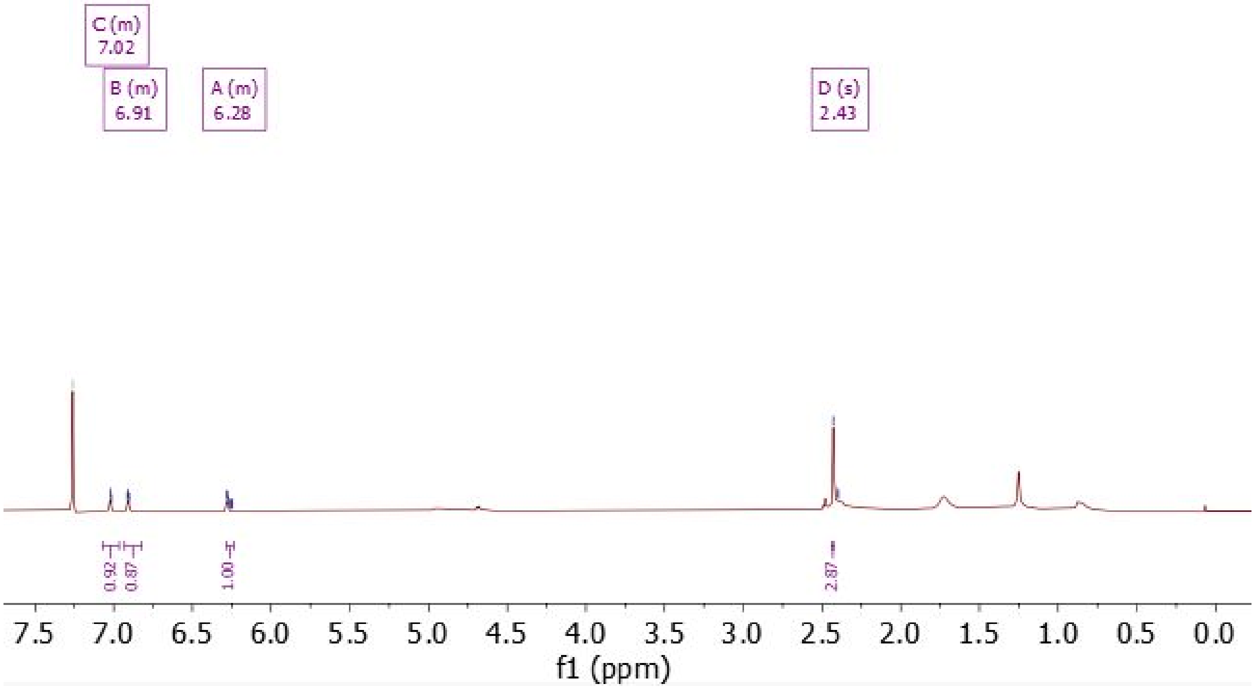

Fig S70: ^{1}H NMR (400 MHz) spectrum of 1-(1*H*-pyrrol-2-yl)ethan-1-one (**18**) in $CDCl_3$

2,2,2-Trichloroethyl 3-tetradecanoyl-1*H*-pyrrole-1-carboxylate (19)

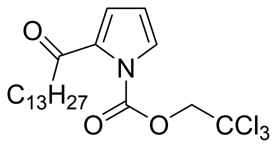

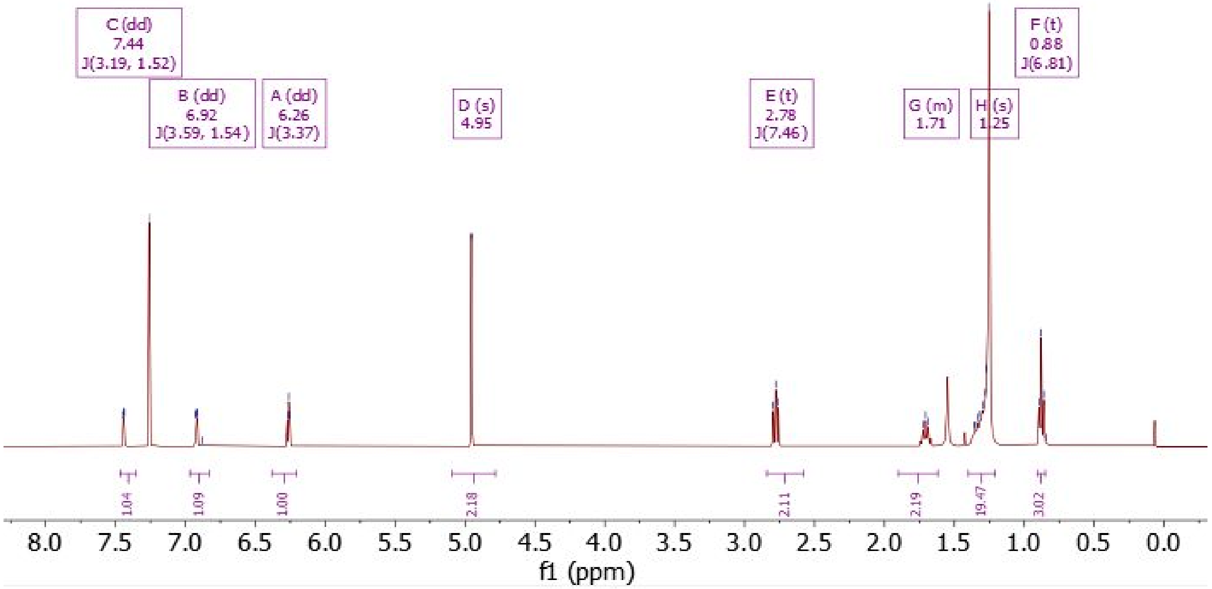

Fig S71: ^{1}H NMR (400 MHz) spectrum of 2,2,2-trichloroethyl 3-tetradecanoyl-1*H*-pyrrole-1-carboxylate (**19**) in $CDCl_3$

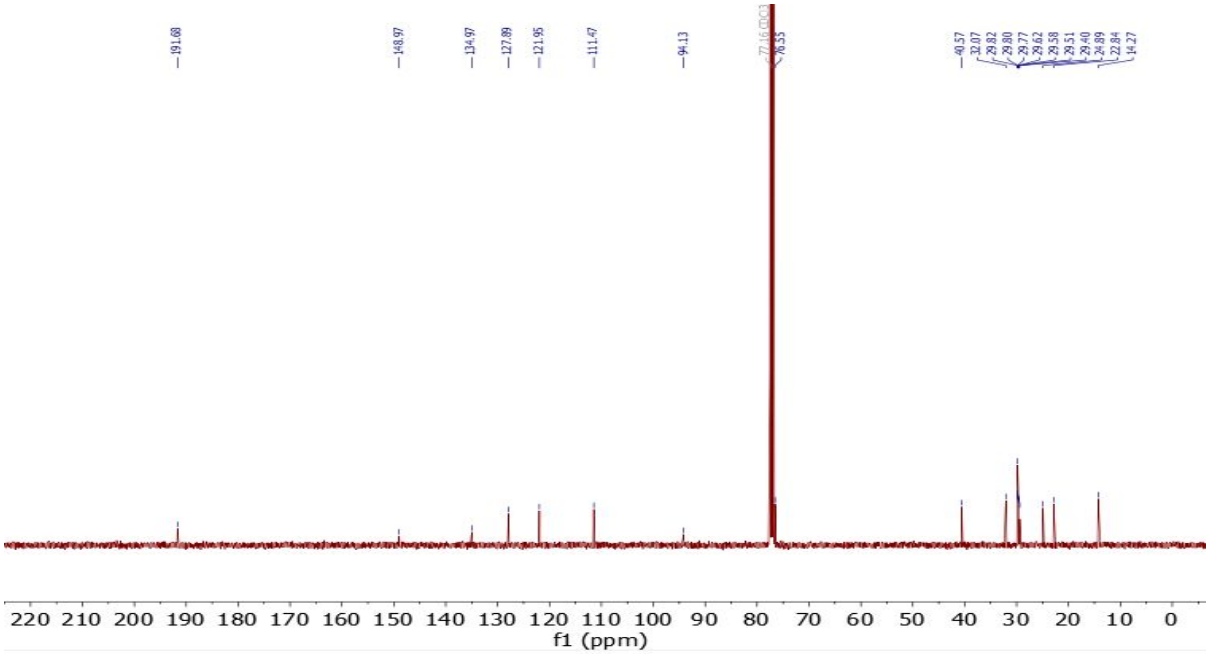

Fig S72: $^{13}C\{^{1}H\}$ NMR (101 MHz) spectrum of 2,2,2-trichloroethyl 3-tetradecanoyl-1*H*-pyrrole-1-carboxylate (**19**) in $CDCl_3$

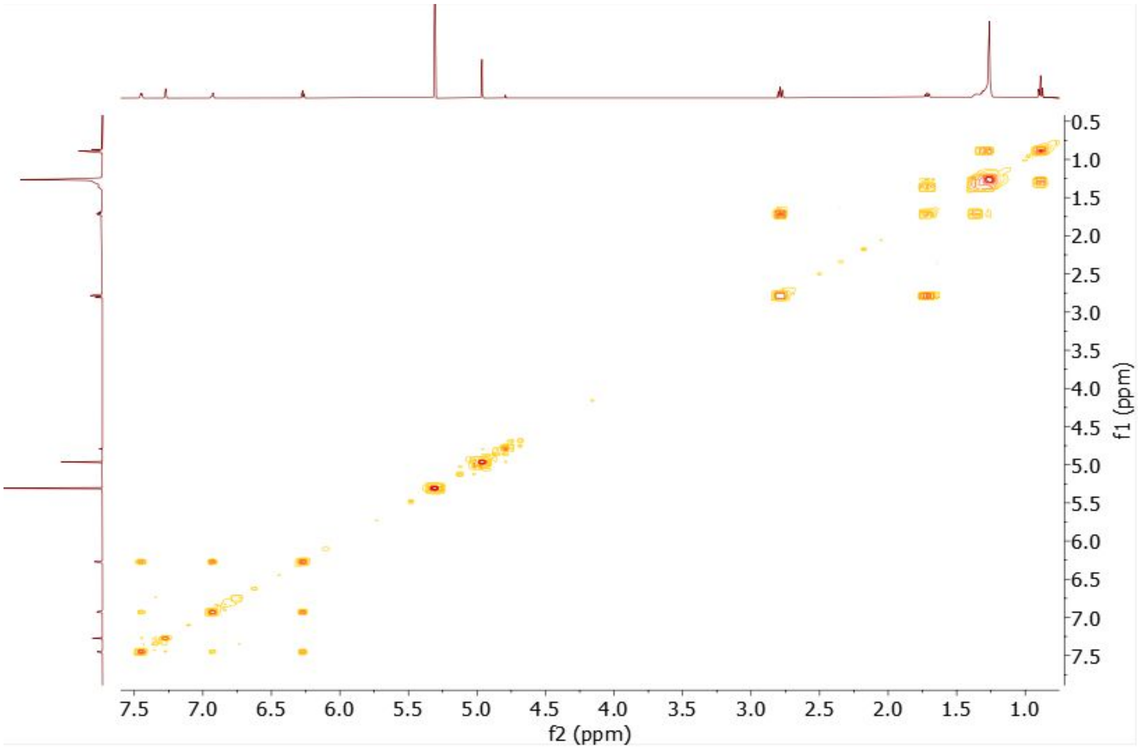

Fig S73: COSY NMR spectrum of 2,2,2-trichloroethyl 3-tetradecanoyl-1*H*-pyrrole-1-carboxylate (**19**) in $CDCl_3$

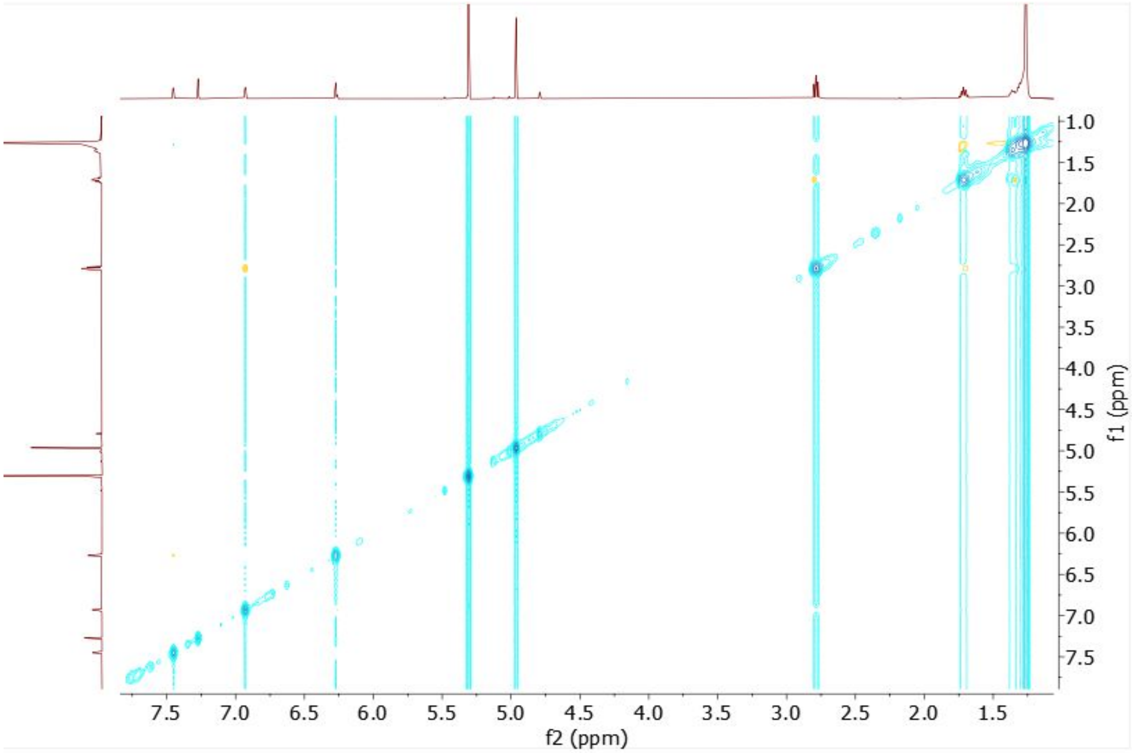

Fig S74: NOESY NMR spectrum of 2,2,2-trichloroethyl 3-tetradecanoyl-1*H*-pyrrole-1-carboxylate (**19**) in $CDCl_3$

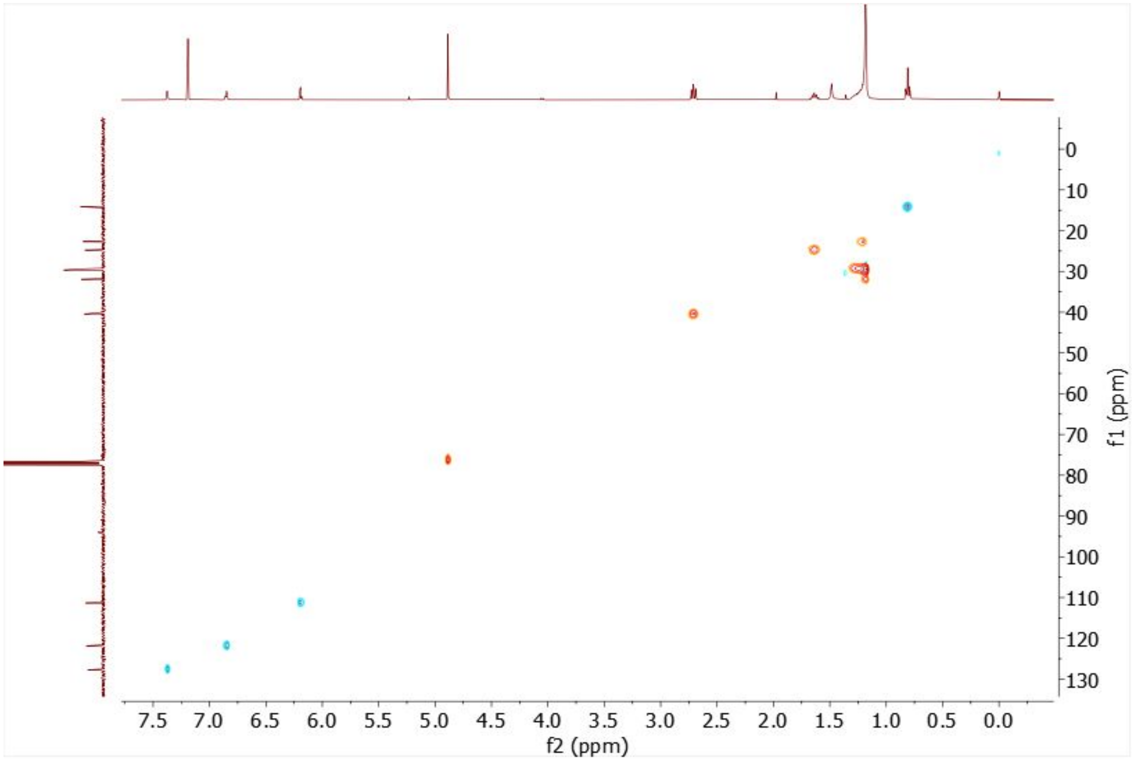

Fig S75: HSQC NMR spectrum of 2,2,2-trichloroethyl 3-tetradecanoyl-1*H*-pyrrole-1-carboxylate (**19**) in $CDCl_3$

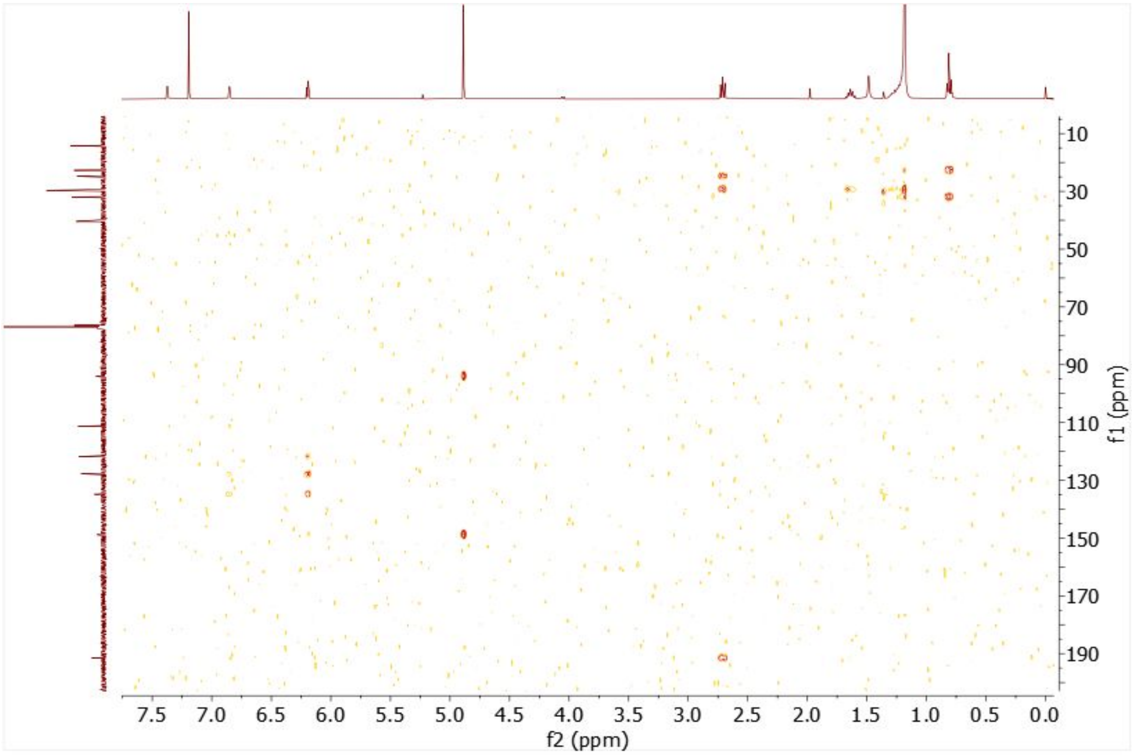

Fig S76: HMBC NMR spectrum of 2,2,2-trichloroethyl 3-tetradecanoyl-1*H*-pyrrole-1-carboxylate (**19**) in $CDCl_3$

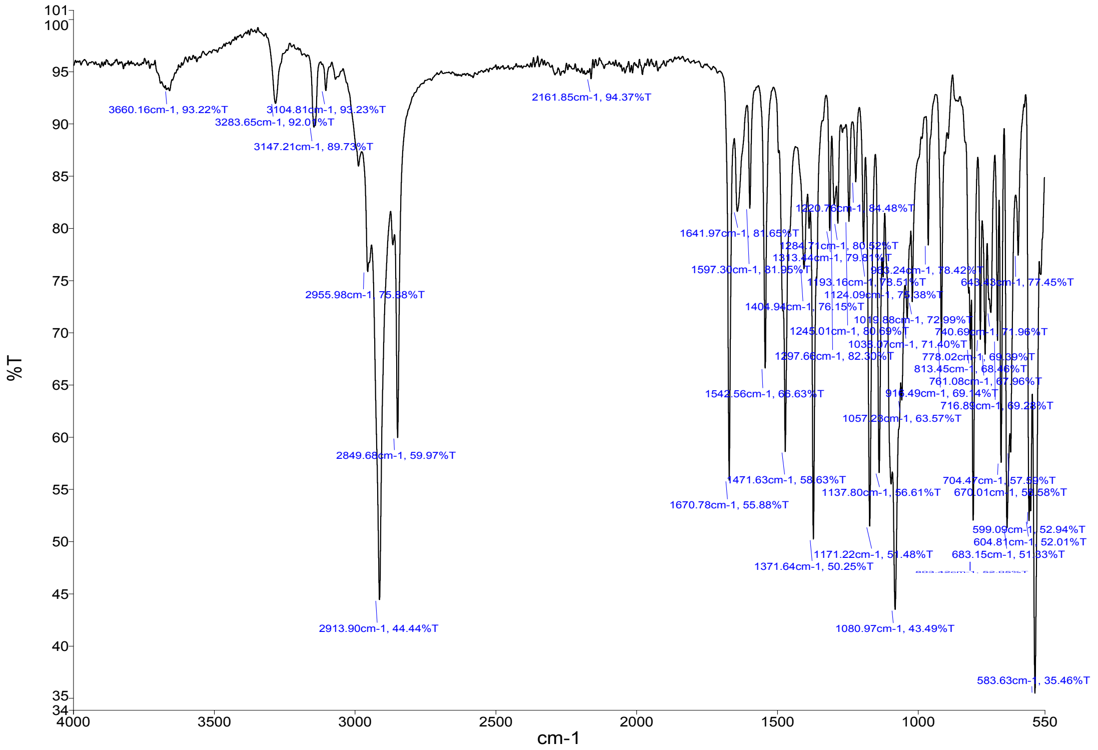

Fig S77: IR (neat) spectrum of 2,2,2-trichloroethyl 3-tetradecanoyl-1*H*-pyrrole-1-carboxylate (**19**)

1-(1*H*-Pyrrol-3-yl)tetradecan-1-one (20)

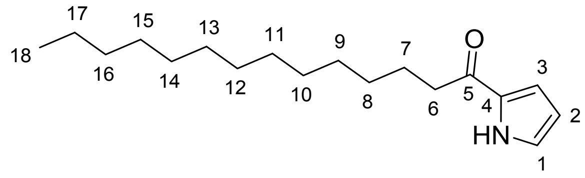

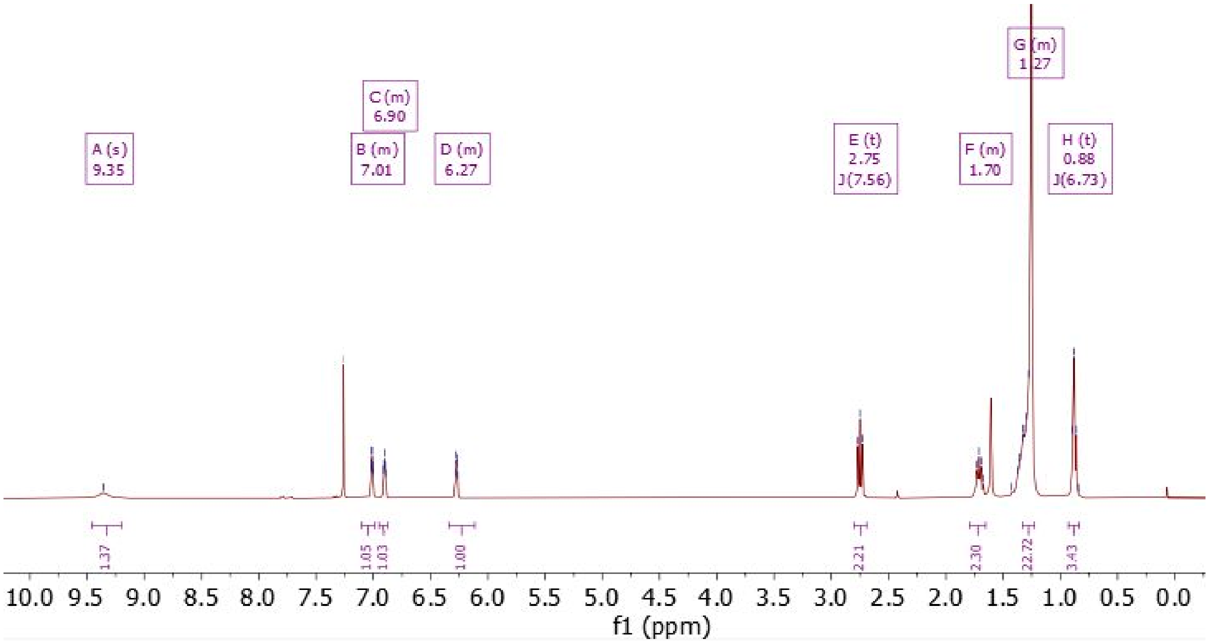

Fig S78: ^{1}H (400 MHz) NMR spectrum of 1-(1*H*-pyrrol-2-yl)tetradecan-1-one (**20**) in $CDCl_3$

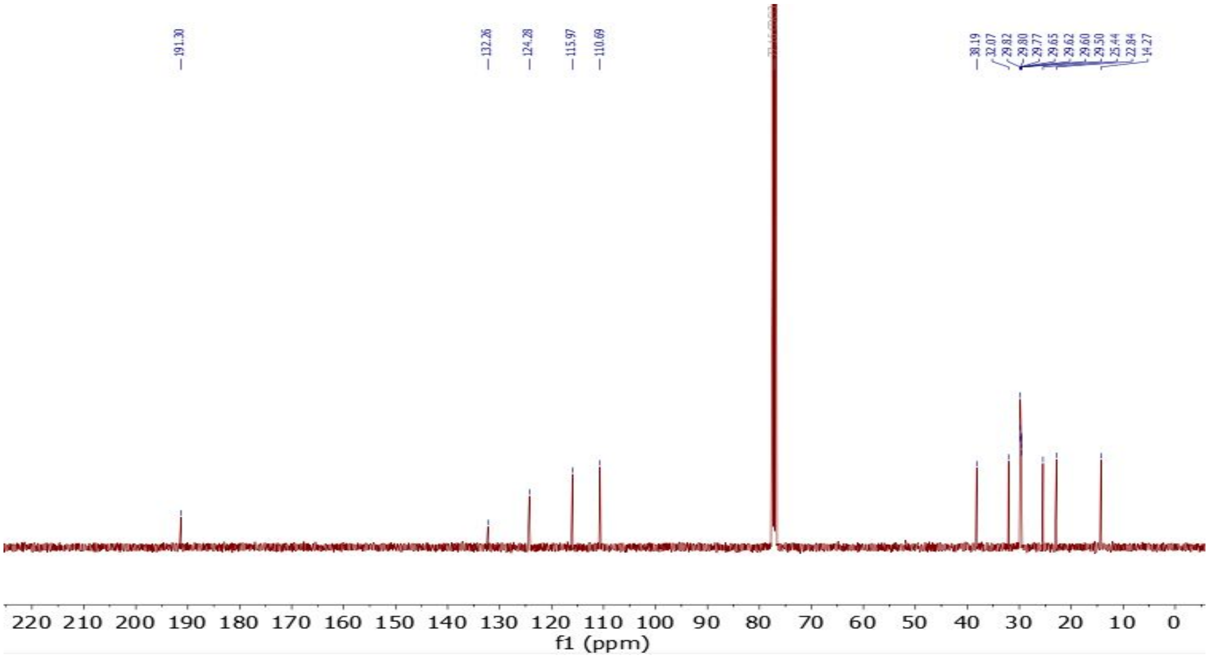

Fig S79: $^{13}C\{^{1}H\}$ NMR (101 MHz) spectrum of 1-(1*H*-pyrrol-2-yl)tetradecan-1-one (**20**) in $CDCl_3$

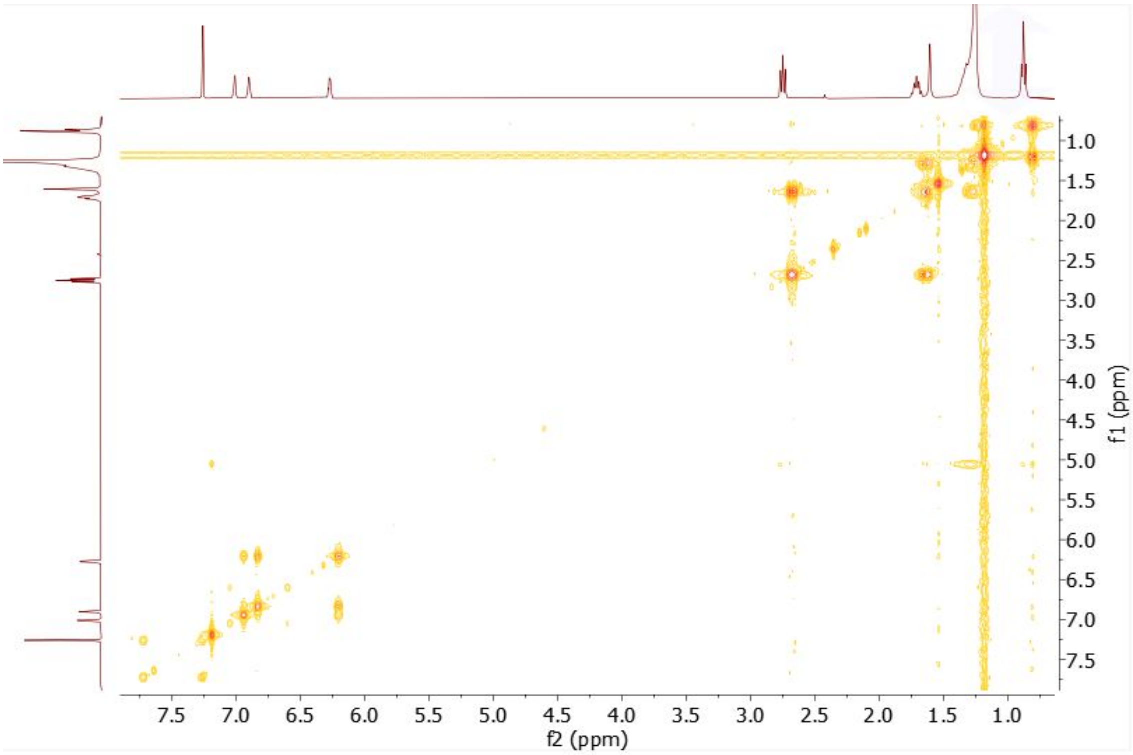

Fig S80: COSY NMR spectrum of 1-(1*H*-pyrrol-2-yl)tetradecan-1-one (**20**) in $CDCl_3$

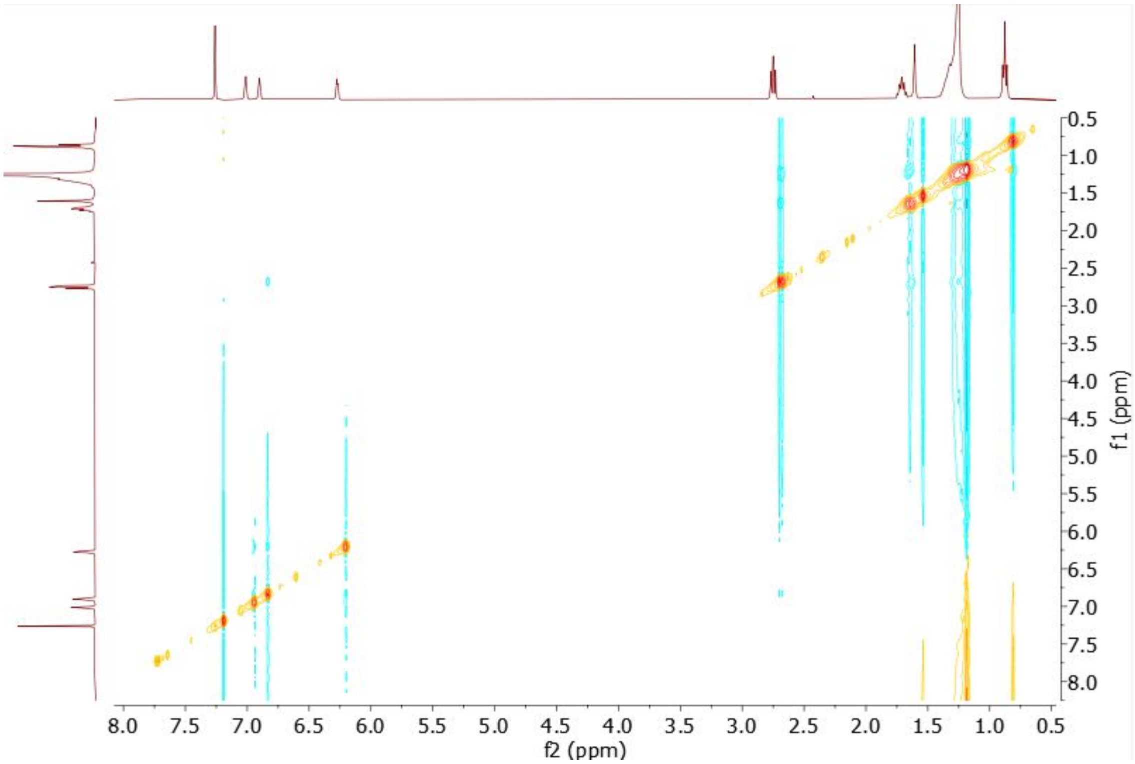

Fig S81: NOESY NMR spectrum of 1-(1*H*-pyrrol-2-yl)tetradecan-1-one (**20**) in $CDCl_3$

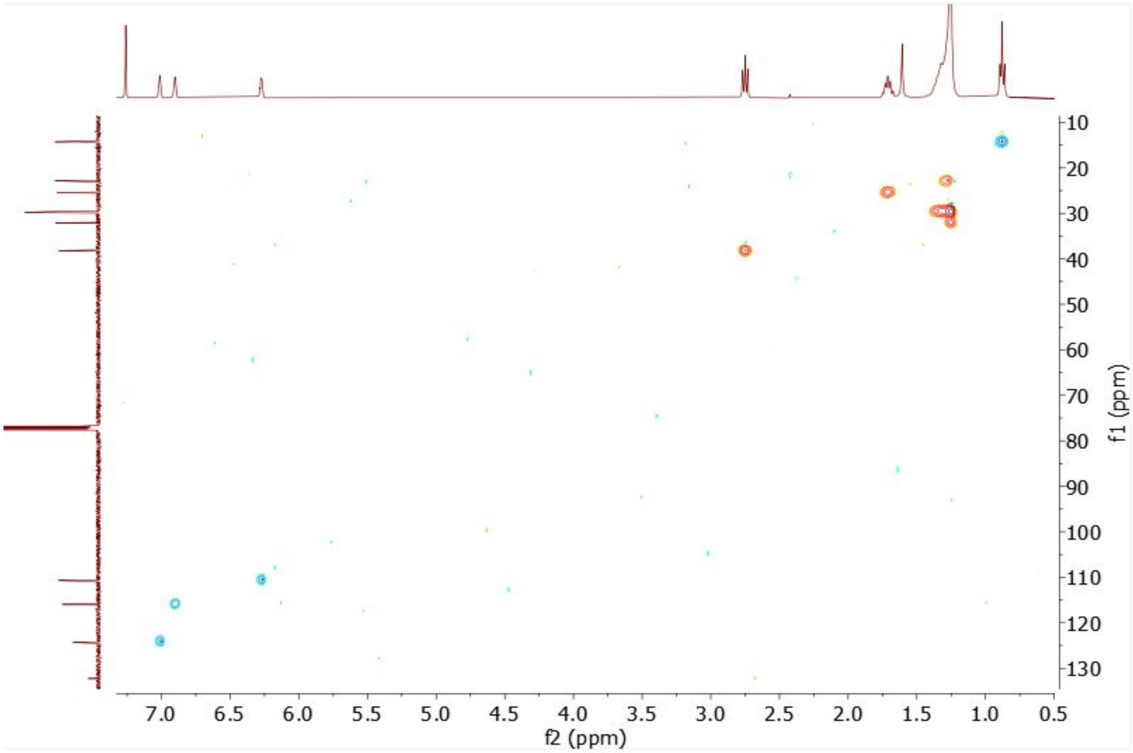

Fig S82: HSQC NMR spectrum of 1-(1*H*-pyrrol-2-yl)tetradecan-1-one (**20**) in $CDCl_3$

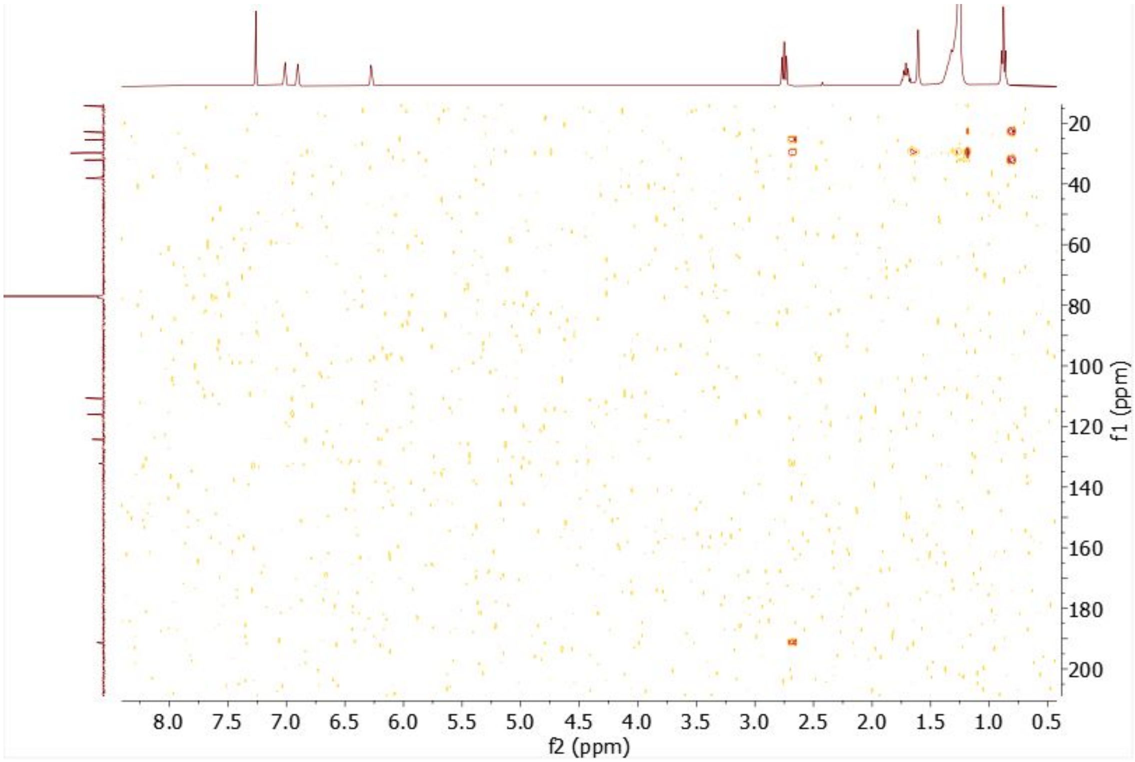

Fig S83: HMBC NMR spectrum of 1-(1*H*-pyrrol-2-yl)tetradecan-1-one (**20**) in $CDCl_3$

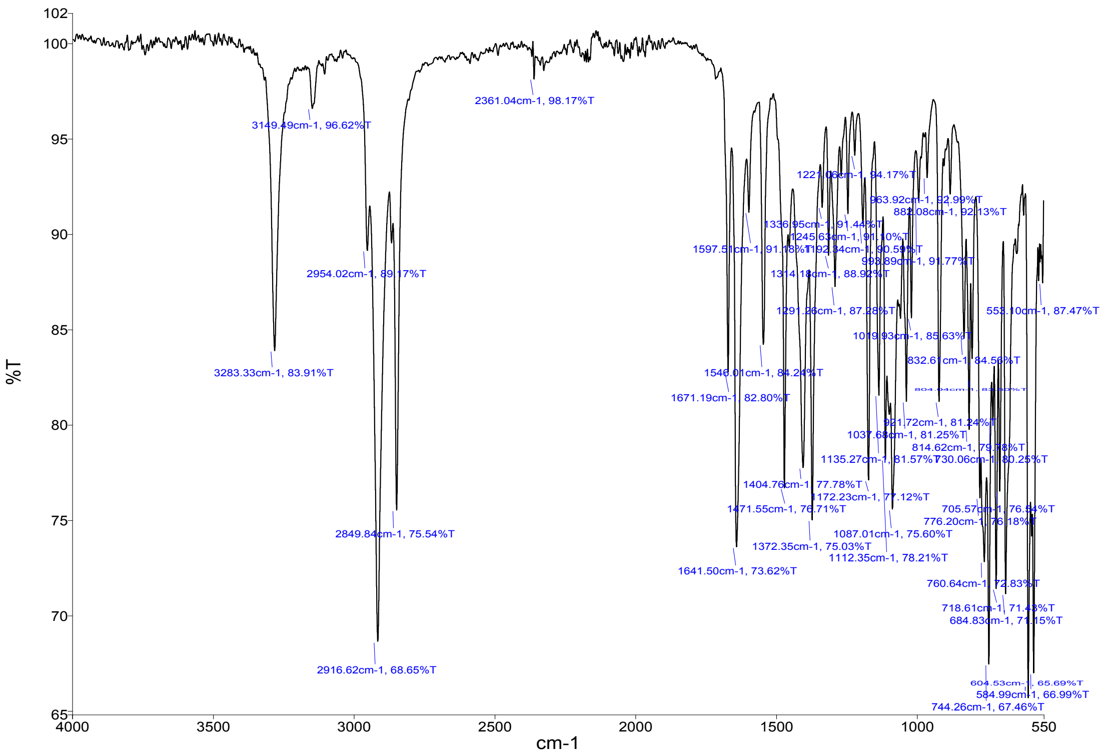

Fig S84: IR spectrum of 1-(1*H*-pyrrol-2-yl)tetradecan-1-one (**20**)

2,2,2-Trichloroethyl 2-(4-chlorobutanoyl)-1*H*-pyrrole-1-carboxylate (21)

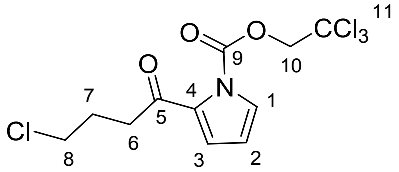

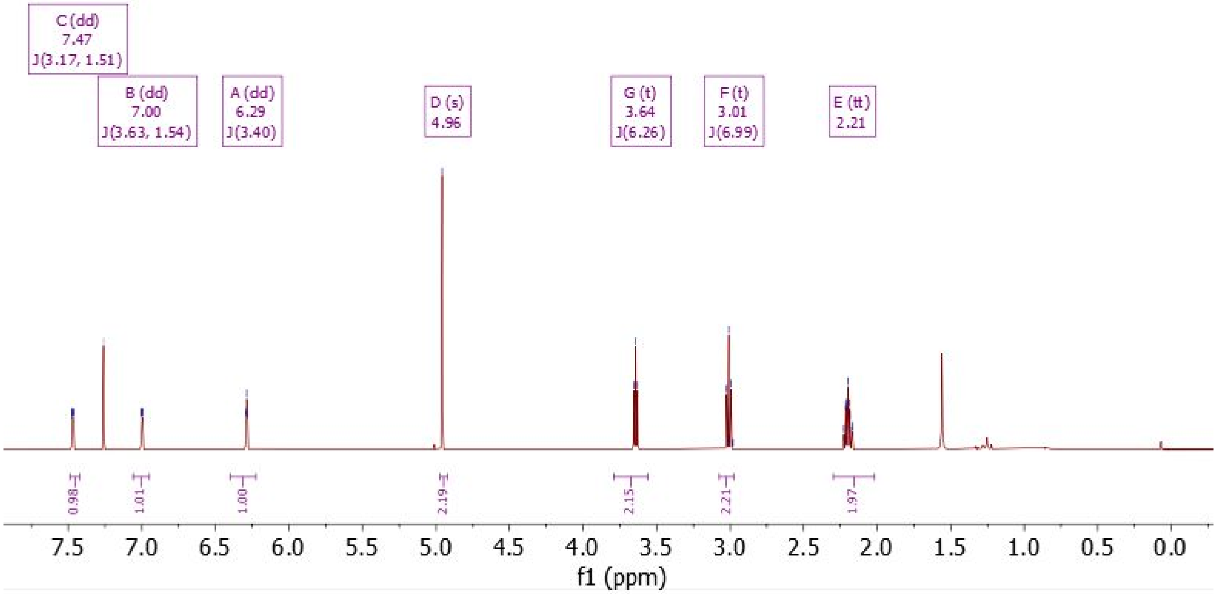

Fig S85: ^{1}H NMR (500 MHz) spectrum of 2,2,2-trichloroethyl 2-(4-chlorobutanoyl)-1*H*-pyrrole-1-carboxylate (**21**)

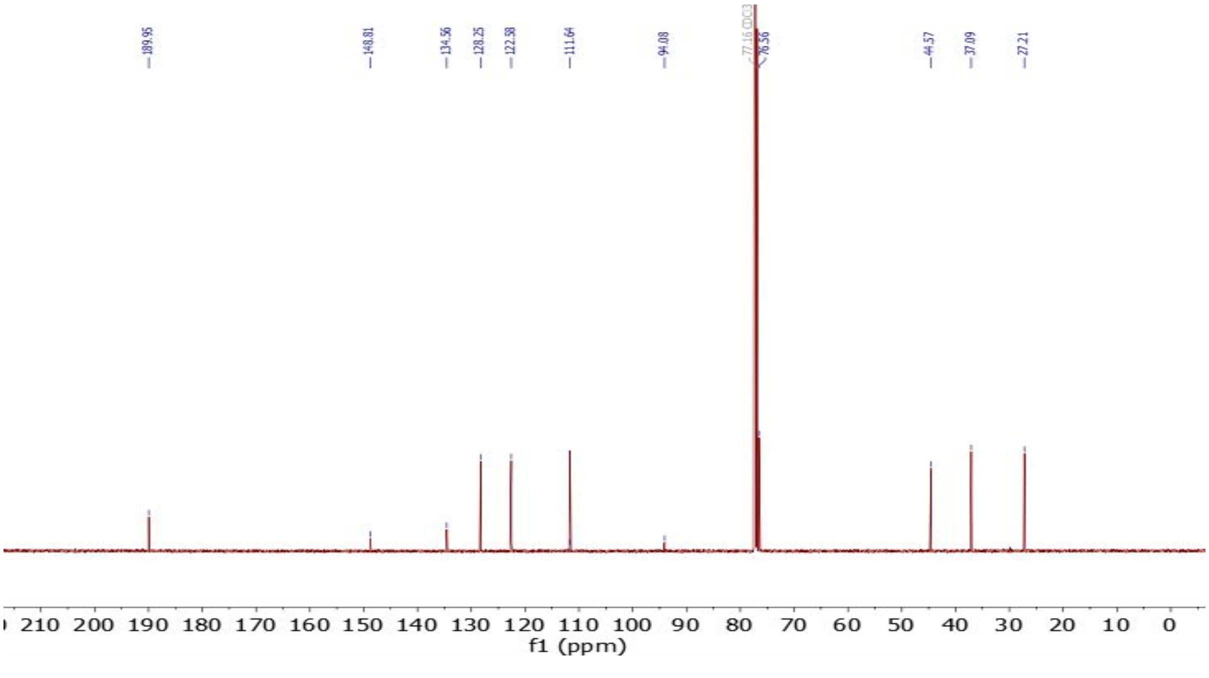

Fig S86: $^{13}C\{^{1}H\}$ NMR (126 MHz) spectrum of 2,2,2-trichloroethyl 2-(4-chlorobutanoyl)-1*H*-pyrrole-1-carboxylate (**21**)

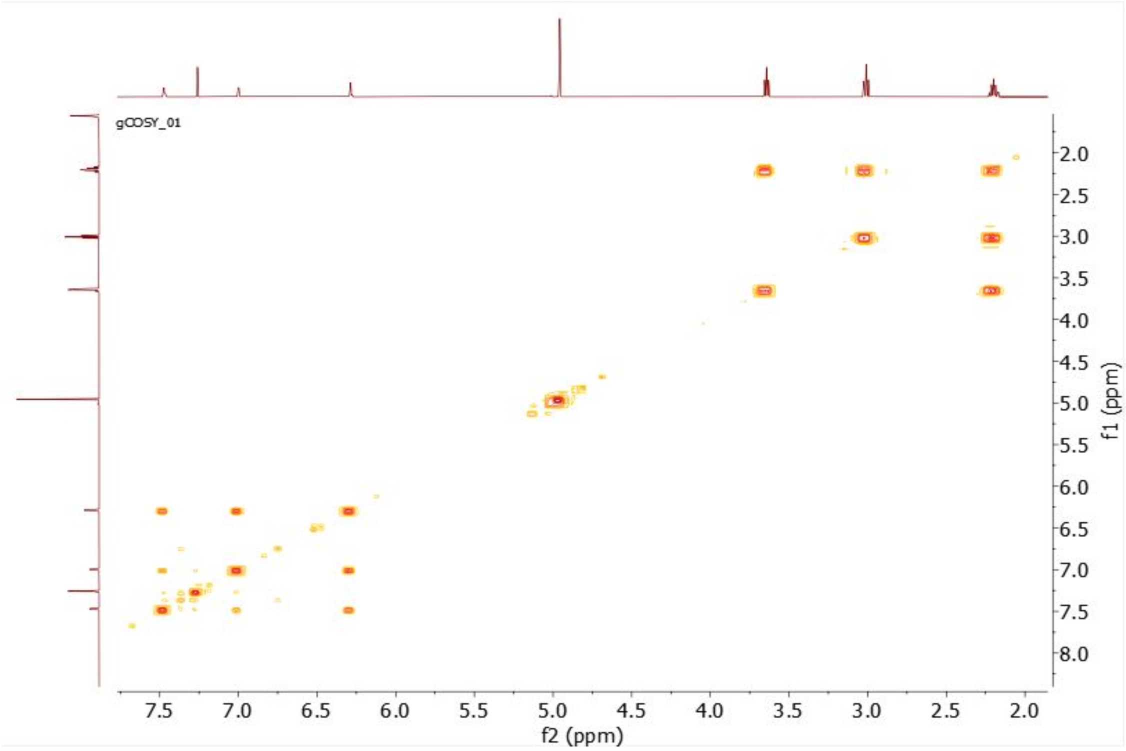

Fig S87: COSY NMR spectrum of 2,2,2-trichloroethyl 2-(4-chlorobutanoyl)-1*H*-pyrrole-1-carboxylate (**21**)

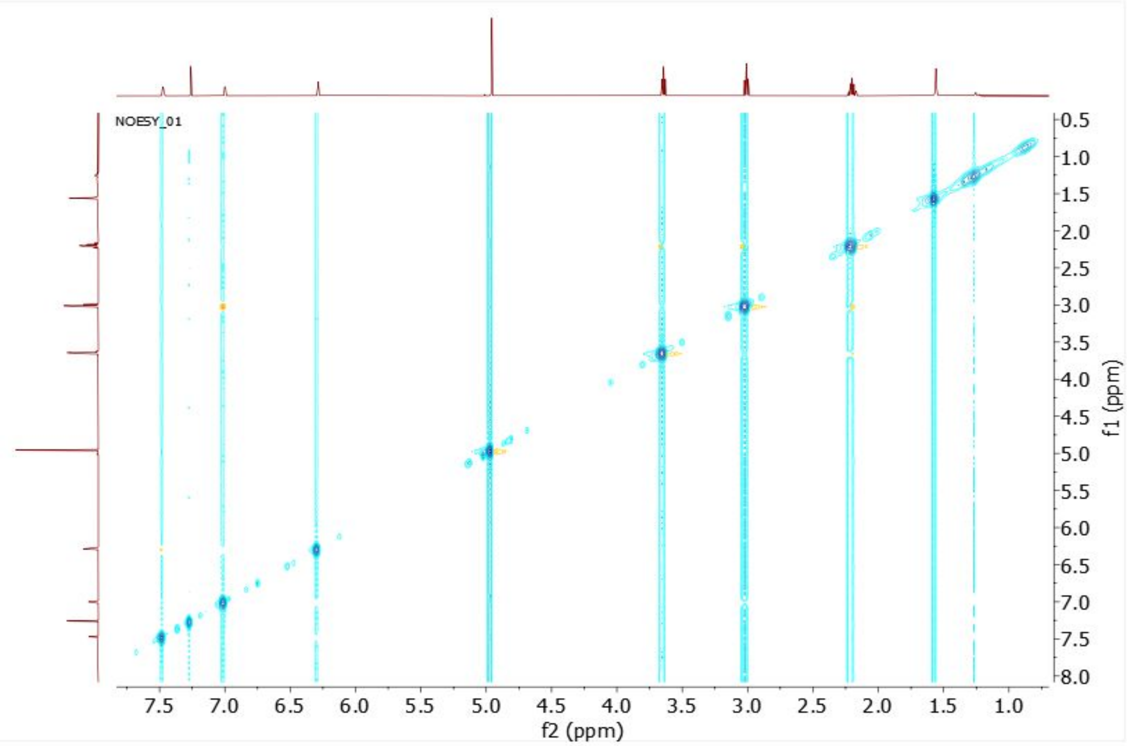

Fig S88: NOESY NMR spectrum of 2,2,2-trichloroethyl 2-(4-chlorobutanoyl)-1*H*-pyrrole-1-carboxylate (**21**)

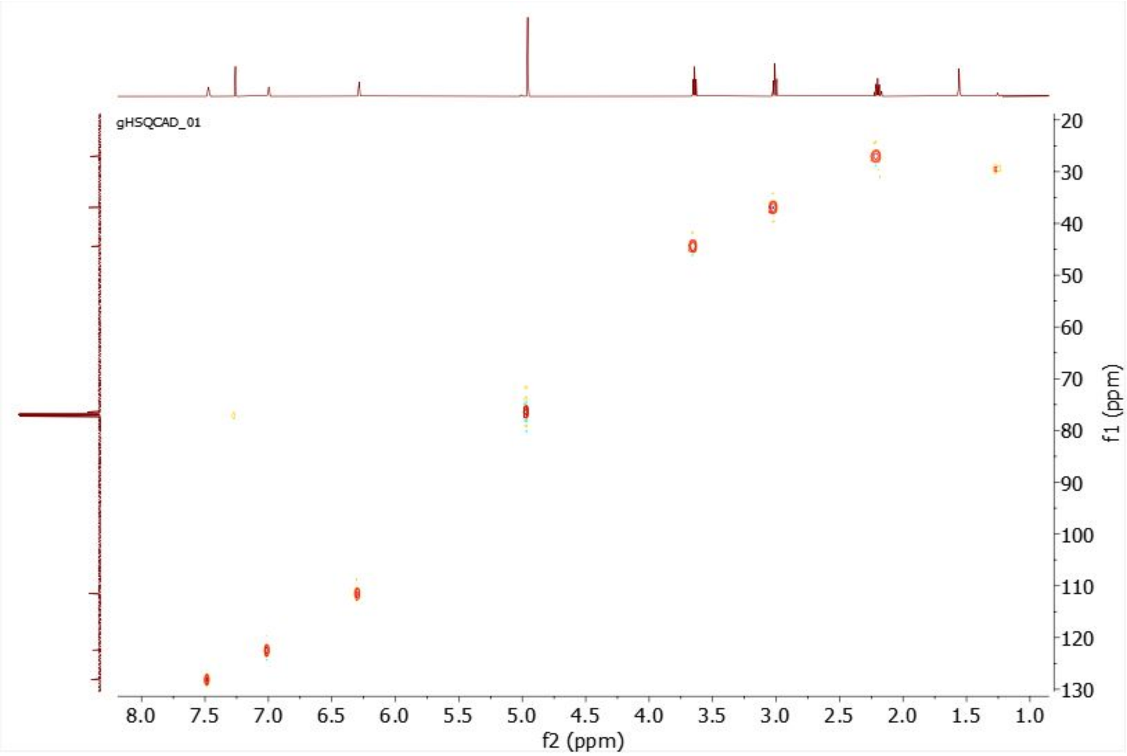

Fig S89: HSQC NMR spectrum of 2,2,2-trichloroethyl 2-(4-chlorobutanoyl)-1*H*-pyrrole-1-carboxylate (**21**)

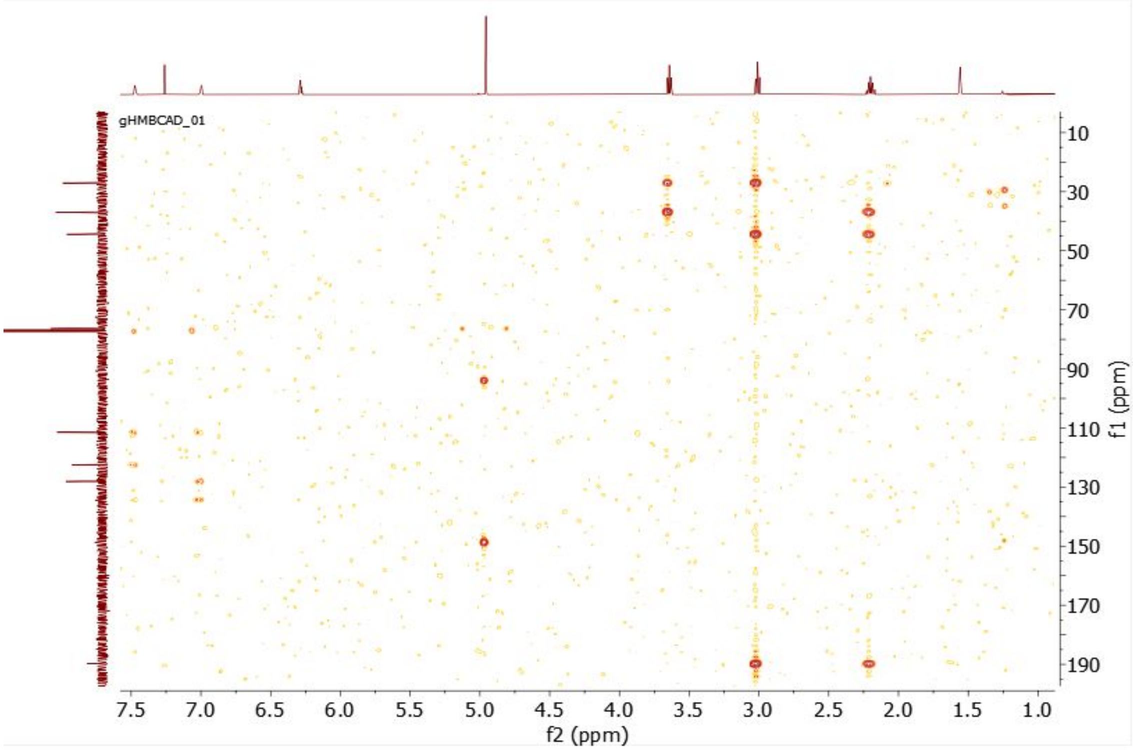

Fig S90: HMBC NMR spectrum of 2,2,2-trichloroethyl 2-(4-chlorobutanoyl)-1*H*-pyrrole-1-carboxylate (**21**)

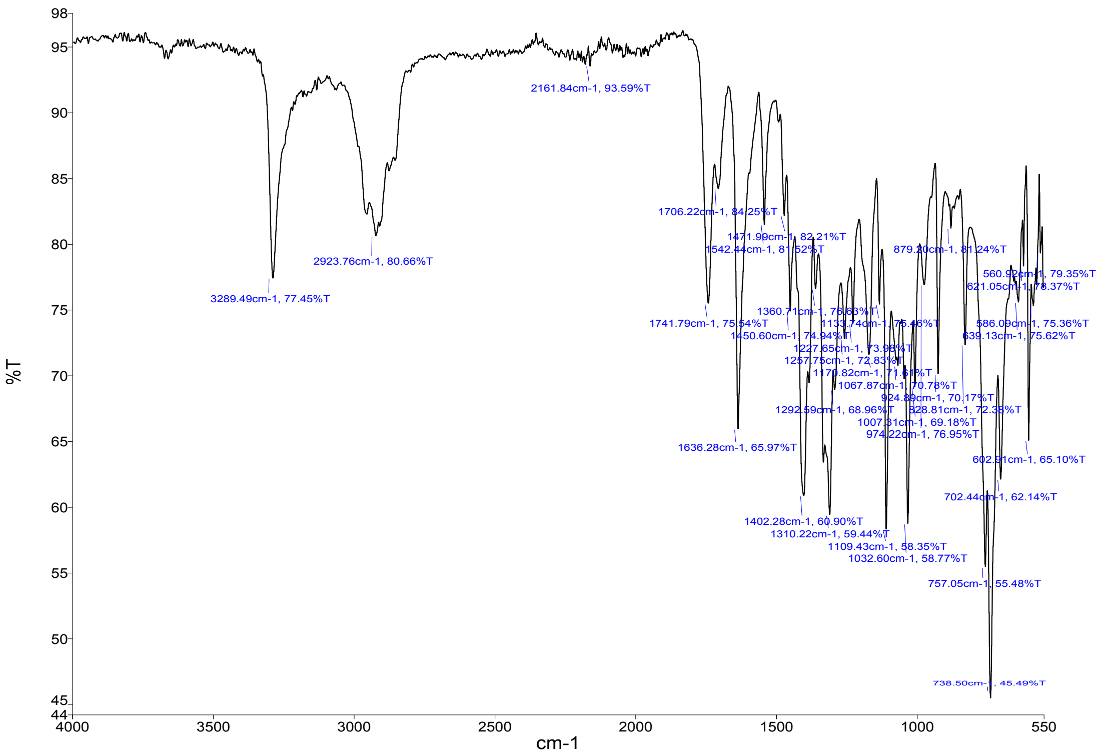

Fig S91: IR (neat) spectrum of 2,2,2-trichloroethyl 2-(4-chlorobutanoyl)-1*H*-pyrrole-1-carboxylate (**21**)

4-Chloro-1-(1*H*-pyrrol-2-yl)butan-1-one (22)

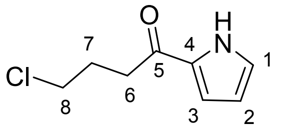

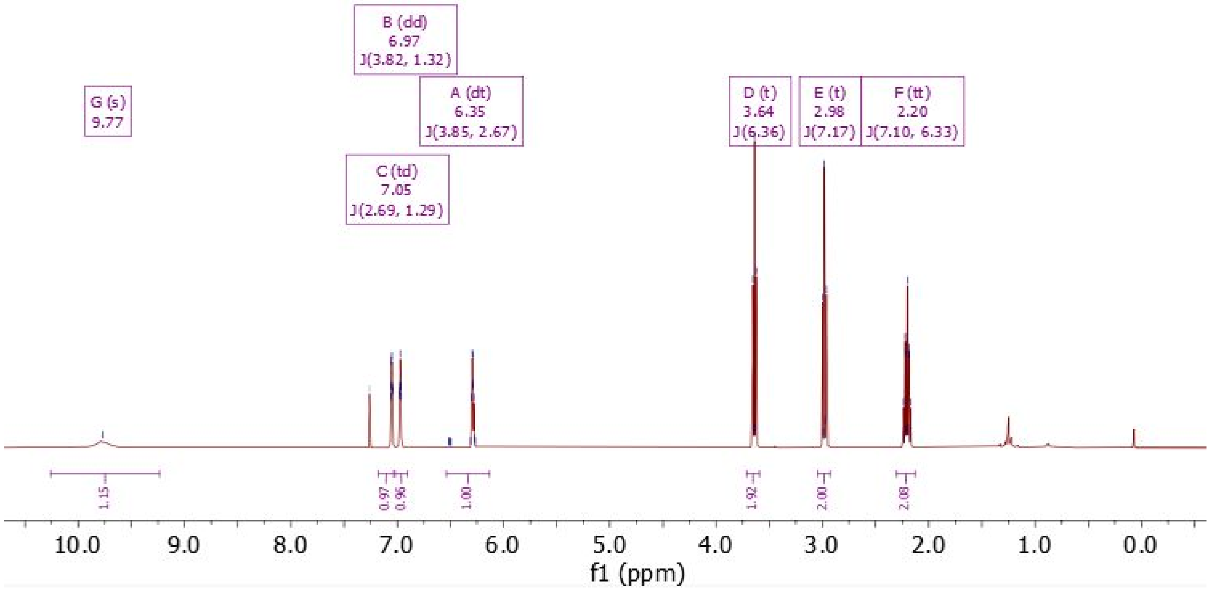

Fig S92: ^{1}H NMR (400 MHz) spectrum of 4-chloro-1-(1*H*-pyrrol-2-yl)butan-1-one (**22**) in $CDCl_3$

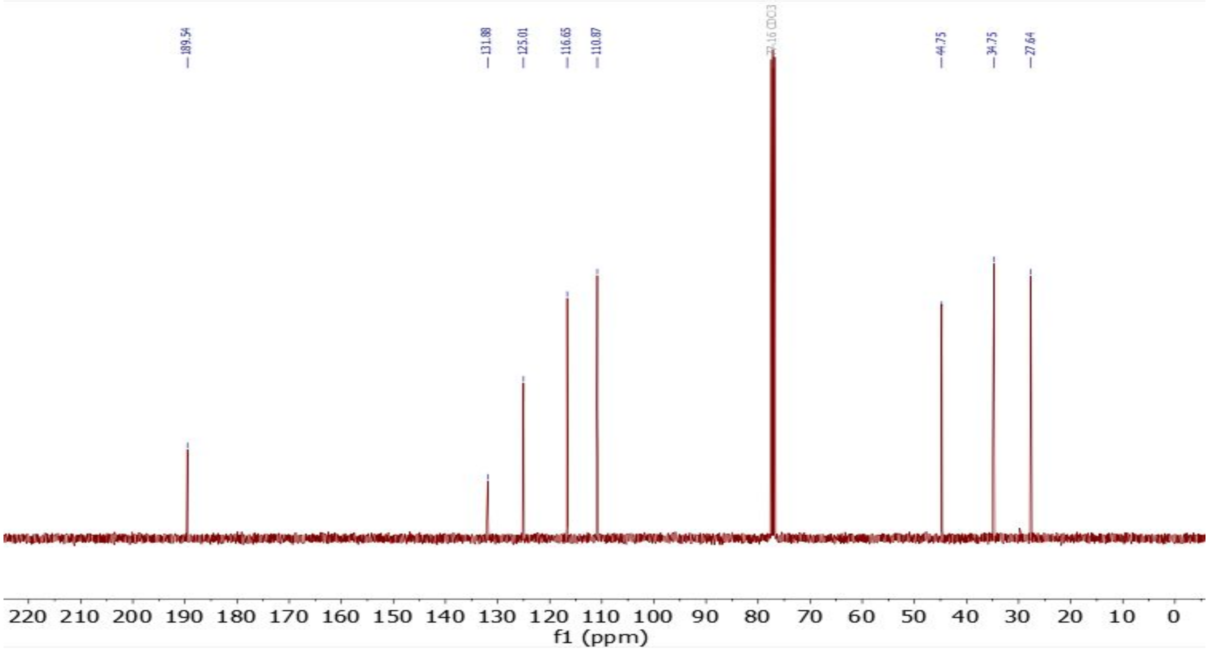

Fig S93: $^{13}C\{^1H\}$ NMR (101 MHz) spectrum 4-chloro-1-(1*H*-pyrrol-2-yl)butan-1-one (**22**) in $CDCl_3$

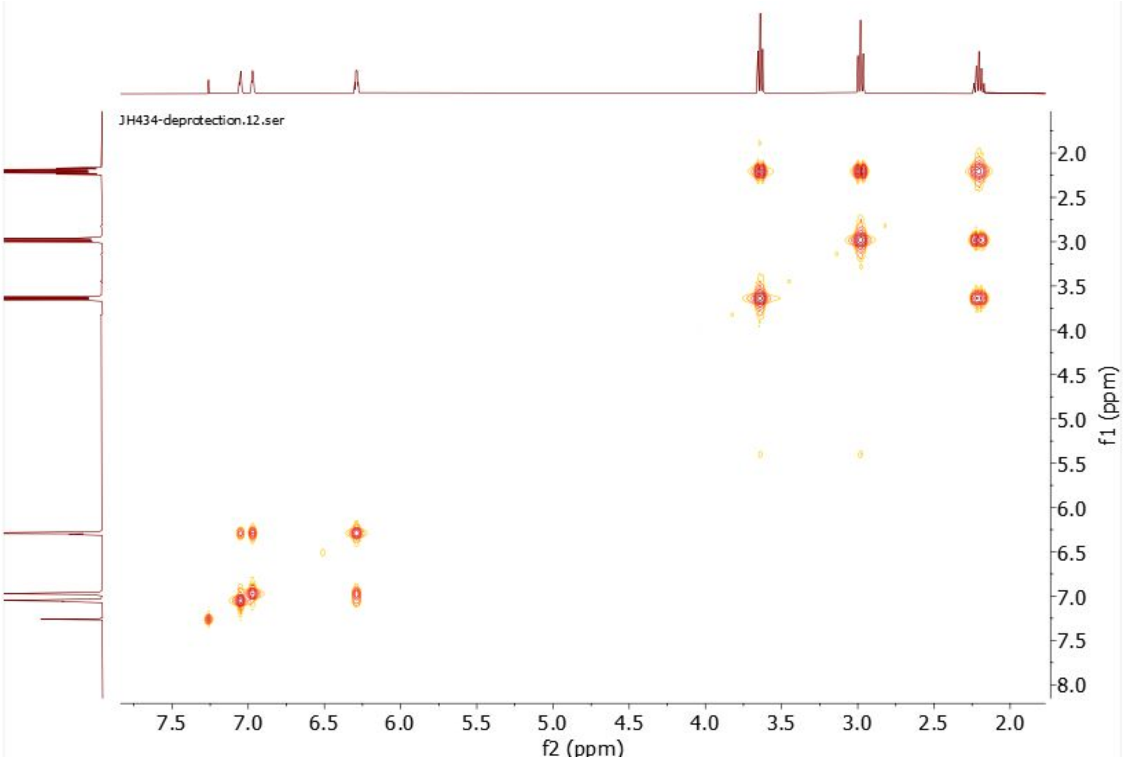

Fig S94: COSY NMR spectrum 4-chloro-1-(1*H*-pyrrol-2-yl)butan-1-one (**22**) in $CDCl_3$

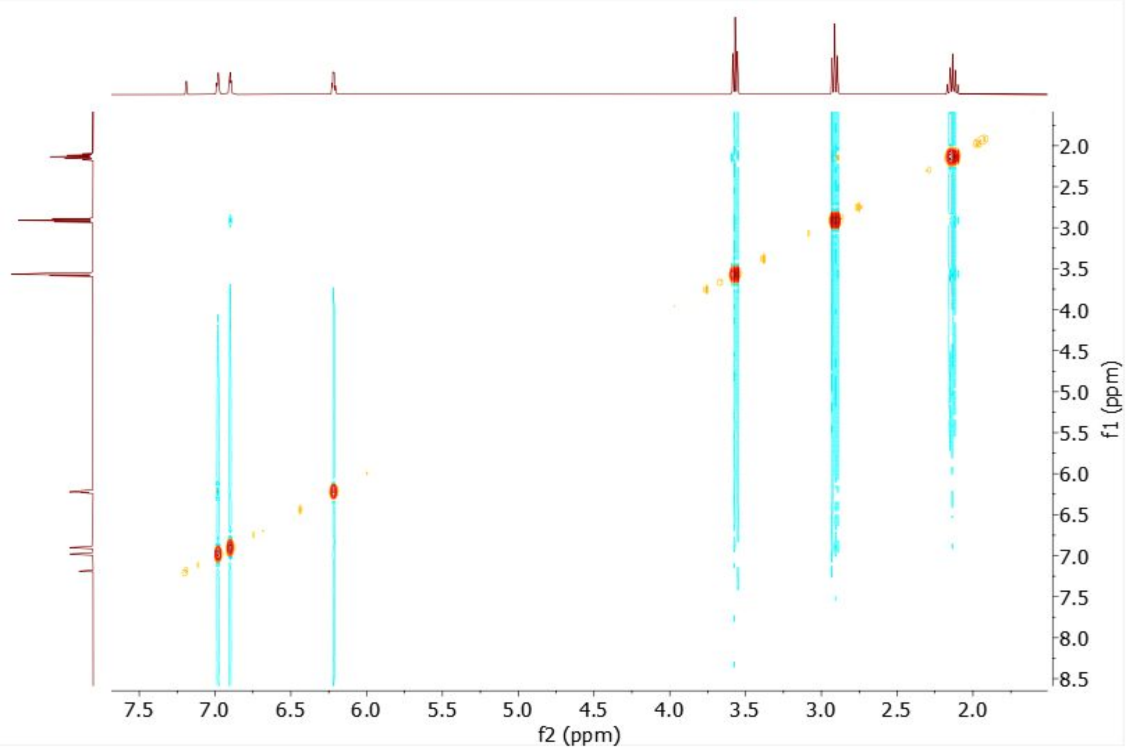

Fig S95: NOESY NMR spectrum 4-chloro-1-(1*H*-pyrrol-2-yl)butan-1-one (**22**) in $CDCl_3$

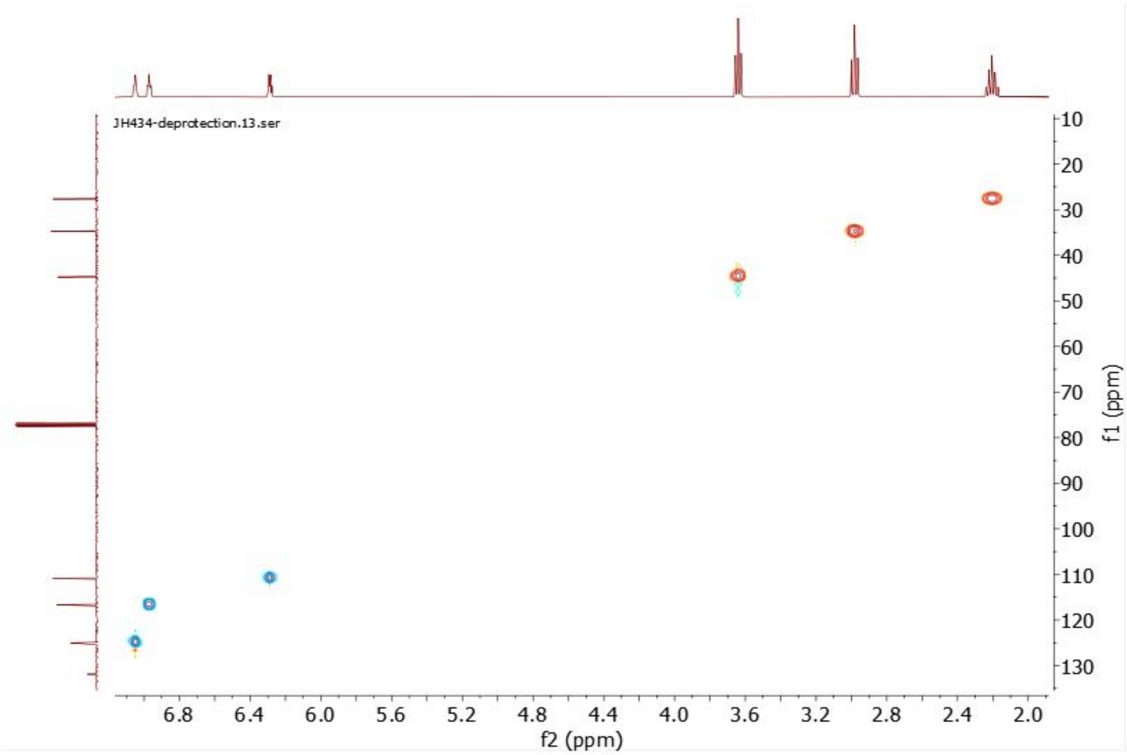

Fig S96: HSQC NMR spectrum 4-chloro-1-(1*H*-pyrrol-2-yl)butan-1-one (**22**) in $CDCl_3$

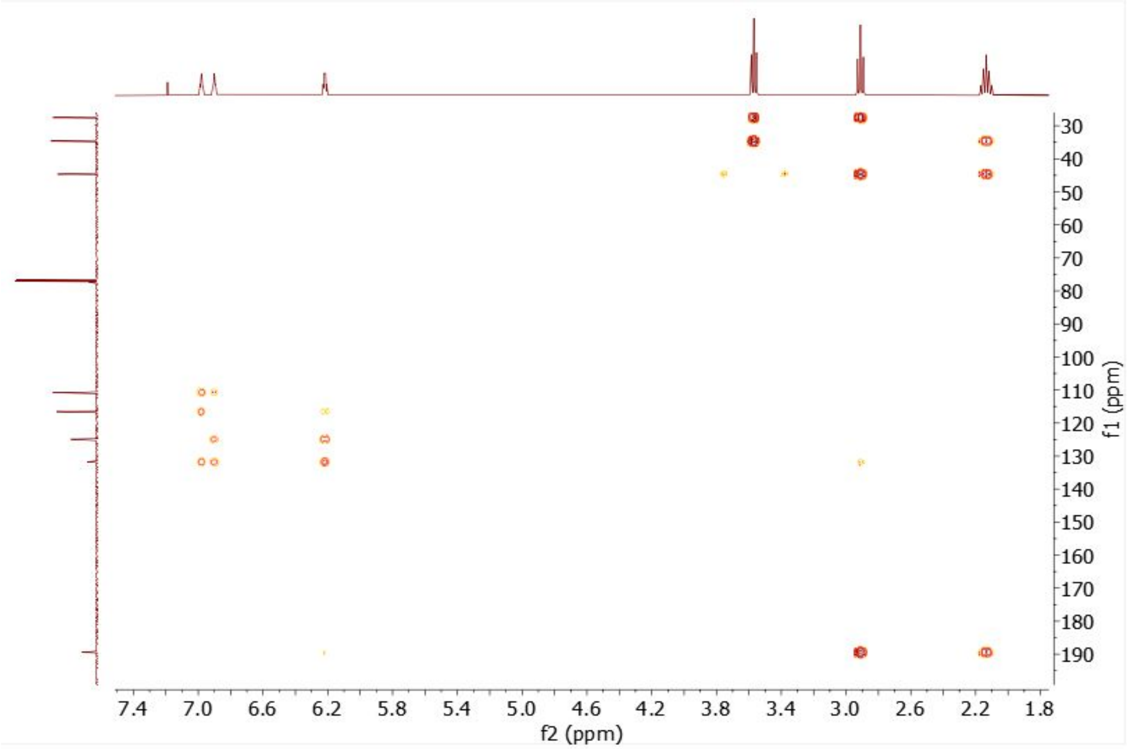

Fig S97: HMBC NMR spectrum 4-chloro-1-(1*H*-pyrrol-2-yl)butan-1-one in $CDCl_3$

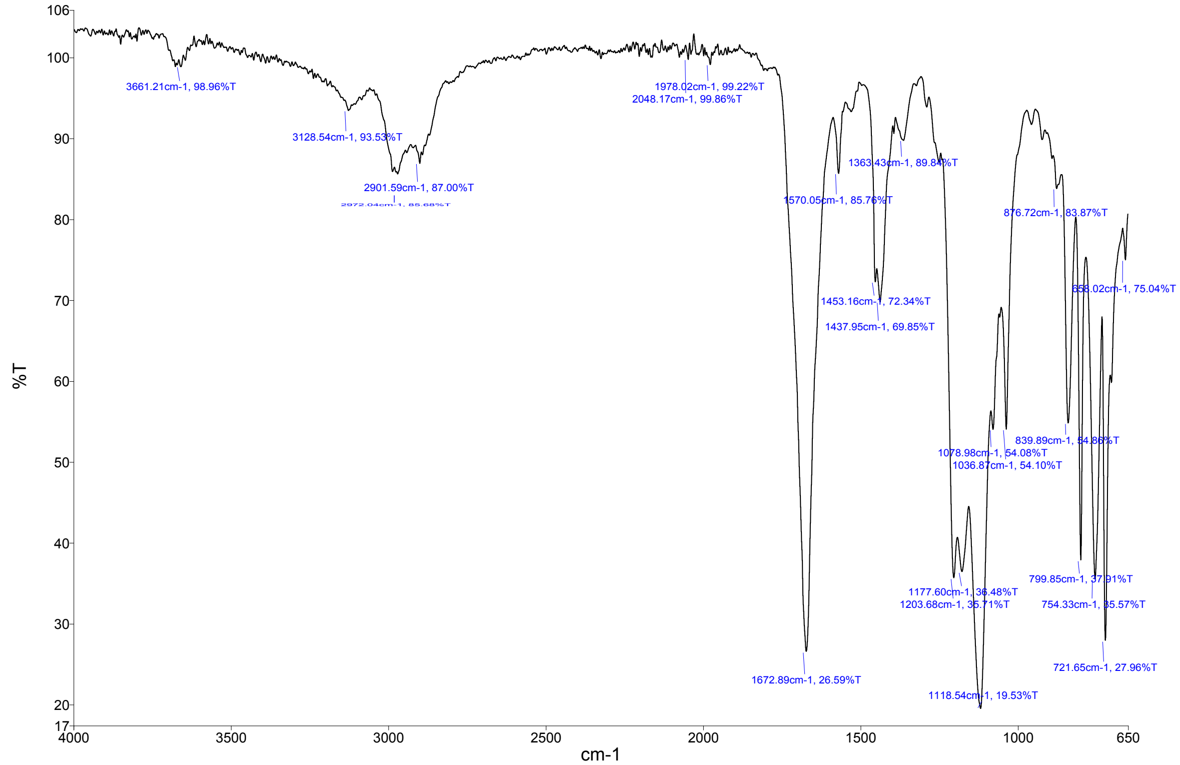

Fig S98: IR spectrum (neat) of 4-chloro-1-(1*H*-pyrrol-2-yl)butan-1-one (**22)**

2,2,2-Trichloroethyl 2-(adamantane-1-carbonyl)-1*H*-pyrrole-1-carboxylate (23)

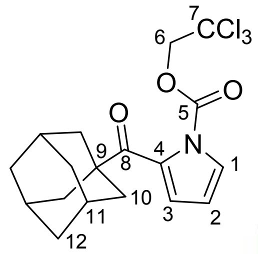

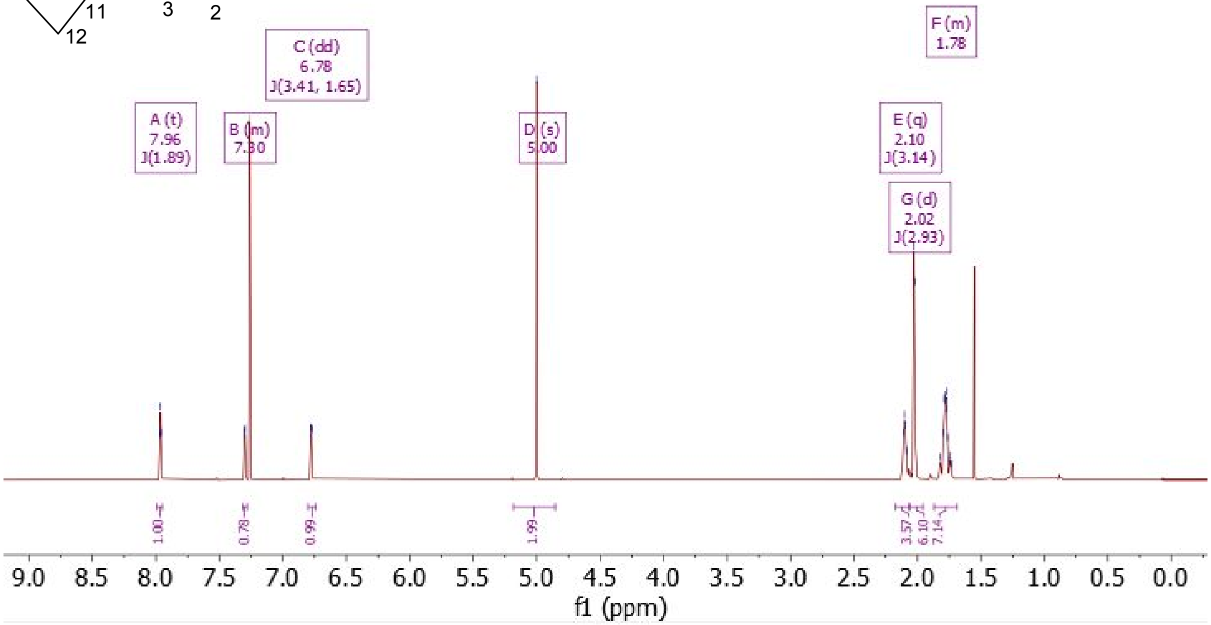

Fig S99: ^{1}H NMR (500 MHz) spectrum of 2,2,2-trichloroethyl 2-(adamantane-1-carbonyl)-1H-pyrrole-1-carboxylate (**23**) in $CDCl_3$

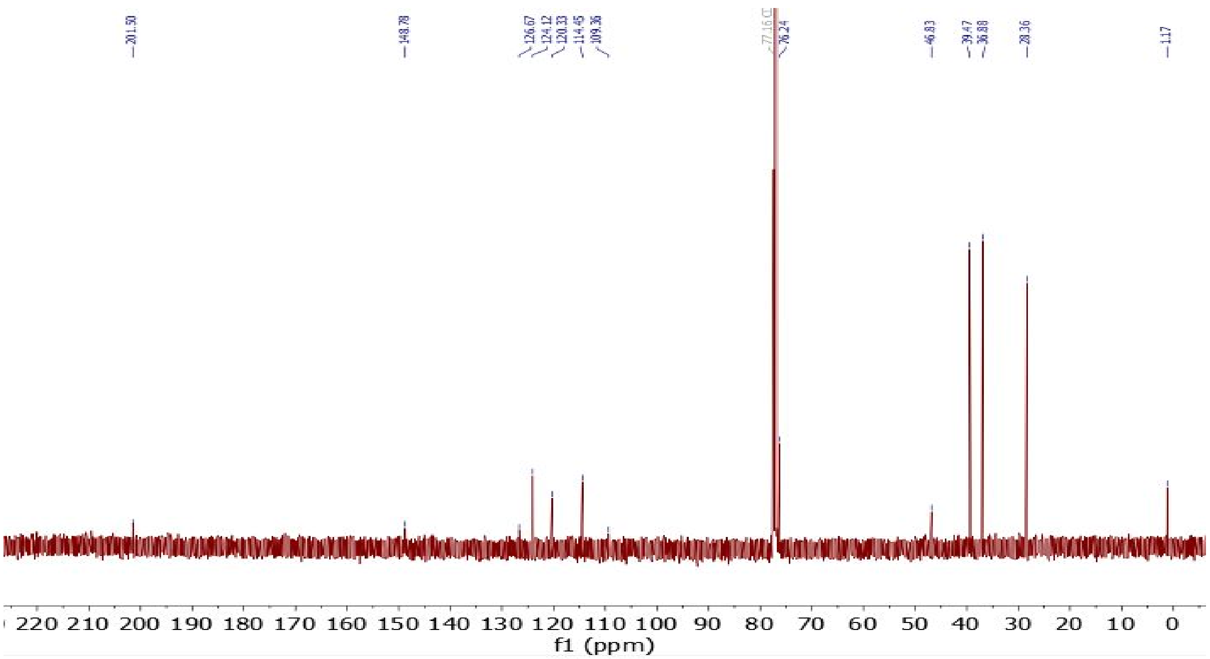

Fig S100: $^{13}C\{^{1}H\}$ NMR (126 MHz) spectrum of 2,2,2-trichloroethyl 2-(adamantane-1-carbonyl)-1H-pyrrole-1-carboxylate (**23**) in $CDCl_3$

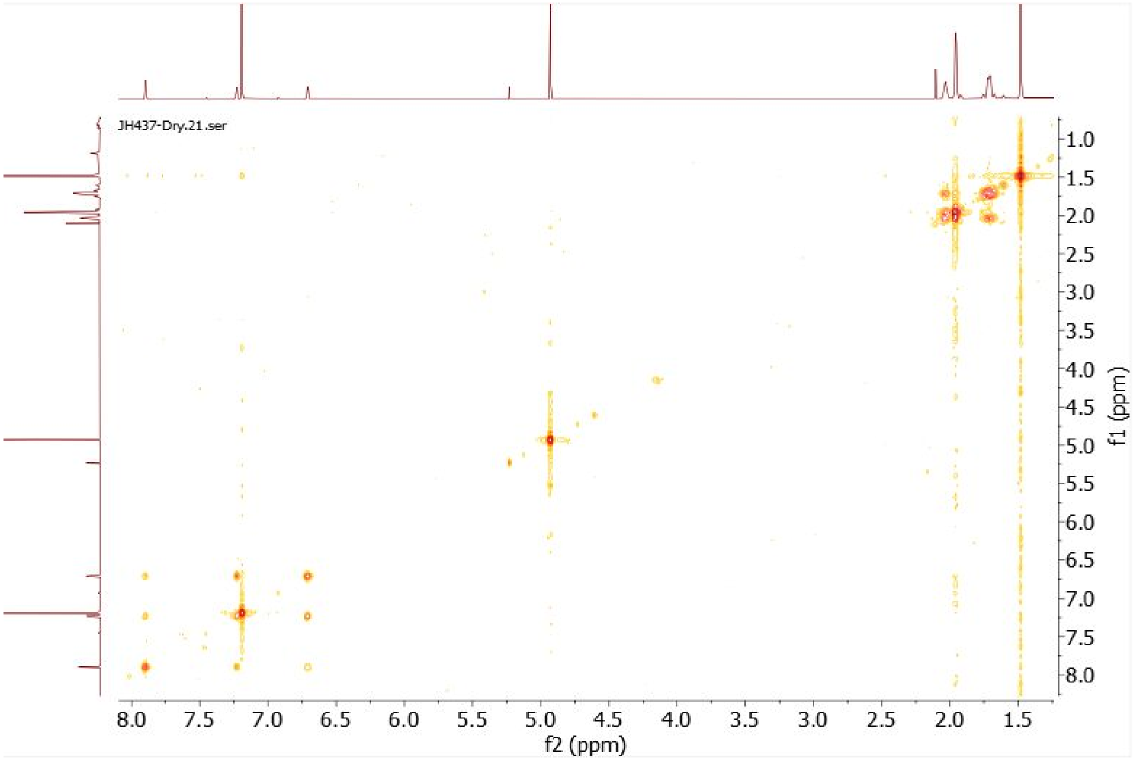

Fig S101: COSY NMR spectrum of 2,2,2-trichloroethyl 2-(adamantane-1-carbonyl)-1H-pyrrole-1-carboxylate (**23**) in $CDCl_3$

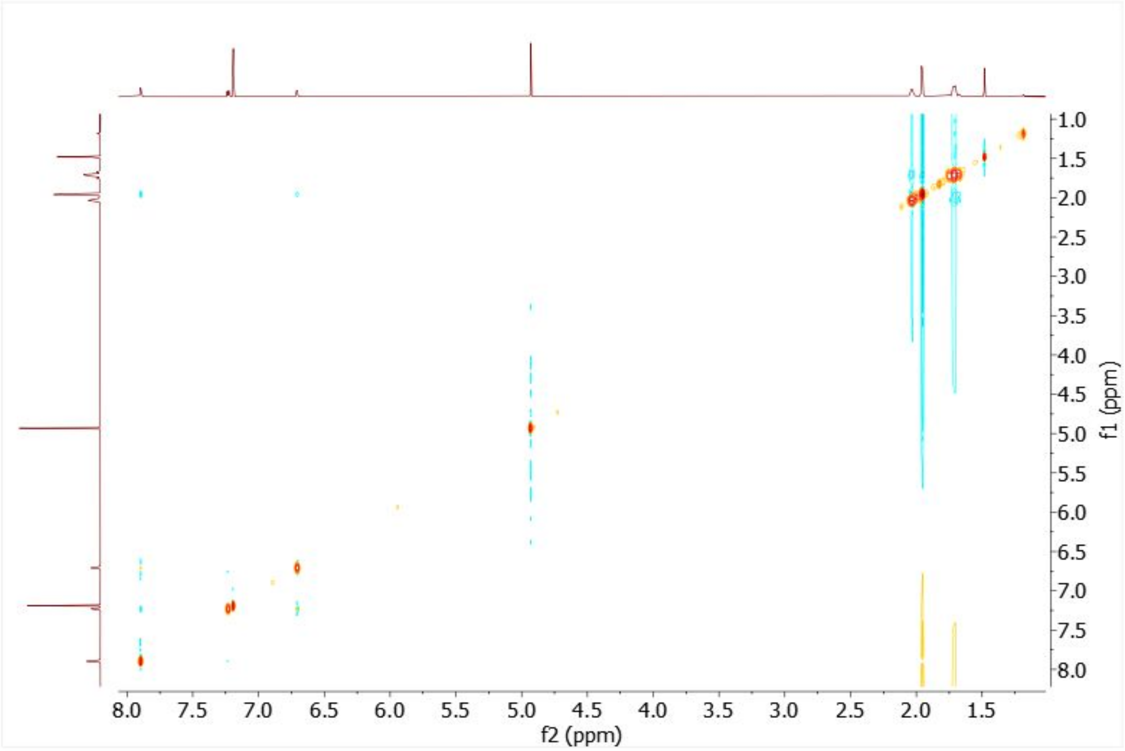

Fig S102: NOESY of 2,2,2-trichloroethyl 2-(adamantane-1-carbonyl)-1H-pyrrole-1-carboxylate (**23**)

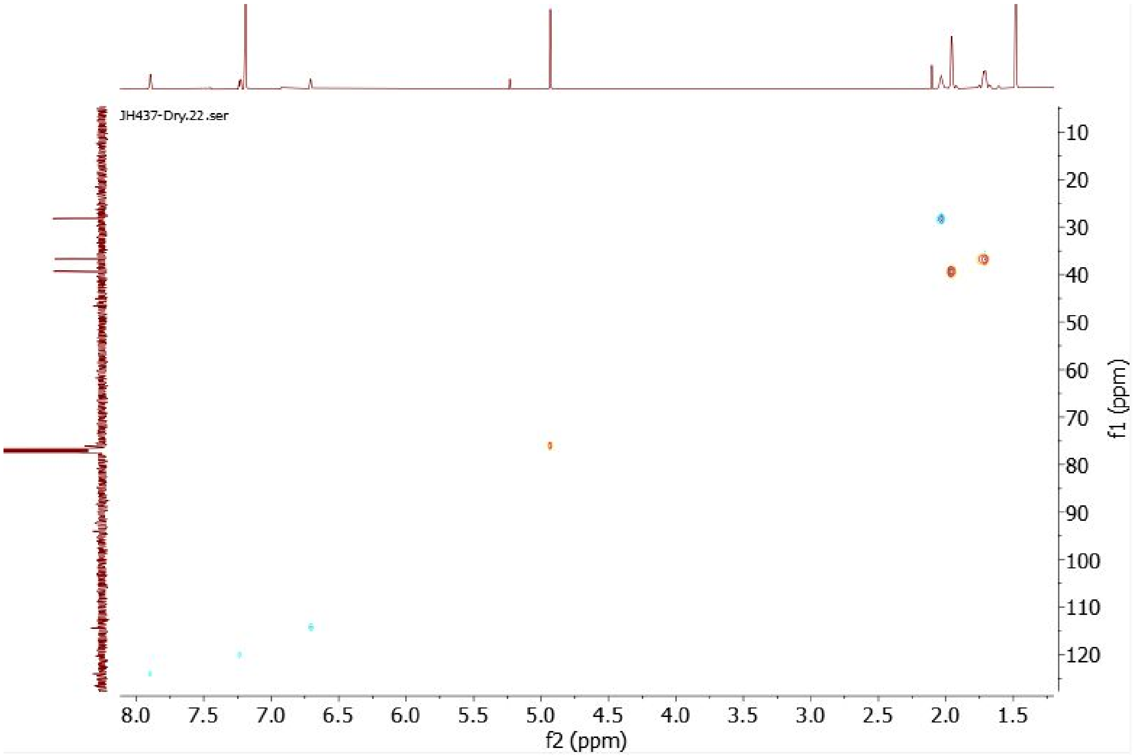

Fig S103: HSQC NMR spectrum of 2,2,2-trichloroethyl 2-(adamantane-1-carbonyl)-1H-pyrrole-1-carboxylate (**23**) in $CDCl_3$

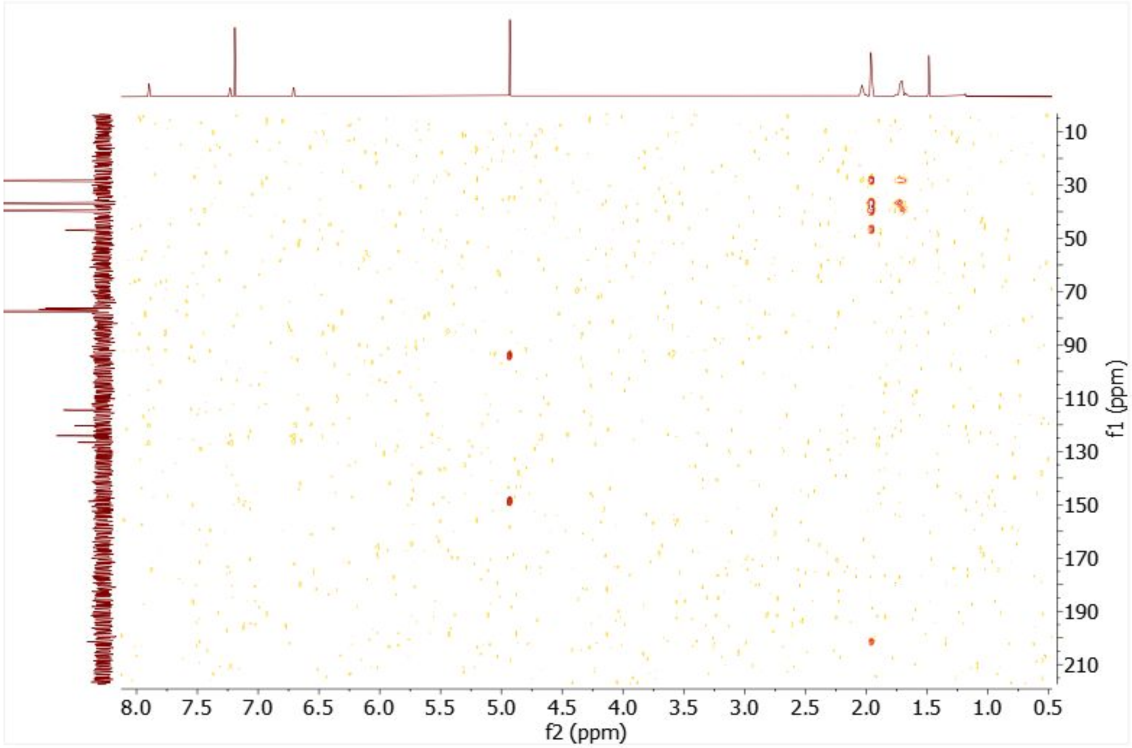

Fig S104: HMBC of 2,2,2-trichloroethyl 2-(adamantane-1-carbonyl)-1H-pyrrole-1-carboxylate (**23**) in $CDCl_3$

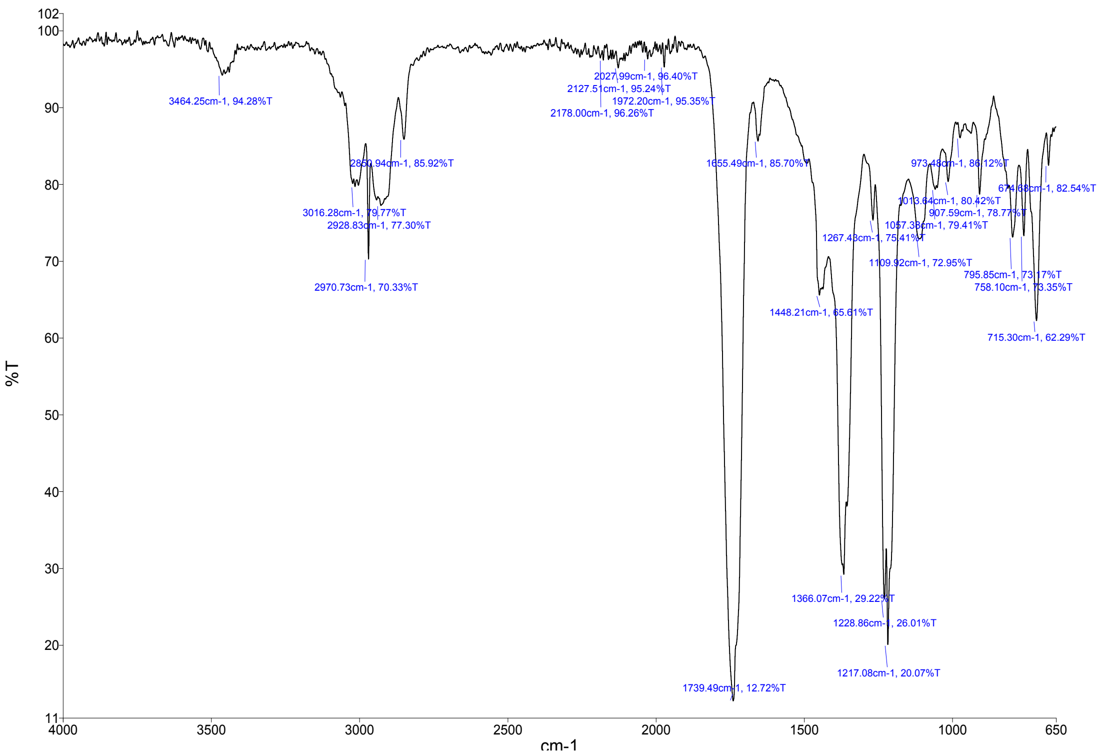

Fig S105: IR spectrum of 2,2,2-trichloroethyl 2-(adamantane-1-carbonyl)-1H-pyrrole-1-carboxylate (**23**) in $CDCl_3$

(Adamantan-1-yl)(1*H*-pyrrol-2-yl)methanone (24)

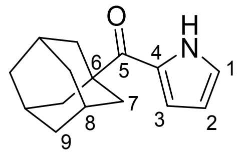

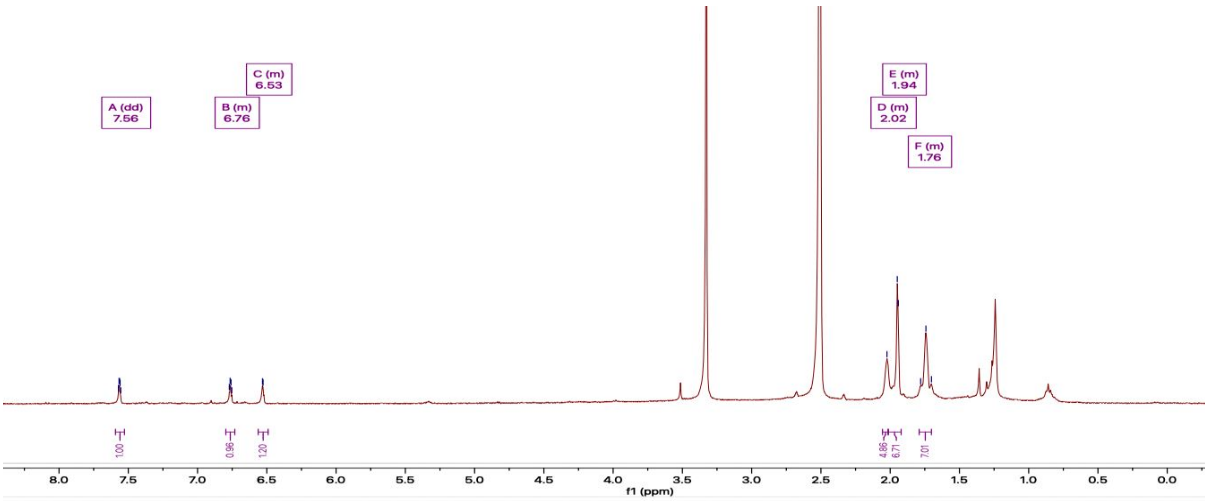

Fig S106: ^{1}H NMR (400 MHz) spectrum of (adamantan-1-yl)(1*H*-pyrrol-2-yl)methanone (**24**) in DMSO-d_6

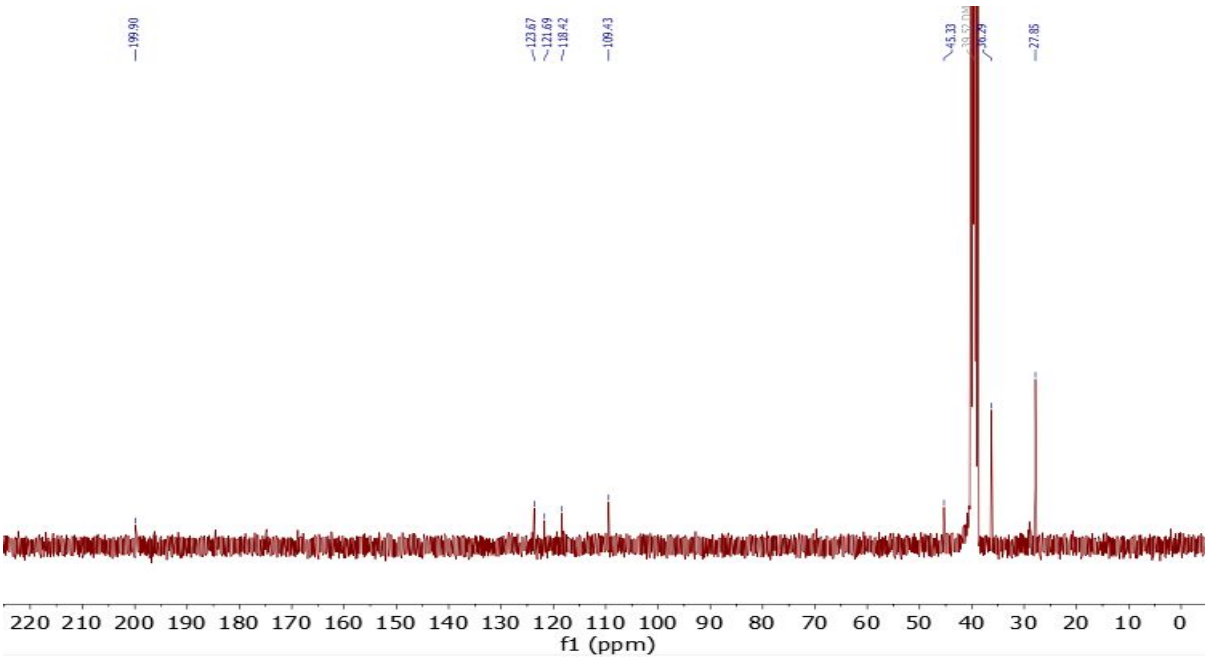

Fig S107: ^{13}C{^{1}H} NMR (101 MHz) spectrum of (adamantan-1-yl)(1*H*-pyrrol-2-yl)methanone (**24**) in DMSO-d_6

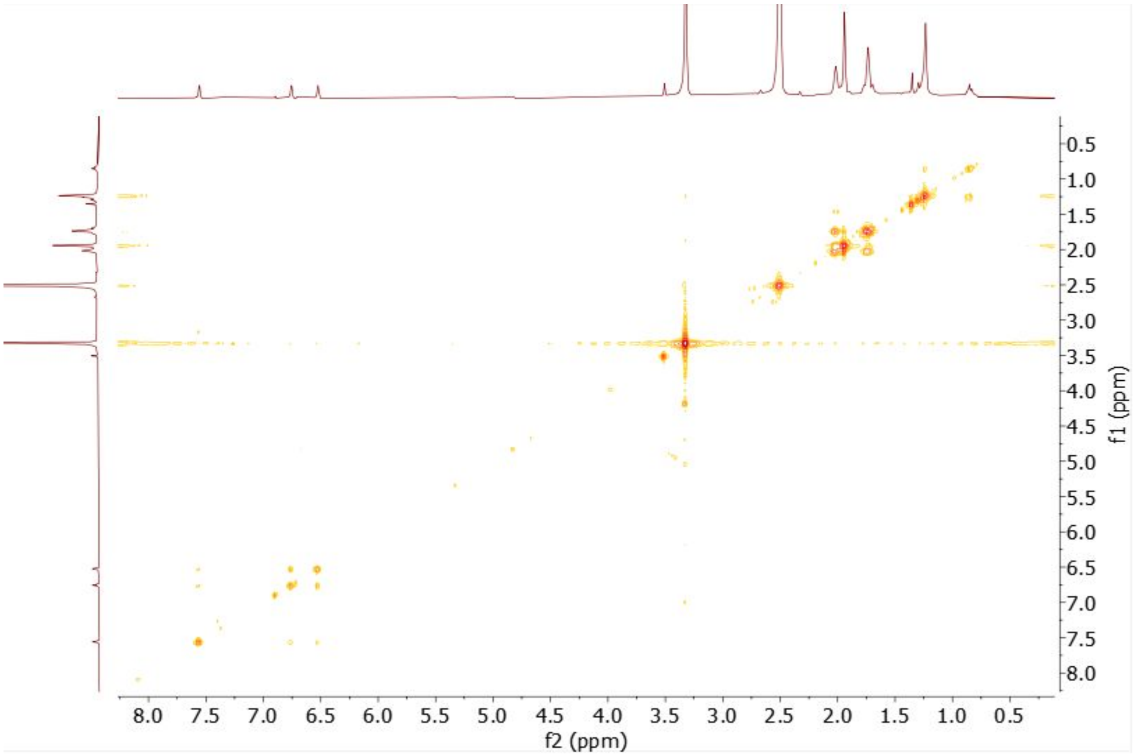

Fig S108: COSY NMR spectrum of (adamantan-1-yl)(1*H*-pyrrol-2-yl)methanone (**24**) in DMSO-d_6

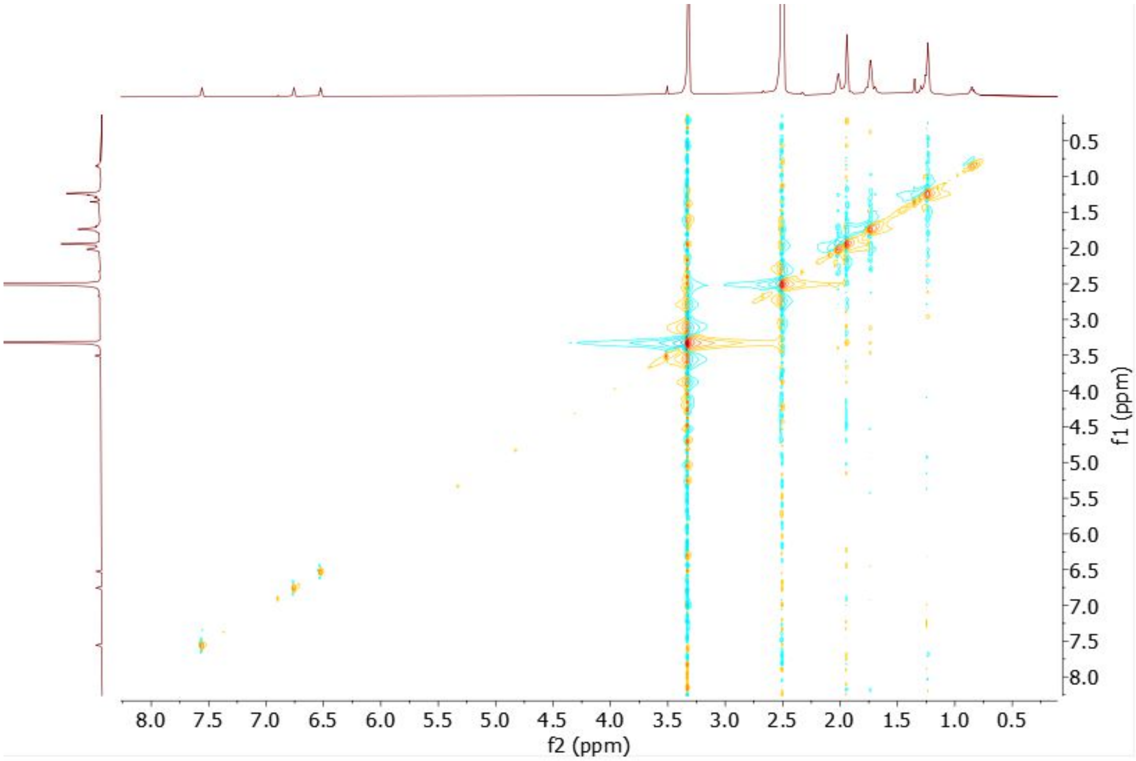

Fig S109: NOESY NMR spectrum of (adamantan-1-yl)(1*H*-pyrrol-2-yl)methanone (**24**) in DMSO-d_6

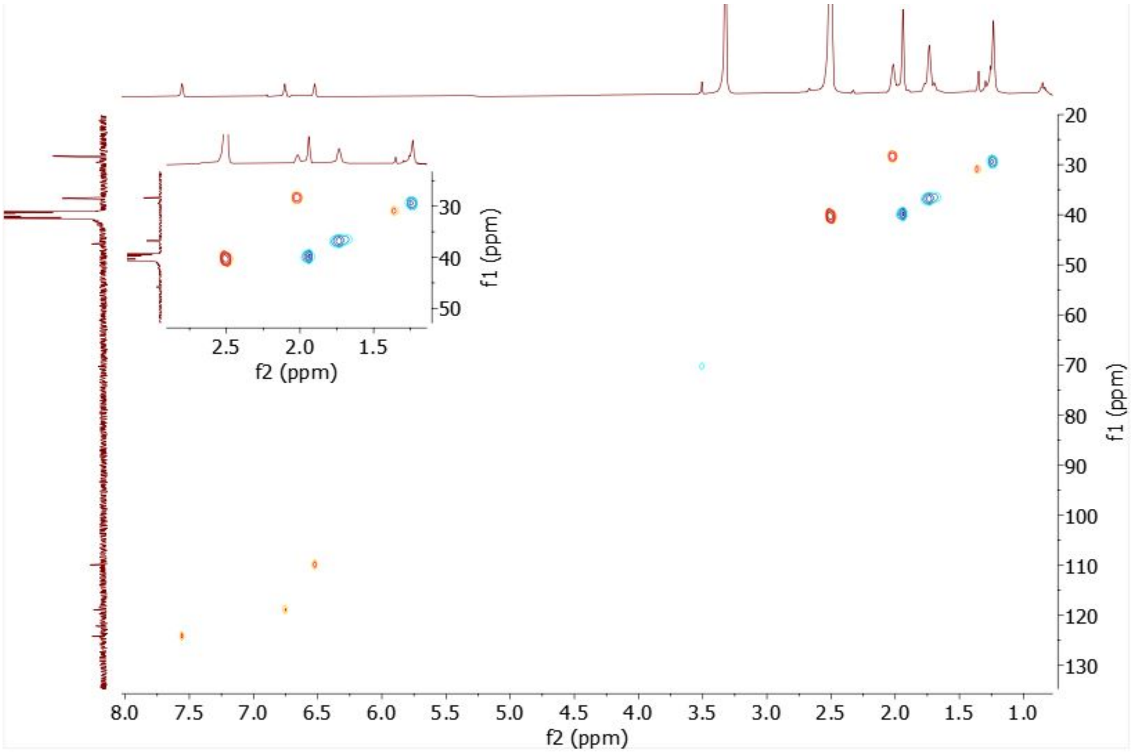

Fig S110: HSQC NMR spectrum of (adamantan-1-yl)(1*H*-pyrrol-2-yl)methanone (**24**) in DMSO-d_6

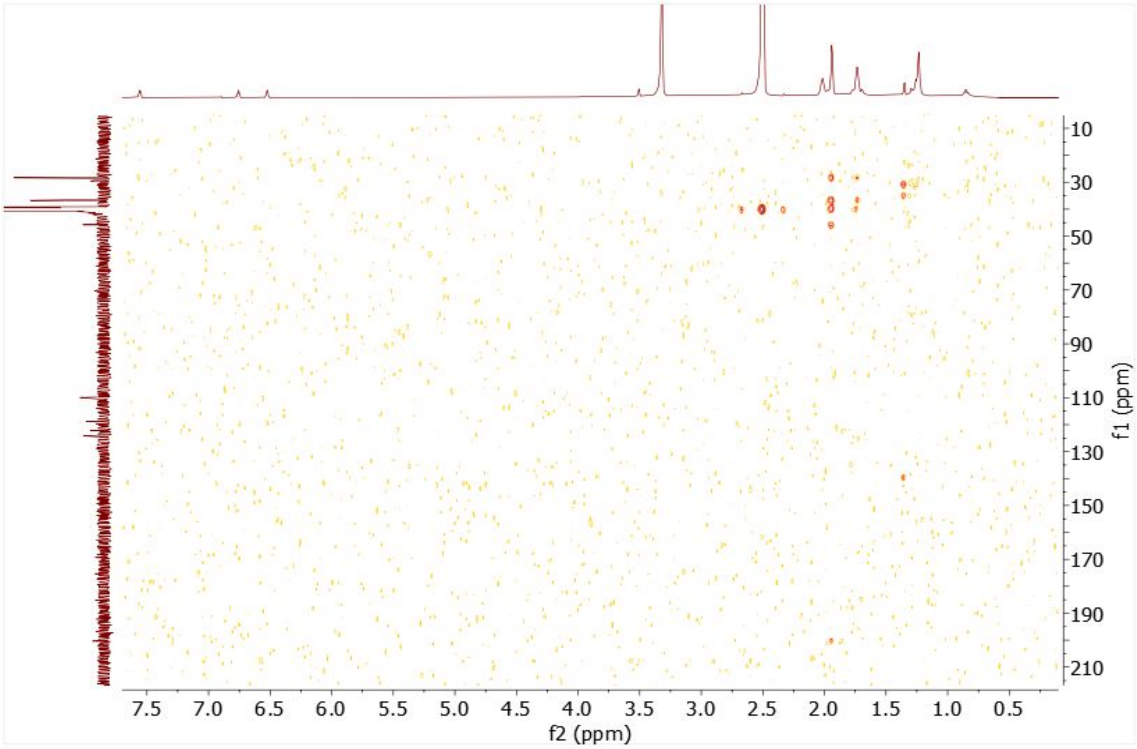

Fig S111: HMBC NMR spectrum of (adamantan-1-yl)(1*H*-pyrrol-2-yl)methanone (**24**) in DMSO-d_6

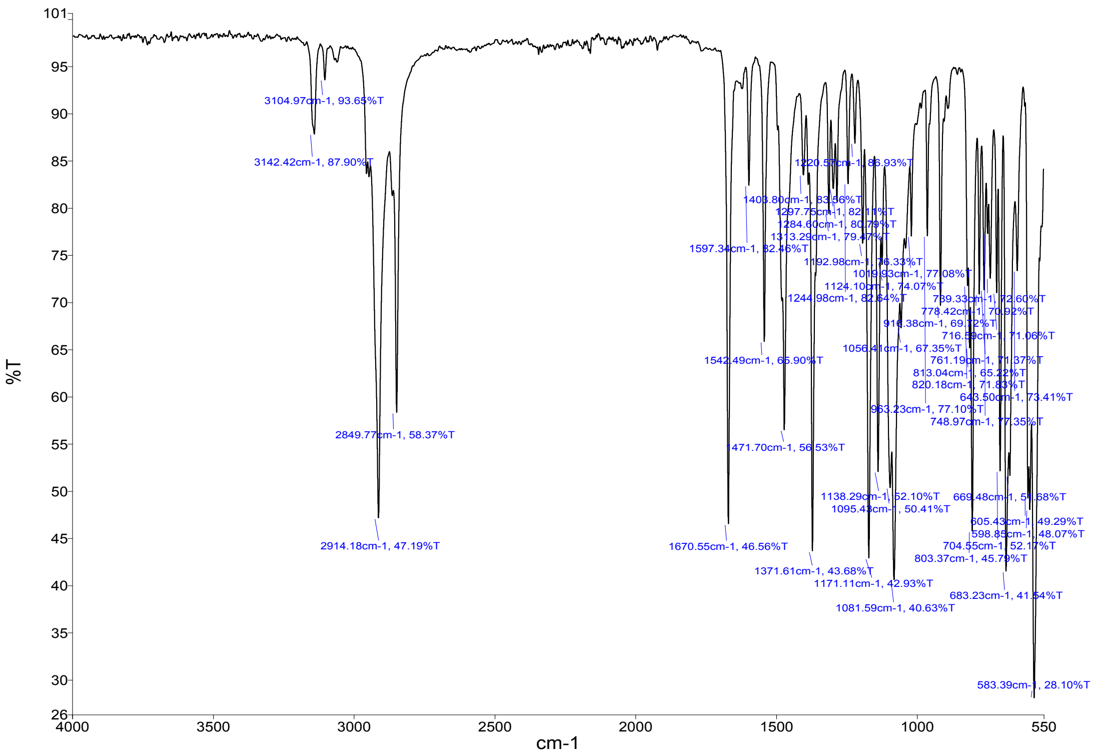

Fig S112: IR (neat) spectrum of (adamantan-1-yl)(1*H*-pyrrol-2-yl)methanone **(24)**

2,2,2-Trichloroethyl 2-(2,2-diphenylacetyl)-1*H*-pyrrole-1-carboxylate (25)

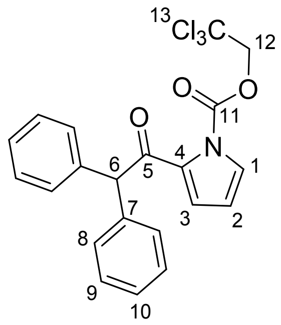

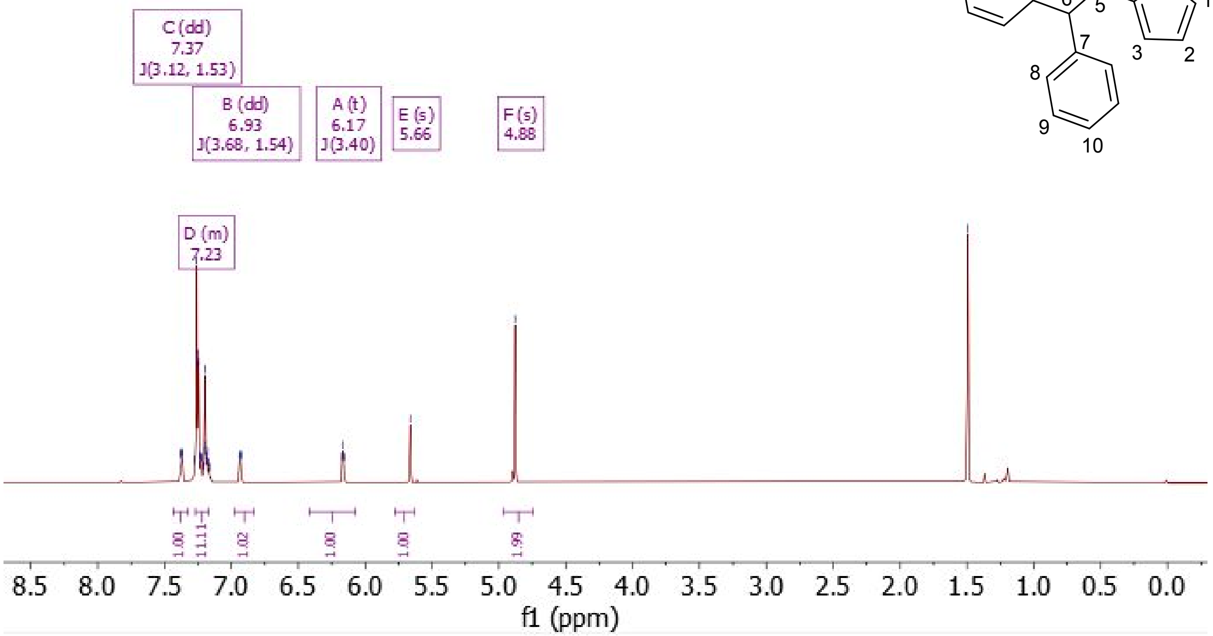

Fig S113: ^{1}H NMR (400 MHz) spectrum of 2,2,2-trichloroethyl 2-(2,2-diphenylacetyl)-1*H*-pyrrole-1-carboxylate (**25**) in $CDCl_3$

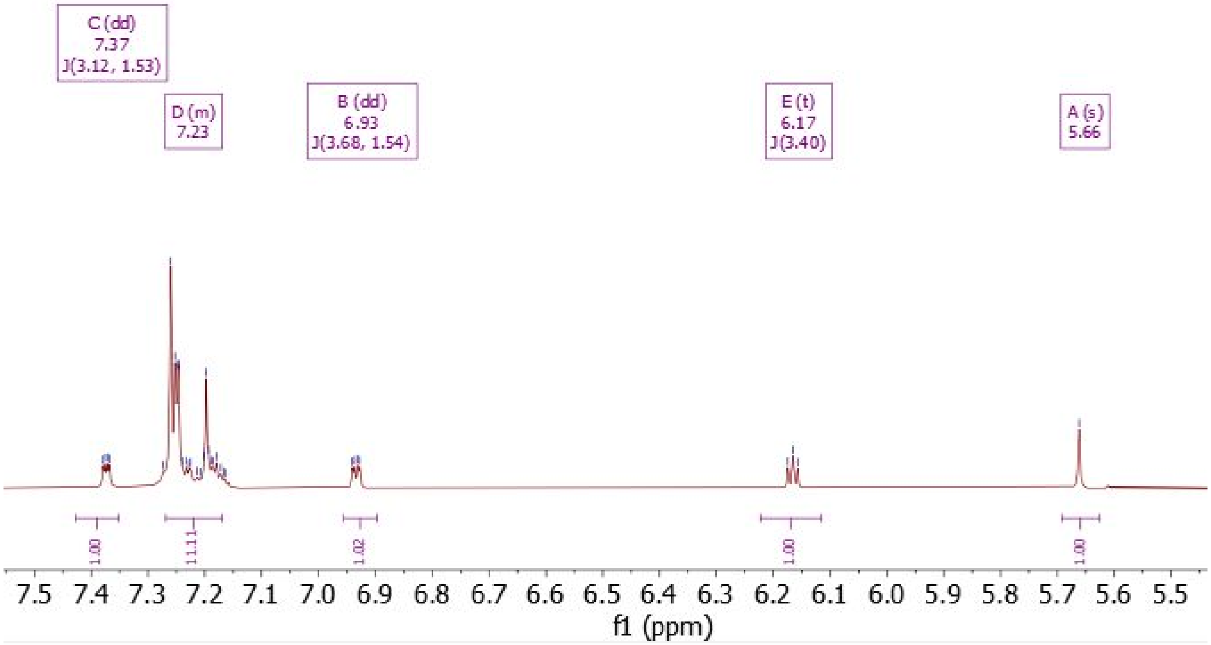

Fig S114: ^{1}H Zoomed NMR spectrum of 2,2,2-trichloroethyl 2-(2,2-diphenylacetyl)-1*H*-pyrrole-1-carboxylate (**25**) in $CDCl_3$

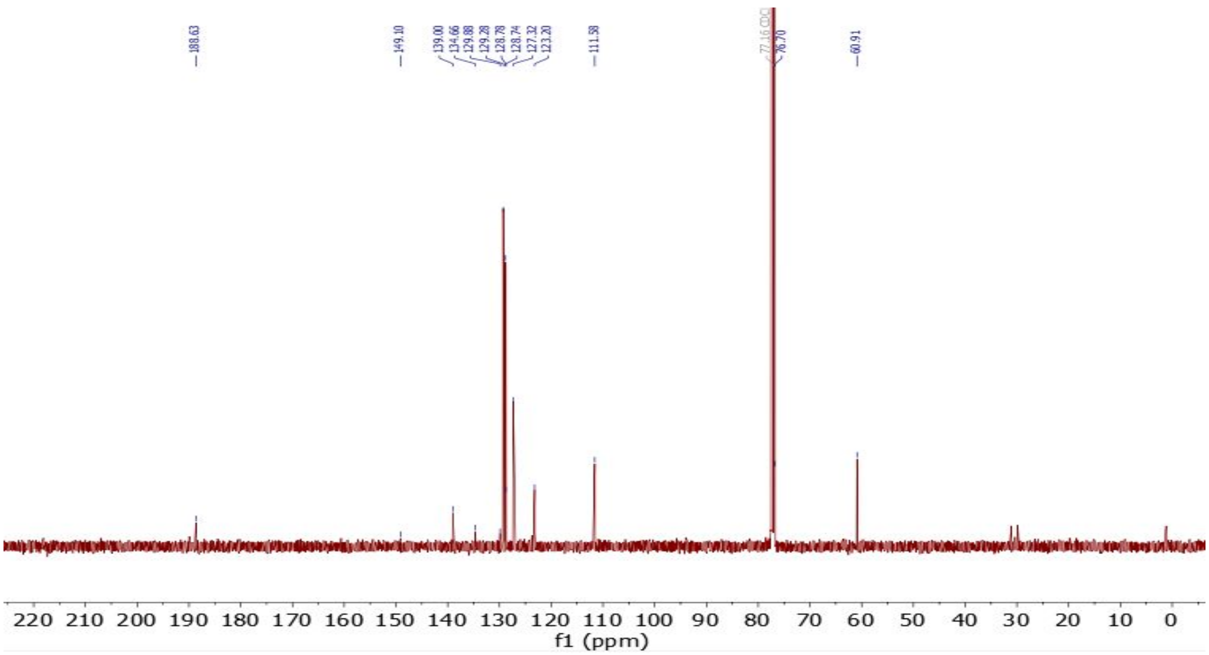

Fig S115: $^{13}C\{^{1}H\}$ NMR (101 MHz) spectrum of 2,2,2-trichloroethyl 2-(2,2-diphenylacetyl)-1*H*-pyrrole-1-carboxylate (**25**)

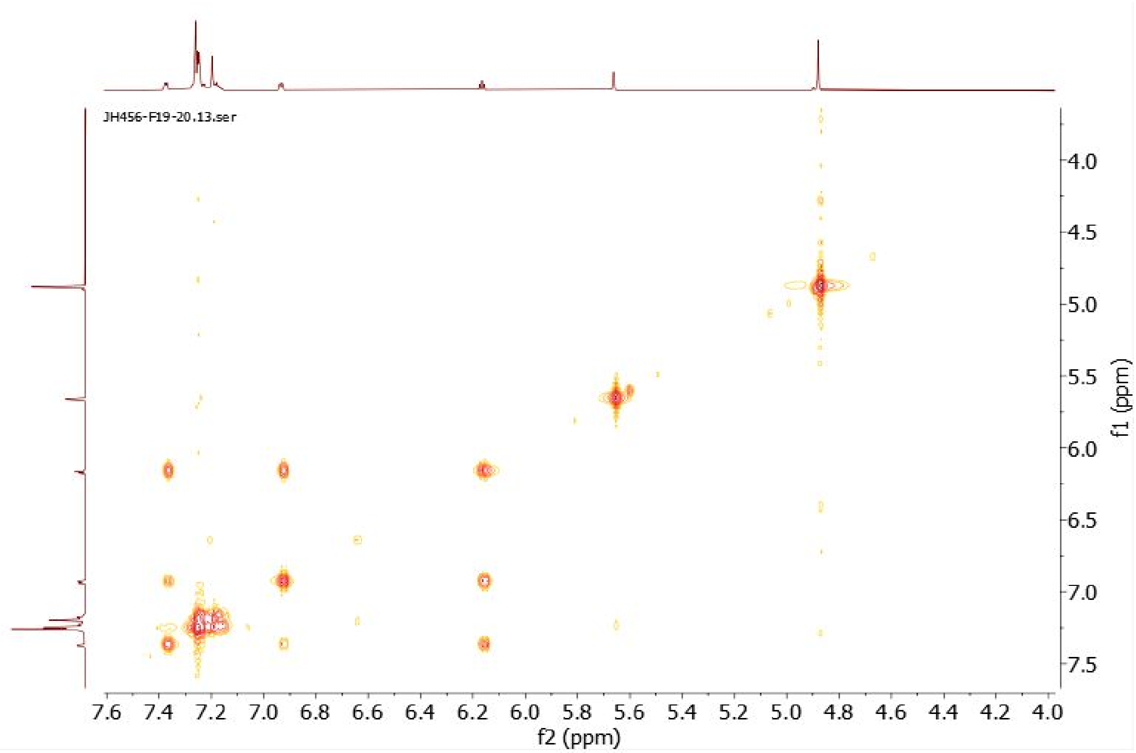

Fig S116: COSY NMR spectrum of 2,2,2-trichloroethyl 2-(2,2-diphenylacetyl)-1*H*-pyrrole-1-carboxylate (**25**) in $CDCl_3$

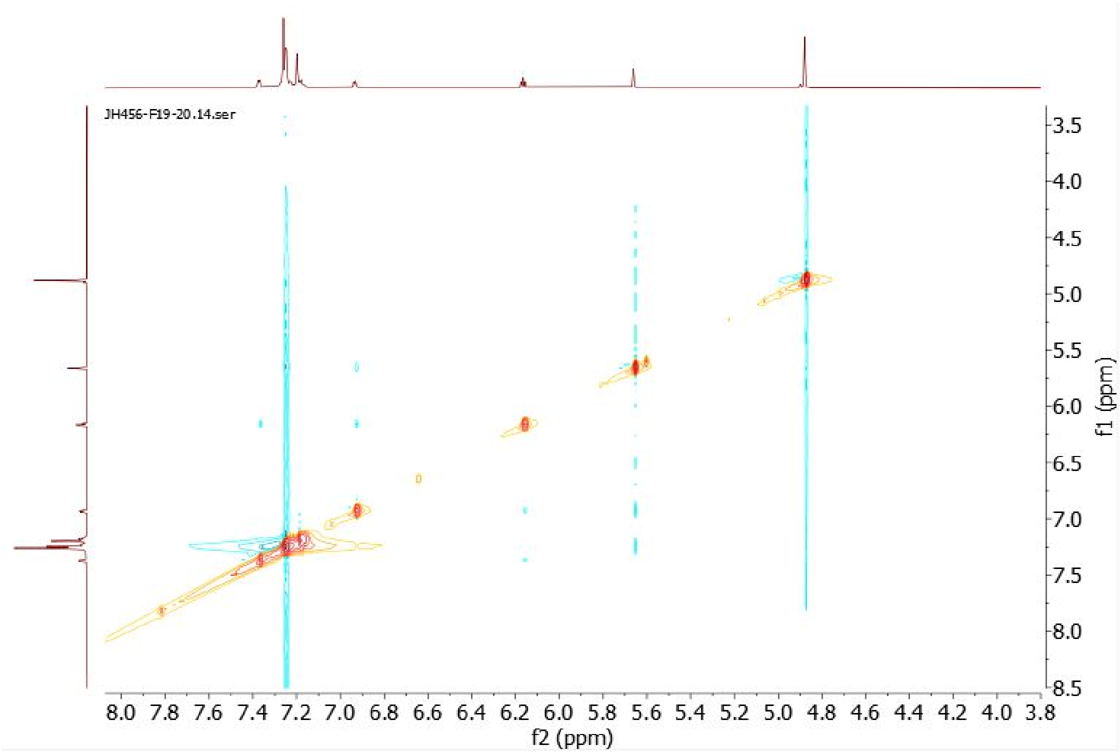

Fig S117: NOESY NMR spectrum of 2,2,2-trichloroethyl 2-(2,2-diphenylacetyl)-1*H*-pyrrole-1-carboxylate (**25**)

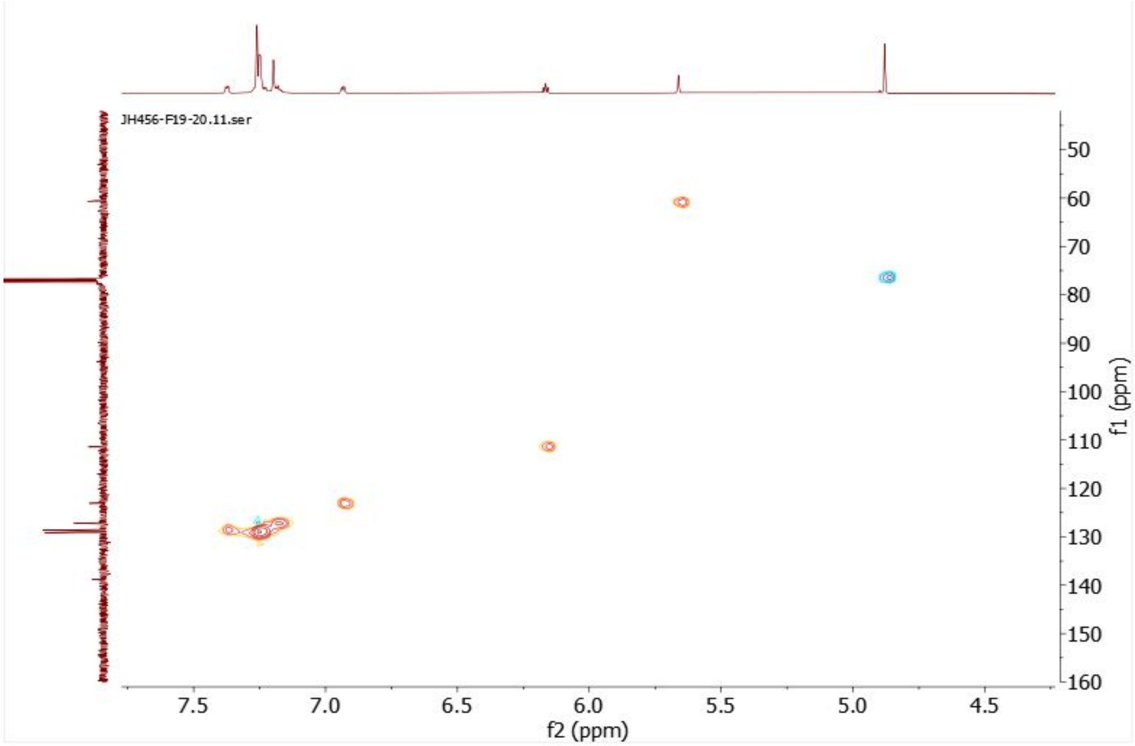

Fig S118: HSQC NMR spectrum of 2,2,2-trichloroethyl 2-(2,2-diphenylacetyl)-1*H*-pyrrole-1-carboxylate (**25**)

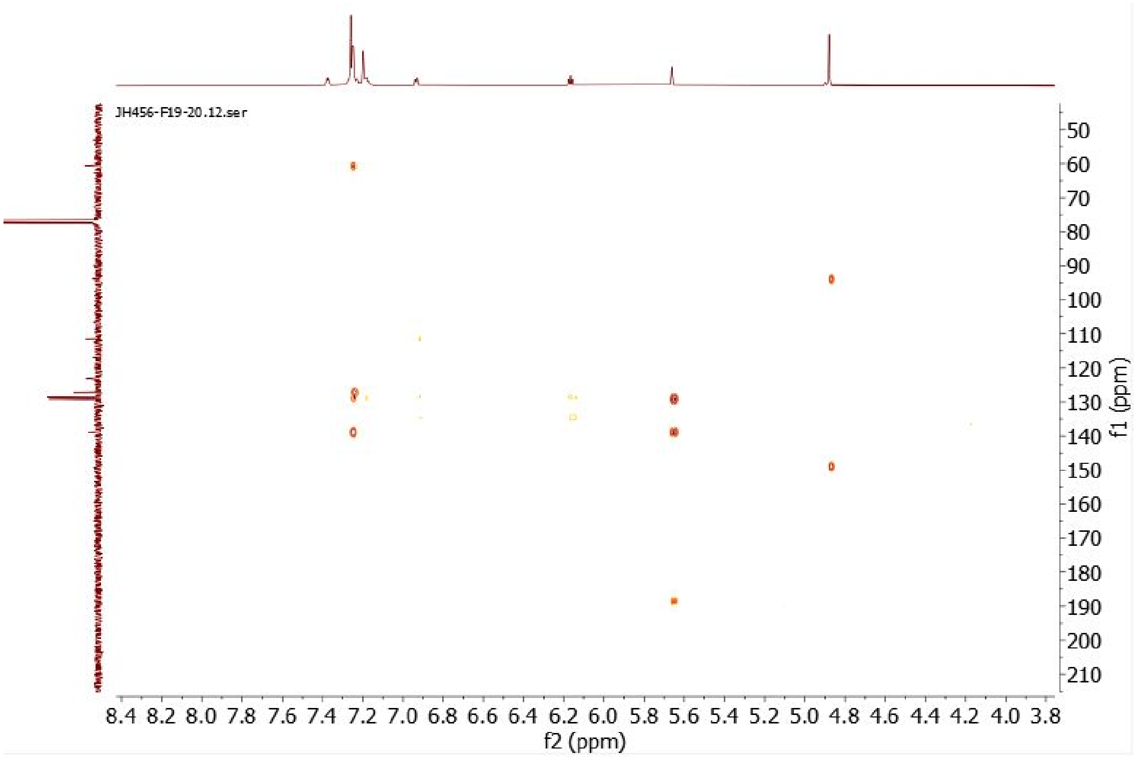

Fig S119: HMBC NMR spectrum of 2,2,2-trichloroethyl 2-(2,2-diphenylacetyl)-1*H*-pyrrole-1-carboxylate (**25**)

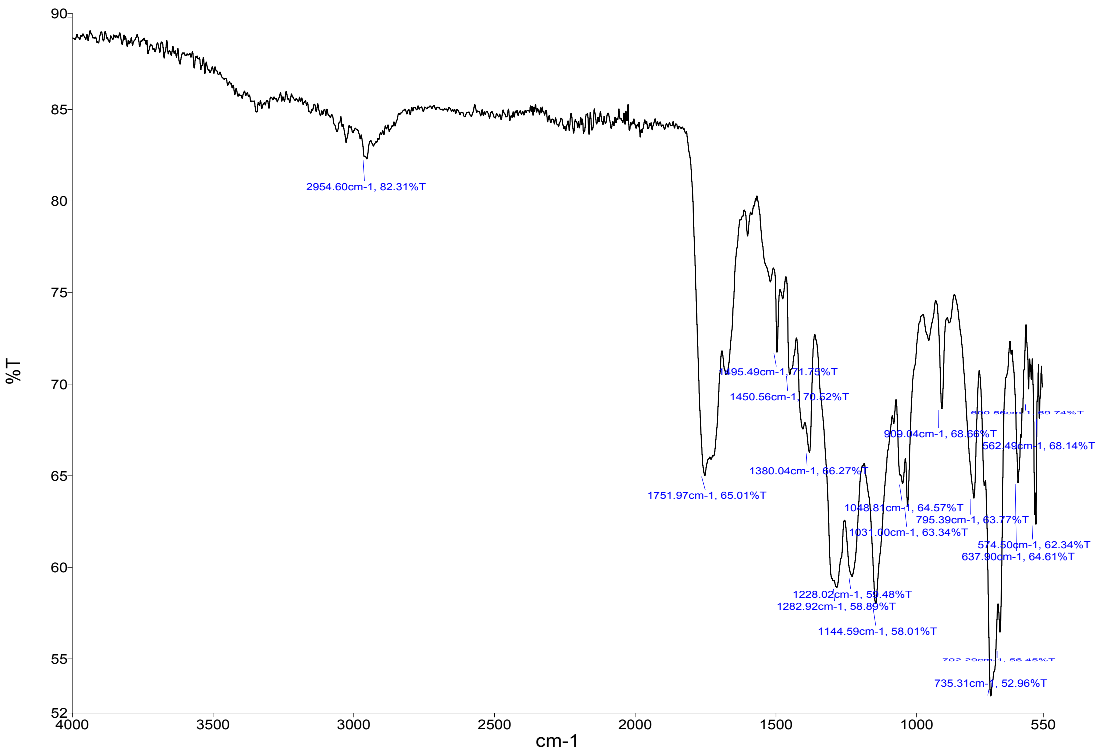

Fig S120: IR spectrum of 2,2,2-trichloroethyl 2-(2,2-diphenylacetyl)-1*H*-pyrrole-1-carboxylate (**25**)

2,2-Diphenyl-1-(1*H*-pyrrol-2-yl)ethan-1-one (26)

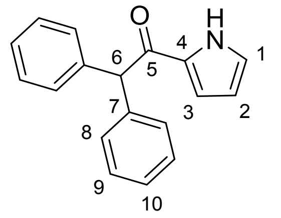

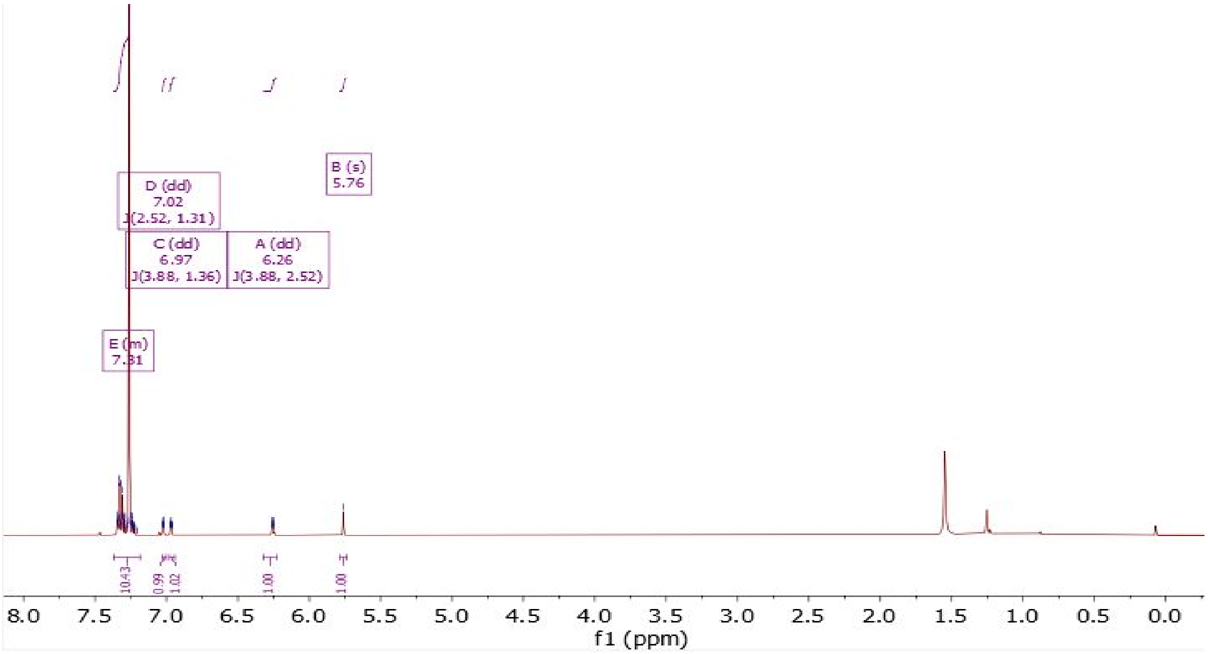

Fig S121: ^{1}H NMR (500 MHz) spectrum of 2,2-diphenyl-1-(1*H*-pyrrol-2-yl)ethan-1-one (**26**) in $CDCl_3$.

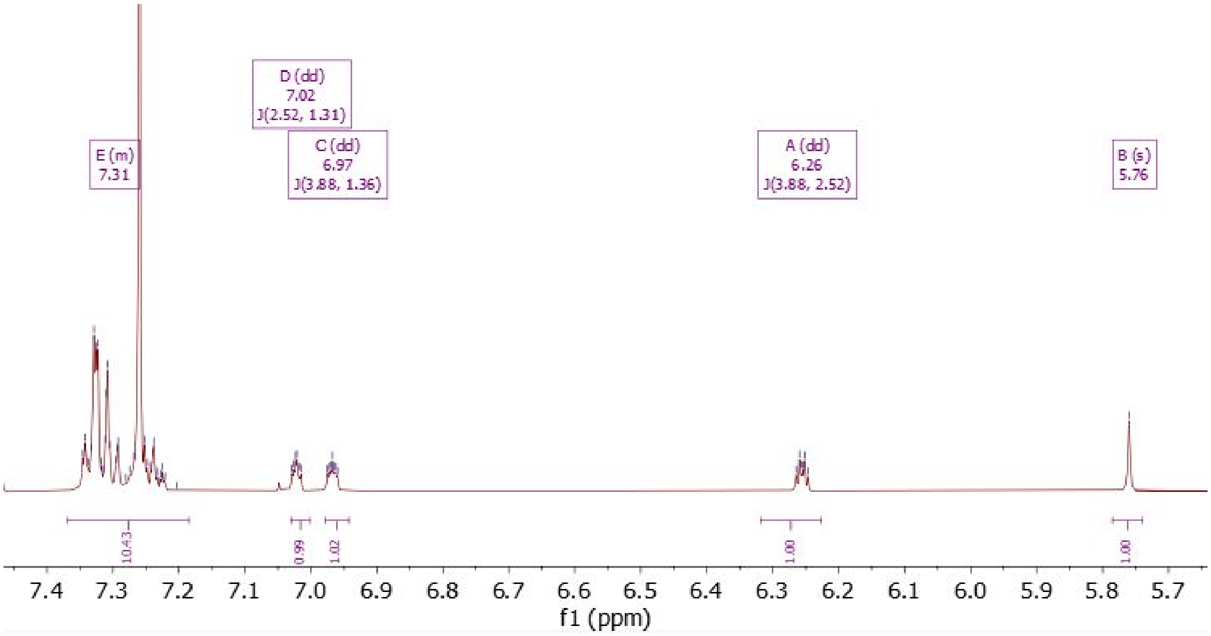

Fig S122: Zoomed ^{1}H NMR (500 MHz) spectrum of 2,2-diphenyl-1-(1*H*-pyrrol-2-yl)ethan-1-one (**26**) in $CDCl_3$

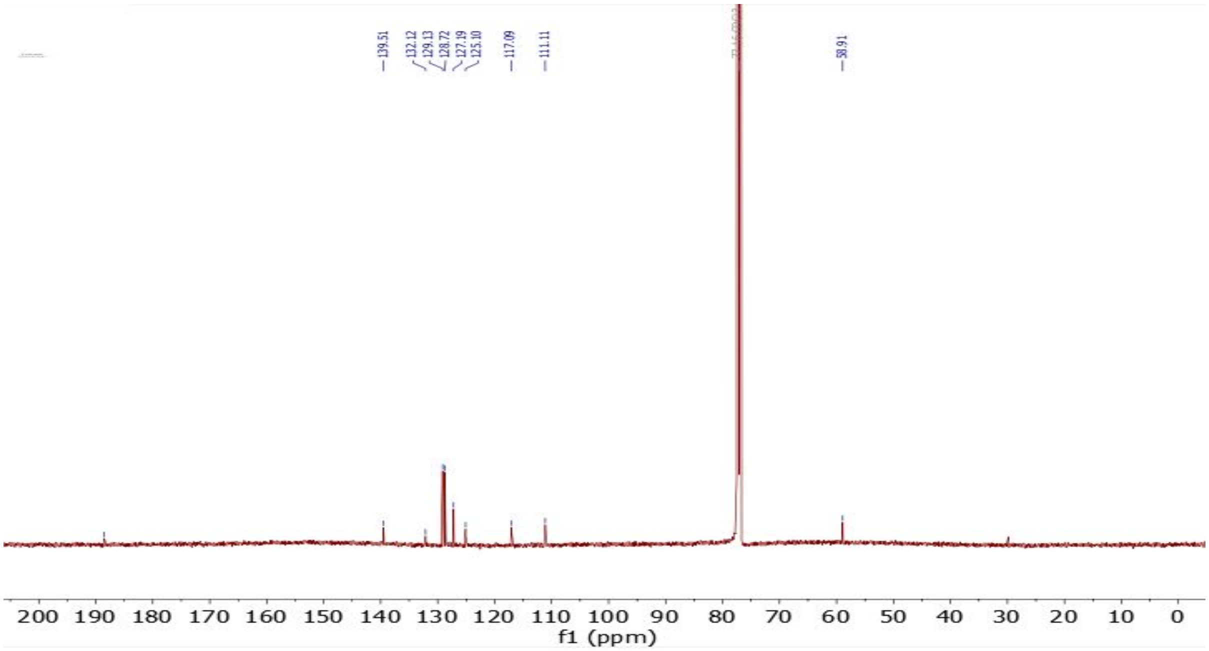

Fig S123: $^{13}C\{^{1}H\}$ NMR (126 MHz) spectrum of 2,2-diphenyl-1-(1*H*-pyrrol-2-yl)ethan-1-one (**26**) in $CDCl_3$.

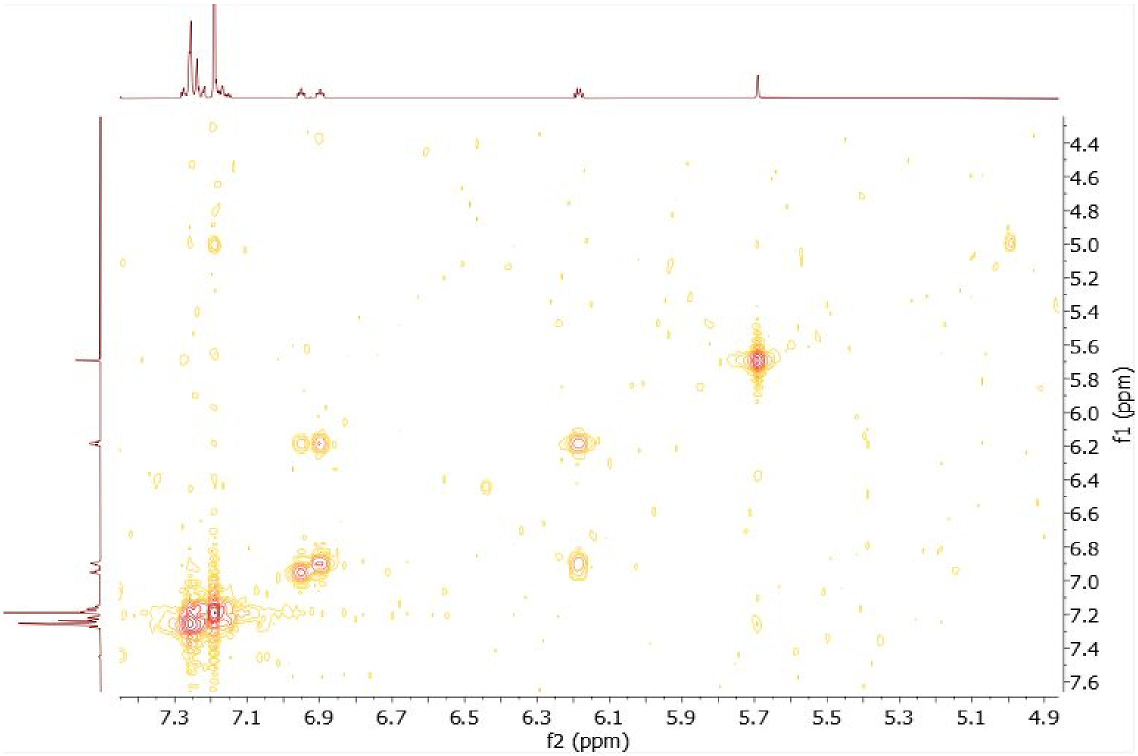

Fig S124: COSY NMR spectrum of 2,2-diphenyl-1-(1*H*-pyrrol-2-yl)ethan-1-one (**26**) in $CDCl_3$.

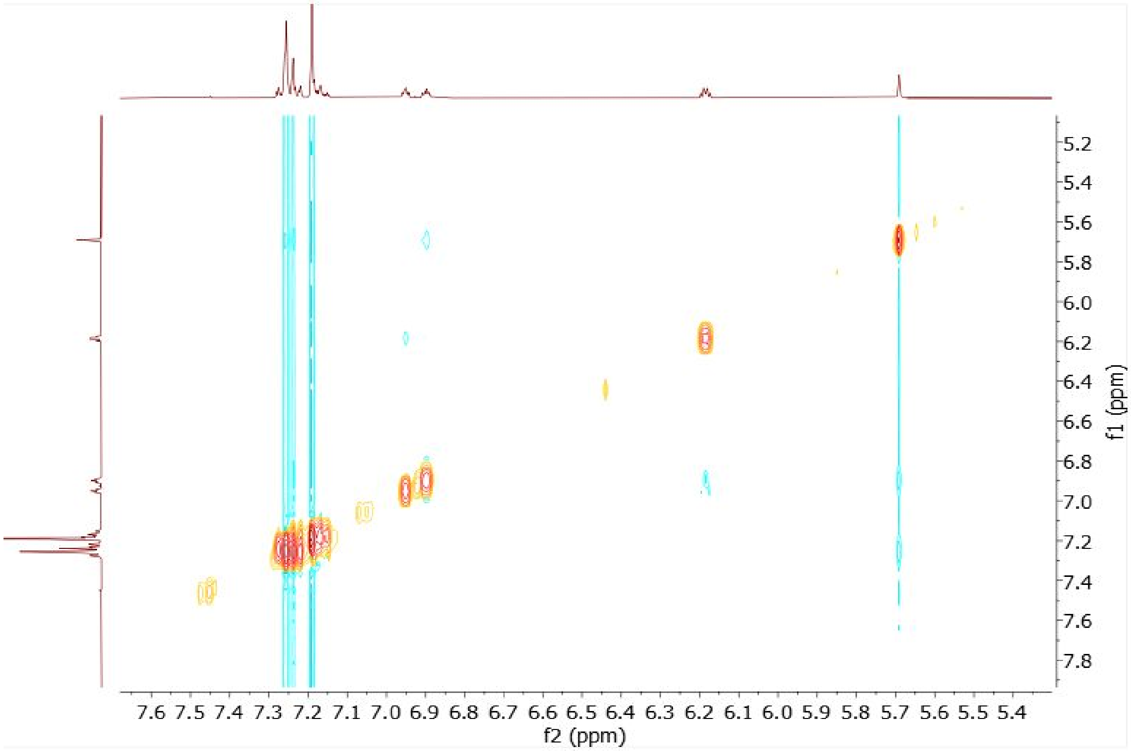

Fig S125: NOESY NMR spectrum of 2,2-diphenyl-1-(1*H*-pyrrol-2-yl)ethan-1-one (**26**) in $CDCl_3$.

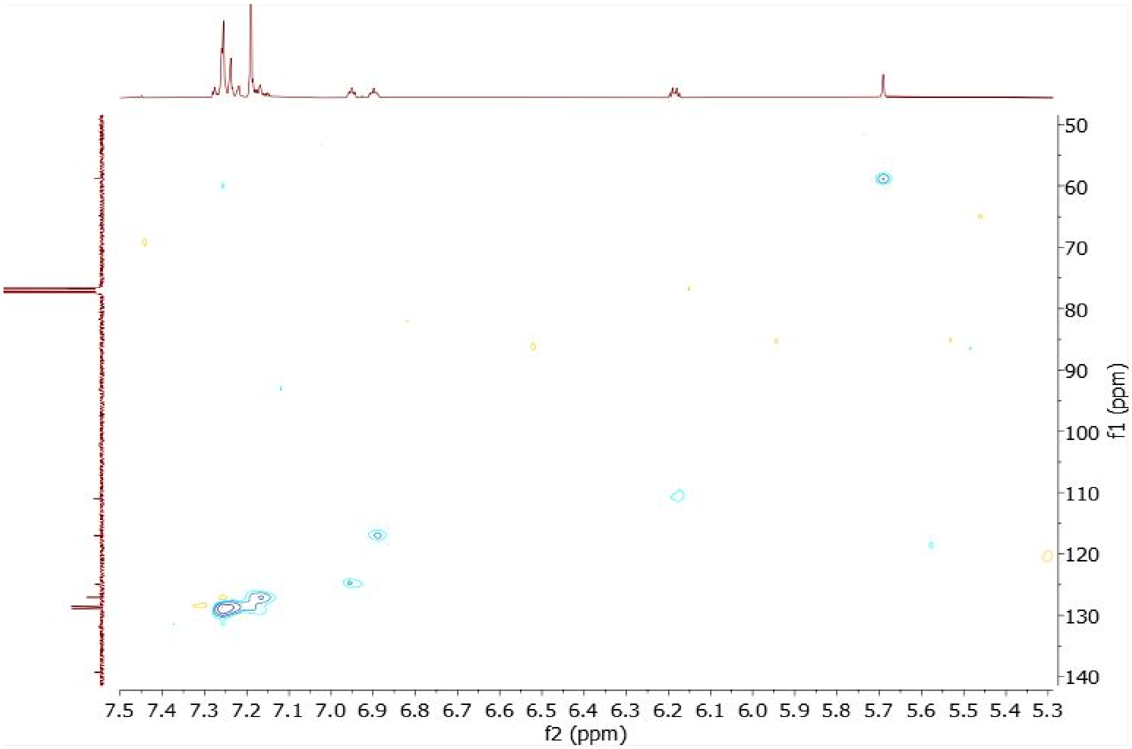

Fig S126: HSQC NMR spectrum of 2,2-diphenyl-1-(1*H*-pyrrol-2-yl)ethan-1-one (**26**) in $CDCl_3$.

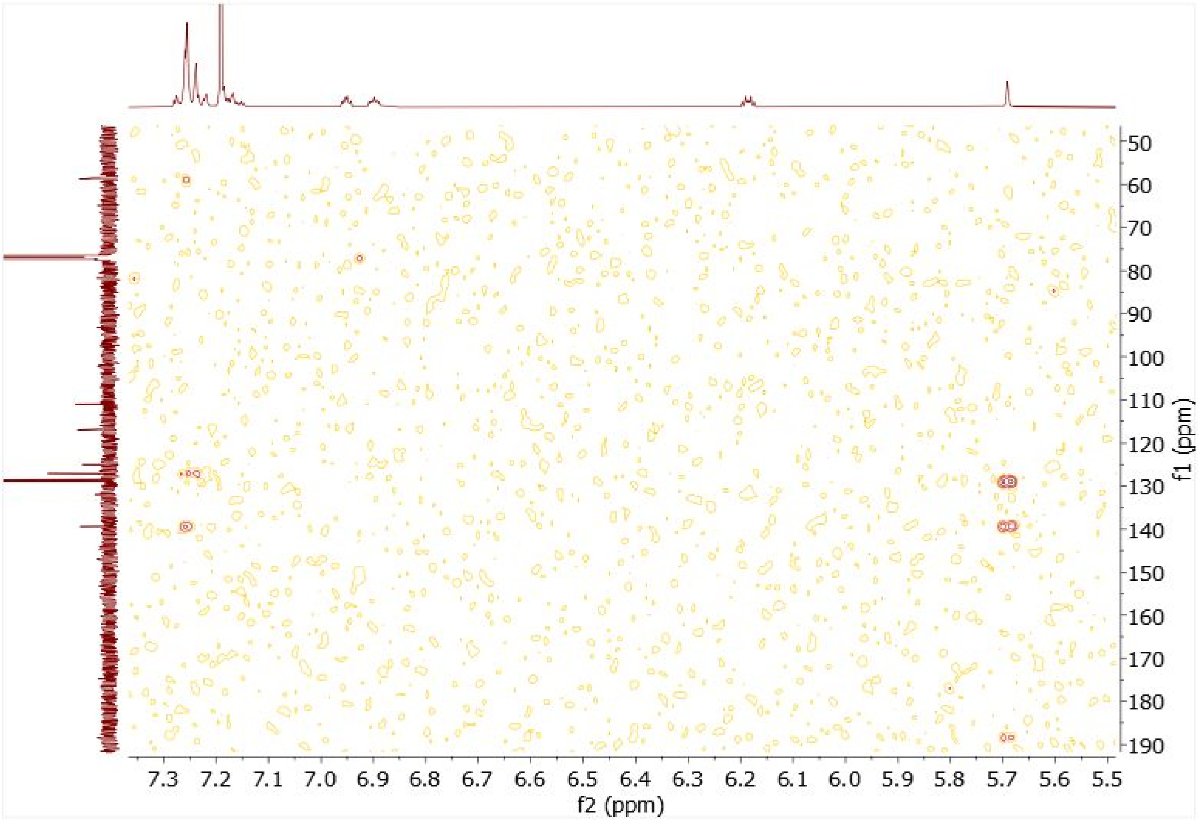

Fig S127: HMBC NMR spectrum of 2,2-diphenyl-1-(1*H*-pyrrol-2-yl)ethan-1-one (**26**) in $CDCl_3$.

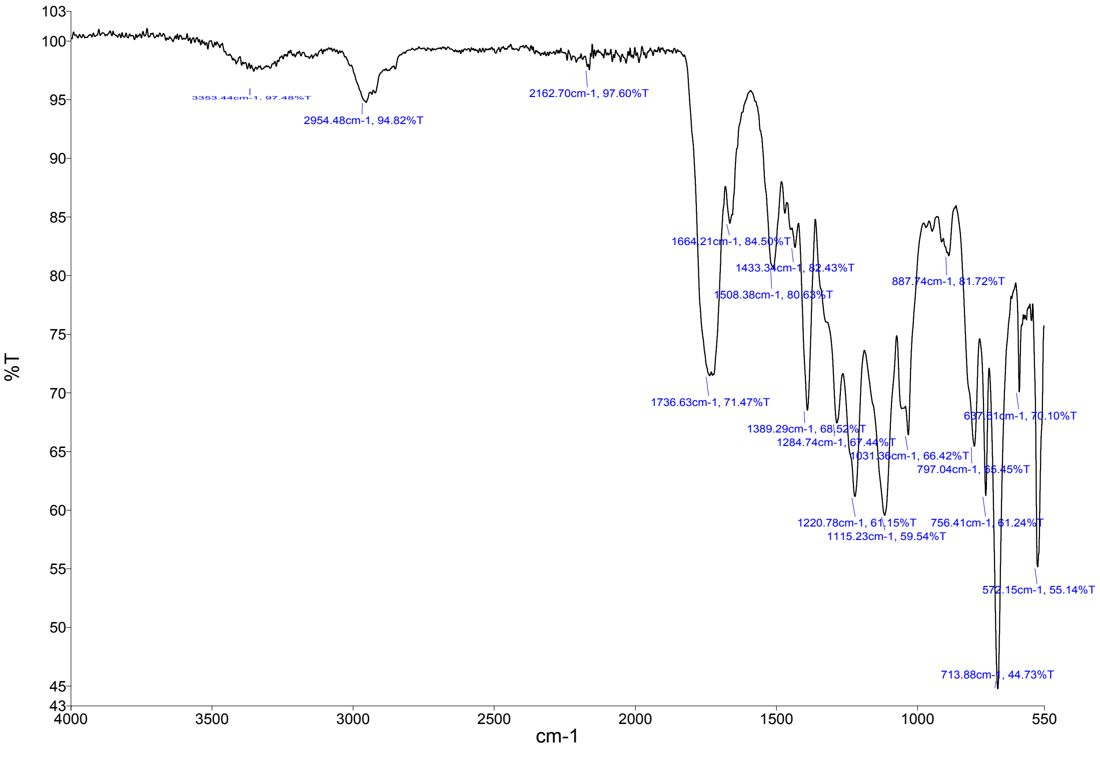

Fig S128: IR (neat) spectrum of 2,2-diphenyl-1-(1*H*-pyrrol-2-yl)ethan-1-one (**26**)

2,2,2-Trichloroethyl 2-(2-(4-*iso*-butylphenyl)propanoyl)-1*H*-pyrrole-1-carboxylate (27)

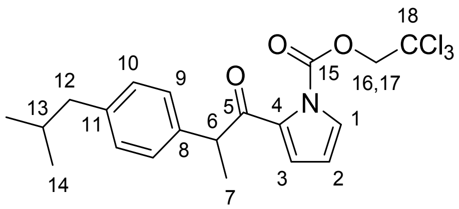

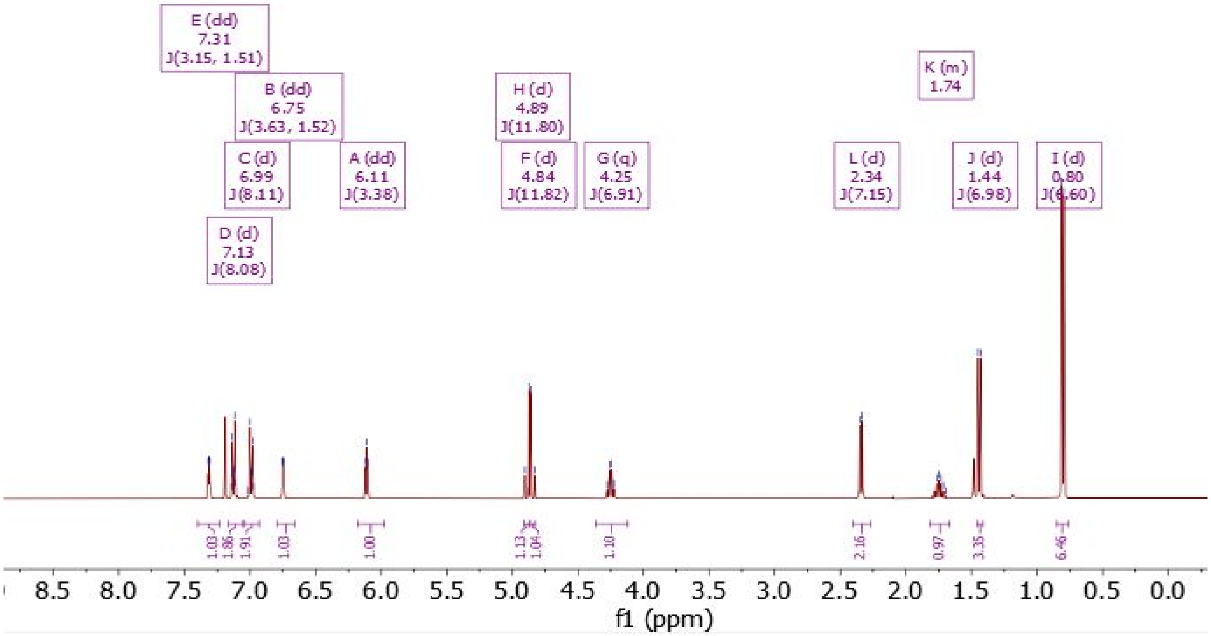

Fig S129: ^{1}H NMR (400 MHz) spectrum of 2,2,2-trichloroethyl 2-(2-(4-*iso*-butylphenyl)propanoyl)-1*H*-pyrrole-1-carboxylate (**27**) in $CDCl_3$

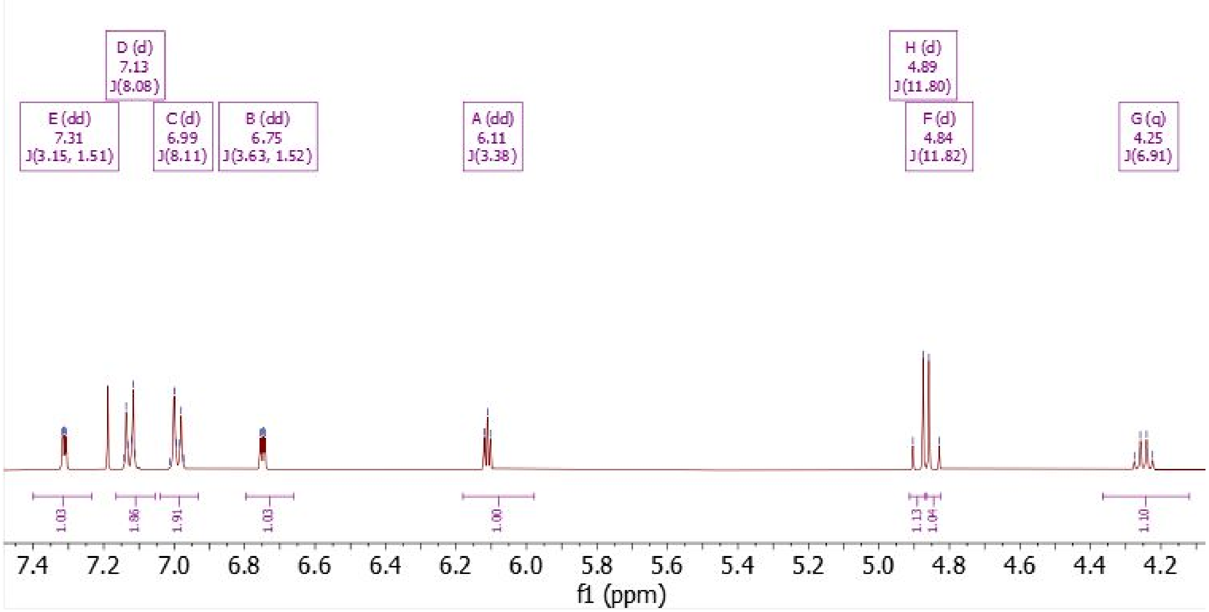

Fig S130: Zoomed region of ^{1}H NMR (400 MHz) spectrum of 2,2,2-trichloroethyl 2-(2-(4-*iso*-butylphenyl)propanoyl)-1*H*-pyrrole-1-carboxylate (**27**) in $CDCl_3$

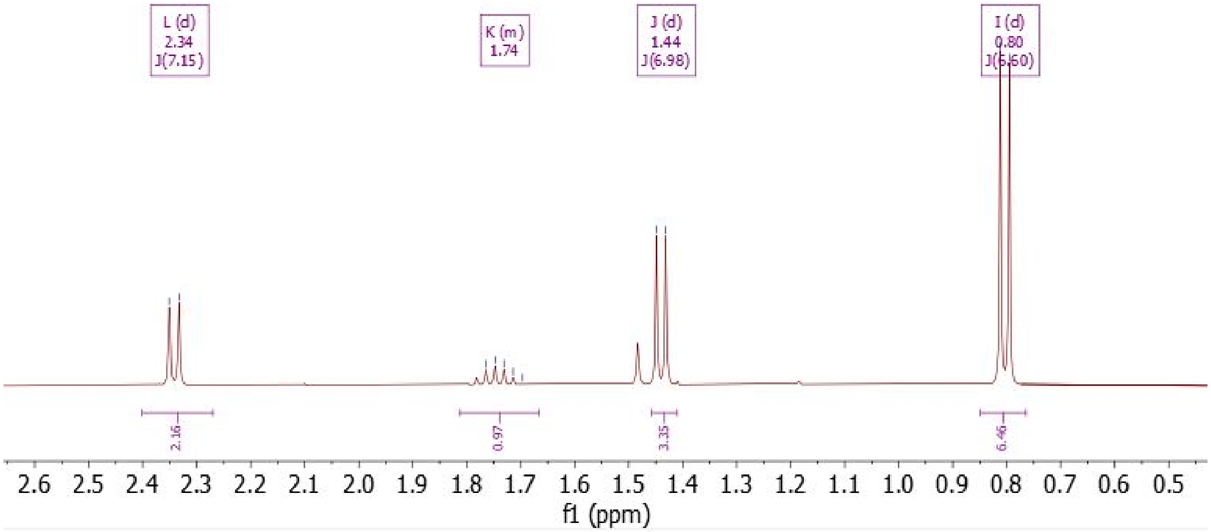

Fig S131: Zoomed region of ^{1}H NMR (400 MHz) spectrum of 2,2,2-trichloroethyl 2-(2-(4-*iso*-butylphenyl)propanoyl)-1*H*-pyrrole-1-carboxylate (**27**) in $CDCl_3$

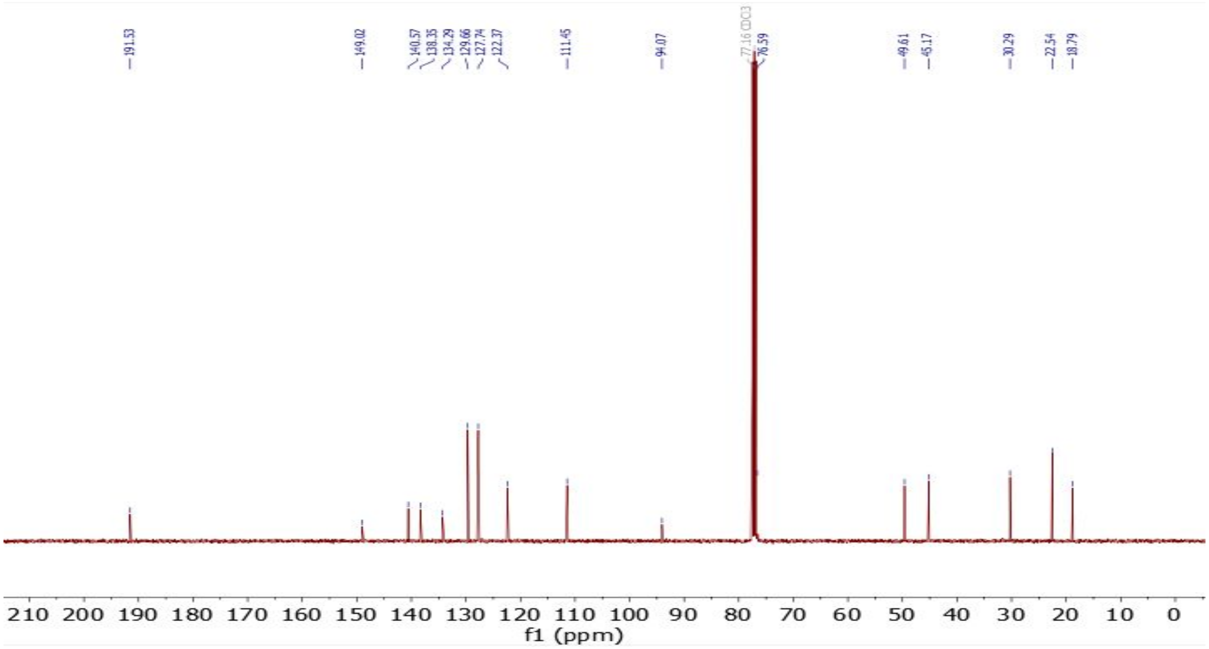

Fig S132: $^{13}C\{^{1}H\}$ NMR (101 MHz) spectrum of 2,2,2-trichloroethyl 2-(2-(4-*iso*-butylphenyl)propanoyl)-1*H*-pyrrole-1-carboxylate (**27**) in $CDCl_3$

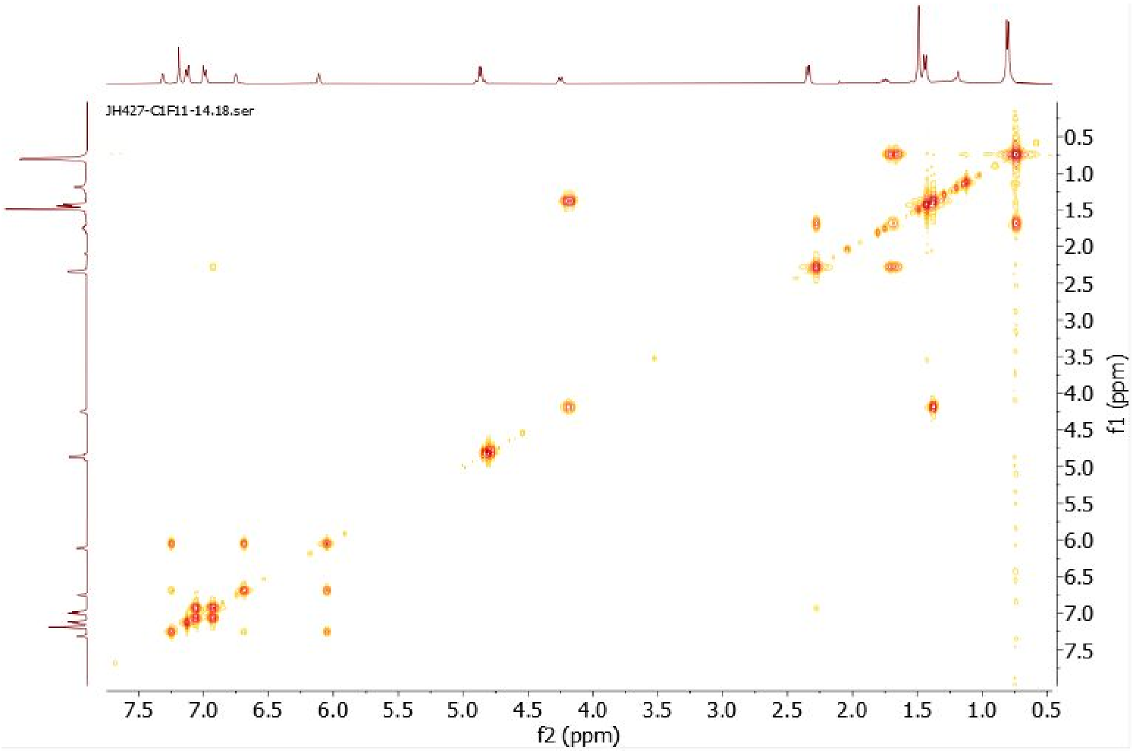

Fig S133: COSY NMR spectrum of 2,2,2-trichloroethyl 2-(2-(4-*iso*-butylphenyl)propanoyl)-1*H*-pyrrole-1-carboxylate (**27**) in $CDCl_3$

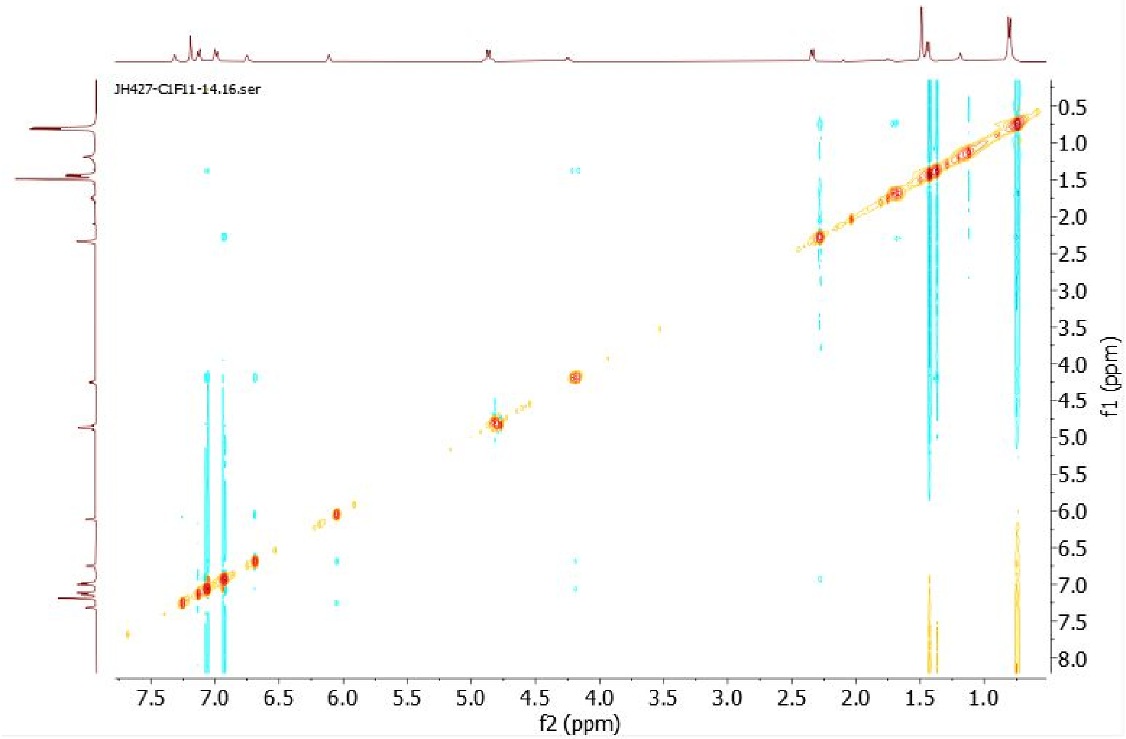

Fig S134: NOESY NMR spectrum of 2,2,2-trichloroethyl 2-(2-(4-*iso*-butylphenyl)propanoyl)-1*H*-pyrrole-1-carboxylate (**27**) in $CDCl_3$

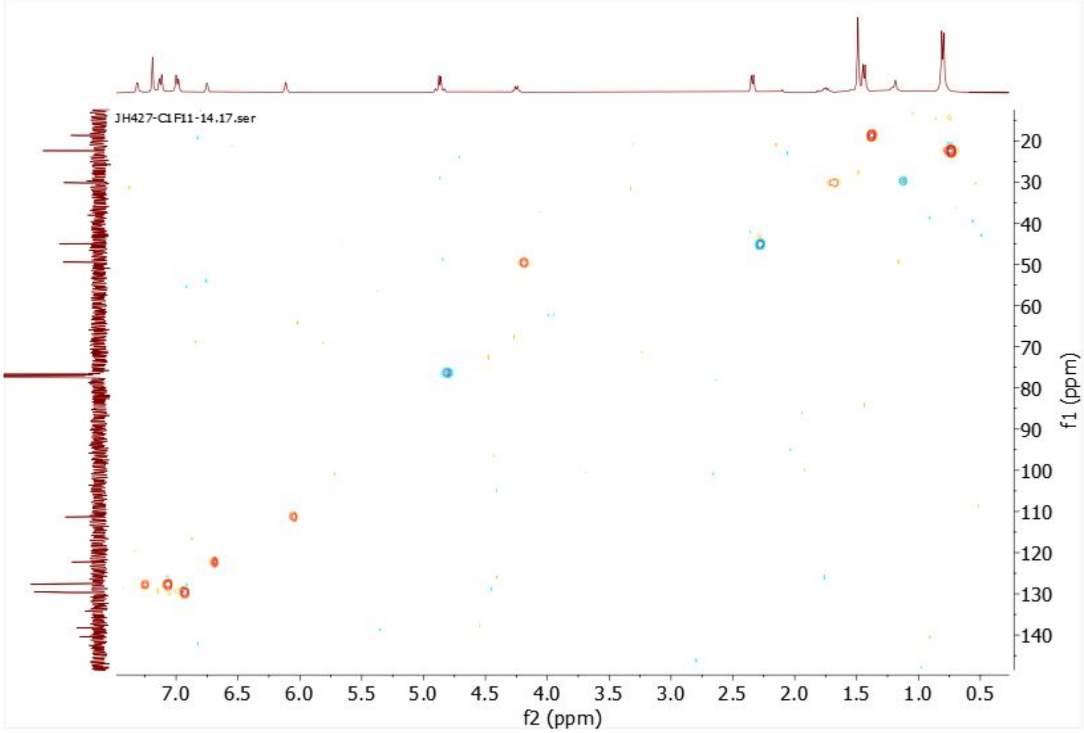

Fig S135: HSQC NMR spectrum of 22,2,2-trichloroethyl 2-(2-(4-*iso*-butylphenyl)propanoyl)-1*H*-pyrrole-1-carboxylate (**27**) in $CDCl_3$

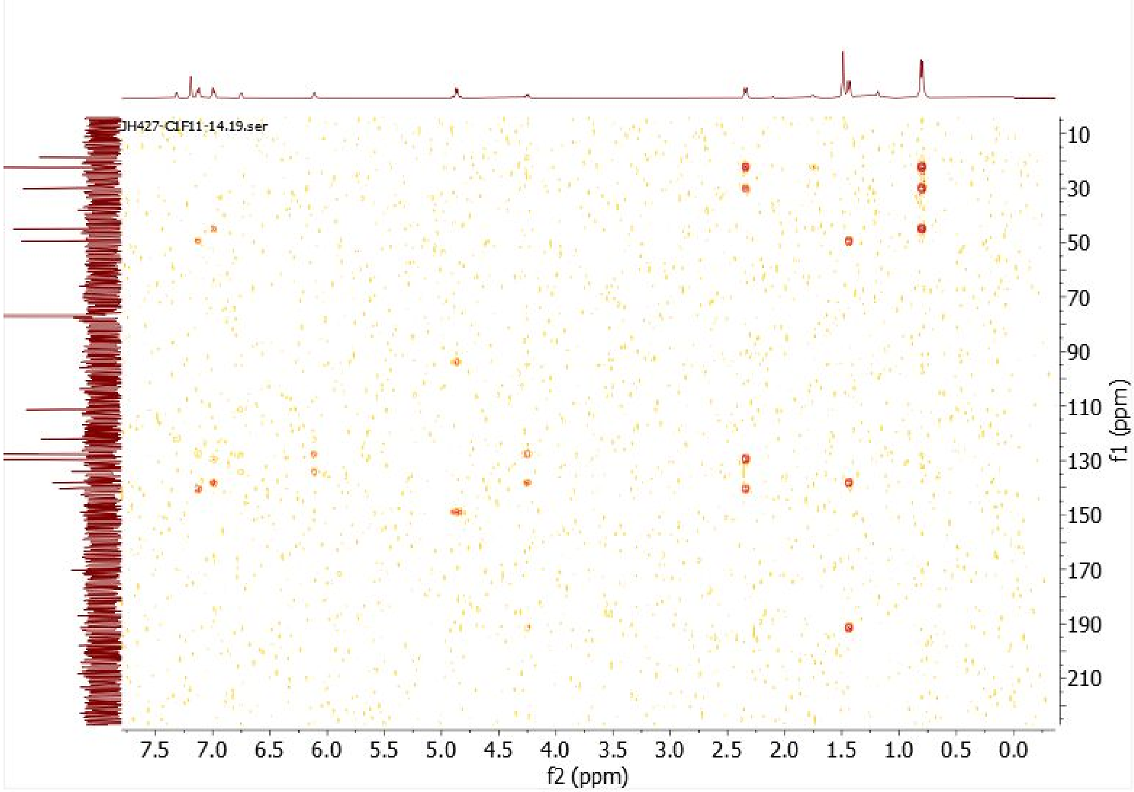

Fig S136: HMBC NMR spectrum of 2,2,2-trichloroethyl 2-(2-(4-*iso*-butylphenyl)propanoyl)-1*H*-pyrrole-1-carboxylate (**27**) in $CDCl_3$

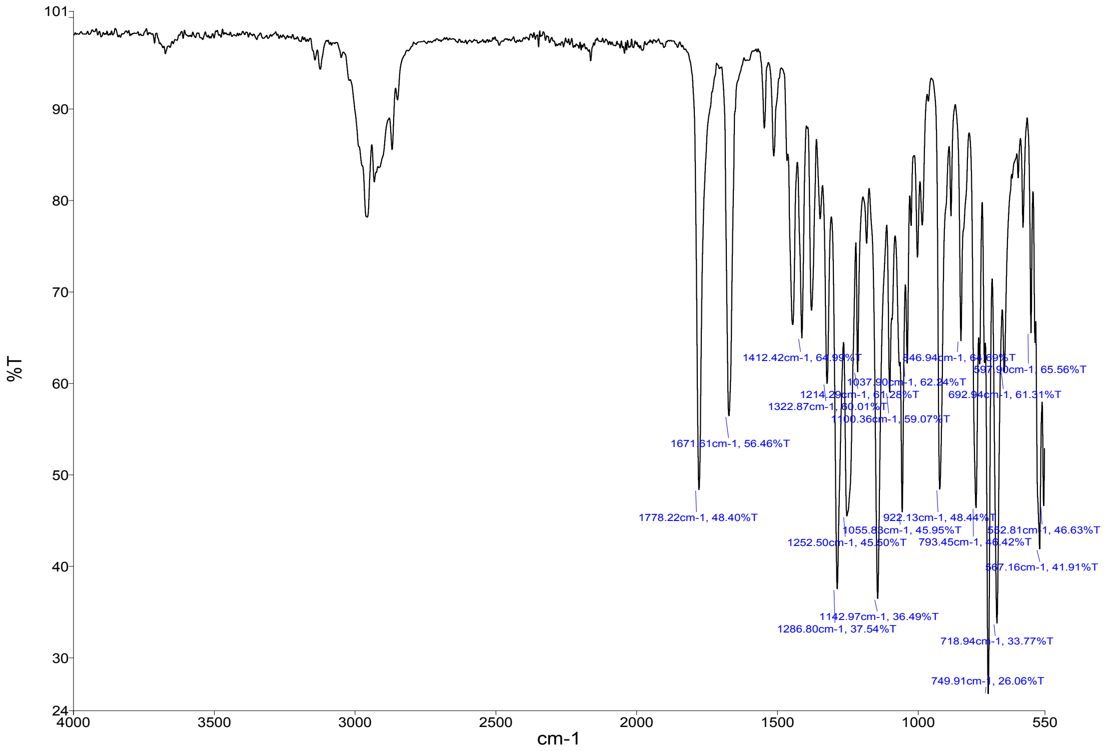

Fig S137: IR spectrum of 2,2,2-trichloroethyl 2-(2-(4-*iso*-butylphenyl)propanoyl)-1*H*-pyrrole-1-carboxylate (**27**)

2-(4-*iso*-Butylphenyl)-1-(1H-pyrrol-2-yl)propan-1-one (28)

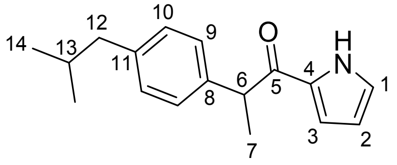

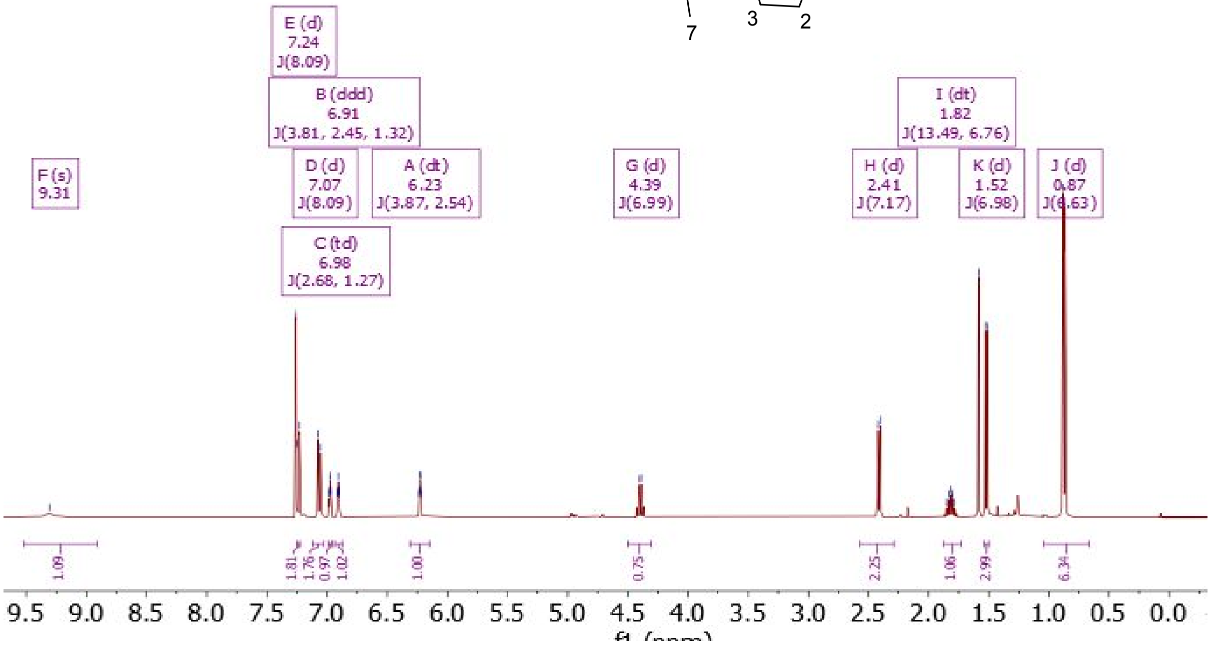

Fig S138: ^{1}H NMR (400 MHz) spectrum of 2-(4-*iso*-butylphenyl)-1-(1*H*-pyrrol-2-yl)propan-1-one (**28**) in $CDCl_3$

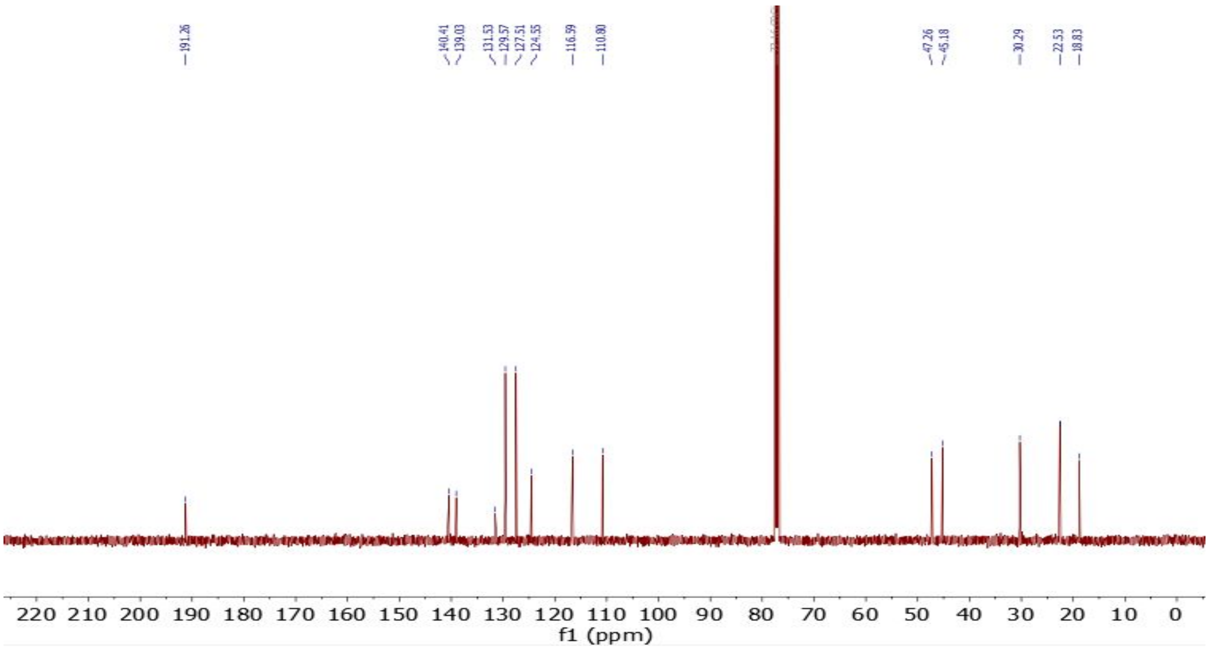

Fig S139: $^{13}C\{^{1}H\}$ NMR (101 MHz) spectrum of 2-(4-*iso*-butylphenyl)-1-(1*H*-pyrrol-2-yl)propan-1-one (**28**) in $CDCl_3$

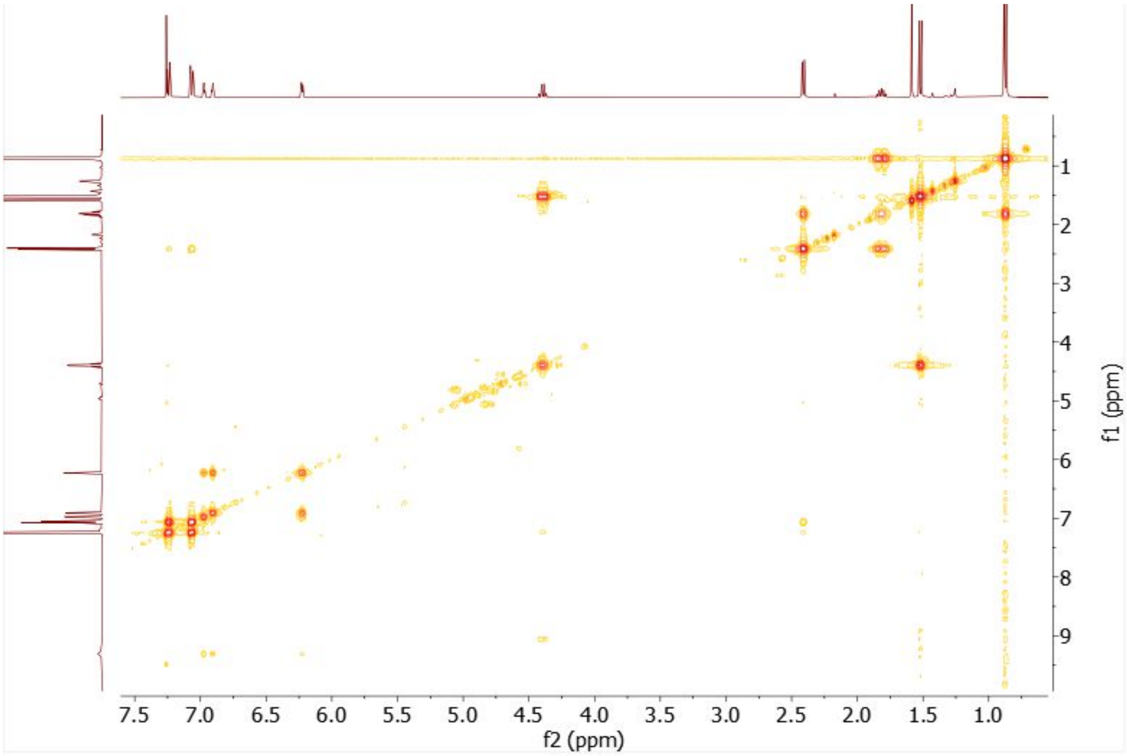

Fig S140: COSY NMR spectrum of 2-(4-*iso*-butylphenyl)-1-(1*H*-pyrrol-2-yl)propan-1-one (**28**) in $CDCl_3$

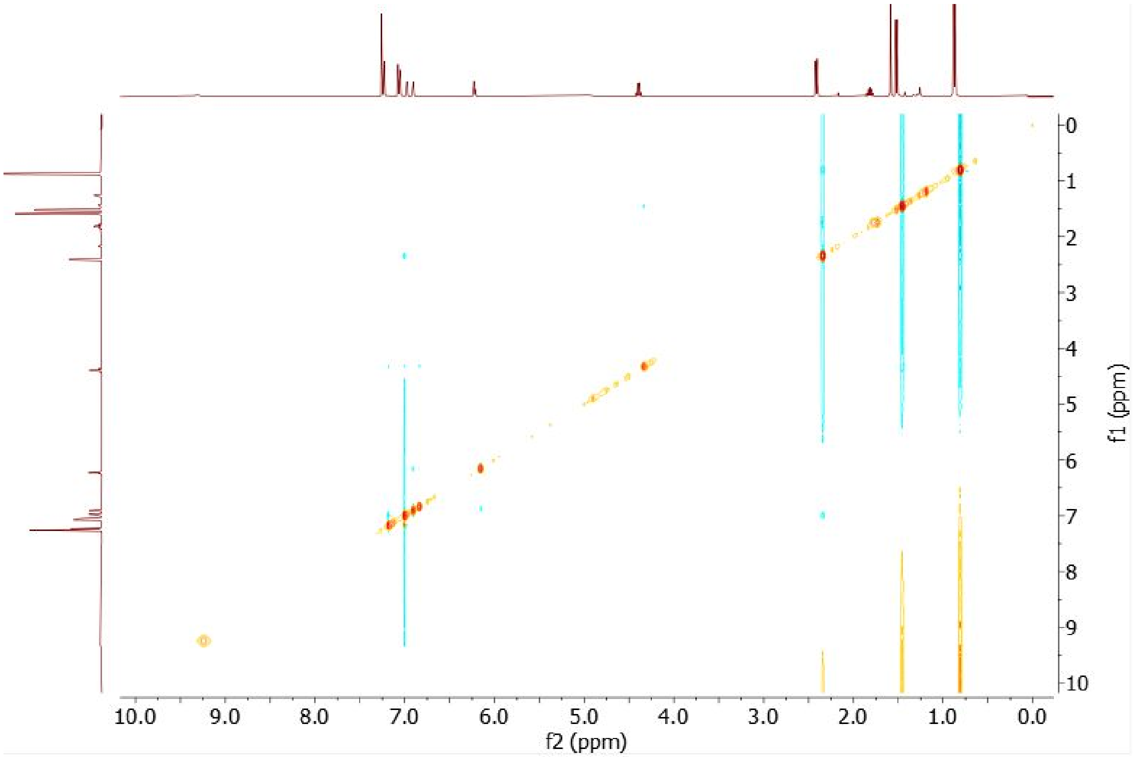

Fig S141: NOESY NMR spectrum of 2-(4-*iso*-butylphenyl)-1-(1*H*-pyrrol-2-yl)propan-1-one (**28**) in $CDCl_3$

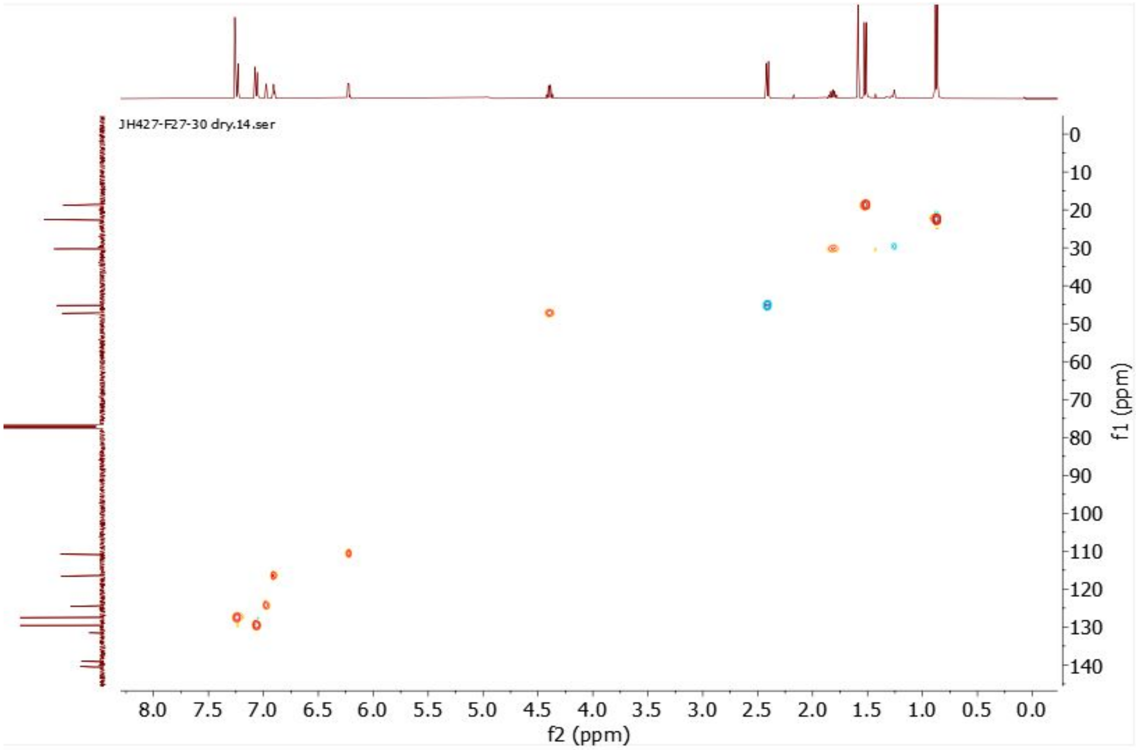

Fig S142: HSQC NMR spectrum of 2-(4-*iso*-butylphenyl)-1-(1*H*-pyrrol-2-yl)propan-1-one (**28**) in $CDCl_3$

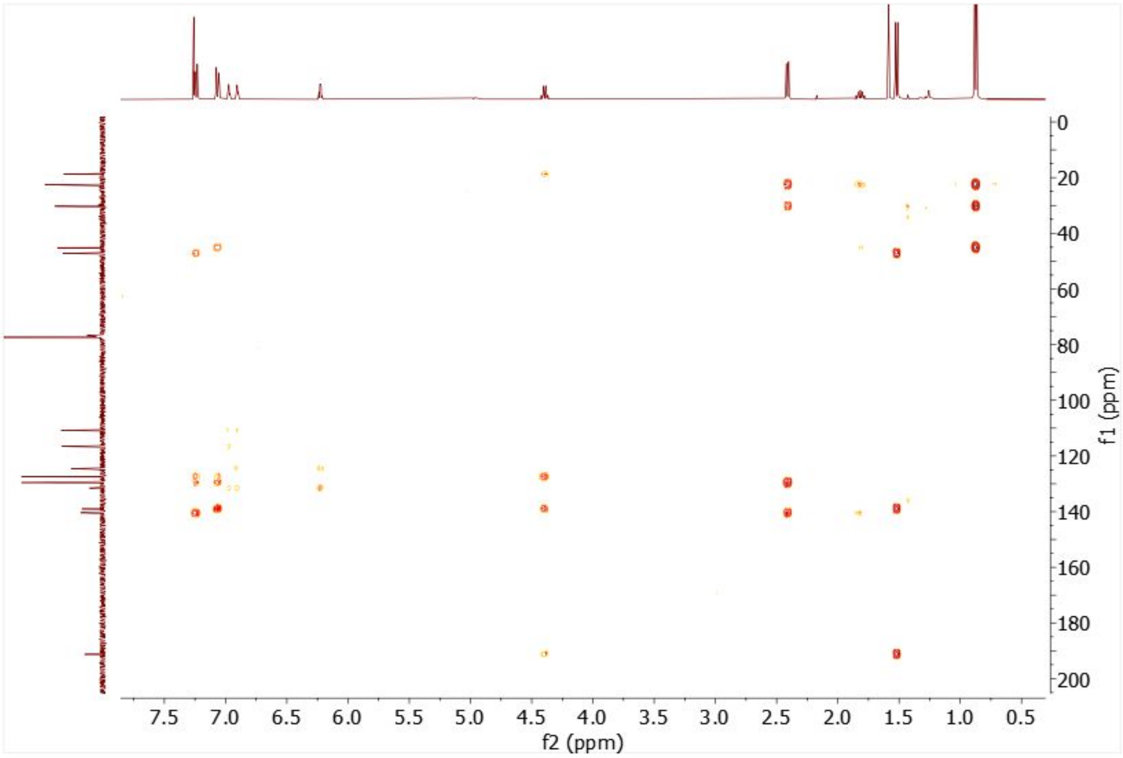

Fig S143: HMBC NMR spectrum of 2-(4-*iso*-butylphenyl)-1-(1*H*-pyrrol-2-yl)propan-1-one (**28**) in $CDCl_3$

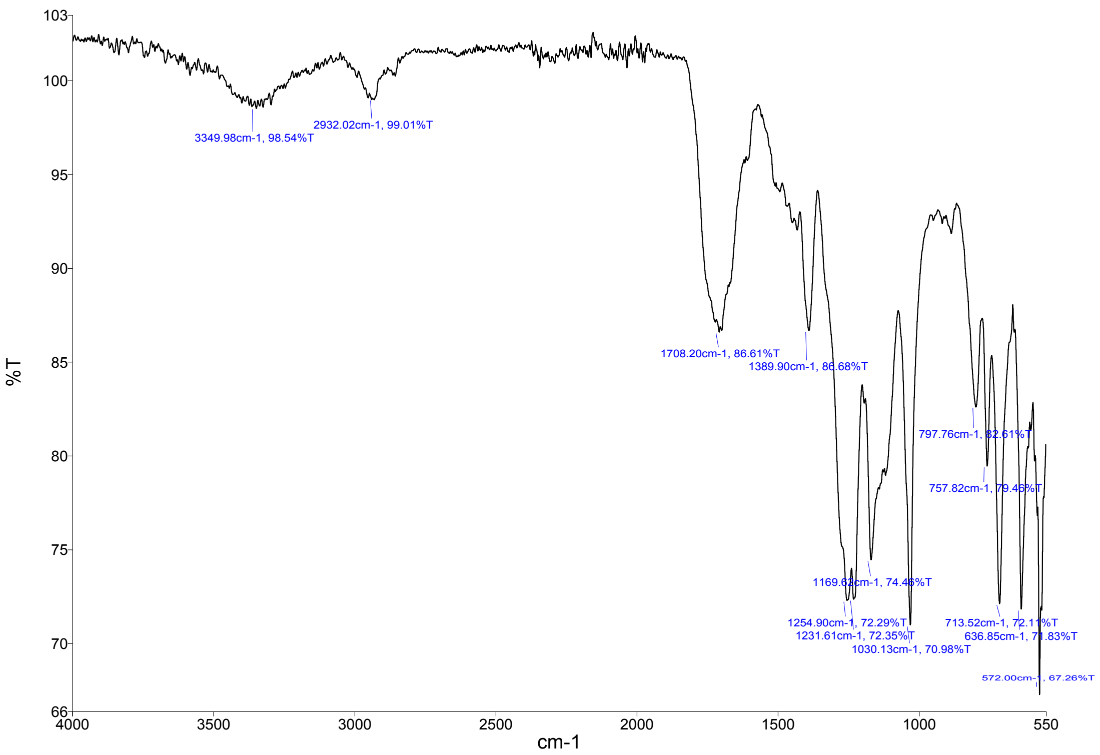

Fig S144: IR (neat) spectrum of 2-(4-*iso*-butylphenyl)-1-(1*H*-pyrrol-2-yl)propan-1-one (**28**)

(2-Acetyl-9*H*-fluoren-9-yl)methyl 2-acetyl-1*H*-pyrrole-1-carboxylate (29)

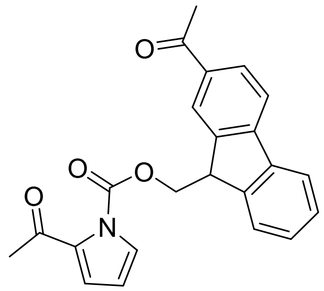

crude product

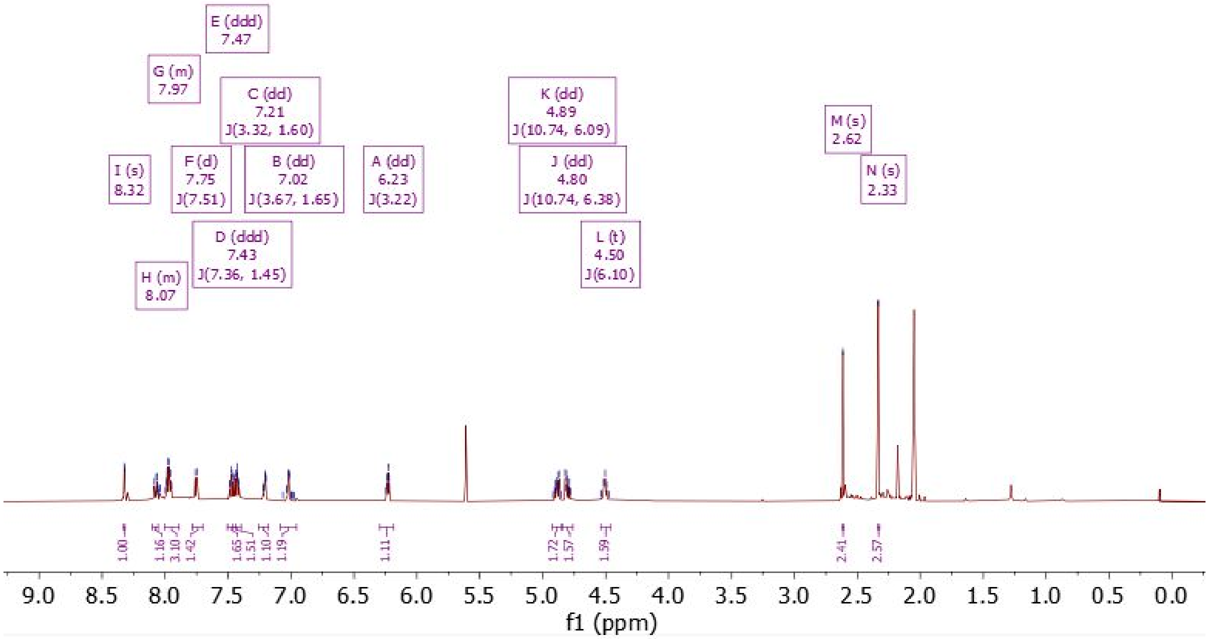

Fig S145: ^{1}H Crude NMR (500 MHz) spectrum of (2-acetyl-9*H*-fluoren-9-yl)methyl 2-acetyl-1*H*-pyrrole-1-carboxylate (**29**) in d_6-acetone.

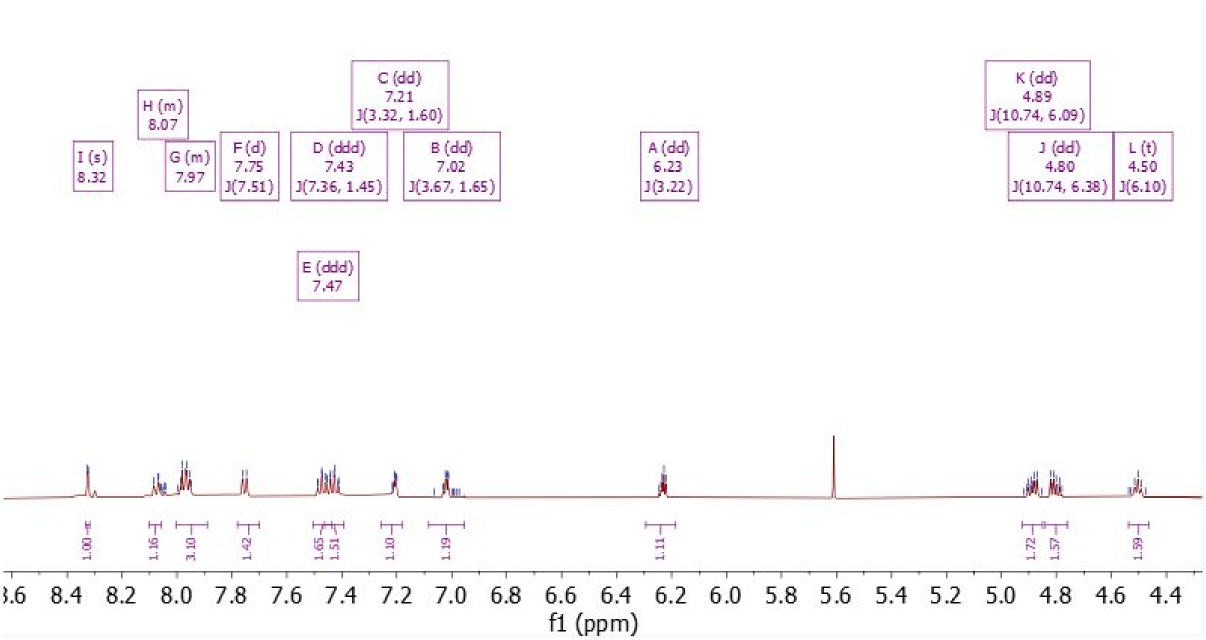

Fig S146: Zoomed ^{1}H Crude (500 MHz) NMR spectrum of (2-acetyl-9*H*-fluoren-9-yl)methyl 2-acetyl-1*H*-pyrrole-1-carboxylate (**29**) in d_6-acetone.

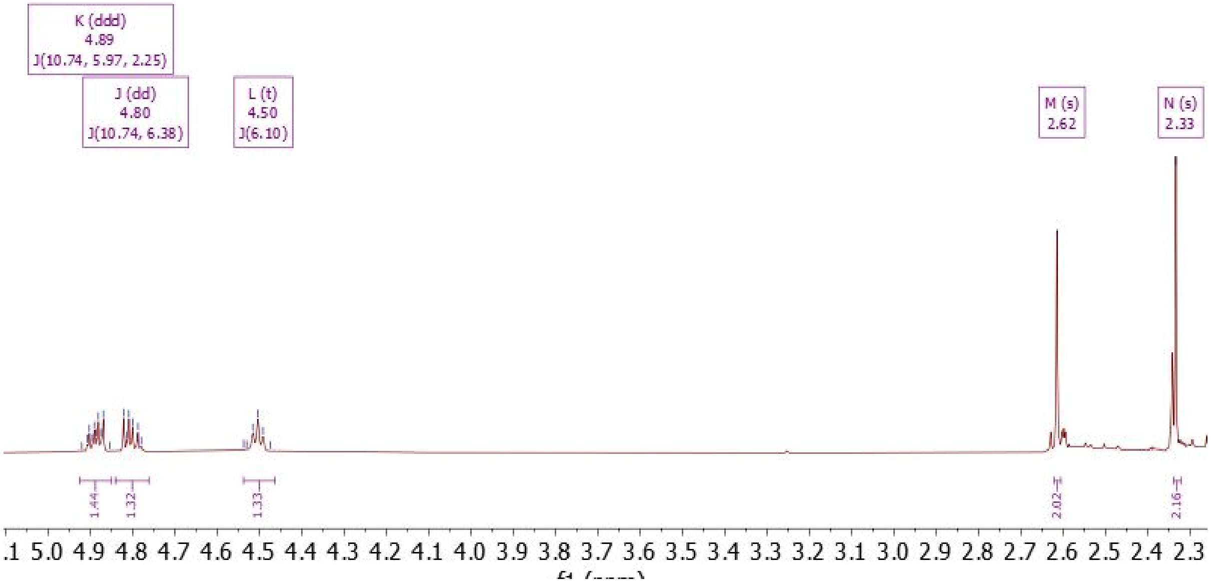

Fig S147: Zoomed ^{1}H Crude NMR spectrum of (2-acetyl-9*H*-fluoren-9-yl)methyl 2-acetyl-1*H*-pyrrole-1-carboxylate (**29**) in d_6-acetone.

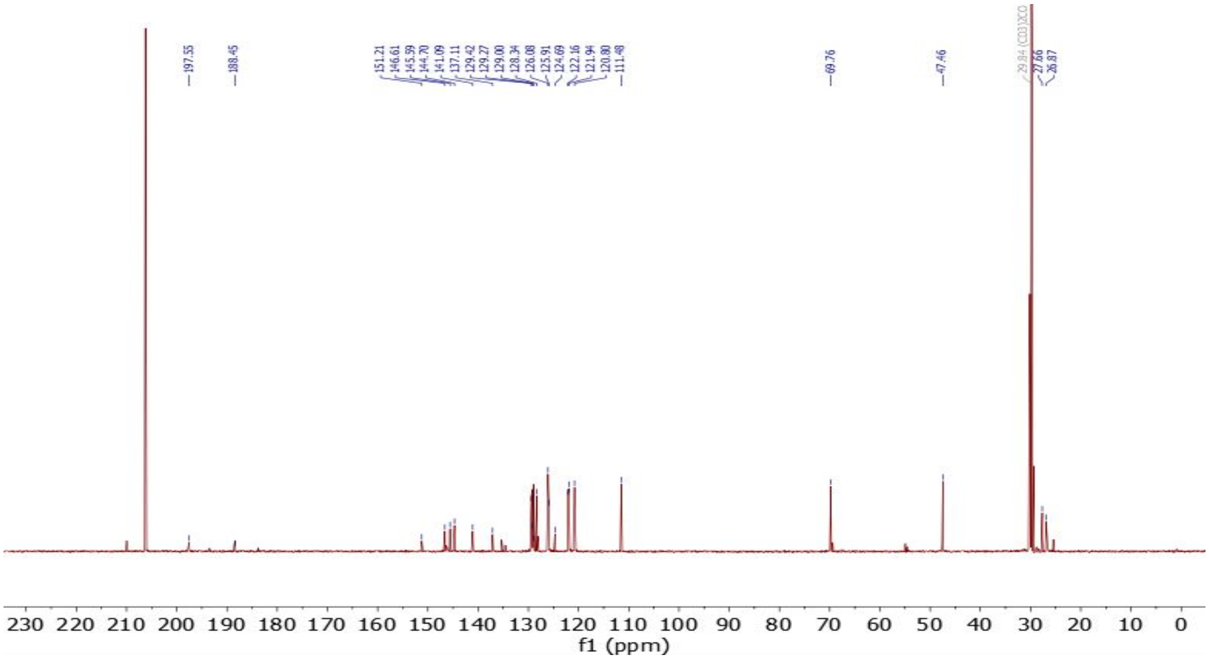

Fig S148: $^{13}C\{^{1}H\}$ Crude NMR (126 MHz) spectrum of (2-acetyl-9*H*-fluoren-9-yl)methyl 2-acetyl-1*H*-pyrrole-1-carboxylate (**29**) in d_6-acetone.

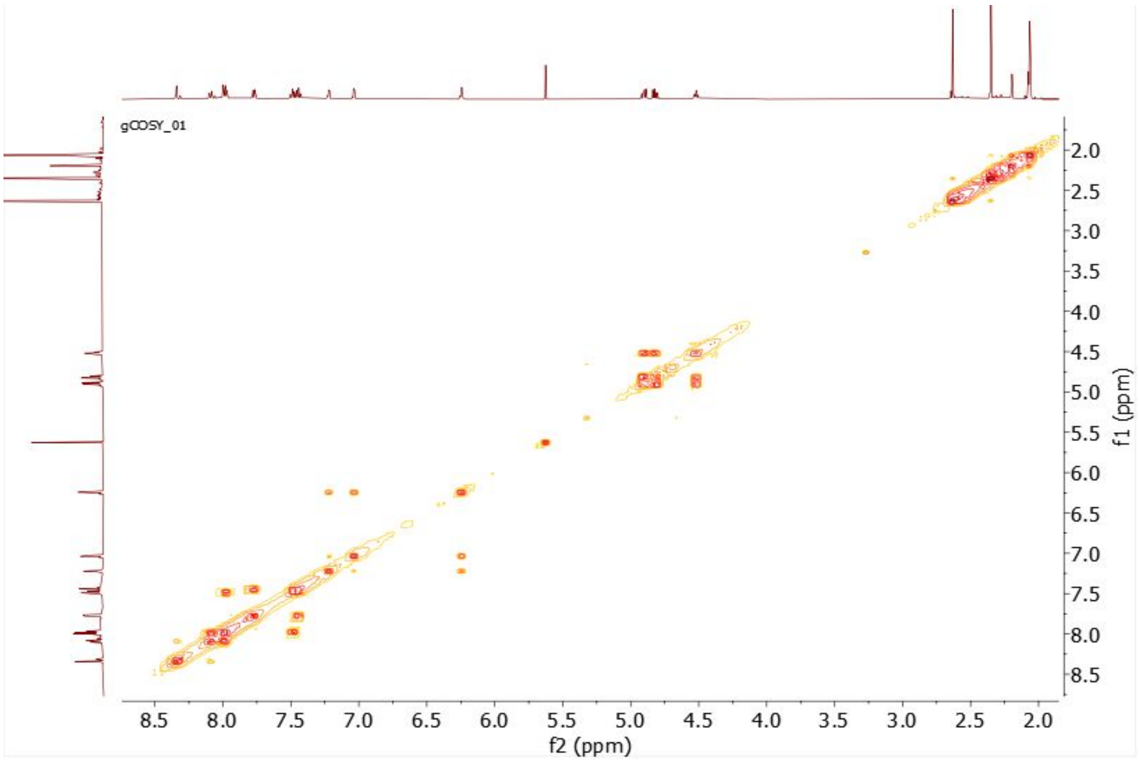

Fig S149: COSY Crude NMR spectrum of (2-acetyl-9*H*-fluoren-9-yl)methyl 2-acetyl-1*H*-pyrrole-1-carboxylate (**29**) in d_6-acetone.

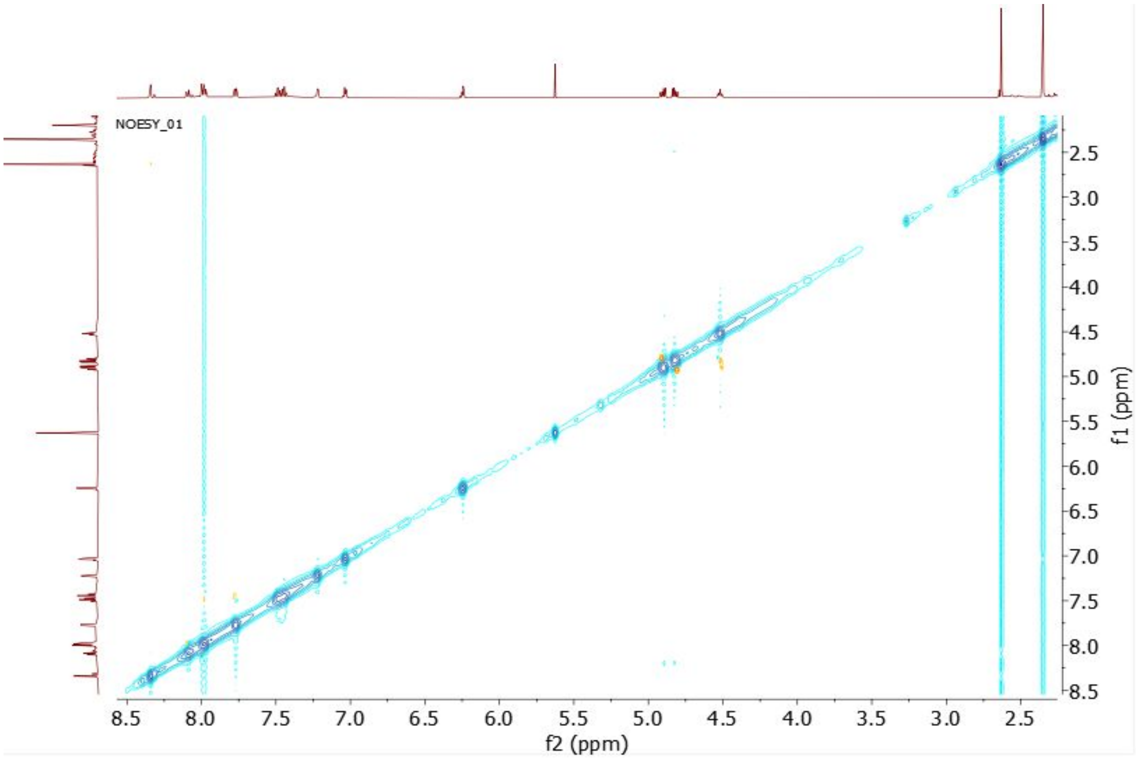

Fig S150: NOESY Crude NMR spectrum of (2-acetyl-9*H*-fluoren-9-yl)methyl 2-acetyl-1*H*-pyrrole-1-carboxylate (**29**) in d_6-acetone.

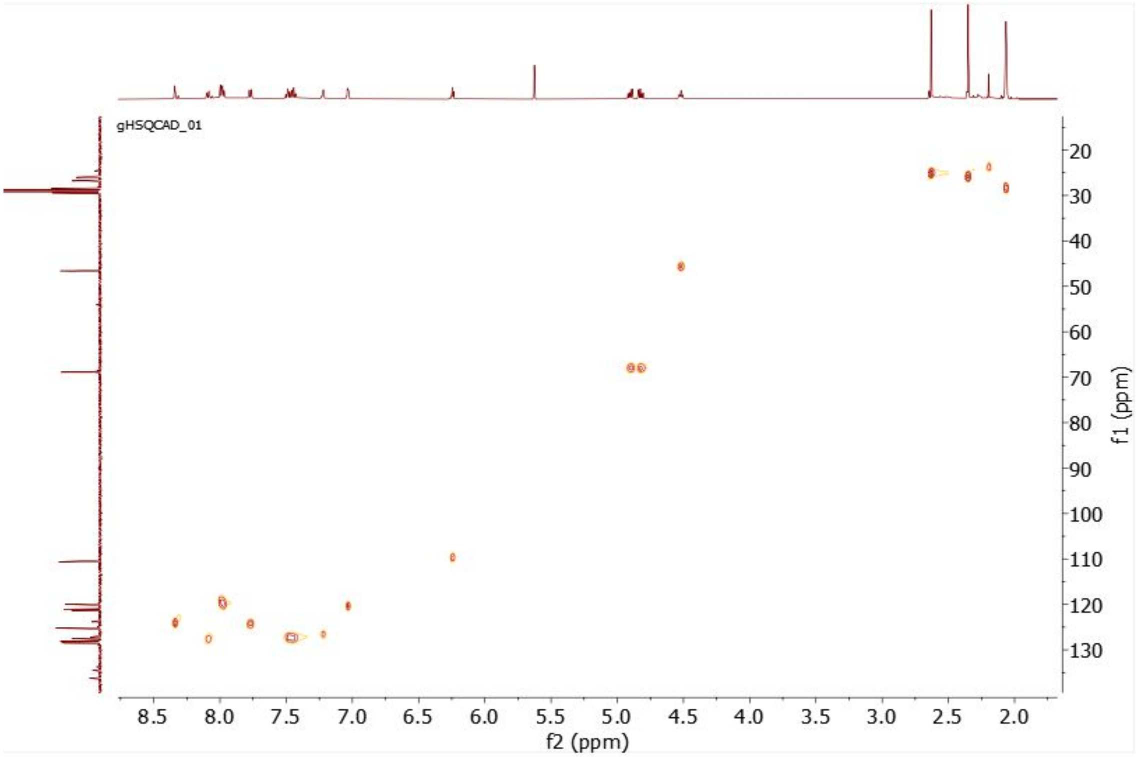

Fig S151: HSQC Crude NMR spectrum of (2-acetyl-9*H*-fluoren-9-yl)methyl 2-acetyl-1*H*-pyrrole-1-carboxylate (**29**) in d_6-acetone.

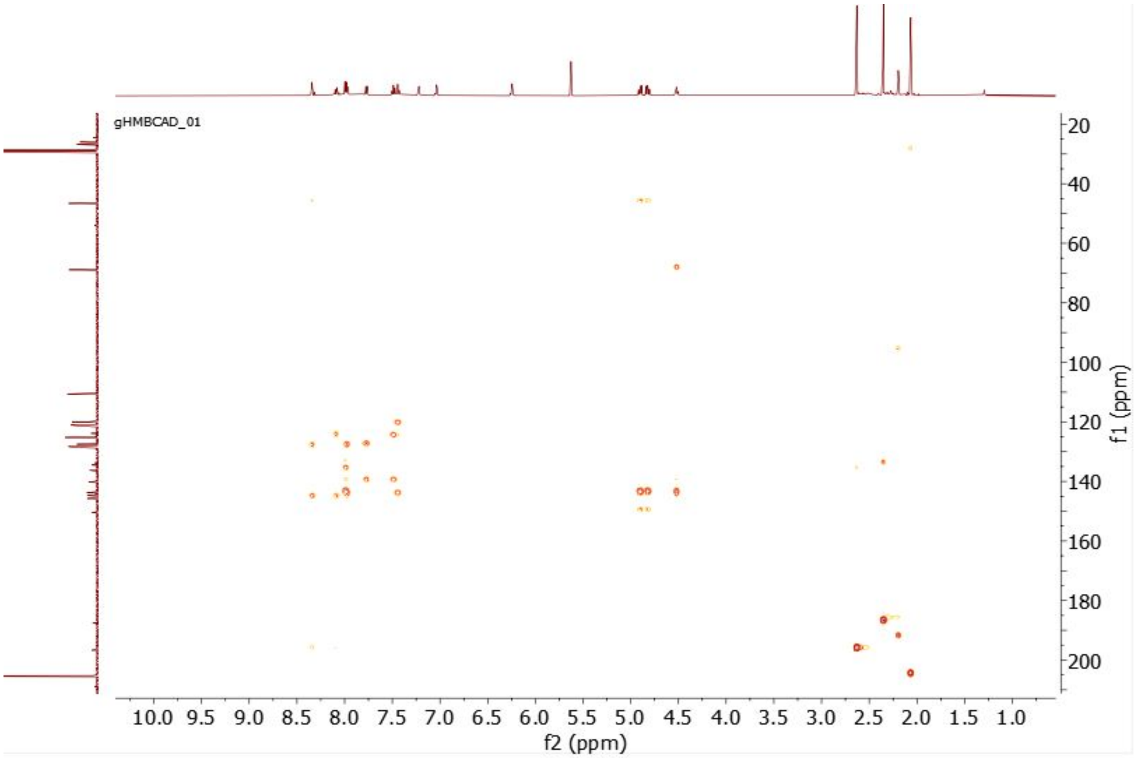

Fig S152: HMBC Crude NMR spectrum of (2-acetyl-9*H*-fluoren-9-yl)methyl 2-acetyl-1*H*-pyrrole-1-carboxylate (**29**) in d_6-acetone.

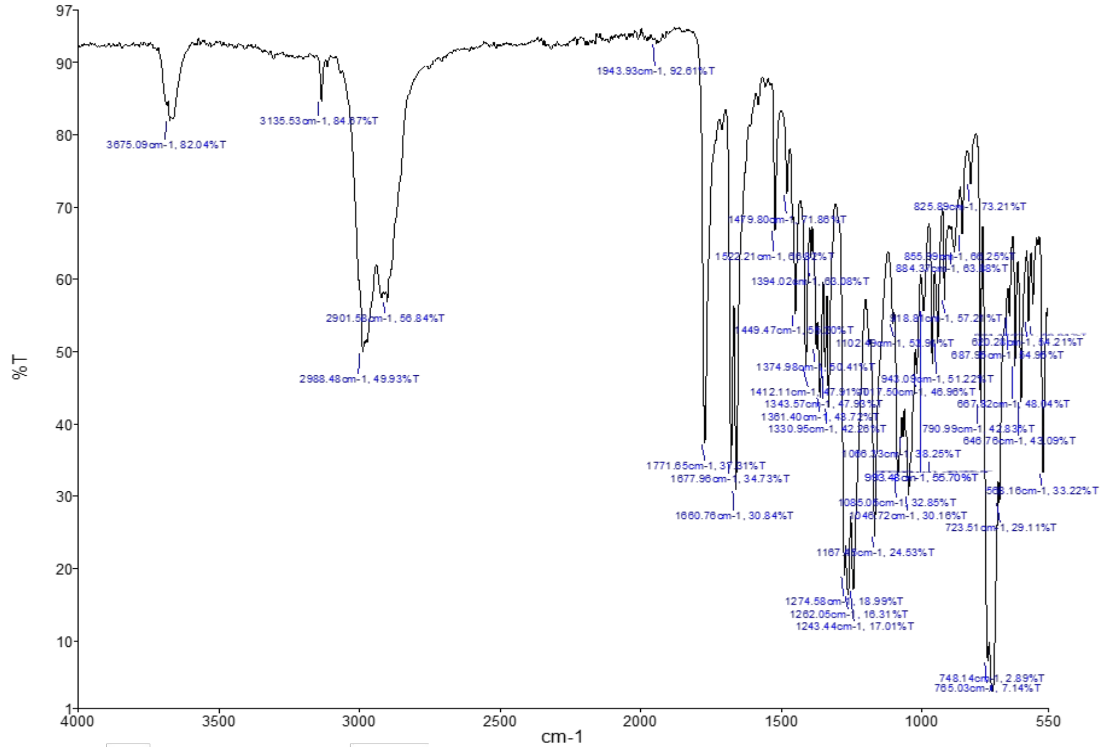

Fig S153: IR (neat) spectrum of crude (2-acetyl-9*H*-fluoren-9-yl)methyl 2-acetyl-1*H*-pyrrole-1-carboxylate (**29**)

2,2-Dimethyl-1-(1-tosyl-1*H*-pyrrol-3-yl)propan-1-one (31)

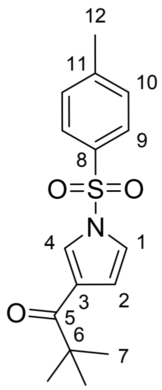

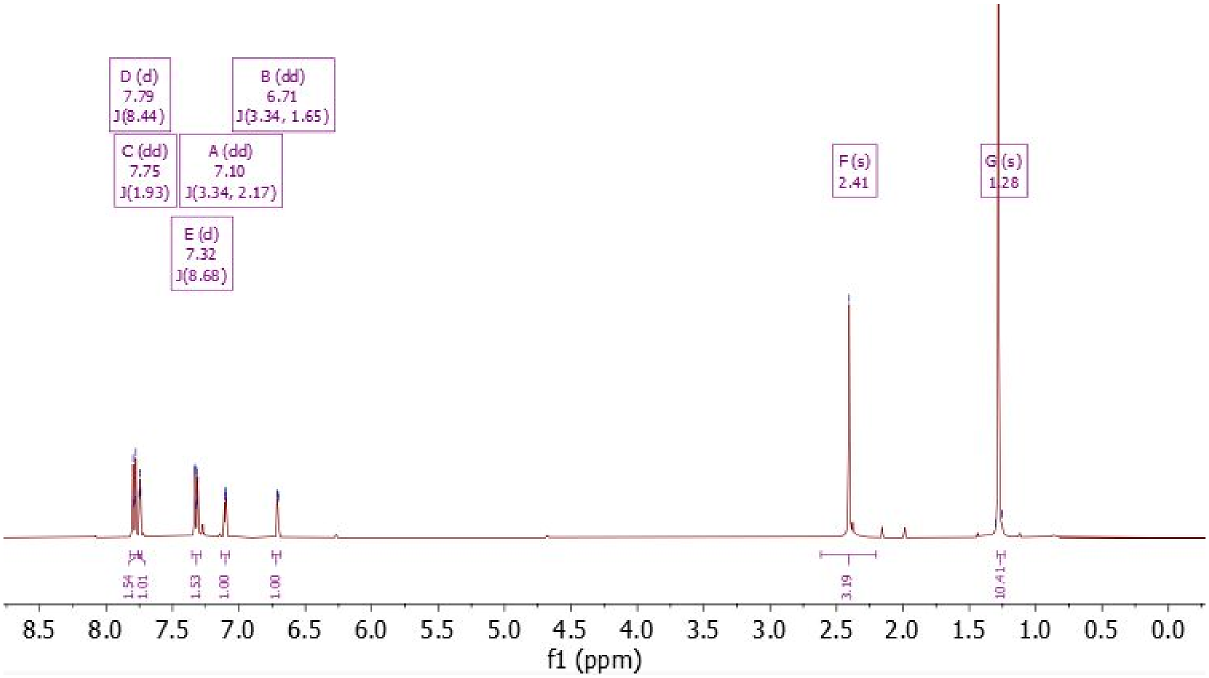

Fig S154: ^{1}H NMR (400 MHz) spectrum of ,2-dimethyl-1-(1-tosyl-1*H*-pyrrol-3-yl)propan-1-one (**31**) in $CDCl_3$.

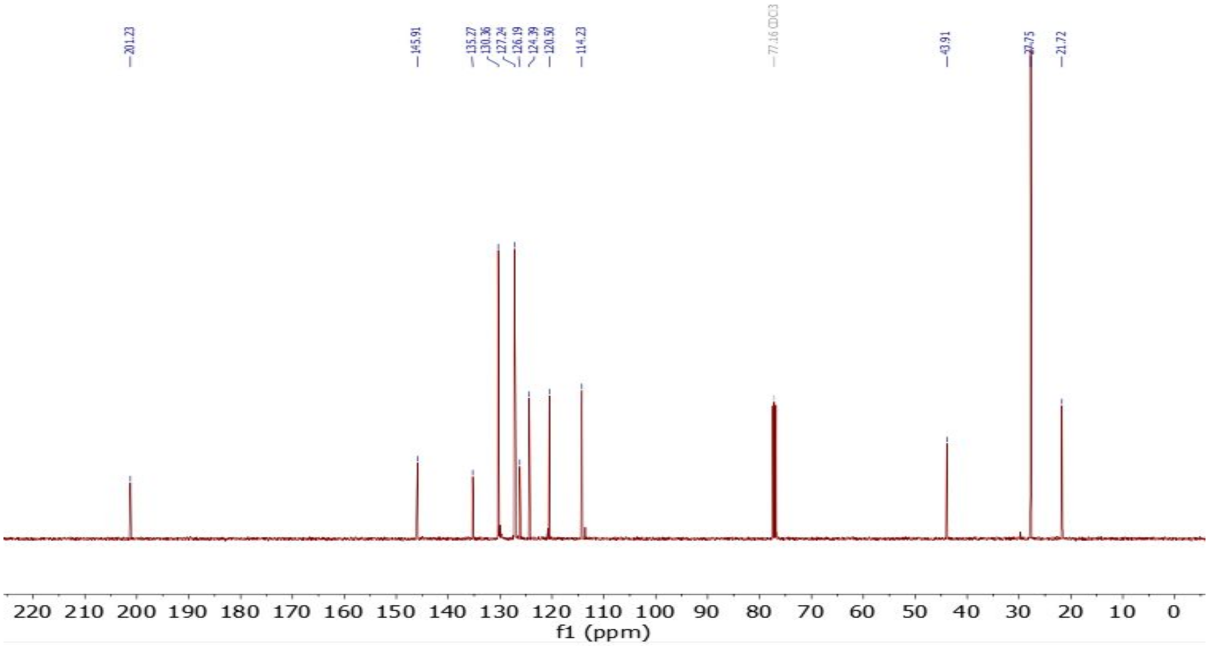

Fig S155: $^{13}C\{^{1}H\}$ NMR (101 MHz) spectrum of 2,2-dimethyl-1-(1-tosyl-1*H*-pyrrol-3-yl)propan-1-one (**31**) in $CDCl_3$.

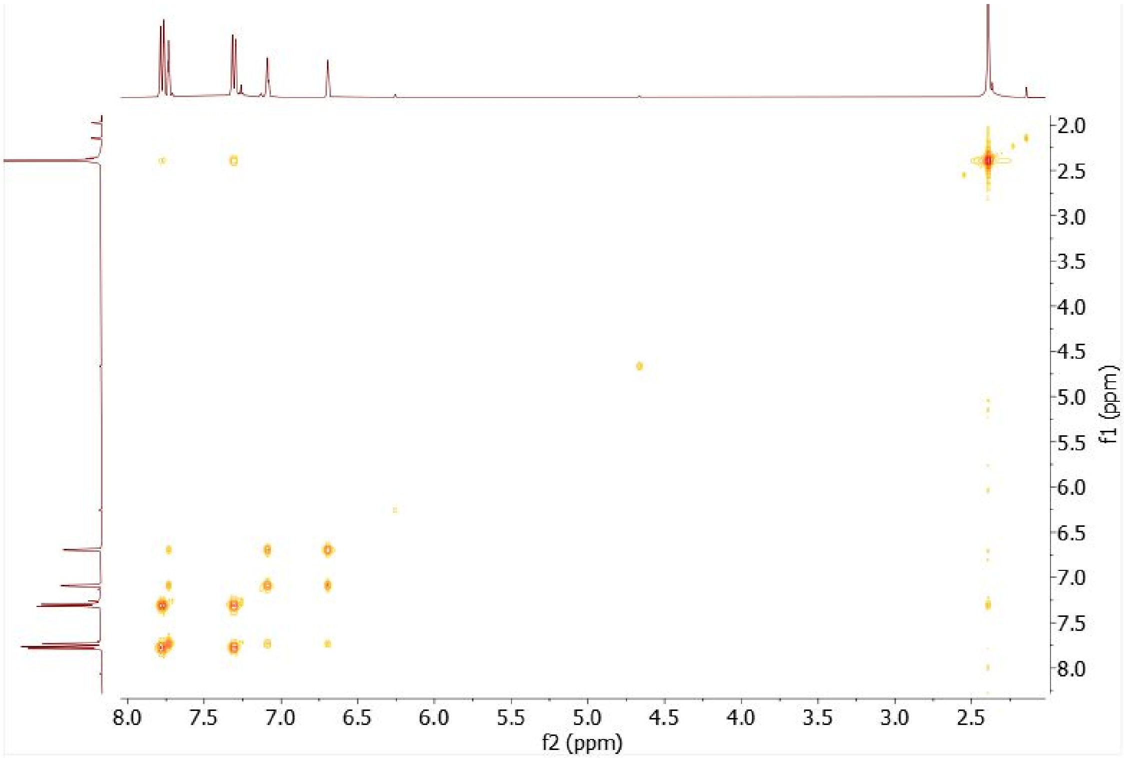

Fig S156: COSY NMR spectrum of 2,2-dimethyl-1-(1-tosyl-1*H*-pyrrol-3-yl)propan-1-one (**31**) in $CDCl_3$.

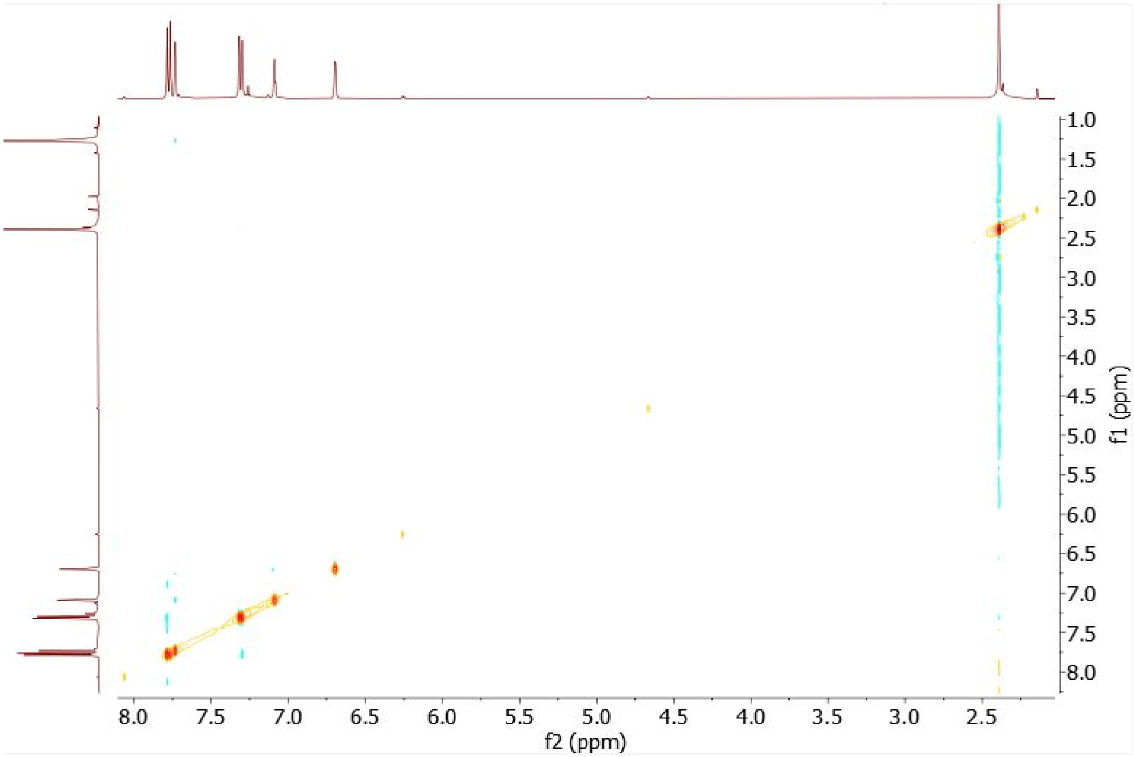

Fig S157: NOESY NMR spectrum of 2,2-dimethyl-1-(1-tosyl-1*H*-pyrrol-3-yl)propan-1-one (**31**) in $CDCl_3$.

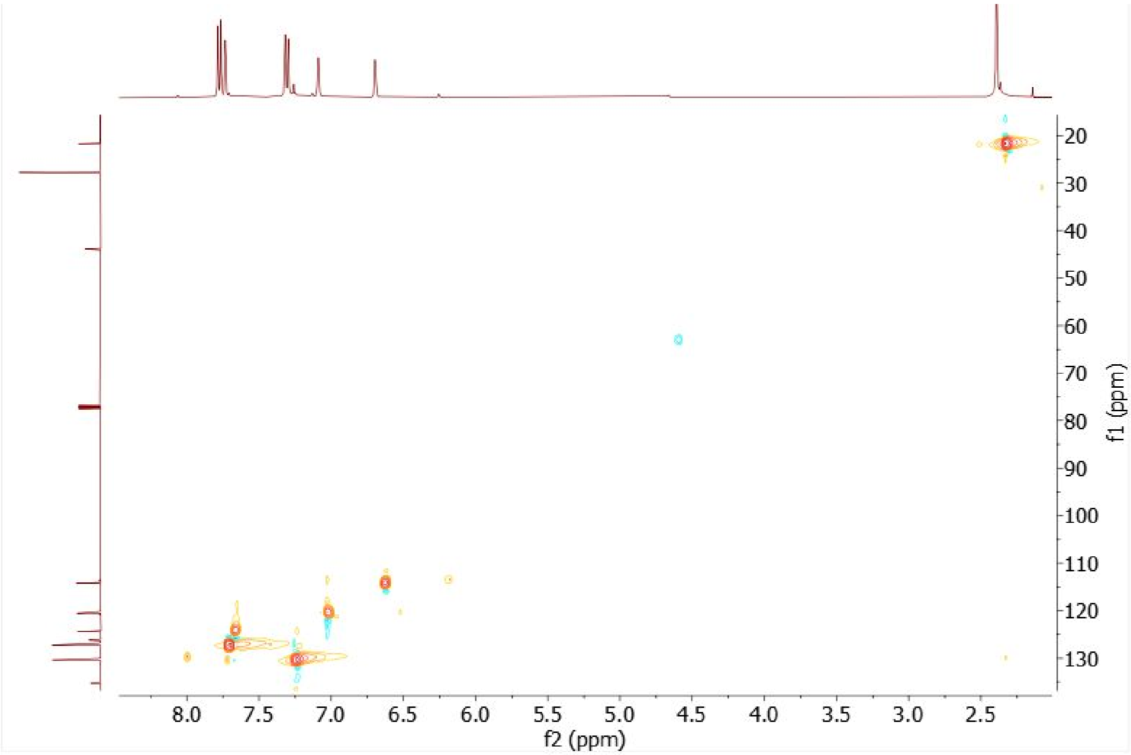

Fig S158: HSQC NMR spectrum of 2,2-dimethyl-1-(1-tosyl-1*H*-pyrrol-3-yl)propan-1-one (**31**) in $CDCl_3$.

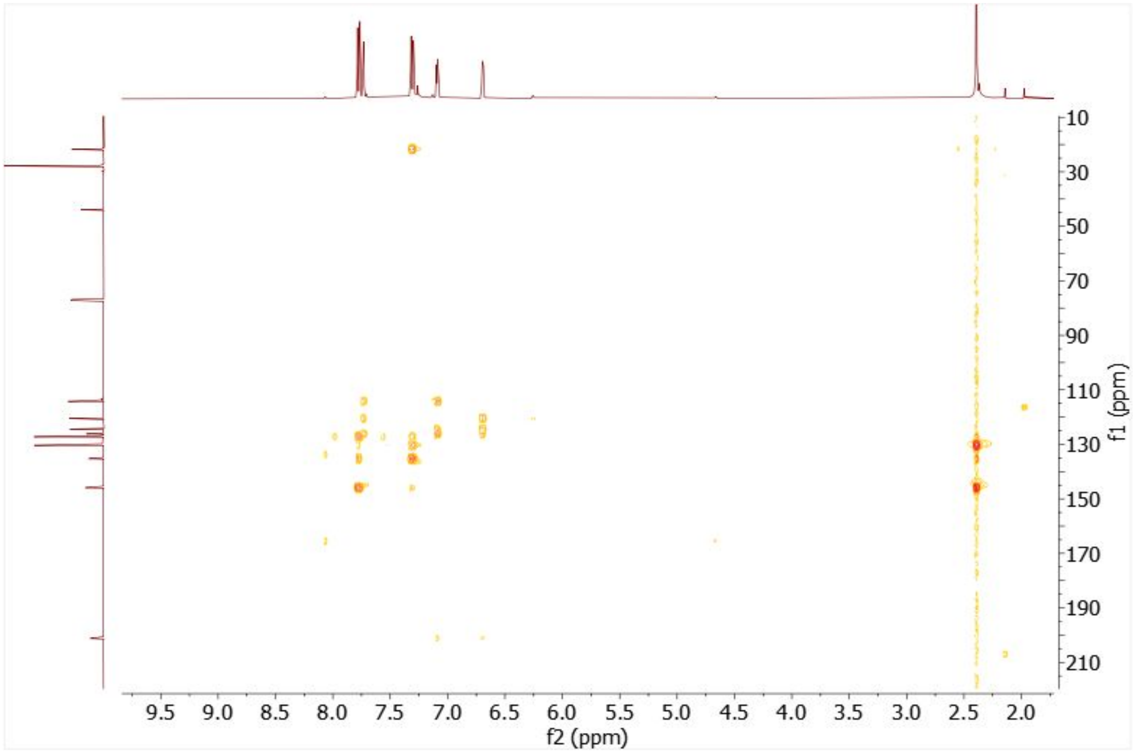

Fig S159: HMBC NMR spectrum of 2,2-dimethyl-1-(1-tosyl-1*H*-pyrrol-3-yl)propan-1-one (**31**) in $CDCl_3$

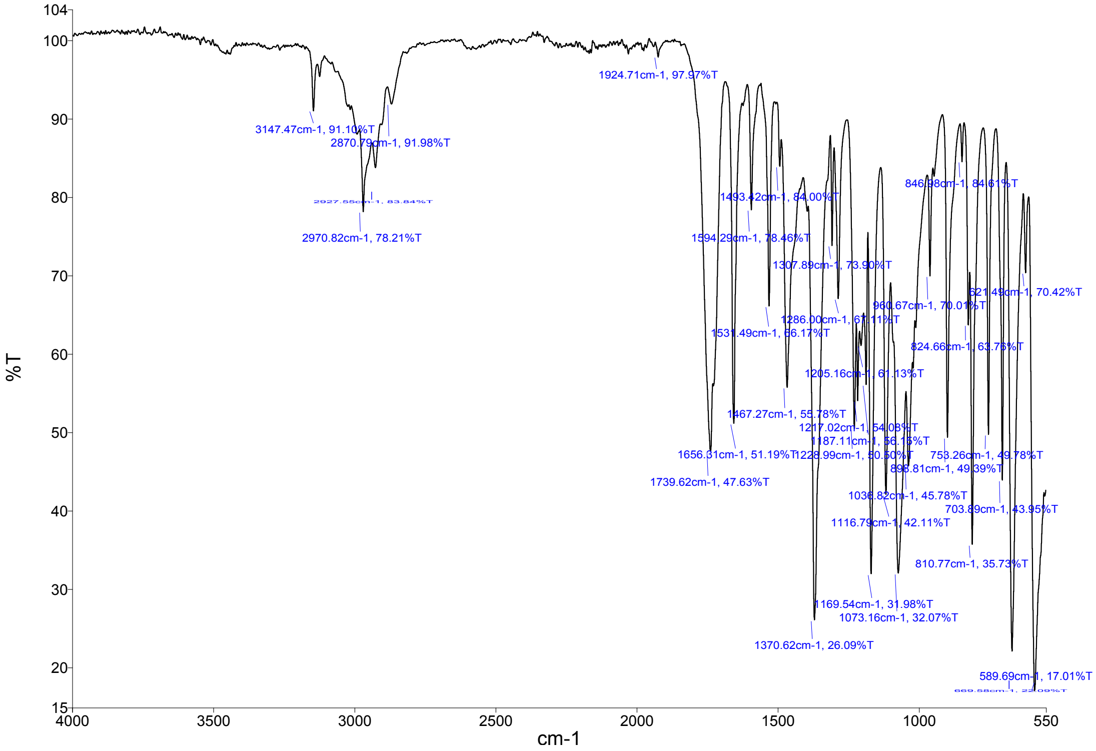

Fig S160: IR (neat) spectrum of 2,2-dimethyl-1-(1-tosyl-1*H*-pyrrol-3-yl)propan-1-one (**31**)

2,2-Dimethyl-1-(1*H*-pyrrol-3-yl)propan-1-one (32)

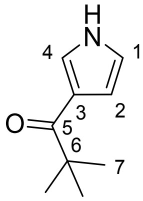

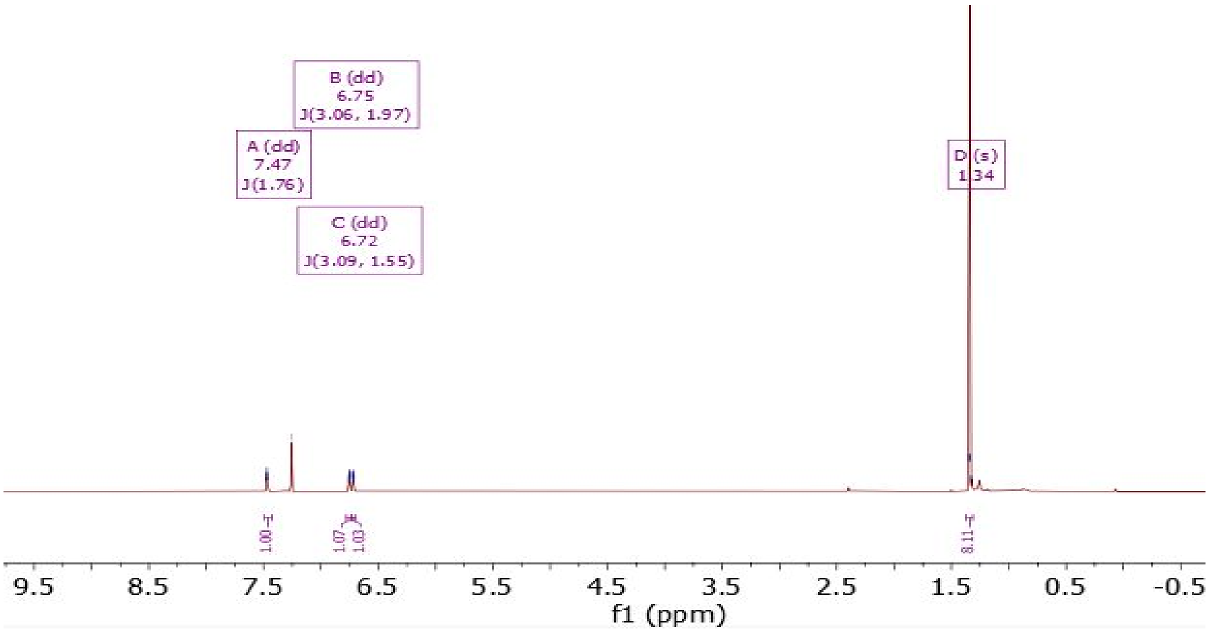

Fig S161: ^{1}H NMR (400 MHz) spectrum of 2,2-dimethyl-1-(1*H*-pyrrol-3-yl)propan-1-one (**32**) in $CDCl_3$.

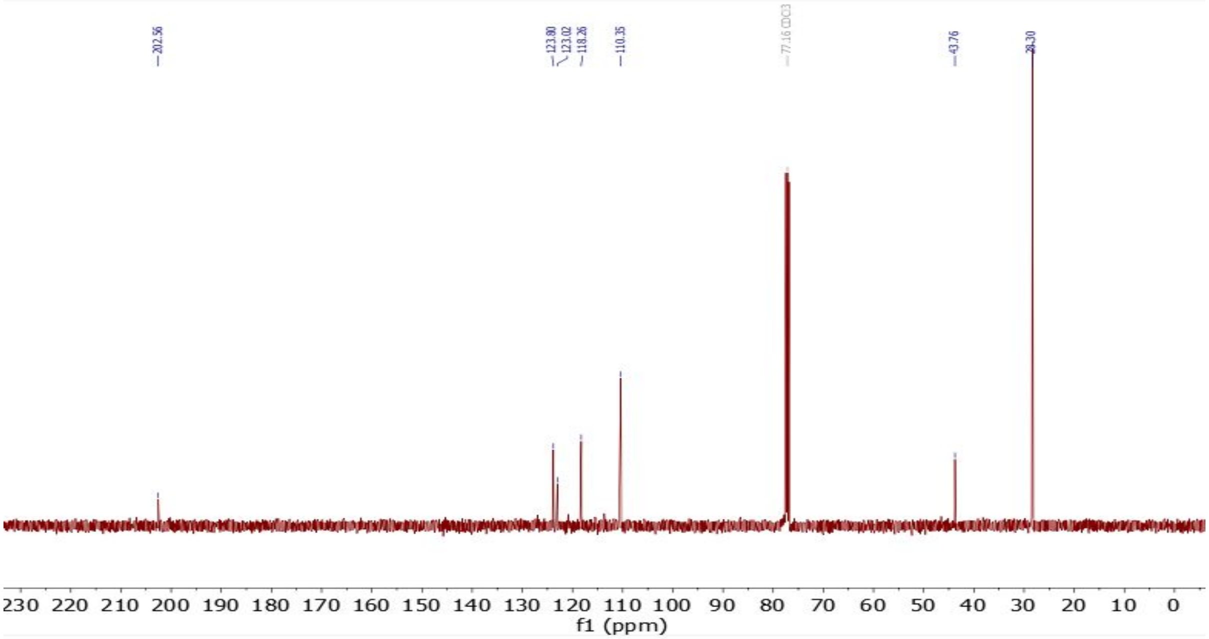

Fig S162: $^{13}C\{^{1}H\}$ NMR (101 MHz) spectrum of 2,2-dimethyl-1-(1*H*-pyrrol-3-yl)propan-1-one (**32**) in $CDCl_3$.

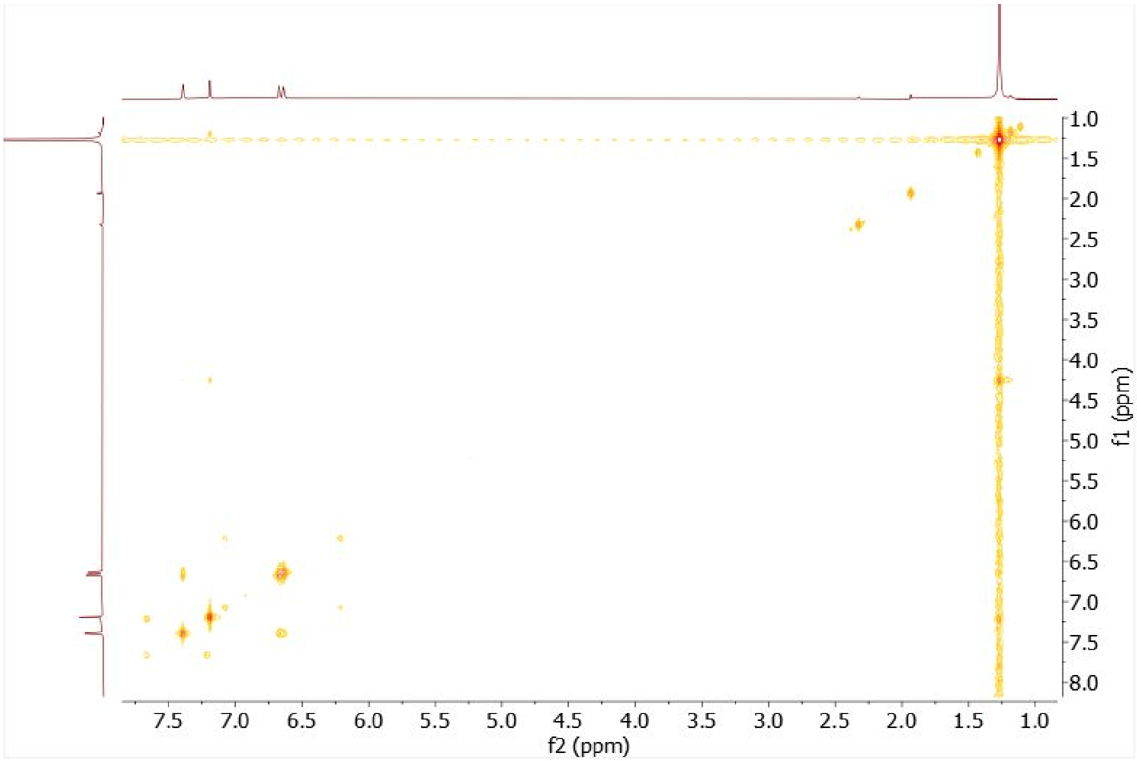

Fig S163: COSY NMR spectrum of 2,2-dimethyl-1-(1*H*-pyrrol-3-yl)propan-1-one (**32**) in $CDCl_3$.

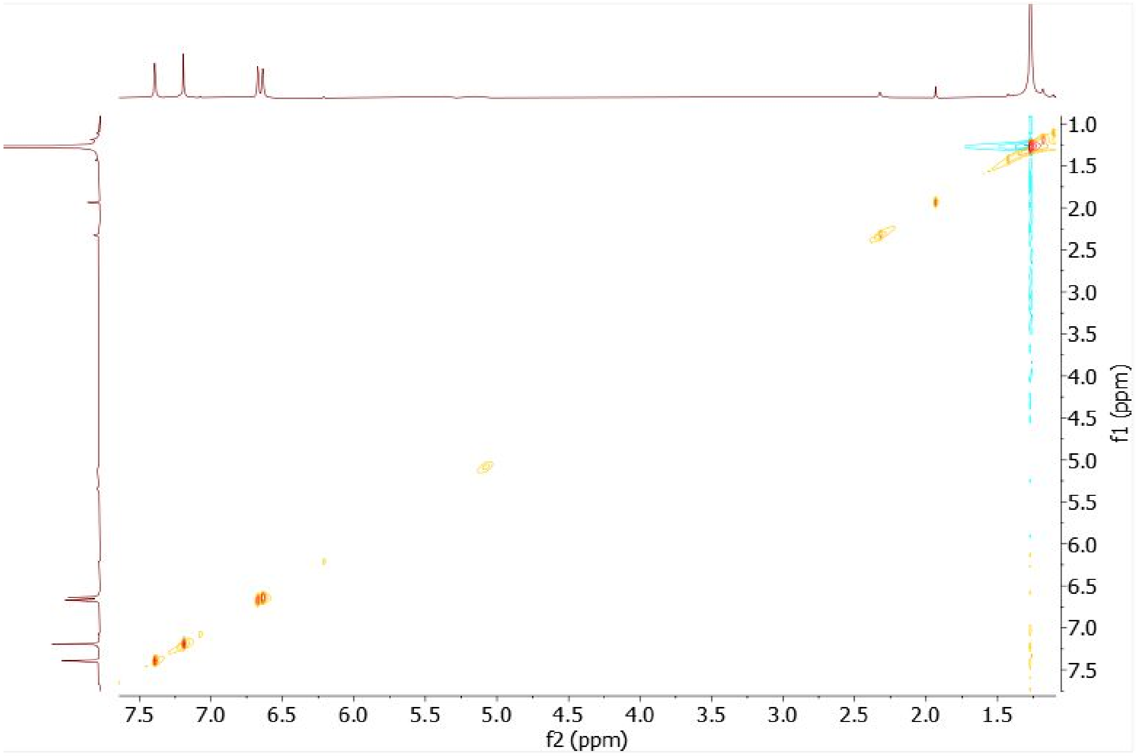

Fig S164: NOESY NMR spectrum of 2,2-dimethyl-1-(1*H*-pyrrol-3-yl)propan-1-one (**32**) in $CDCl_3$.

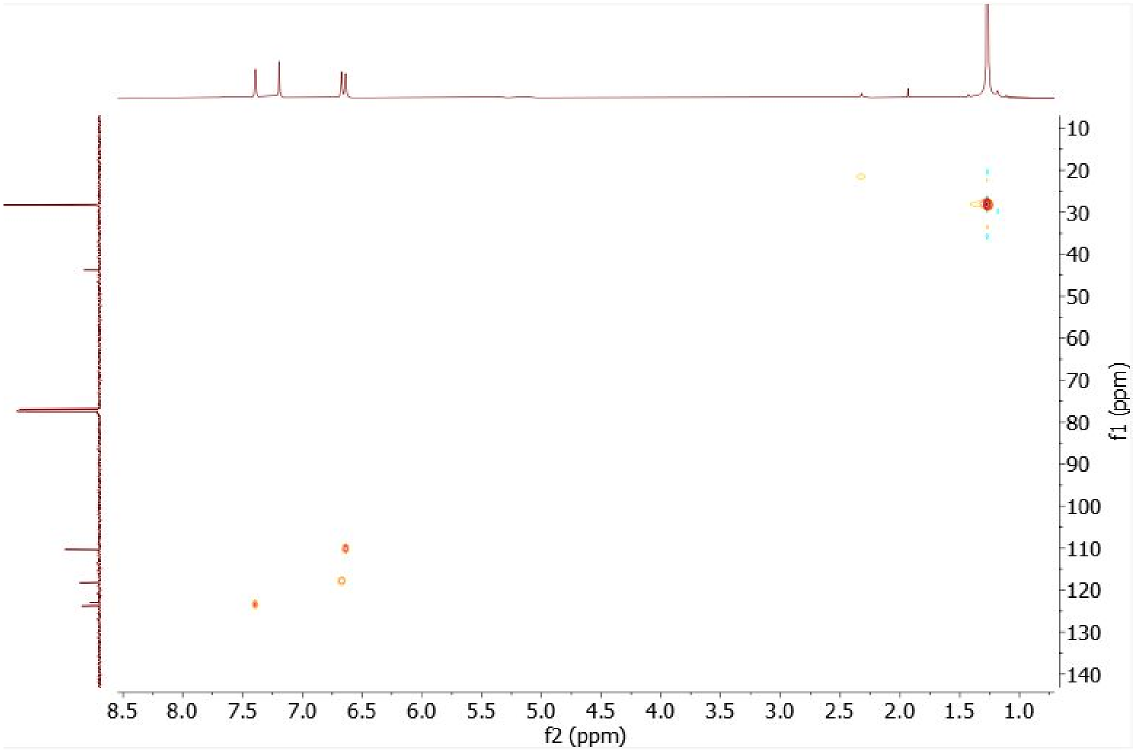

Fig S165: HSQC NMR spectrum of 2,2-dimethyl-1-(1*H*-pyrrol-3-yl)propan-1-one (**32**) in $CDCl_3$.

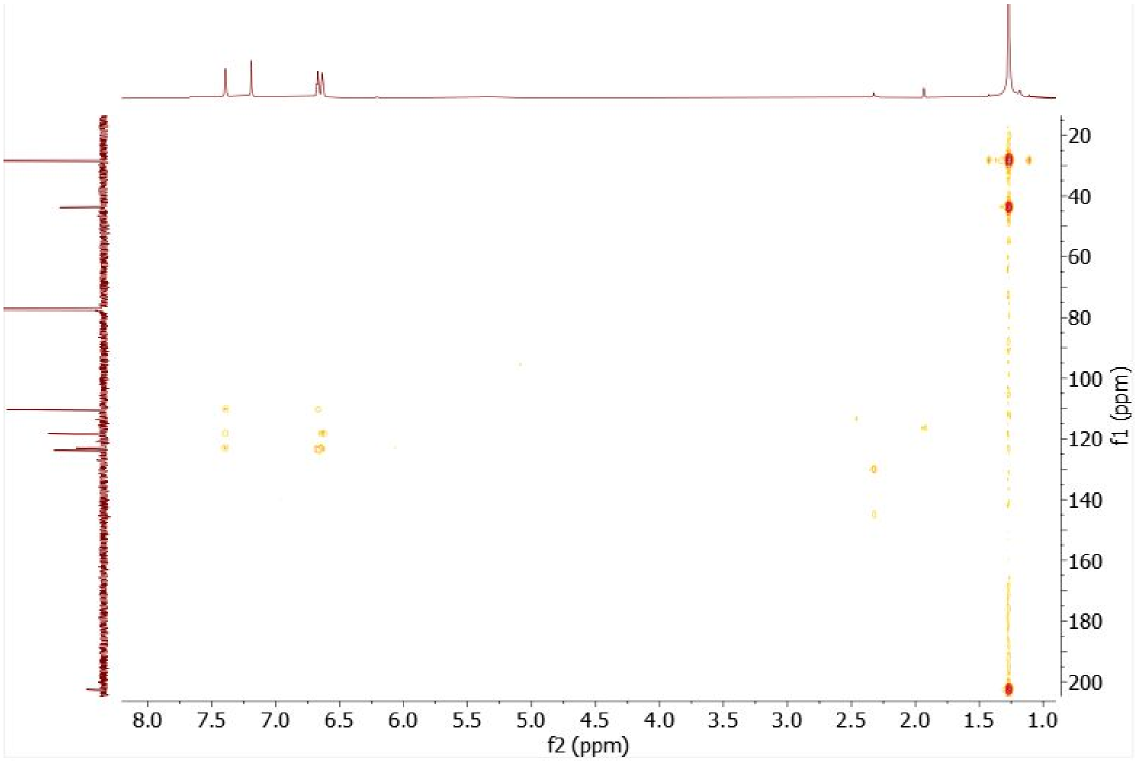

Fig S166: HMBC NMR spectrum of 2,2-dimethyl-1-(1*H*-pyrrol-3-yl)propan-1-one (**32**) in $CDCl_3$.

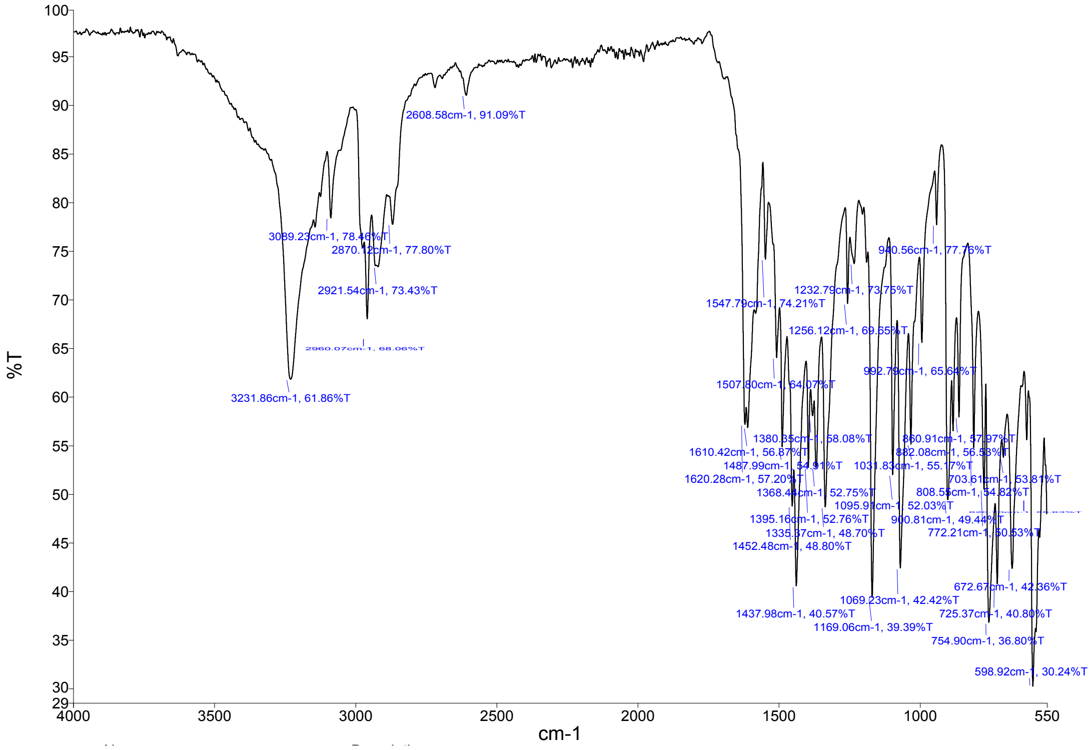

Fig S167: IR (neat) spectrum of 2,2-dimethyl-1-(1*H*-pyrrol-3-yl)propan-1-one (**32**)

2-(4-*iso*-Butylphenyl)-1-(1-tosyl-1*H*-pyrrol-3-yl)propan-1-one (33)

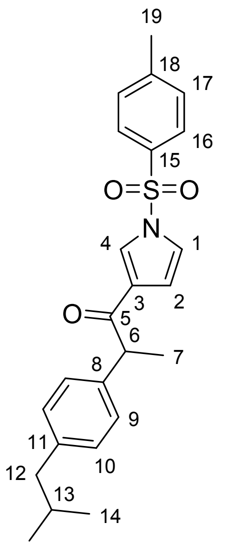

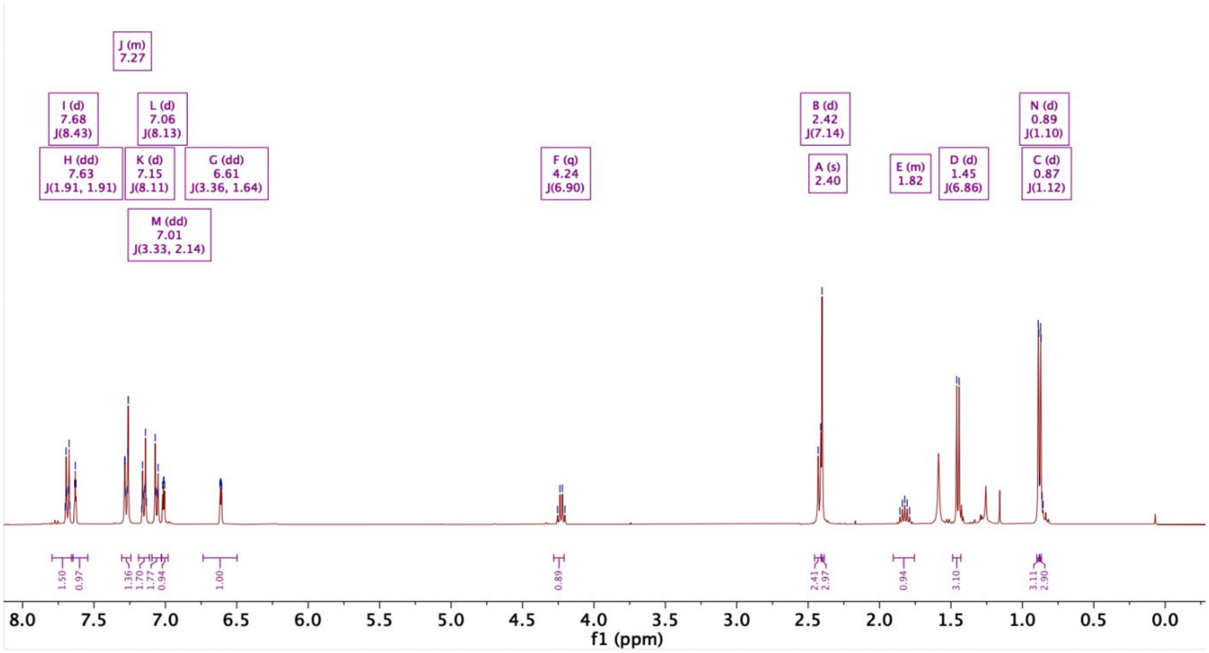

Fig S168: ^{1}H NMR (400 MHz) spectrum of 2-(4-*iso*-butylphenyl)-1-(1-tosyl-1*H*-pyrrol-3-yl)propan-1-one (**33**) in $CDCl_3$

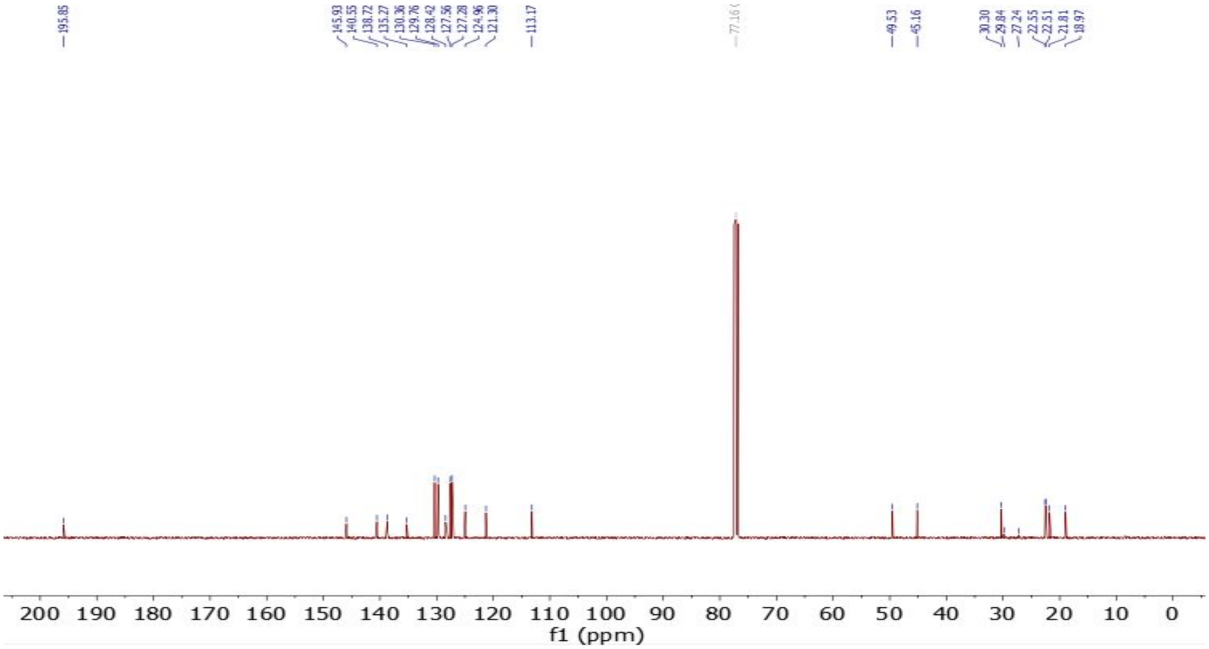

Fig S169: $^{13}C\{^{1}H\}$ NMR (101 MHz) spectrum of 2-(4-*iso*-butylphenyl)-1-(1-tosyl-1*H*-pyrrol-3-yl)propan-1-one (**33**) in $CDCl_3$

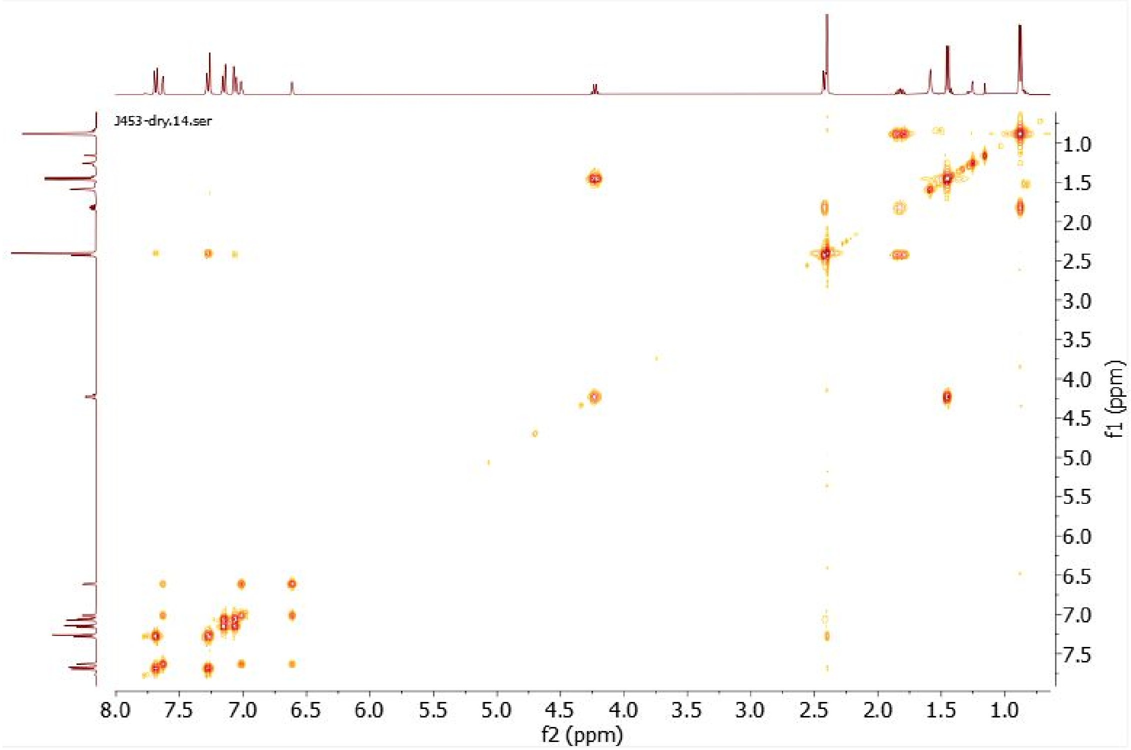

Fig S170: COSY NMR spectrum of 2-(4-*iso*-butylphenyl)-1-(1-tosyl-1*H*-pyrrol-3-yl)propan-1-one (**33**) in $CDCl_3$

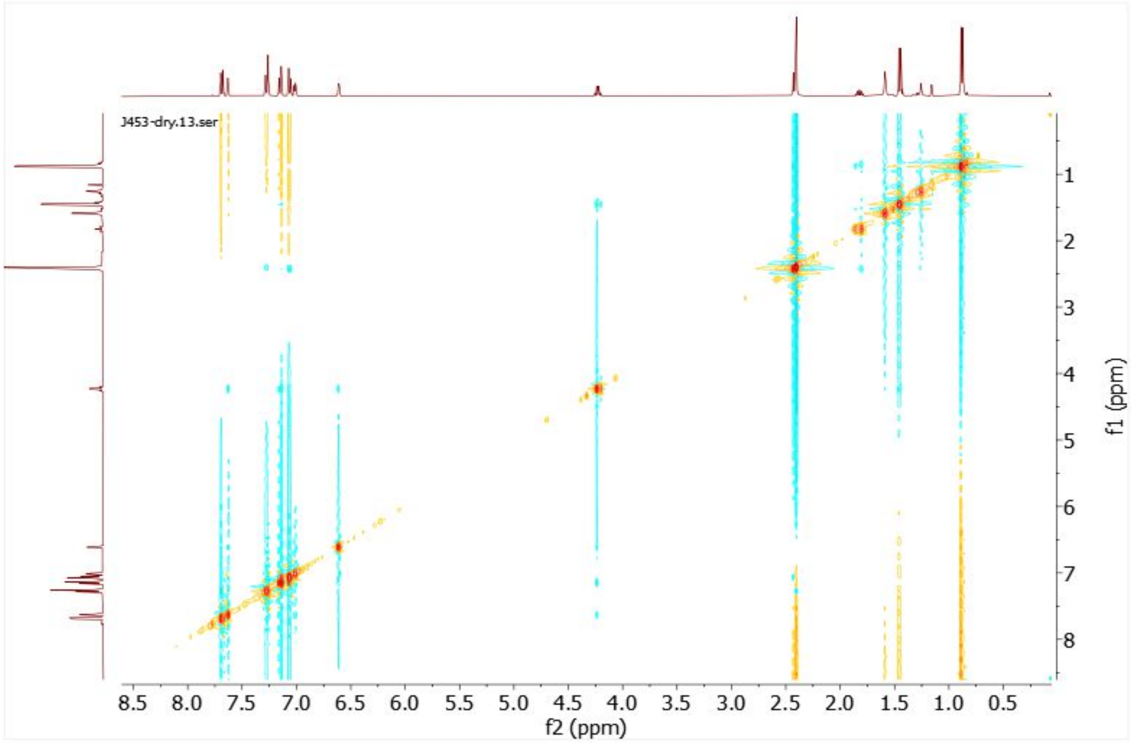

Fig S171: NOESY NMR spectrum of 2-(4-*iso*-butylphenyl)-1-(1-tosyl-1*H*-pyrrol-3-yl)propan-1-one (**33**) in $CDCl_3$

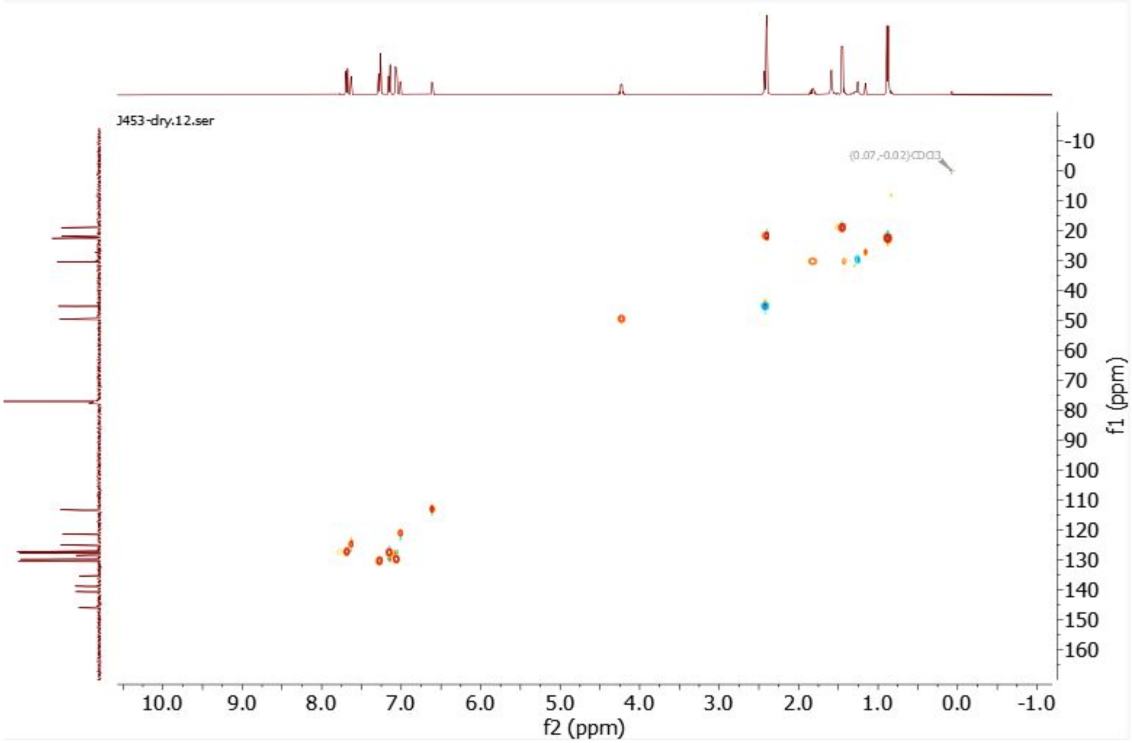

Fig S172: HSQC NMR spectrum of 2-(4-*iso*-butylphenyl)-1-(1-tosyl-1*H*-pyrrol-3-yl)propan-1-one (**33**) in $CDCl_3$

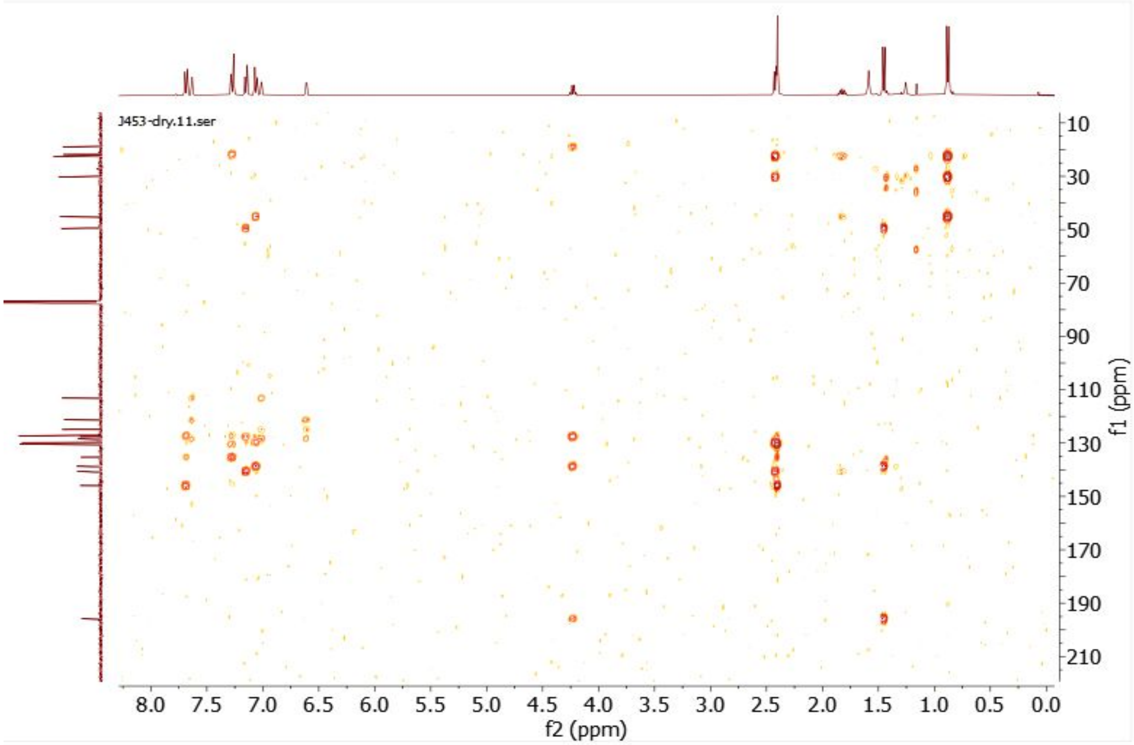

Fig S173: HMBC NMR spectrum of 2-(4-*iso*-butylphenyl)-1-(1-tosyl-1*H*-pyrrol-3-yl)propan-1-one (**33**) in $CDCl_3$

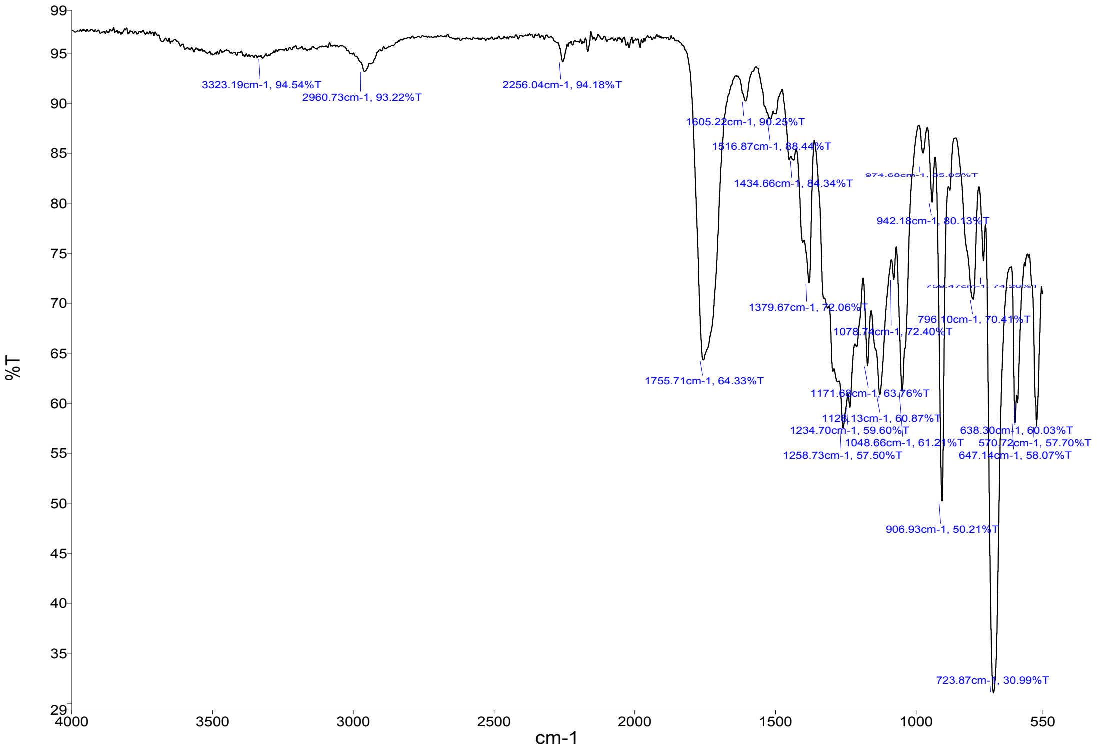

Fig S174: IR spectrum of of 2-(4-*iso*-butylphenyl)-1-(1-tosyl-1*H*-pyrrol-3-yl)propan-1-one (**33**)

2-(4-*iso*-Butylphenyl)-1-(1*H*-pyrrol-3-yl)propan-1-one (34)

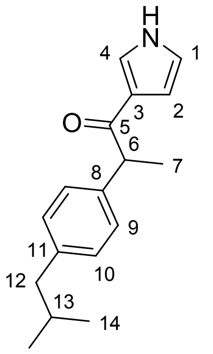

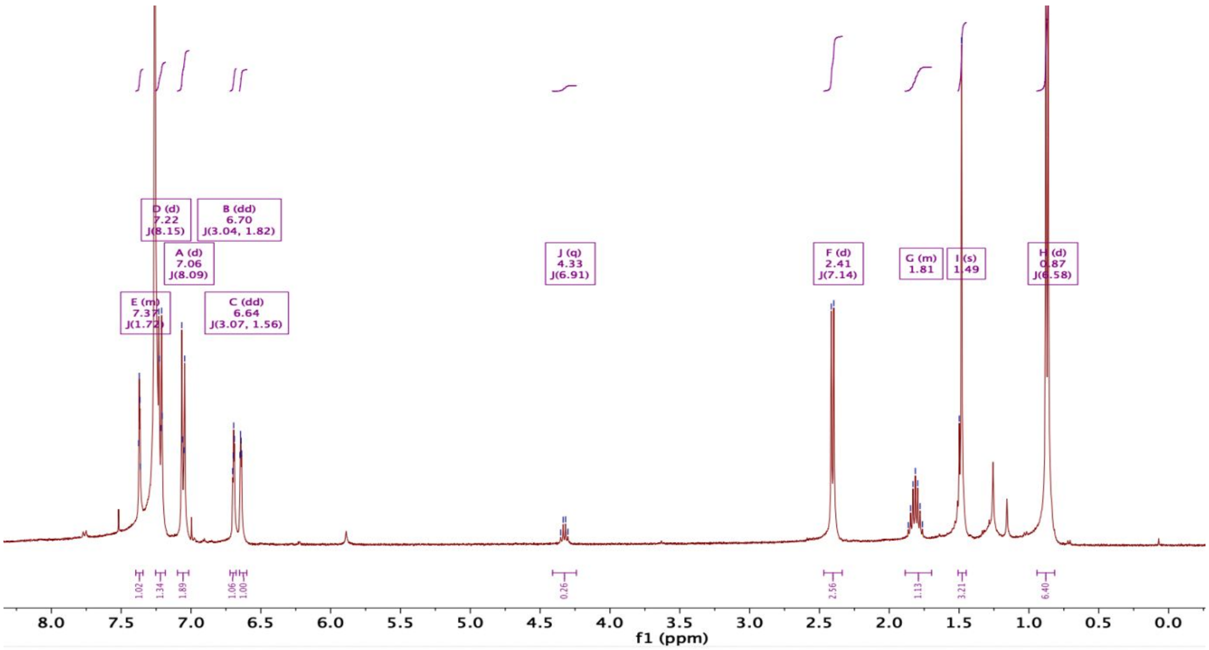

Fig S175: ^{1}H NMR (400 MHz) spectrum of 2-(4-*iso*-butylphenyl)-1-(1*H*-pyrrol-3-yl)propan-1-one (**34**) in $CDCl_3$

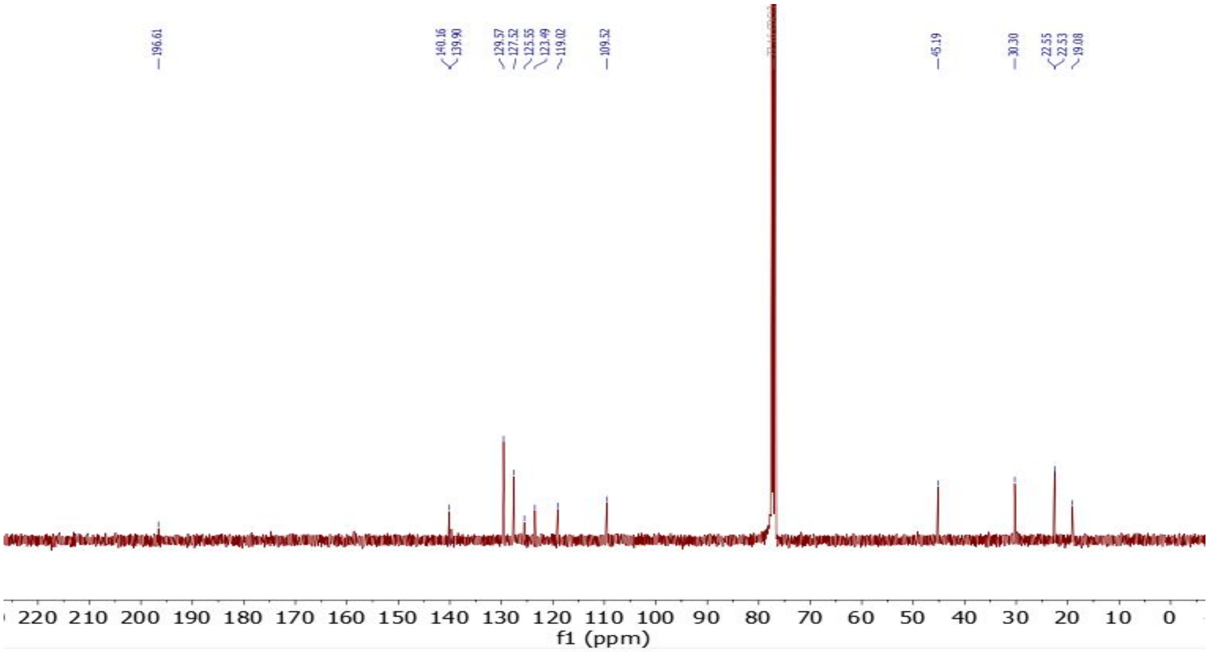

Fig S176: $^{13}C\{^{1}H\}$ NMR (101 MHz) spectrum of 2-(4-*iso*-butylphenyl)-1-(1*H*-pyrrol-3-yl)propan-1-one (**34**) in $CDCl_3$

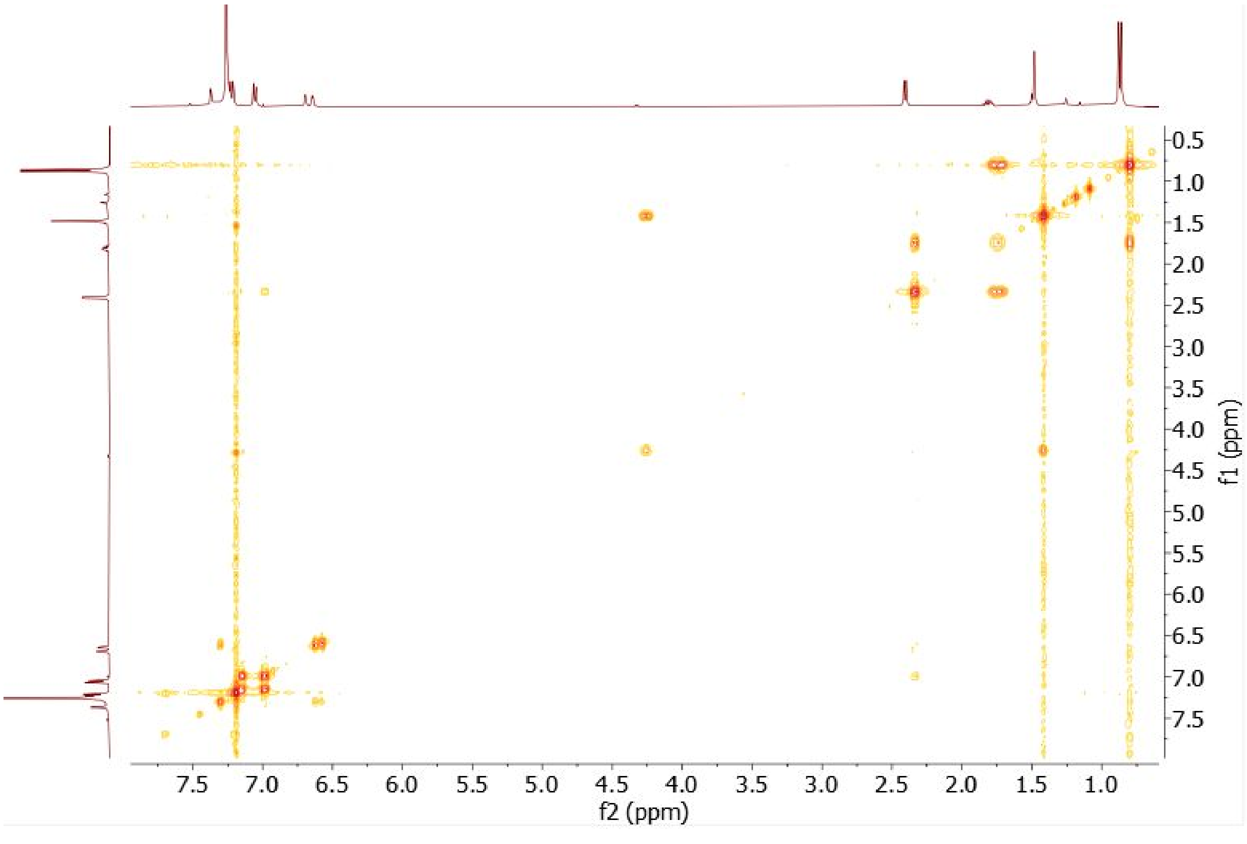

Fig S177: COSY NMR spectrum of 2-(4-*iso*-butylphenyl)-1-(1*H*-pyrrol-3-yl)propan-1-one (**34**) in $CDCl_3$

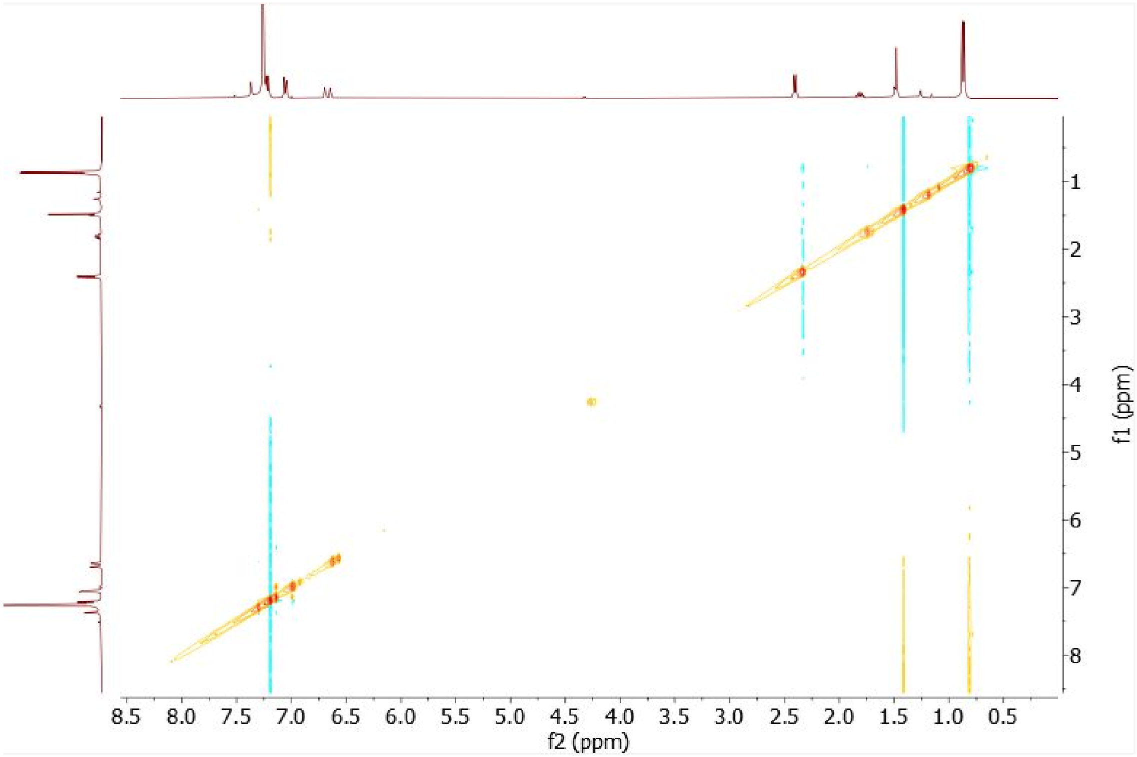

Fig S178: NOESY NMR spectrum of 2-(4-*iso*-butylphenyl)-1-(1*H*-pyrrol-3-yl)propan-1-one (**34**) in $CDCl_3$

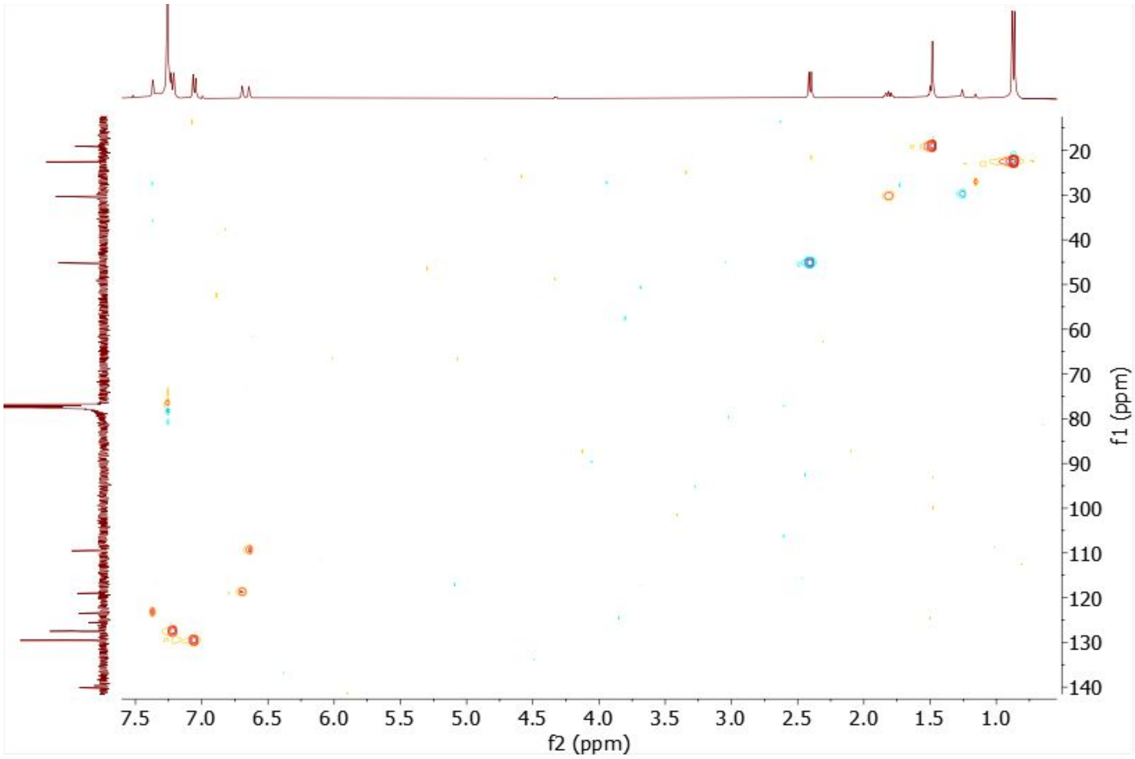

Fig S179: HSQC NMR spectrum of 2-(4-*iso*-butylphenyl)-1-(1*H*-pyrrol-3-yl)propan-1-one (**34**) in $CDCl_3$

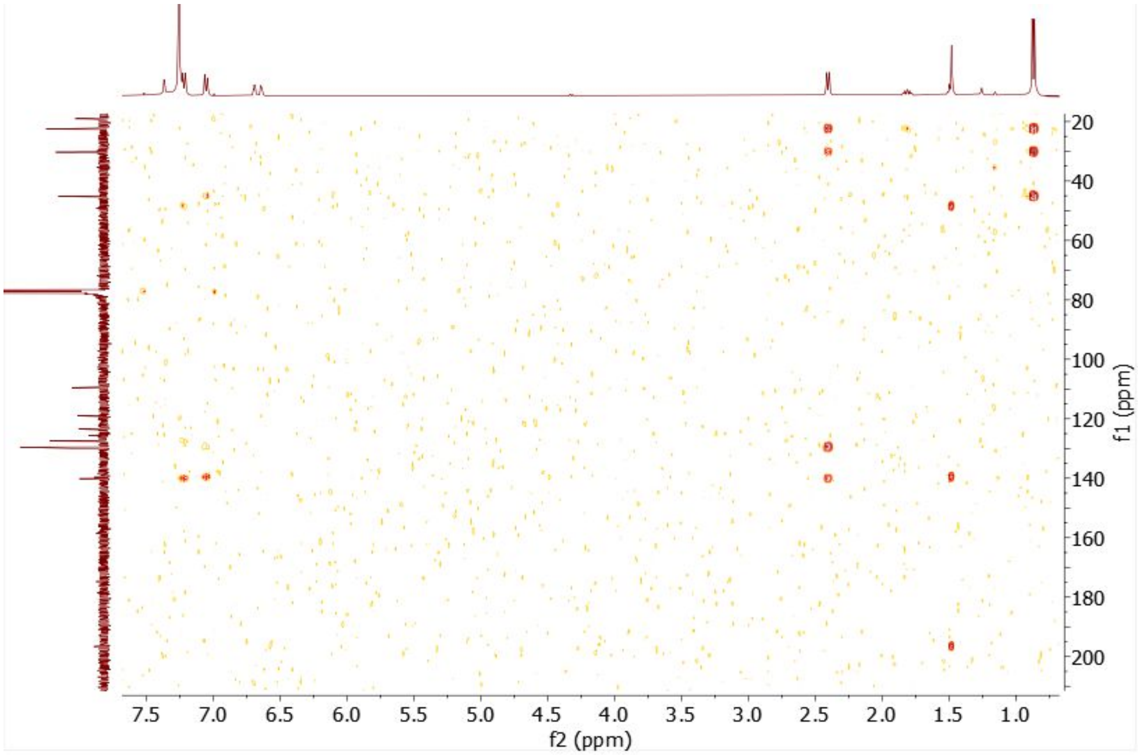

Fig S180: HMBC NMR spectrum of 2-(4-*iso*-butylphenyl)-1-(1*H*-pyrrol-3-yl)propan-1-one (**34**) in $CDCl_3$

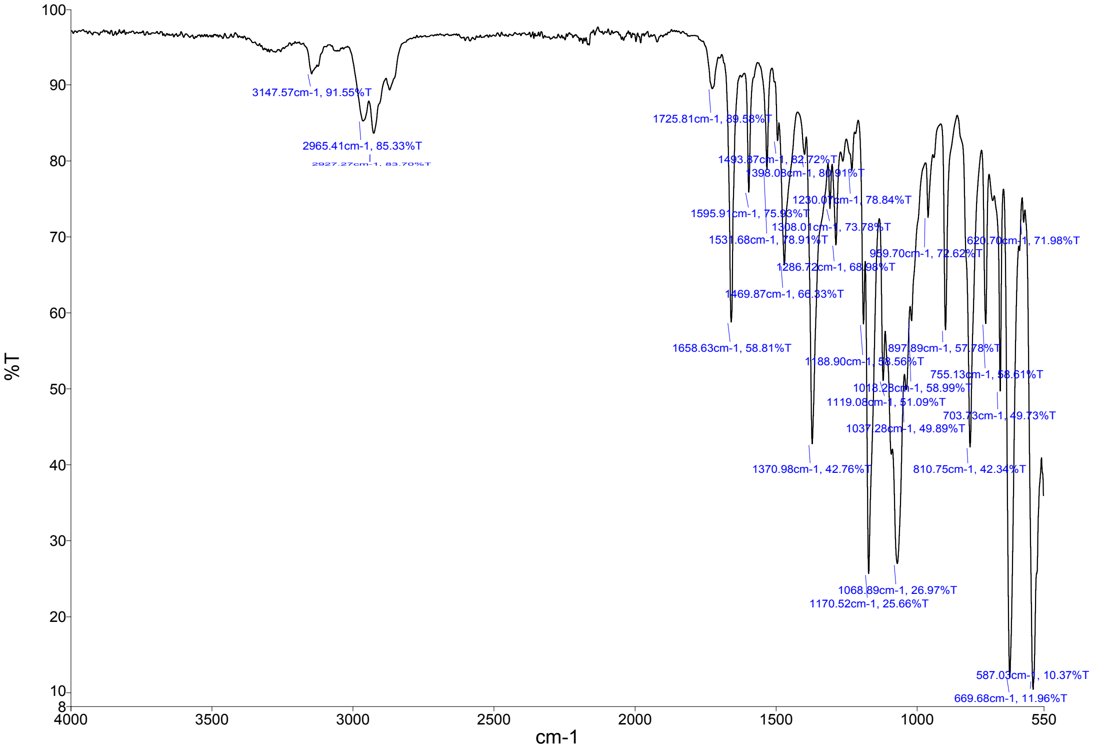

Fig S181: IR (neat) spectrum of 2-(4-*iso*-butylphenyl)-1-(1*H*-pyrrol-3-yl)propan-1-one (**34**)

2,2-Diphenyl-1-(1-tosyl-1*H*-pyrrol-3-yl)ethan-1-one (35)

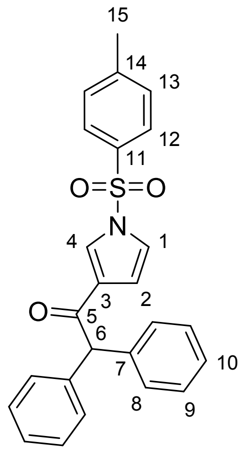

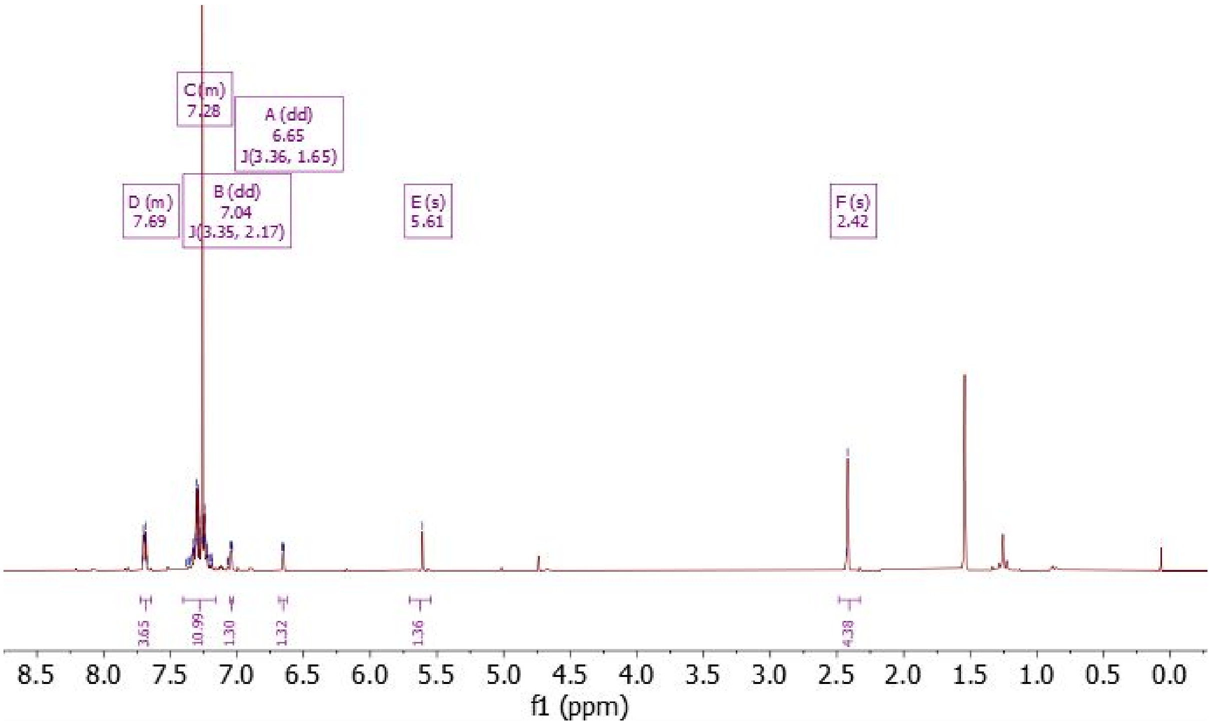

Fig S182: ^{1}H NMR (400 MHz) spectrum of 2,2-diphenyl-1-(1-tosyl-1*H*-pyrrol-3-yl)ethan-1-one (**35**) in $CDCl_3$

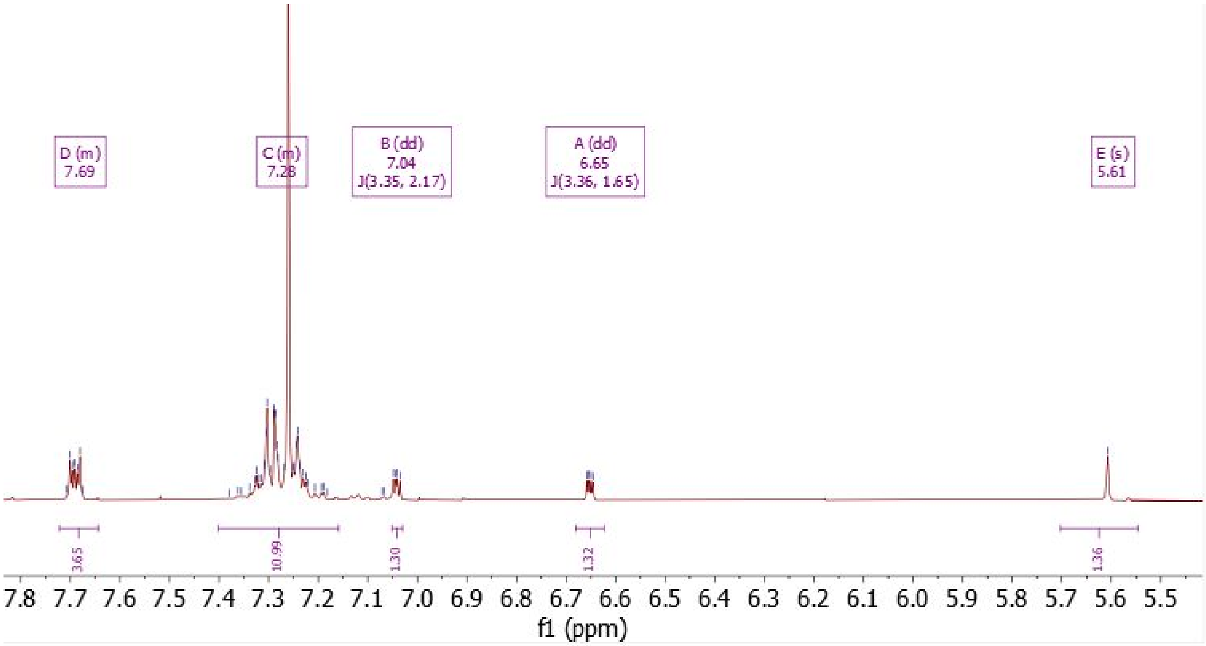

Fig S183: Zoomed ^{1}H NMR (400 MHz) spectrum of 2,2-diphenyl-1-(1-tosyl-1*H*-pyrrol-3-yl)ethan-1-one (**35**) in $CDCl_3$

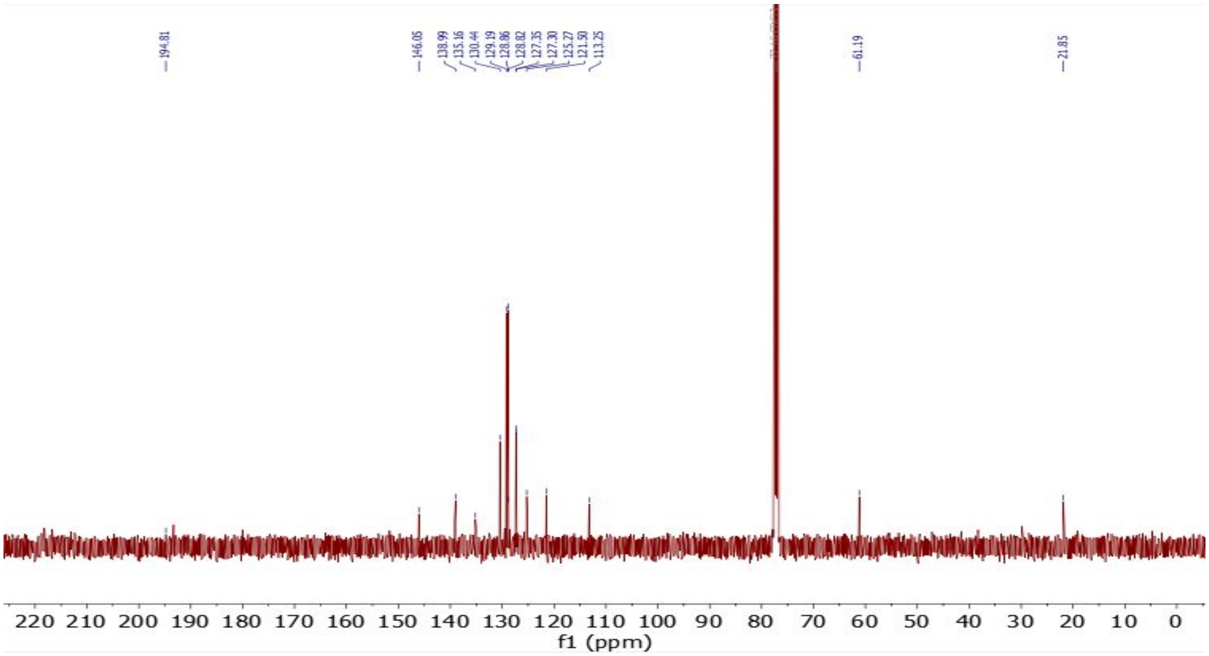

Fig S184: $^{13}C\{^{1}H\}$ NMR (101 MHz) spectrum of 2,2-diphenyl-1-(1-tosyl-1*H*-pyrrol-3-yl)ethan-1-one (**35**) in $CDCl_3$

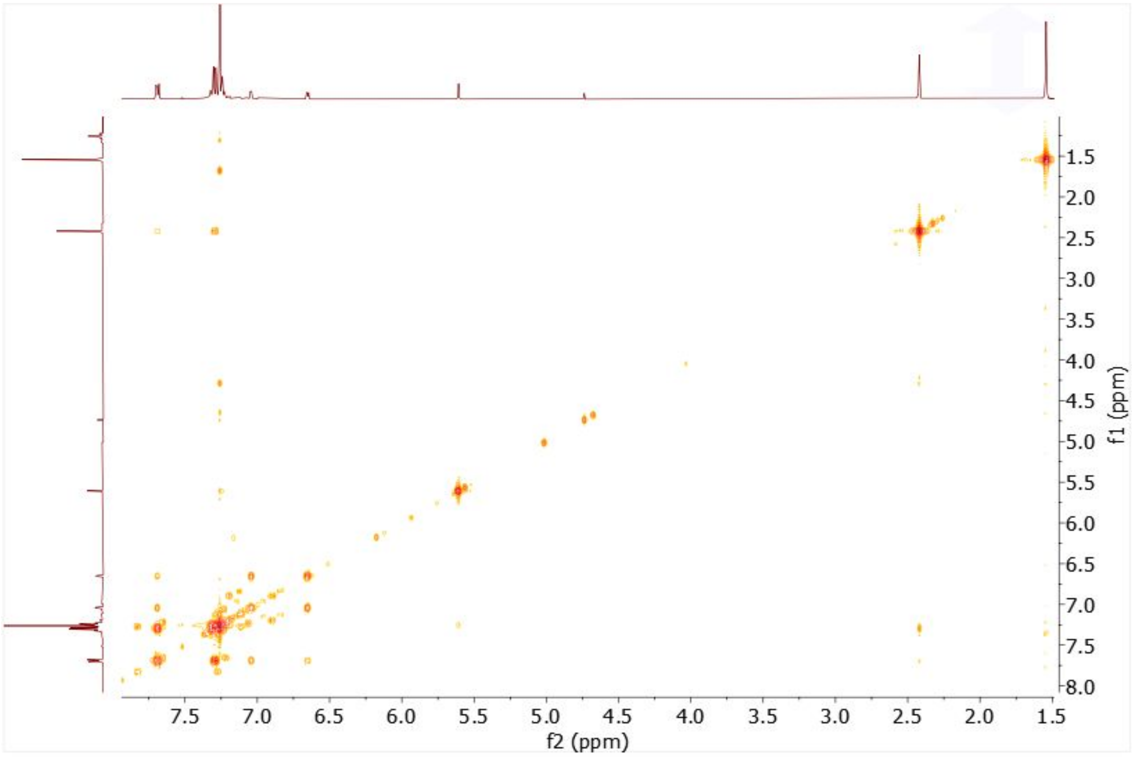

Fig S185: COSY NMR spectrum of 2,2-diphenyl-1-(1-tosyl-1*H*-pyrrol-3-yl)ethan-1-one (**35**) in $CDCl_3$

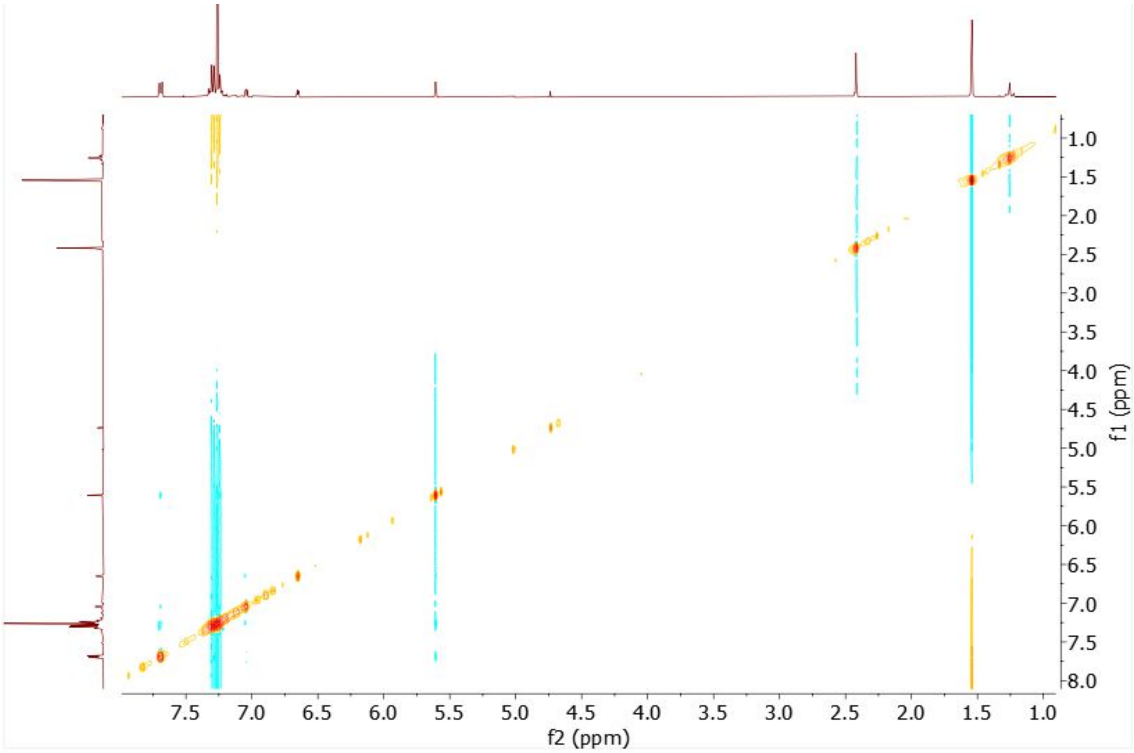

Fig S186: NOESY NMR spectrum of 2,2-diphenyl-1-(1-tosyl-1*H*-pyrrol-3-yl)ethan-1-one (**35**) in $CDCl_3$

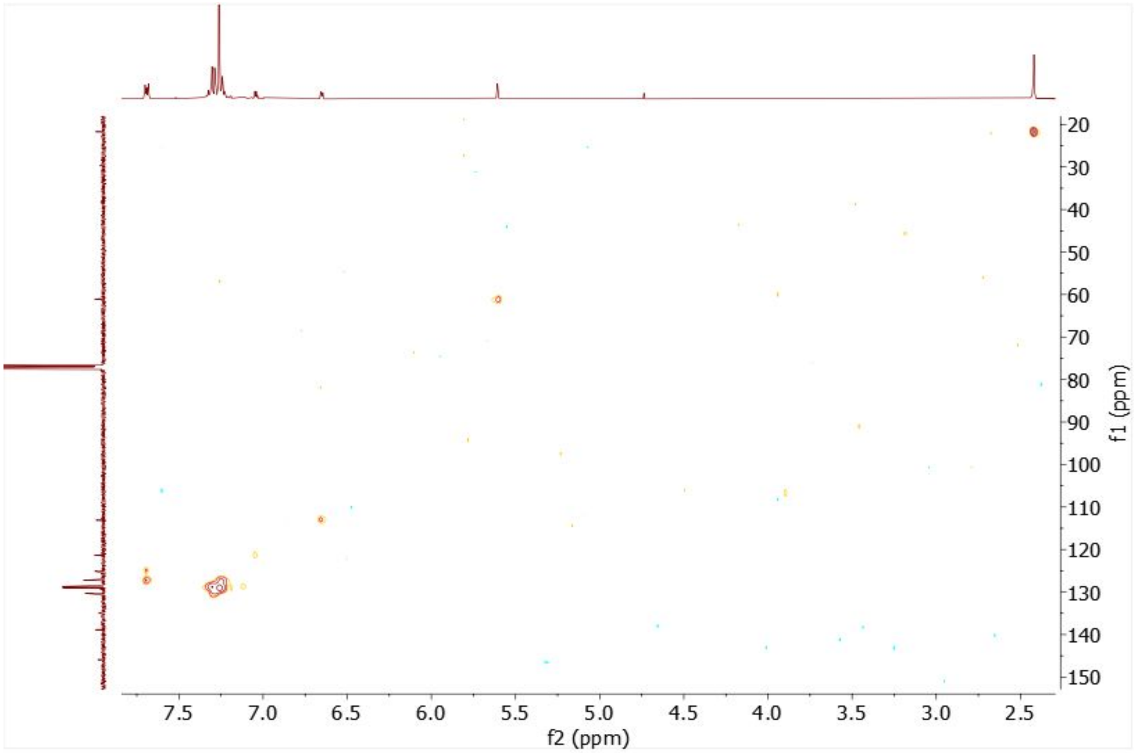

Fig S187: HSQC NMR spectrum of 2,2-diphenyl-1-(1-tosyl-1*H*-pyrrol-3-yl)ethan-1-one (**35**) in $CDCl_3$

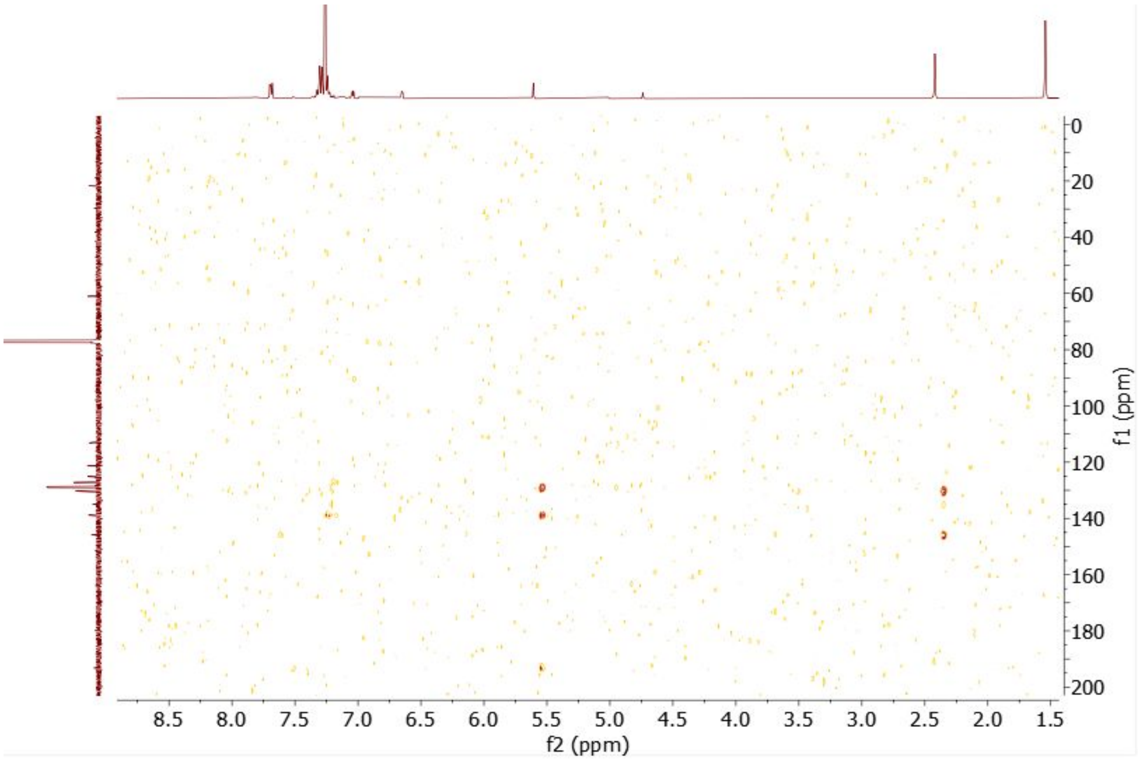

Fig S188: HMBC NMR spectrum of 2,2-diphenyl-1-(1-tosyl-1*H*-pyrrol-3-yl)ethan-1-one (**35**) in $CDCl_3$

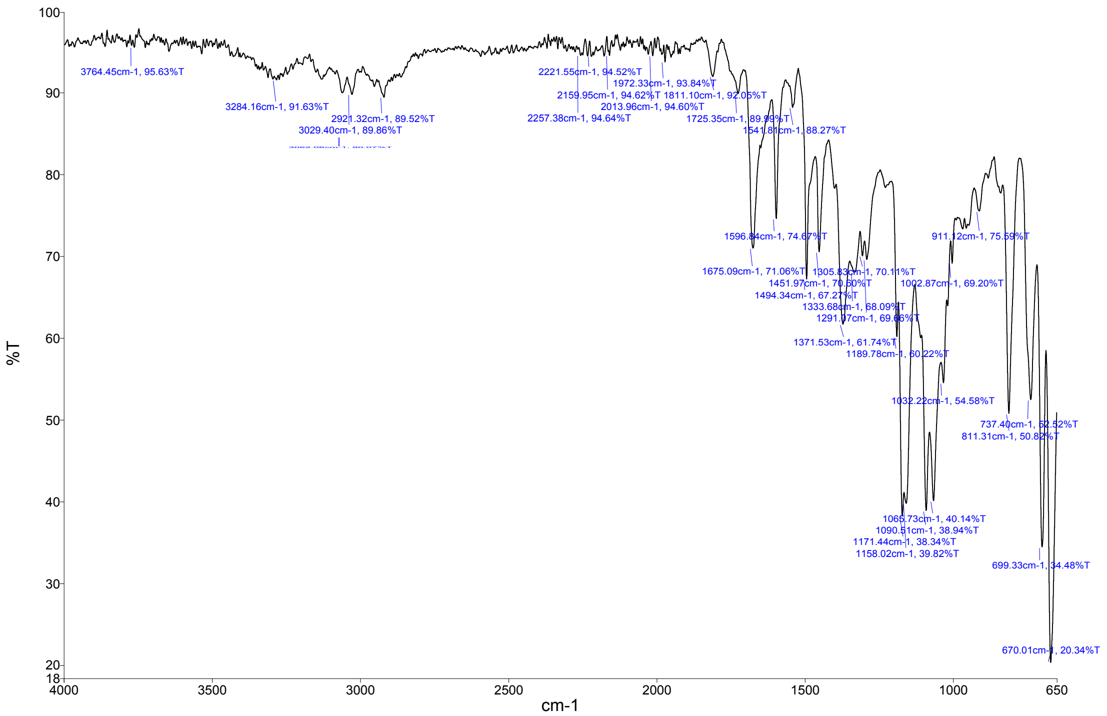

Fig S189: IR (neat) spectrum of 2,2-diphenyl-1-(1-tosyl-1*H*-pyrrol-3-yl)ethan-1-one (**35**)

2,2-Diphenyl-1-(1*H*-pyrrol-3-yl)ethan-1-one (36)

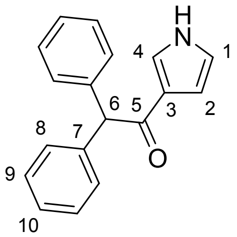

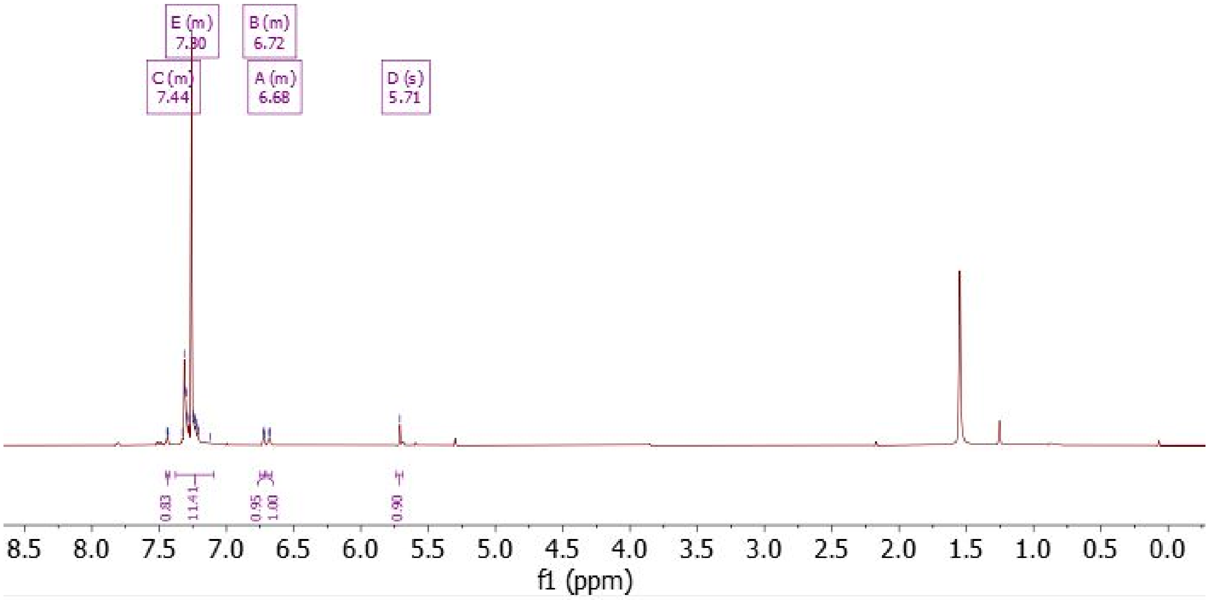

Fig S190: ^{1}H NMR (400 MHz) spectrum of 2,2-diphenyl-1-(1*H*-pyrrol-3-yl)ethan-1-one (**36**) in $CDCl_3$

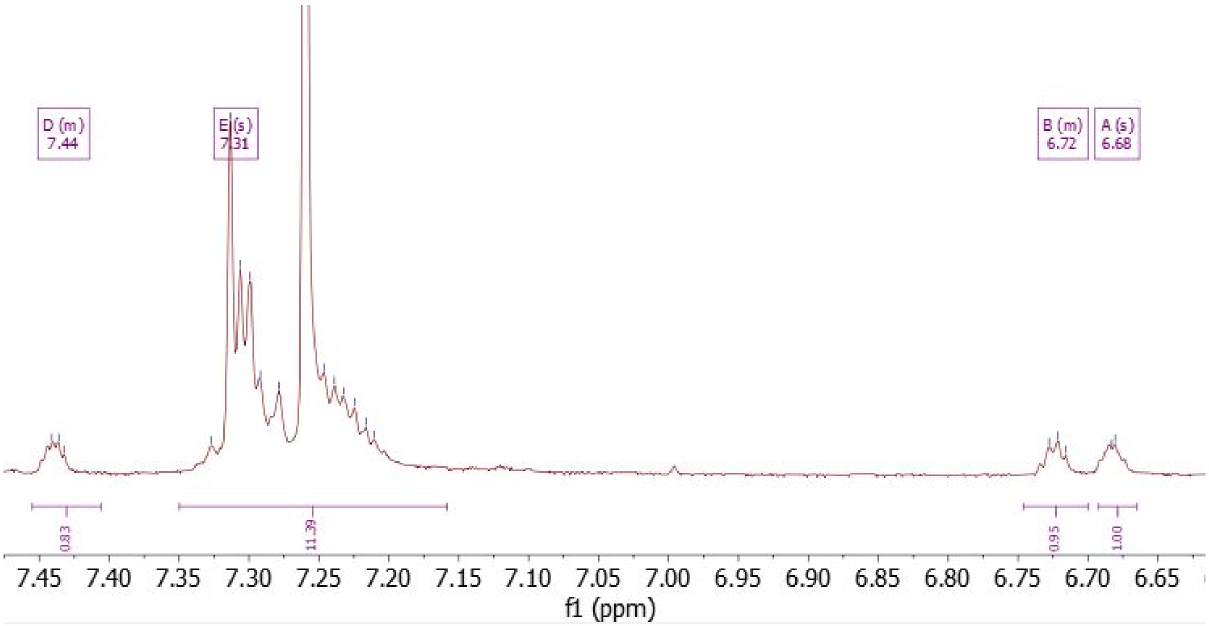

Fig S191: Zoomed ^{1}H NMR (400 MHz) spectrum of 2,2-diphenyl-1-(1*H*-pyrrol-3-yl)ethan-1-one (**36**) in $CDCl_3$

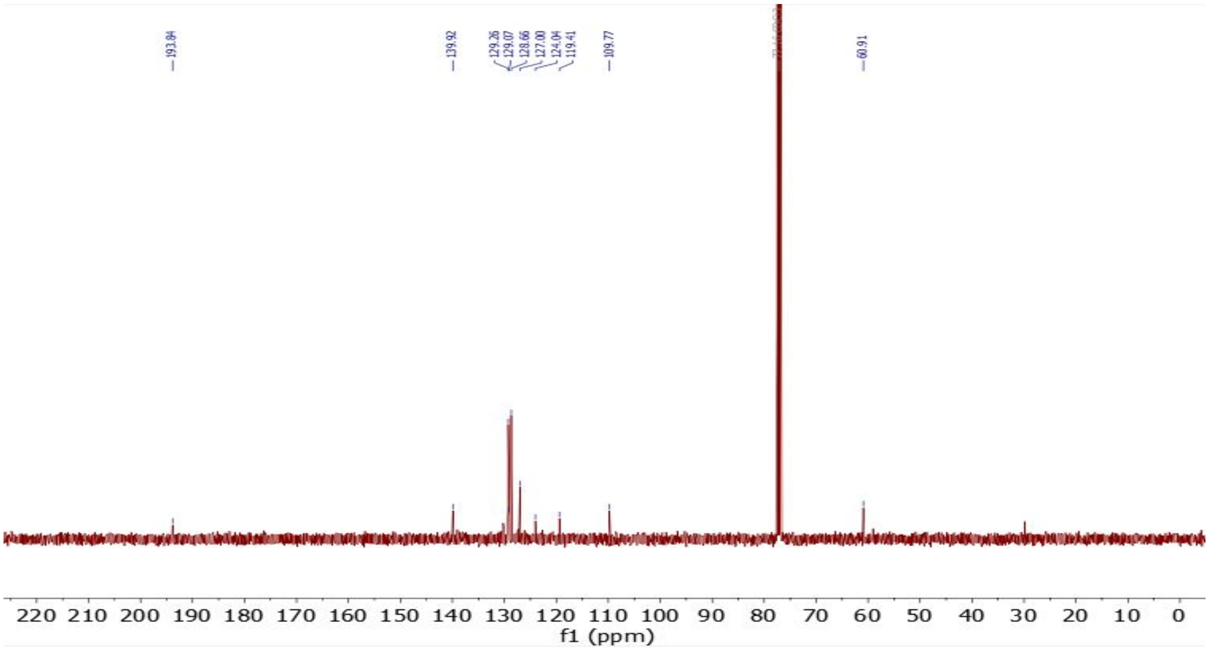

Fig S192: $^{13}C\{^{1}H\}$ NMR (101 MHz) spectrum of 2,2-diphenyl-1-(1*H*-pyrrol-3-yl)ethan-1-one (**36**) in $CDCl_3$

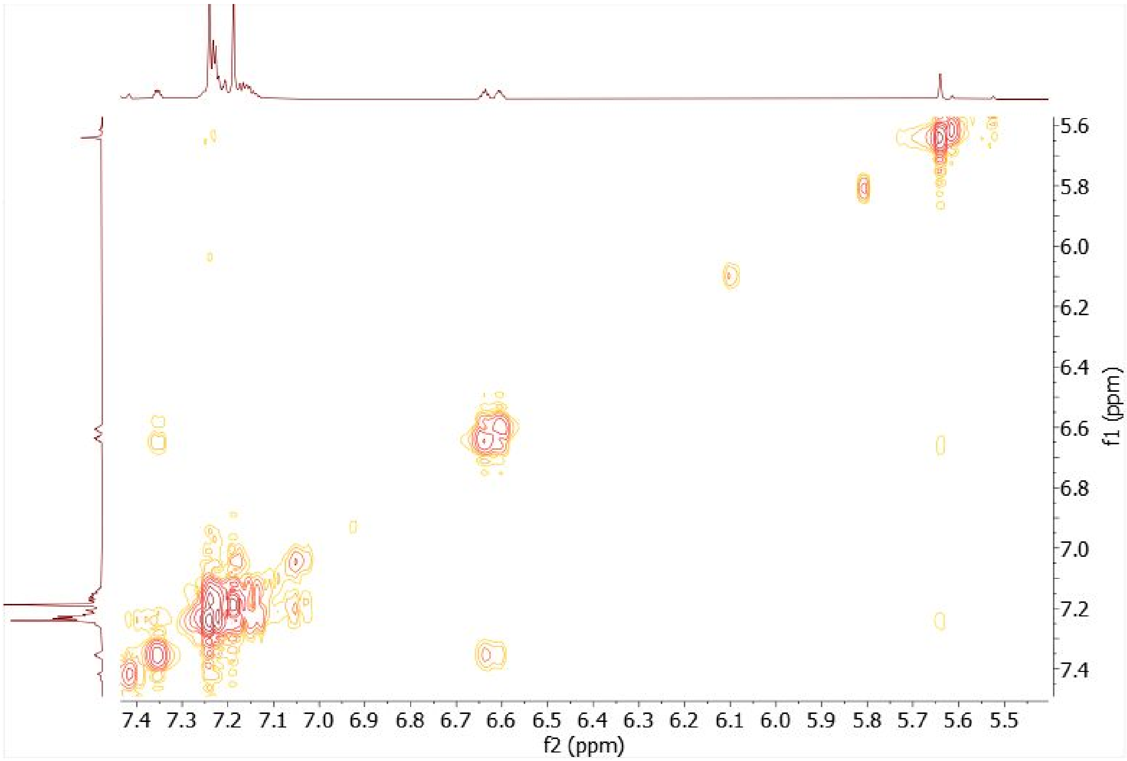

Fig S193: COSY NMR spectrum of 2,2-diphenyl-1-(1*H*-pyrrol-3-yl)ethan-1-one (**36**) in $CDCl_3$

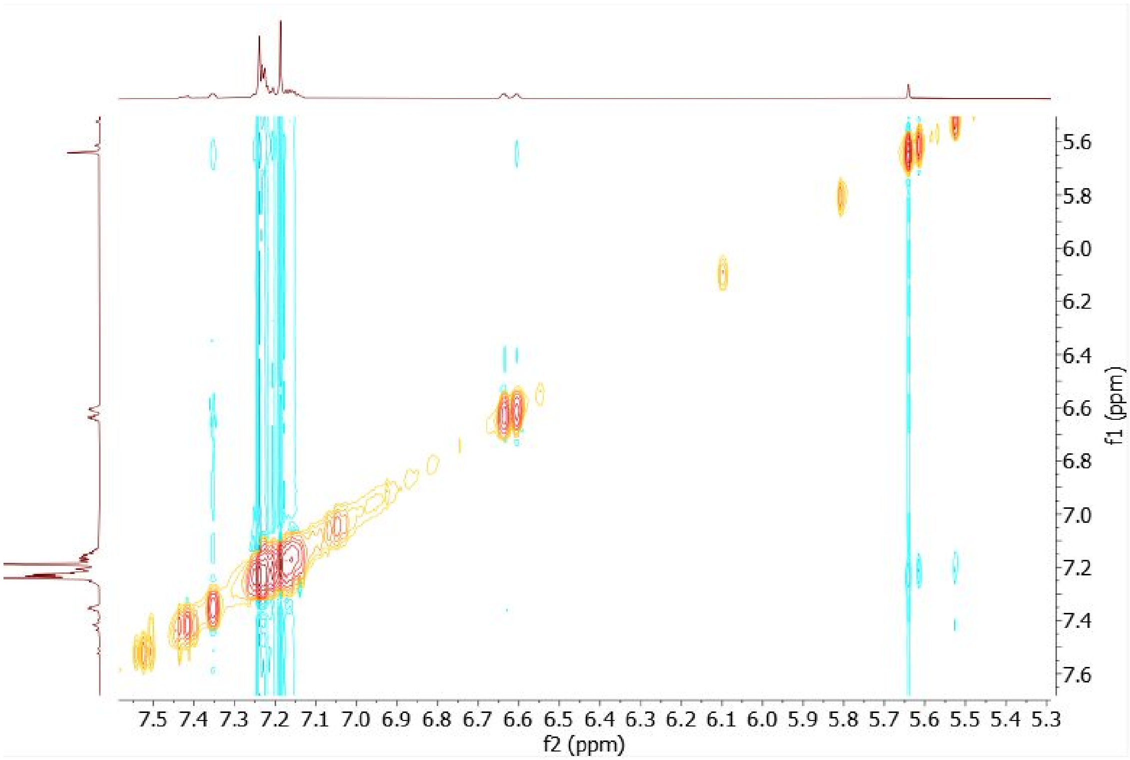

Fig S194: NOESY NMR spectrum of 2,2-diphenyl-1-(1*H*-pyrrol-3-yl)ethan-1-one (**36**) in $CDCl_3$

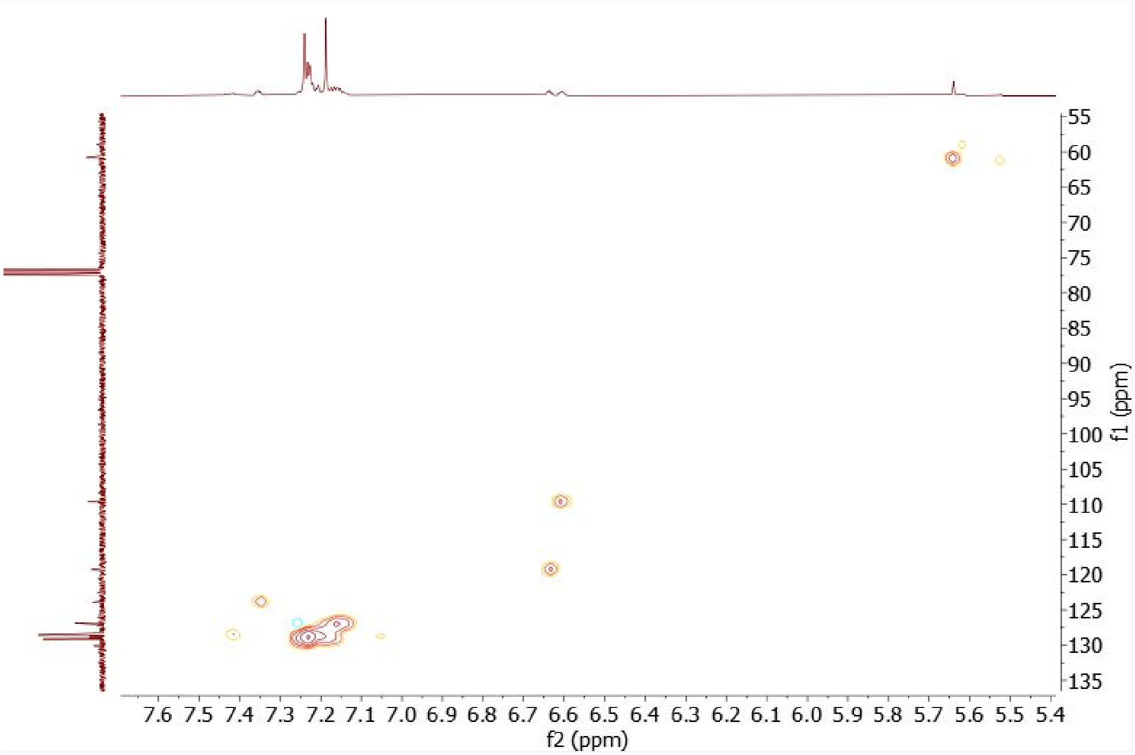

Fig S195: HSQC NMR spectrum of 2,2-diphenyl-1-(1*H*-pyrrol-3-yl)ethan-1-one (**36**) in $CDCl_3$

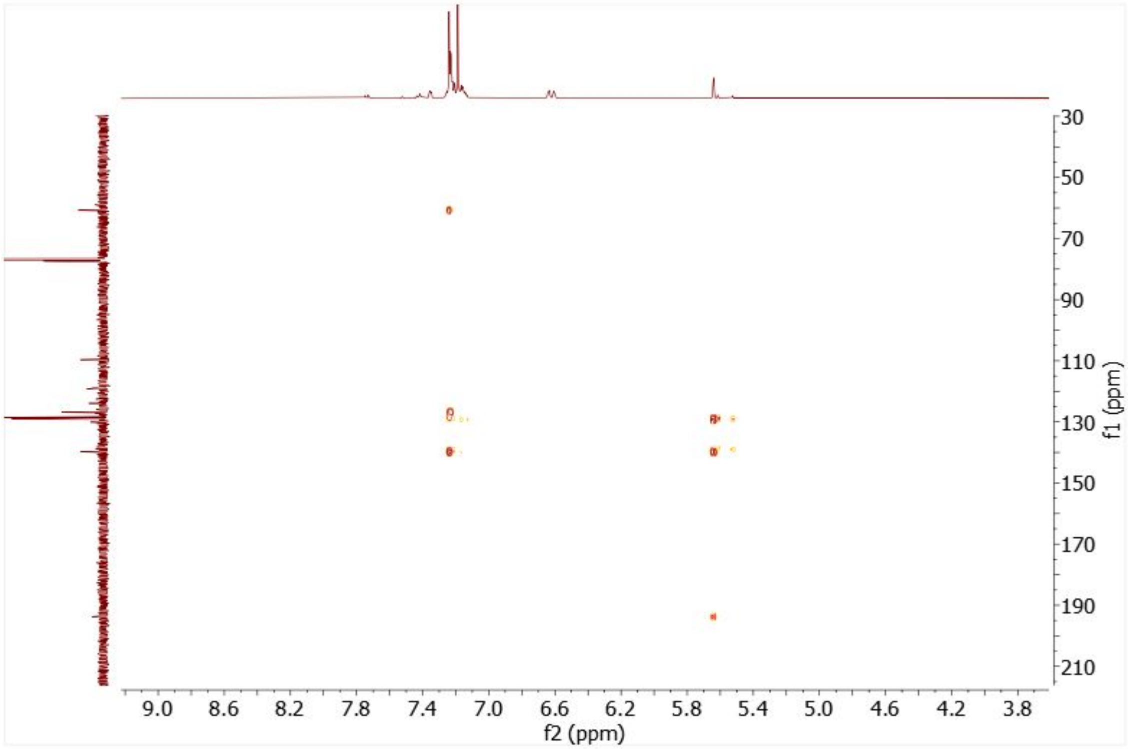

Fig S196: HMBC NMR spectrum of 2,2-diphenyl-1-(1*H*-pyrrol-3-yl)ethan-1-one (**36**) in $CDCl_3$

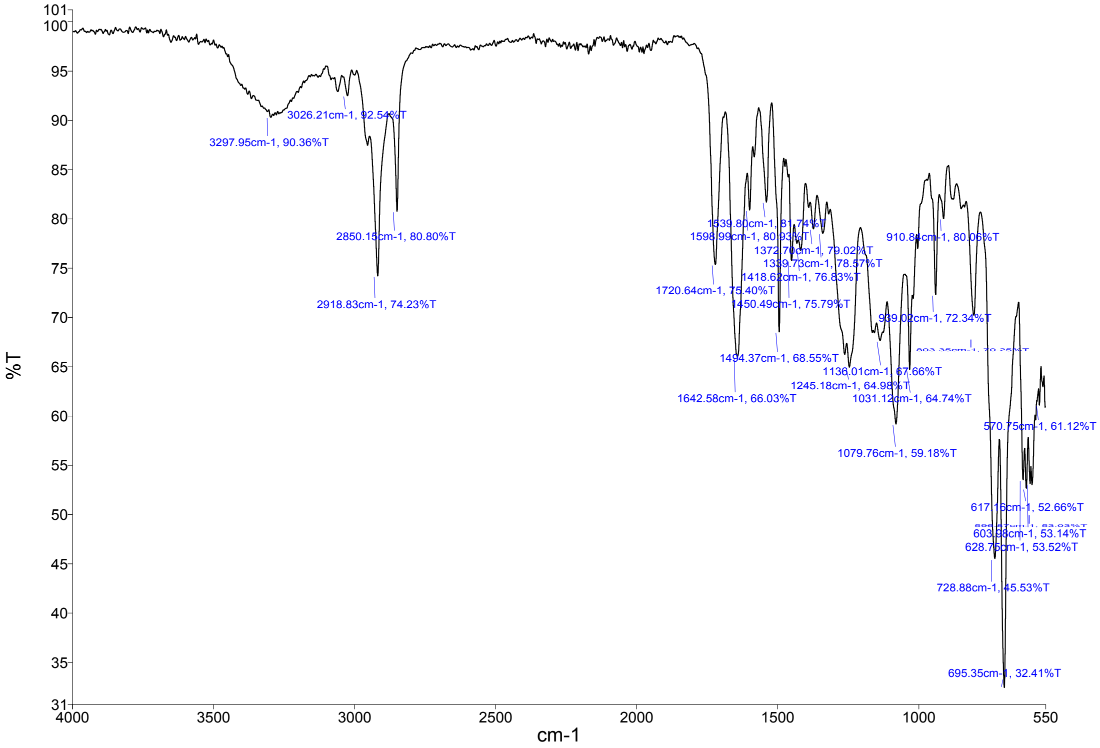

Fig S197: IR (neat) spectrum of 2,2-diphenyl-1-(1*H*-pyrrol-3-yl)ethan-1-one (**36**).

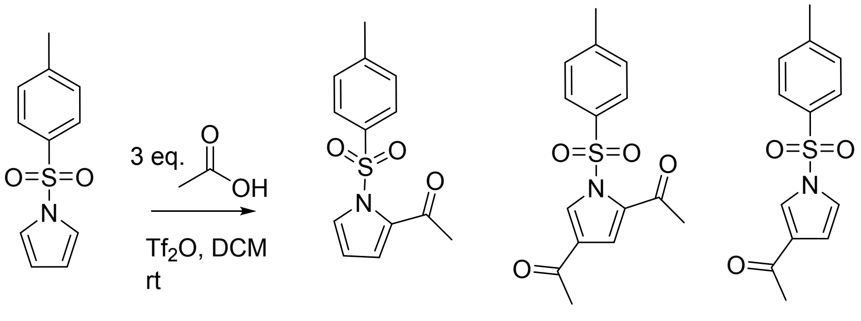

37 **38** **39**

Scheme S5: Formation of compounds **37**, **38** and **39** from 3 equivalents of acetic acid, showing multiple additions to pyrrole ring.

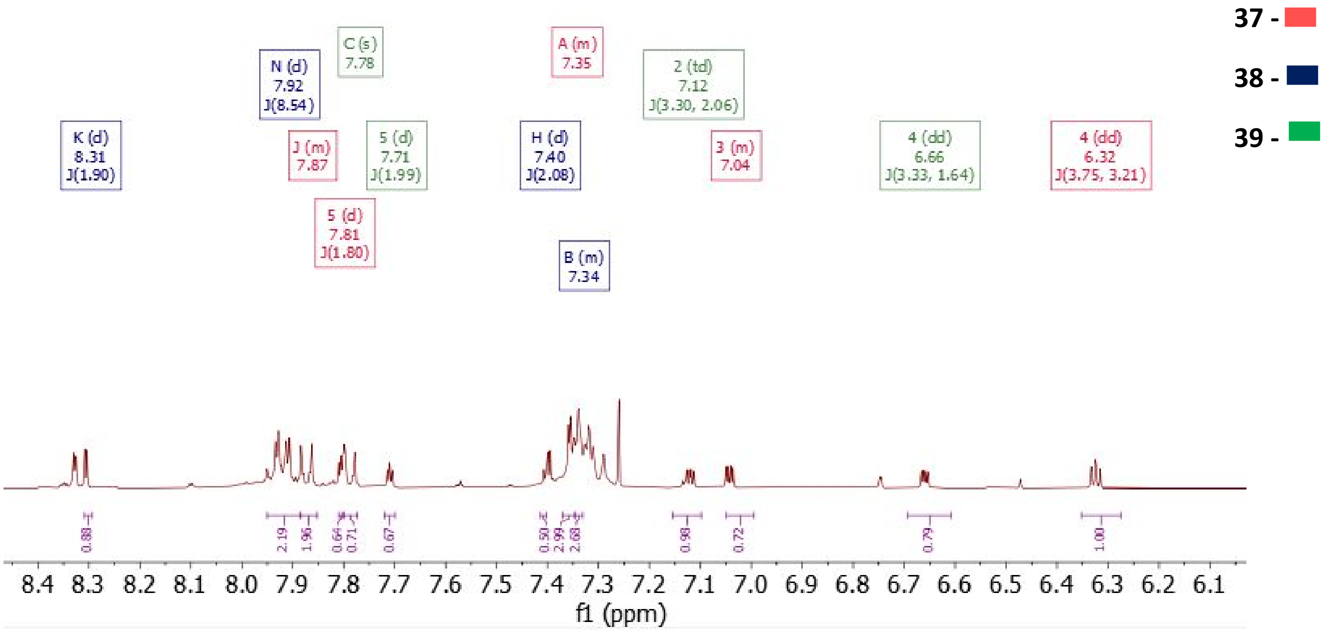

Fig S198: Crude ^{1}H NMR (400 MHz) spectrum (zoomed) showing formation of **37**, **38** and **39** by comparison to literature values (aromatic region). [15-17]

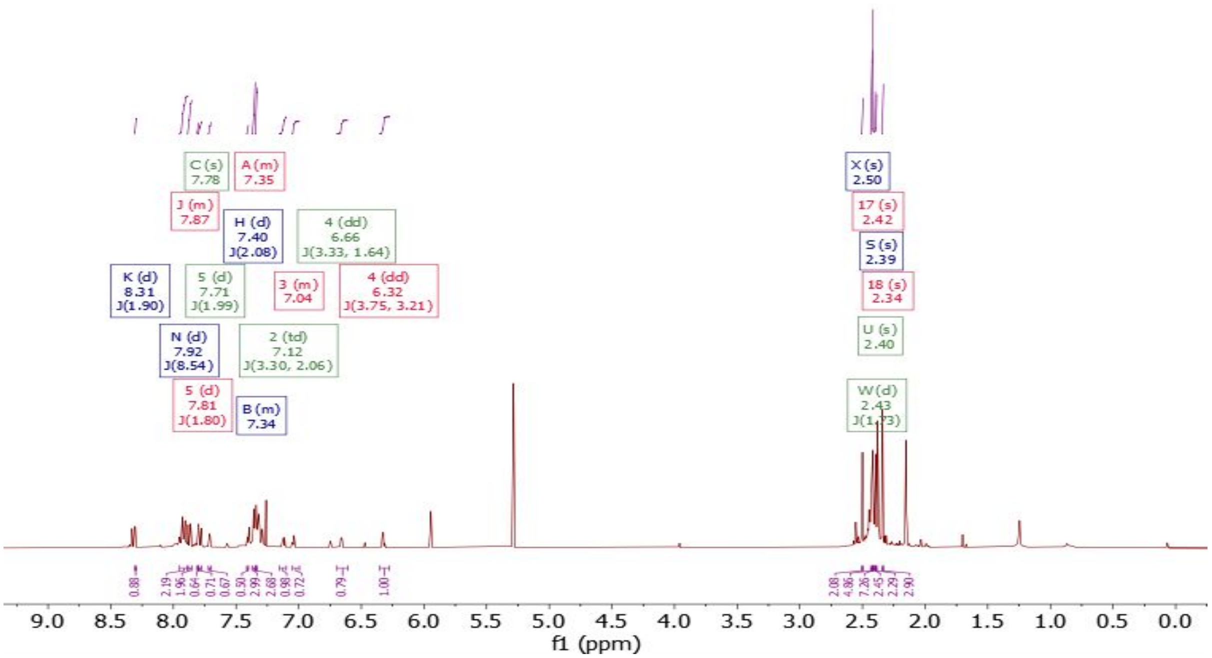

Fig S199: Crude ^{1}H NMR (400 MHz) spectrum showing formation of **37**, **38** and **39** by comparison to literature values

Fig S200: ORTEP diagram for 1**3**.

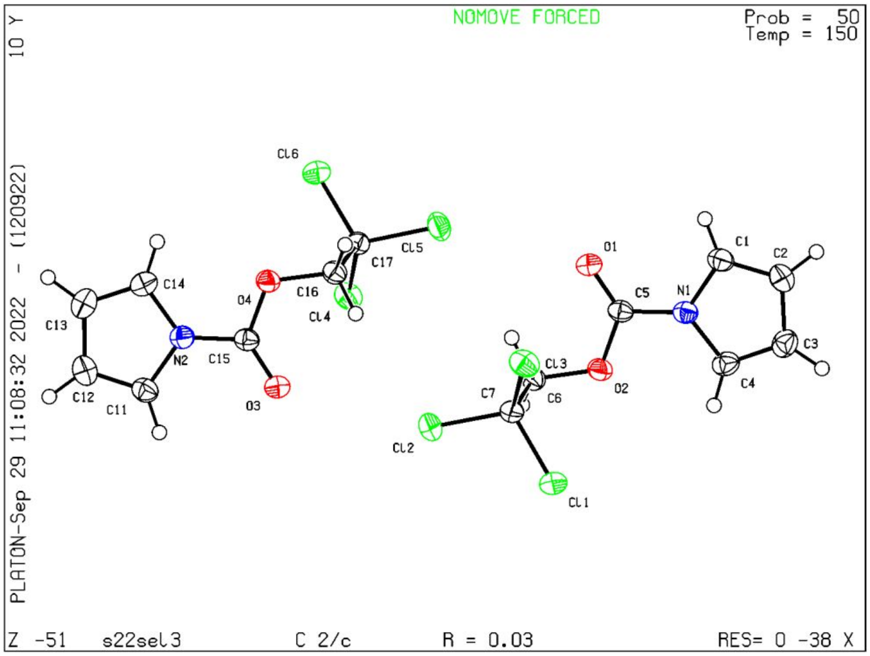

Table S1. Crystal data and structure refinement for **13**.

CCDC Identification code	2241194	
Empirical formula	$C_7H_6Cl_3NO_2$	
Formula weight	242.48	
Temperature	150(2) K	
Wavelength	1.54184 Å	
Crystal system	Monoclinic	
Space group	C2/c	
Unit cell dimensions	a = 20.86476(18) Å	a= 90°.
	b = 10.88425(7) Å	b= 109.4471(11)°.
	c = 18.06246(19) Å	g = 90°.
Volume	3867.92(6) $Å^3$	
Z	16	
Density (calculated)	1.666 Mg/m^3	
Absorption coefficient	8.329 mm^{-1}	
F(000)	1952	
Crystal size	0.535 x 0.425 x 0.343 mm^3	
Theta range for data collection	4.495 to 72.889°.	
Index ranges	-25<=h<=25, -13<=k<=13, -17<=l<=22	
Reflections collected	40299	
Independent reflections	3846 [R(int) = 0.0405]	
Completeness to theta = 67.684°	100.0 %	
Refinement method	Full-matrix least-squares on F^2	
Data / restraints / parameters	3846 / 0 / 236	
Goodness-of-fit on F^2	1.115	
Final R indices [I>2sigma(I)]	R1 = 0.0270, wR2 = 0.0743	
R indices (all data)	R1 = 0.0273, wR2 = 0.0747	
Extinction coefficient	0.00109(5)	
Largest diff. peak and hole	0.345 and -0.245 e.$Å^{-3}$	

Table S2. Atomic coordinates (x 10^4) and equivalent isotropic displacement parameters ($Å^2$x 10^3) for **13**. U(eq) is defined as one third of the trace of the orthogonalized U^{ij} tensor.

	x	y	z	U(eq)
C(1)	8928(1)	3049(1)	6083(1)	30(1)
C(2)	9307(1)	2132(1)	6521(1)	34(1)
C(3)	8869(1)	1376(1)	6790(1)	34(1)
C(4)	8233(1)	1850(1)	6510(1)	30(1)
C(5)	7733(1)	3666(1)	5672(1)	25(1)
C(6)	6573(1)	4076(1)	5502(1)	28(1)
C(7)	6484(1)	4794(1)	6182(1)	27(1)
C(11)	3634(1)	9482(1)	3984(1)	30(1)
C(12)	3257(1)	10370(1)	3512(1)	34(1)
C(13)	3684(1)	11002(2)	3161(1)	37(1)
C(14)	4310(1)	10489(1)	3426(1)	32(1)
C(15)	4818(1)	8787(1)	4364(1)	25(1)
C(16)	5976(1)	8355(1)	4532(1)	26(1)
C(17)	6053(1)	7569(1)	3872(1)	25(1)
N(1)	8262(1)	2888(1)	6069(1)	26(1)
N(2)	4287(1)	9544(1)	3939(1)	26(1)
O(1)	7787(1)	4520(1)	5279(1)	31(1)
O(2)	7157(1)	3295(1)	5791(1)	28(1)
O(3)	4768(1)	7983(1)	4793(1)	32(1)
O(4)	5390(1)	9124(1)	4224(1)	28(1)
Cl(1)	6420(1)	3776(1)	6920(1)	34(1)
Cl(2)	5725(1)	5656(1)	5806(1)	37(1)
Cl(3)	7176(1)	5806(1)	6596(1)	35(1)
Cl(4)	5370(1)	6522(1)	3535(1)	32(1)
Cl(5)	6825(1)	6745(1)	4254(1)	35(1)
Cl(6)	6080(1)	8507(1)	3081(1)	34(1)

Table S3. Bond lengths [Å] for **13**.

C(1)-C(2)	1.354(2)	C(11)-C(12)	1.353(2)
C(1)-N(1)	1.3923(17)	C(11)-N(2)	1.3921(18)
C(1)-H(1)	0.9500	C(11)-H(11)	0.9500
C(2)-C(3)	1.429(2)	C(12)-C(13)	1.430(2)
C(2)-H(2)	0.9500	C(12)-H(12)	0.9500
C(3)-C(4)	1.354(2)	C(13)-C(14)	1.353(2)
C(3)-H(3)	0.9500	C(13)-H(13)	0.9500
C(4)-N(1)	1.3956(18)	C(14)-N(2)	1.3954(18)
C(4)-H(4)	0.9500	C(14)-H(14)	0.9500
C(5)-O(1)	1.1970(17)	C(15)-O(3)	1.1959(17)
C(5)-O(2)	1.3515(16)	C(15)-O(4)	1.3510(16)
C(5)-N(1)	1.3864(18)	C(15)-N(2)	1.3894(18)
C(6)-O(2)	1.4341(17)	C(16)-O(4)	1.4312(16)
C(6)-C(7)	1.5181(19)	C(16)-C(17)	1.5193(18)
C(6)-H(6A)	0.9900	C(16)-H(16A)	0.9900
C(6)-H(6B)	0.9900	C(16)-H(16B)	0.9900
C(7)-Cl(3)	1.7705(13)	C(17)-Cl(4)	1.7682(13)
C(7)-Cl(2)	1.7706(14)	C(17)-Cl(5)	1.7694(13)
C(7)-Cl(1)	1.7733(14)	C(17)-Cl(6)	1.7717(13)

Table S4. Bond angles [°] for **13**.

C(2)-C(1)-N(1)	107.65(13)	C(11)-C(12)-H(12)	126.1
C(2)-C(1)-H(1)	126.2	C(13)-C(12)-H(12)	126.1
N(1)-C(1)-H(1)	126.2	C(14)-C(13)-C(12)	108.28(14)
C(1)-C(2)-C(3)	107.93(13)	C(14)-C(13)-H(13)	125.9
C(1)-C(2)-H(2)	126.0	C(12)-C(13)-H(13)	125.9
C(3)-C(2)-H(2)	126.0	C(13)-C(14)-N(2)	107.31(13)
C(4)-C(3)-C(2)	108.15(13)	C(13)-C(14)-H(14)	126.3
C(4)-C(3)-H(3)	125.9	N(2)-C(14)-H(14)	126.3
C(2)-C(3)-H(3)	125.9	O(3)-C(15)-O(4)	126.42(13)
C(3)-C(4)-N(1)	107.40(13)	O(3)-C(15)-N(2)	124.45(12)
C(3)-C(4)-H(4)	126.3	O(4)-C(15)-N(2)	109.12(11)
N(1)-C(4)-H(4)	126.3	O(4)-C(16)-C(17)	108.97(11)
O(1)-C(5)-O(2)	126.17(13)	O(4)-C(16)-H(16A)	109.9
O(1)-C(5)-N(1)	124.40(12)	C(17)-C(16)-H(16A)	109.9
O(2)-C(5)-N(1)	109.43(11)	O(4)-C(16)-H(16B)	109.9
O(2)-C(6)-C(7)	109.15(11)	C(17)-C(16)-H(16B)	109.9
O(2)-C(6)-H(6A)	109.9	H(16A)-C(16)-H(16B)	108.3
C(7)-C(6)-H(6A)	109.9	C(16)-C(17)-Cl(4)	110.38(9)
O(2)-C(6)-H(6B)	109.9	C(16)-C(17)-Cl(5)	107.46(9)
C(7)-C(6)-H(6B)	109.9	Cl(4)-C(17)-Cl(5)	109.42(7)
H(6A)-C(6)-H(6B)	108.3	C(16)-C(17)-Cl(6)	110.41(10)
C(6)-C(7)-Cl(3)	111.13(9)	Cl(4)-C(17)-Cl(6)	109.42(7)
C(6)-C(7)-Cl(2)	107.22(9)	Cl(5)-C(17)-Cl(6)	109.74(7)
Cl(3)-C(7)-Cl(2)	109.42(7)	C(5)-N(1)-C(1)	123.40(12)
C(6)-C(7)-Cl(1)	110.27(10)	C(5)-N(1)-C(4)	127.73(12)
Cl(3)-C(7)-Cl(1)	109.02(7)	C(1)-N(1)-C(4)	108.87(12)
Cl(2)-C(7)-Cl(1)	109.75(7)	C(15)-N(2)-C(11)	123.69(12)
C(12)-C(11)-N(2)	107.68(13)	C(15)-N(2)-C(14)	127.37(12)
C(12)-C(11)-H(11)	126.2	C(11)-N(2)-C(14)	108.92(12)
N(2)-C(11)-H(11)	126.2	C(5)-O(2)-C(6)	117.24(10)
C(11)-C(12)-C(13)	107.82(13)	C(15)-O(4)-C(16)	117.52(10)

Table S5. Anisotropic displacement parameters ($Å^2 x 10^3$) for **13**. The anisotropic displacement factor exponent takes the form: $-2p^2[h^2 a^{*2}U^{11} + ... + 2 h k a^* b^* U^{12}]$

	U^{11}	U^{22}	U^{33}	U^{23}	U^{13}	U^{12}
C(1)	28(1)	32(1)	32(1)	0(1)	15(1)	-1(1)
C(2)	30(1)	36(1)	37(1)	-2(1)	13(1)	4(1)
C(3)	42(1)	26(1)	36(1)	1(1)	15(1)	4(1)
C(4)	37(1)	24(1)	33(1)	-1(1)	16(1)	-3(1)
C(5)	27(1)	26(1)	25(1)	-4(1)	12(1)	-3(1)
C(6)	24(1)	31(1)	28(1)	1(1)	9(1)	-1(1)
C(7)	24(1)	28(1)	30(1)	2(1)	10(1)	-2(1)
C(11)	30(1)	31(1)	32(1)	-3(1)	14(1)	-1(1)
C(12)	30(1)	36(1)	35(1)	-4(1)	8(1)	4(1)
C(13)	41(1)	32(1)	34(1)	4(1)	8(1)	3(1)
C(14)	36(1)	29(1)	32(1)	2(1)	13(1)	-3(1)
C(15)	28(1)	24(1)	25(1)	-4(1)	12(1)	-2(1)
C(16)	24(1)	26(1)	27(1)	-1(1)	8(1)	-2(1)
C(17)	22(1)	26(1)	28(1)	1(1)	8(1)	-1(1)
N(1)	28(1)	25(1)	27(1)	-2(1)	13(1)	-2(1)
N(2)	29(1)	24(1)	27(1)	-2(1)	12(1)	-1(1)
O(1)	30(1)	33(1)	34(1)	7(1)	15(1)	0(1)
O(2)	26(1)	26(1)	37(1)	1(1)	16(1)	-1(1)
O(3)	32(1)	33(1)	34(1)	7(1)	17(1)	2(1)
O(4)	27(1)	23(1)	36(1)	2(1)	16(1)	0(1)
Cl(1)	36(1)	38(1)	33(1)	8(1)	18(1)	0(1)
Cl(2)	27(1)	39(1)	46(1)	7(1)	13(1)	7(1)
Cl(3)	31(1)	34(1)	41(1)	-8(1)	12(1)	-9(1)
Cl(4)	27(1)	30(1)	37(1)	-8(1)	9(1)	-6(1)
Cl(5)	24(1)	36(1)	43(1)	2(1)	9(1)	6(1)
Cl(6)	38(1)	37(1)	31(1)	6(1)	17(1)	0(1)

Table **S6**. Hydrogen coordinates (x 10^4) and isotropic displacement parameters ($Å^2$x 10 ^{3}) for **13**.

	x	y	z	U(eq)
H(1)	9088	3688	5832	36
H(2)	9780	2011	6630	41
H(3)	9001	665	7110	41
H(4)	7840	1533	6598	36
H(6A)	6163	3574	5248	33
H(6B)	6634	4651	5106	33
H(11)	3481	8918	4290	36
H(12)	2792	10542	3429	41
H(13)	3551	11669	2803	44
H(14)	4693	10727	3288	38
H(16A)	5922	7822	4952	31
H(16B)	6386	8867	4760	31

Table S7. Torsion angles [°] for **13**.

N(1)-C(1)-C(2)-C(3)	-0.16(17)	C(2)-C(1)-N(1)-C(4)	0.20(16)
C(1)-C(2)-C(3)-C(4)	0.06(18)	C(3)-C(4)-N(1)-C(5)	-179.92(13)
C(2)-C(3)-C(4)-N(1)	0.06(17)	C(3)-C(4)-N(1)-C(1)	-0.16(16)
O(2)-C(6)-C(7)-Cl(3)	64.64(12)	O(3)-C(15)-N(2)-C(11)	2.2(2)
O(2)-C(6)-C(7)-Cl(2)	-175.82(8)	O(4)-C(15)-N(2)-C(11)	-176.58(12)
O(2)-C(6)-C(7)-Cl(1)	-56.37(12)	O(3)-C(15)-N(2)-C(14)	-179.46(13)
N(2)-C(11)-C(12)-C(13)	0.21(17)	O(4)-C(15)-N(2)-C(14)	1.74(18)
C(11)-C(12)-C(13)-C(14)	-0.08(18)	C(12)-C(11)-N(2)-C(15)	178.33(12)
C(12)-C(13)-C(14)-N(2)	-0.09(17)	C(12)-C(11)-N(2)-C(14)	-0.26(16)
O(4)-C(16)-C(17)-Cl(4)	-66.30(12)	C(13)-C(14)-N(2)-C(15)	-178.31(13)
O(4)-C(16)-C(17)-Cl(5)	174.45(8)	C(13)-C(14)-N(2)-C(11)	0.22(16)
O(4)-C(16)-C(17)-Cl(6)	54.80(12)	O(1)-C(5)-O(2)-C(6)	-8.3(2)
O(1)-C(5)-N(1)-C(1)	3.1(2)	N(1)-C(5)-O(2)-C(6)	172.76(11)
O(2)-C(5)-N(1)-C(1)	-177.88(12)	C(7)-C(6)-O(2)-C(5)	-102.25(13)
O(1)-C(5)-N(1)-C(4)	-177.13(13)	O(3)-C(15)-O(4)-C(16)	8.91(19)
O(2)-C(5)-N(1)-C(4)	1.85(18)	N(2)-C(15)-O(4)-C(16)	-172.32(10)
C(2)-C(1)-N(1)-C(5)	179.97(12)	C(17)-C(16)-O(4)-C(15)	102.10(13)

Table S8. Hydrogen bonds for **13** [Å and °].

D-H...A	d(D-H)	d(H...A)	d(D...A)	<(DHA)
C(1)-H(1)...O(4)#1	0.95	2.63	3.5110(17)	153.6
C(6)-H(6A)...O(3)#2	0.99	2.56	3.4840(17)	155.5
C(16)-H(16A)...Cl(2)	0.99	2.92	3.8730(14)	161.6
C(16)-H(16B)...O(1)#1	0.99	2.48	3.3985(17)	154.3

Symmetry transformations used to generate equivalent atoms:
#1 -x+3/2,-y+3/2,-z+1   #2 -x+1,-y+1,-z+1

Fig S201: ORTEP diagram for **25**.

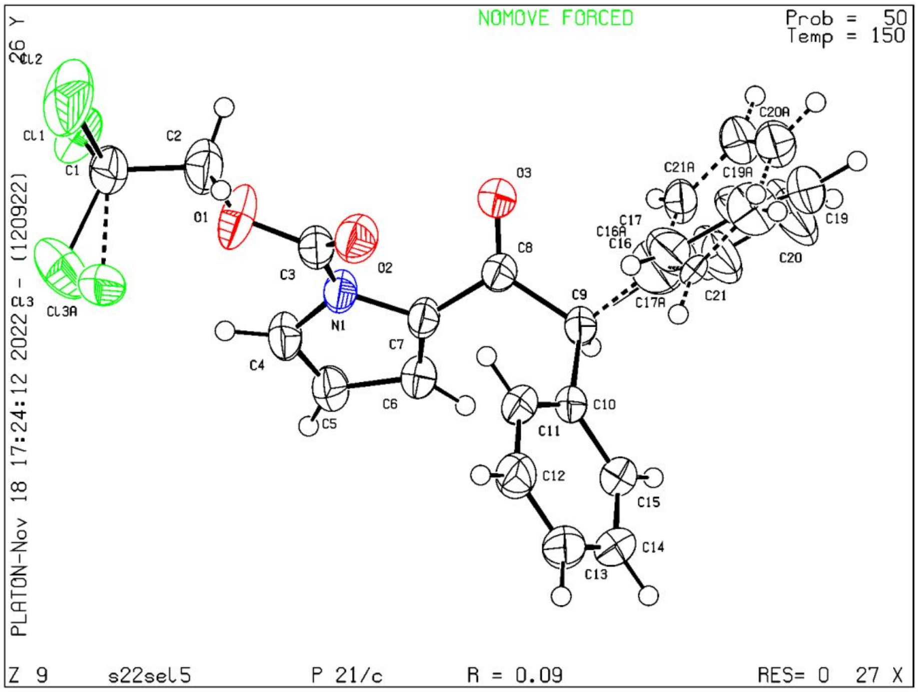

Table S9. Crystal data and structure refinement for **25**.

CCDC Identification code	2241195	
Empirical formula	$C_{21}H_{16}Cl_3NO_3$	
Formula weight	436.70	
Temperature	150.00(10) K	
Wavelength	1.54184 Å	
Crystal system	Monoclinic	
Space group	P21/c	
Unit cell dimensions	a = 13.1795(6) Å	?= 90°.
	b = 14.4050(8) Å	?= 95.145(4)°.
	c = 10.5716(4) Å	? = 90°.
Volume	1998.94(16) Å3	
Z	4	
Density (calculated)	1.451 Mg/m3	
Absorption coefficient	4.343 mm-1	
F(000)	896	
Crystal size	0.350 x 0.200 x 0.080 mm3	
Theta range for data collection	4.557 to 73.389°.	
Index ranges	-16<=h<=16, -17<=k<=17, -10<=l<=13	
Reflections collected	22530	
Independent reflections	3980 [R(int) = 0.0803]	
Completeness to theta = 67.684°	100.0 %	
Absorption correction	Gaussian	
Max. and min. transmission	1.000 and 0.241	
Refinement method	Full-matrix least-squares on F2	
Data / restraints / parameters	3980 / 36 / 293	
Goodness-of-fit on F2	1.086	
Final R indices [I>2sigma(I)]	R1 = 0.0931, wR2 = 0.2336	
R indices (all data)	R1 = 0.0976, wR2 = 0.2373	
Extinction coefficient	n/a	
Largest diff. peak and hole	0.566 and -0.463 e.Å-3	

Table S10 Atomic coordinates (x 10^4) and equivalent isotropic displacement parameters ($Å^2$x 10^3) for **25**. U(eq) is defined as one third of the trace of the orthogonalized Uij tensor.

	x	y	z	U(eq)
Cl(1)	2302(1)	6814(1)	8596(2)	81(1)
Cl(2)	3255(2)	6334(2)	11069(2)	119(1)
N(1)	5070(2)	6416(2)	5988(3)	44(1)
O(1)	4298(2)	6454(3)	7760(3)	71(1)
O(2)	5999(2)	6370(2)	7923(3)	57(1)
O(3)	6765(2)	7717(2)	6358(3)	54(1)
C(1)	3282(4)	6153(4)	9415(5)	61(1)
C(2)	4307(4)	6496(4)	9108(5)	64(1)
C(3)	5213(3)	6414(3)	7298(4)	45(1)
C(4)	4233(3)	6029(3)	5270(5)	52(1)
C(5)	4453(3)	5937(3)	4057(5)	51(1)
C(6)	5468(3)	6279(3)	3981(4)	45(1)
C(7)	5838(3)	6564(3)	5167(4)	40(1)
C(8)	6753(3)	7126(3)	5538(4)	41(1)
C(9)	7663(3)	6925(3)	4777(4)	40(1)
C(10)	8003(3)	5914(3)	4900(4)	40(1)
C(11)	7918(3)	5402(3)	5990(4)	46(1)
C(12)	8285(4)	4503(3)	6099(5)	57(1)
C(13)	8742(4)	4103(3)	5096(6)	67(2)
C(14)	8831(4)	4608(3)	4013(6)	65(1)
C(15)	8457(3)	5507(3)	3898(4)	50(1)
C(16)	8541(3)	7579(3)	5108(5)	44(3)
C(17)	9177(5)	7468(4)	6219(6)	76(3)
C(18)	10024(4)	8037(5)	6458(6)	105(7)
C(19)	10235(4)	8717(4)	5586(7)	114(5)
C(20)	9599(6)	8828(4)	4475(6)	146(6)
C(21)	8751(5)	8259(4)	4236(5)	93(3)
Cl(3)	3030(3)	4982(2)	9114(3)	83(1)
C(16A)	8567(12)	7632(8)	5232(17)	83(19)
C(17A)	9434(13)	7376(8)	5997(17)	27(4)
C(18A)	10116(11)	8050(12)	6483(15)	71(14)
C(19A)	9930(10)	8981(10)	6204(15)	52(5)
C(20A)	9063(10)	9237(7)	5438(15)	49(5)
C(21A)	8381(9)	8563(9)	4952(14)	41(4)
Cl(3A)	3487(7)	4967(6)	8834(11)	70(2)

Table S11. Bond lengths [Å] for **25**.

Cl(1)-C(1)	1.767(6)	C(12)-C(13)	1.391(7)
Cl(2)-C(1)	1.771(5)	C(12)-H(12)	0.9500
N(1)-C(3)	1.381(6)	C(13)-C(14)	1.370(8)
N(1)-C(4)	1.397(6)	C(13)-H(13)	0.9500
N(1)-C(7)	1.408(5)	C(14)-C(15)	1.387(7)
O(1)-C(3)	1.342(5)	C(14)-H(14)	0.9500
O(1)-C(2)	1.425(6)	C(15)-H(15)	0.9500
O(2)-C(3)	1.179(5)	C(16)-C(17)	1.3900
O(3)-C(8)	1.214(5)	C(16)-C(21)	1.3900
C(1)-C(2)	1.501(6)	C(17)-C(18)	1.3900
C(1)-Cl(3)	1.743(6)	C(17)-H(17)	0.9500
C(1)-Cl(3A)	1.844(9)	C(18)-C(19)	1.3900
C(2)-H(2A)	0.9900	C(18)-H(18)	0.9500
C(2)-H(2B)	0.9900	C(19)-C(20)	1.3900
C(4)-C(5)	1.346(7)	C(19)-H(19)	0.9500
C(4)-H(4)	0.9500	C(20)-C(21)	1.3900
C(5)-C(6)	1.435(5)	C(20)-H(20)	0.9500
C(5)-H(5)	0.9500	C(21)-H(21)	0.9500
C(6)-C(7)	1.367(6)	C(16A)-C(17A)	1.3900
C(6)-H(6)	0.9500	C(16A)-C(21A)	1.3900
C(7)-C(8)	1.477(5)	C(17A)-C(18A)	1.3900
C(8)-C(9)	1.530(5)	C(17A)-H(17A)	0.9500
C(9)-C(16)	1.508(5)	C(18A)-C(19A)	1.3900
C(9)-C(10)	1.525(5)	C(18A)-H(18A)	0.9500
C(9)-C(16A)	1.607(13)	C(19A)-C(20A)	1.3900
C(9)-H(9)	1.0000	C(19A)-H(19A)	0.9500
C(10)-C(11)	1.382(6)	C(20A)-C(21A)	1.3900
C(10)-C(15)	1.392(5)	C(20A)-H(20A)	0.9500
C(11)-C(12)	1.384(6)	C(21A)-H(21A)	0.9500
C(11)-H(11)	0.9500		

Table S12. Bond angles [°] for **25**.

C(3)-N(1)-C(4)	125.1(3)	C(12)-C(11)-H(11)	119.5
C(3)-N(1)-C(7)	125.3(4)	C(11)-C(12)-C(13)	119.7(5)
C(4)-N(1)-C(7)	107.6(3)	C(11)-C(12)-H(12)	120.1
C(3)-O(1)-C(2)	116.0(4)	C(13)-C(12)-H(12)	120.1
C(2)-C(1)-Cl(3)	116.0(4)	C(14)-C(13)-C(12)	119.5(4)
C(2)-C(1)-Cl(1)	110.4(4)	C(14)-C(13)-H(13)	120.3
Cl(3)-C(1)-Cl(1)	108.2(3)	C(12)-C(13)-H(13)	120.3
C(2)-C(1)-Cl(2)	105.3(4)	C(13)-C(14)-C(15)	120.9(4)
Cl(3)-C(1)-Cl(2)	107.6(3)	C(13)-C(14)-H(14)	119.6
Cl(1)-C(1)-Cl(2)	109.0(3)	C(15)-C(14)-H(14)	119.6
C(2)-C(1)-Cl(3A)	94.0(5)	C(14)-C(15)-C(10)	120.0(4)
Cl(1)-C(1)-Cl(3A)	117.3(4)	C(14)-C(15)-H(15)	120.0
Cl(2)-C(1)-Cl(3A)	118.8(5)	C(10)-C(15)-H(15)	120.0
O(1)-C(2)-C(1)	106.0(4)	C(17)-C(16)-C(21)	120.0
O(1)-C(2)-H(2A)	110.5	C(17)-C(16)-C(9)	121.1(4)
C(1)-C(2)-H(2A)	110.5	C(21)-C(16)-C(9)	118.8(4)
O(1)-C(2)-H(2B)	110.5	C(18)-C(17)-C(16)	120.0
C(1)-C(2)-H(2B)	110.5	C(18)-C(17)-H(17)	120.0
H(2A)-C(2)-H(2B)	108.7	C(16)-C(17)-H(17)	120.0
O(2)-C(3)-O(1)	124.8(4)	C(19)-C(18)-C(17)	120.0
O(2)-C(3)-N(1)	126.6(4)	C(19)-C(18)-H(18)	120.0
O(1)-C(3)-N(1)	108.6(4)	C(17)-C(18)-H(18)	120.0
C(5)-C(4)-N(1)	109.3(3)	C(18)-C(19)-C(20)	120.0
C(5)-C(4)-H(4)	125.4	C(18)-C(19)-H(19)	120.0
N(1)-C(4)-H(4)	125.4	C(20)-C(19)-H(19)	120.0
C(4)-C(5)-C(6)	107.5(4)	C(21)-C(20)-C(19)	120.0
C(4)-C(5)-H(5)	126.2	C(21)-C(20)-H(20)	120.0
C(6)-C(5)-H(5)	126.2	C(19)-C(20)-H(20)	120.0
C(7)-C(6)-C(5)	108.0(4)	C(20)-C(21)-C(16)	120.0
C(7)-C(6)-H(6)	126.0	C(20)-C(21)-H(21)	120.0
C(5)-C(6)-H(6)	126.0	C(16)-C(21)-H(21)	120.0
C(6)-C(7)-N(1)	107.7(3)	C(17A)-C(16A)-C(21A)	120.0
C(6)-C(7)-C(8)	128.6(4)	C(17A)-C(16A)-C(9)	123.6(10)
N(1)-C(7)-C(8)	122.3(4)	C(21A)-C(16A)-C(9)	115.9(10)
O(3)-C(8)-C(7)	122.1(3)	C(18A)-C(17A)-C(16A)	120.0
O(3)-C(8)-C(9)	123.2(4)	C(18A)-C(17A)-H(17A)	120.0
C(7)-C(8)-C(9)	114.7(3)	C(16A)-C(17A)-H(17A)	120.0
C(16)-C(9)-C(10)	111.3(3)	C(17A)-C(18A)-C(19A)	120.0
C(16)-C(9)-C(8)	112.4(3)	C(17A)-C(18A)-H(18A)	120.0
C(10)-C(9)-C(8)	111.9(3)	C(19A)-C(18A)-H(18A)	120.0
C(10)-C(9)-C(16A)	112.1(6)	C(18A)-C(19A)-C(20A)	120.0
C(8)-C(9)-C(16A)	108.5(7)	C(18A)-C(19A)-H(19A)	120.0
C(16)-C(9)-H(9)	106.9	C(20A)-C(19A)-H(19A)	120.0
C(10)-C(9)-H(9)	106.9	C(21A)-C(20A)-C(19A)	120.0
C(8)-C(9)-H(9)	106.9	C(21A)-C(20A)-H(20A)	120.0
C(11)-C(10)-C(15)	118.8(4)	C(19A)-C(20A)-H(20A)	120.0
C(11)-C(10)-C(9)	122.4(3)	C(20A)-C(21A)-C(16A)	120.0
C(15)-C(10)-C(9)	118.7(4)	C(20A)-C(21A)-H(21A)	120.0
C(10)-C(11)-C(12)	121.0(4)	C(16A)-C(21A)-H(21A)	120.0
C(10)-C(11)-H(11)	119.5		

Table S13. Anisotropic displacement parameters ($Å^2$x 10^3) for **25**. The anisotropic displacement factor exponent takes the form: $-2p^2[h^2 a^{*2}U^{11} + ... + 2 h k a^* b^* U^{12}]$

	U^{11}	U^{22}	U^{33}	U^{23}	U^{13}	U^{12}
Cl(1)	62(1)	98(1)	87(1)	-9(1)	34(1)	9(1)
Cl(2)	106(1)	205(2)	52(1)	-19(1)	38(1)	-50(1)
N(1)	34(2)	44(2)	55(2)	3(2)	15(2)	-2(1)
O(1)	43(2)	117(3)	56(2)	14(2)	22(2)	1(2)
O(2)	45(2)	67(2)	59(2)	-2(2)	8(2)	0(1)
O(3)	47(2)	42(2)	74(2)	-11(1)	22(2)	-6(1)
C(1)	64(3)	65(3)	57(3)	-4(2)	27(2)	-18(2)
C(2)	53(3)	87(4)	54(3)	-1(2)	17(2)	-18(3)
C(3)	37(2)	41(2)	58(3)	4(2)	12(2)	-2(2)
C(4)	33(2)	57(2)	66(3)	1(2)	12(2)	-10(2)
C(5)	37(2)	58(3)	58(3)	-6(2)	8(2)	-10(2)
C(6)	34(2)	49(2)	54(2)	-2(2)	10(2)	-3(2)
C(7)	30(2)	38(2)	53(2)	4(2)	15(2)	1(1)
C(8)	36(2)	37(2)	52(2)	-1(2)	14(2)	0(2)
C(9)	31(2)	35(2)	54(2)	-1(2)	13(2)	-4(1)
C(10)	30(2)	37(2)	54(2)	-7(2)	13(2)	-5(1)
C(11)	39(2)	43(2)	60(3)	-5(2)	19(2)	1(2)
C(12)	53(3)	42(2)	78(3)	7(2)	23(2)	3(2)
C(13)	60(3)	37(2)	109(4)	-4(2)	35(3)	4(2)
C(14)	61(3)	50(2)	89(4)	-21(2)	38(3)	-4(2)
C(15)	48(2)	48(2)	55(2)	-12(2)	21(2)	-9(2)
C(16)	31(3)	27(3)	78(5)	-8(3)	22(3)	-6(2)
C(17)	51(5)	47(4)	127(9)	-14(4)	-1(5)	-7(3)
C(18)	45(4)	60(6)	204(16)	-50(7)	-18(6)	2(4)
C(19)	67(5)	95(7)	189(12)	-73(7)	67(7)	-47(5)
C(20)	197(13)	133(9)	119(8)	-44(7)	77(9)	-129(10)
C(21)	126(7)	74(5)	82(5)	-3(4)	33(5)	-61(5)
Cl(3)	102(2)	65(1)	81(2)	16(1)	-2(1)	-37(2)
C(16A)	80(20)	80(20)	90(20)	-2(10)	8(10)	-3(10)
C(17A)	25(8)	30(7)	28(7)	-6(5)	14(6)	0(6)
C(18A)	71(16)	67(17)	75(16)	1(9)	6(9)	-6(9)
C(19A)	43(8)	47(8)	64(9)	5(7)	5(7)	-3(7)
C(20A)	46(8)	47(8)	55(9)	9(7)	4(7)	-7(7)
C(21A)	32(7)	38(7)	56(9)	10(6)	11(6)	-1(6)
Cl(3A)	60(5)	48(4)	105(6)	-21(4)	21(4)	-4(3)

Table S14. Hydrogen coordinates (x 10^4) and isotropic displacement parameters (Å^{2}x 10 ^{3}) for **25**.

	x	y	z	U(eq)
H(2A)	4421	7141	9411	77
H(2B)	4855	6097	9515	77
H(4)	3608	5857	5588	62
H(5)	4015	5691	3376	61
H(6)	5821	6303	3236	54
H(9)	7430	7032	3863	48
H(11)	7603	5671	6676	56
H(12)	8226	4160	6857	68
H(13)	8991	3484	5160	80
H(14)	9153	4339	3332	78
H(15)	8512	5845	3135	59
H(17)	9033	7003	6815	91
H(18)	10459	7961	7217	126
H(19)	10814	9106	5749	136
H(20)	9743	9293	3879	175
H(21)	8316	8335	3476	111
H(17A)	9561	6740	6188	32
H(18A)	10709	7875	7006	85
H(19A)	10396	9442	6536	62
H(20A)	8936	9874	5247	59
H(21A)	7788	8738	4429	50

Table S15. Torsion angles [°] for **25**.

C(3)-O(1)-C(2)-C(1)	-157.7(4)	C(8)-C(9)-C(10)-C(15)	150.9(4)
Cl(3)-C(1)-C(2)-O(1)	67.6(6)	C(16A)-C(9)-C(10)-C(15)	-86.9(8)
Cl(1)-C(1)-C(2)-O(1)	-56.0(5)	C(15)-C(10)-C(11)-C(12)	0.6(6)
Cl(2)-C(1)-C(2)-O(1)	-173.5(4)	C(9)-C(10)-C(11)-C(12)	-176.3(4)
Cl(3A)-C(1)-C(2)-O(1)	65.1(6)	C(10)-C(11)-C(12)-C(13)	-0.4(7)
C(2)-O(1)-C(3)-O(2)	3.5(7)	C(11)-C(12)-C(13)-C(14)	0.6(8)
C(2)-O(1)-C(3)-N(1)	-177.6(4)	C(12)-C(13)-C(14)-C(15)	-1.0(8)
C(4)-N(1)-C(3)-O(2)	147.0(5)	C(13)-C(14)-C(15)-C(10)	1.3(8)
C(7)-N(1)-C(3)-O(2)	-14.4(7)	C(11)-C(10)-C(15)-C(14)	-1.1(6)
C(4)-N(1)-C(3)-O(1)	-31.9(6)	C(9)-C(10)-C(15)-C(14)	176.0(4)
C(7)-N(1)-C(3)-O(1)	166.7(4)	C(10)-C(9)-C(16)-C(17)	-49.4(5)
C(3)-N(1)-C(4)-C(5)	-164.3(4)	C(8)-C(9)-C(16)-C(17)	77.1(5)
C(7)-N(1)-C(4)-C(5)	-0.2(5)	C(10)-C(9)-C(16)-C(21)	125.9(4)
N(1)-C(4)-C(5)-C(6)	-0.2(5)	C(8)-C(9)-C(16)-C(21)	-107.6(5)
C(4)-C(5)-C(6)-C(7)	0.5(5)	C(21)-C(16)-C(17)-C(18)	0.0
C(5)-C(6)-C(7)-N(1)	-0.6(5)	C(9)-C(16)-C(17)-C(18)	175.3(4)
C(5)-C(6)-C(7)-C(8)	-167.1(4)	C(16)-C(17)-C(18)-C(19)	0.0
C(3)-N(1)-C(7)-C(6)	164.6(4)	C(17)-C(18)-C(19)-C(20)	0.0
C(4)-N(1)-C(7)-C(6)	0.5(5)	C(18)-C(19)-C(20)-C(21)	0.0
C(3)-N(1)-C(7)-C(8)	-27.9(6)	C(19)-C(20)-C(21)-C(16)	0.0
C(4)-N(1)-C(7)-C(8)	168.0(4)	C(17)-C(16)-C(21)-C(20)	0.0
C(6)-C(7)-C(8)-O(3)	141.0(5)	C(9)-C(16)-C(21)-C(20)	-175.4(4)
N(1)-C(7)-C(8)-O(3)	-23.8(6)	C(10)-C(9)-C(16A)-C(17A)	-18.7(12)
C(6)-C(7)-C(8)-C(9)	-37.0(6)	C(8)-C(9)-C(16A)-C(17A)	105.5(9)
N(1)-C(7)-C(8)-C(9)	158.2(4)	C(10)-C(9)-C(16A)-C(21A)	169.4(7)
O(3)-C(8)-C(9)-C(16)	-3.4(6)	C(8)-C(9)-C(16A)-C(21A)	-66.5(9)
C(7)-C(8)-C(9)-C(16)	174.5(4)	C(21A)-C(16A)-C(17A)-C(18A)	0.0
O(3)-C(8)-C(9)-C(10)	122.7(4)	C(9)-C(16A)-C(17A)-C(18A)	-171.6(14)
C(7)-C(8)-C(9)-C(10)	-59.3(5)	C(16A)-C(17A)-C(18A)-C(19A)	0.0
O(3)-C(8)-C(9)-C(16A)	-1.5(8)	C(17A)-C(18A)-C(19A)-C(20A)	0.0
C(7)-C(8)-C(9)-C(16A)	176.5(7)	C(18A)-C(19A)-C(20A)-C(21A)	0.0
C(16)-C(9)-C(10)-C(11)	94.6(5)	C(19A)-C(20A)-C(21A)-C(16A)	0.0
C(8)-C(9)-C(10)-C(11)	-32.2(5)	C(17A)-C(16A)-C(21A)-C(20A)	0.0
C(16A)-C(9)-C(10)-C(11)	90.1(8)	C(9)-C(16A)-C(21A)-C(20A)	172.3(13)
C(16)-C(9)-C(10)-C(15)	-82.3(5)		

Fig S202: ORTEP diagram for **27**.

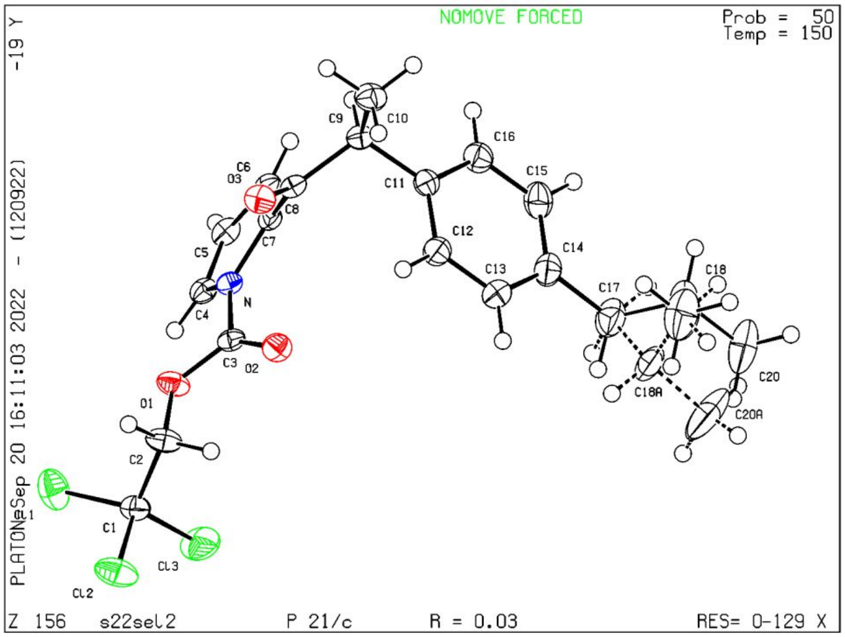

Table S16. Crystal data and structure refinement for **27**.

CCDC Identification code	2241193	
Empirical formula	$C_{20}H_{22}Cl_3NO_3$	
Formula weight	430.73	
Temperature	150.00(10) K	
Wavelength	1.54184 Å	
Crystal system	Monoclinic	
Space group	$P2_1/c$	
Unit cell dimensions	a = 14.59396(14) Å	a= 90°.
	b = 14.46655(15) Å	b= 97.2582(9)°.
	c = 10.28532(11) Å	g = 90°.
Volume	2154.08(4) $Å^3$	
Z	4	
Density (calculated)	1.328 Mg/m^3	
Absorption coefficient	4.014 mm^{-1}	
F(000)	896	
Crystal size	0.180 x 0.150 x 0.030 mm^3	
Theta range for data collection	4.321 to 72.872°.	
Index ranges	-18<=h<=18, -17<=k<=17, -12<=l<=12	
Reflections collected	33579	
Independent reflections	4276 [R(int) = 0.0296]	
Completeness to theta = 67.684°	100.0 %	
Absorption correction	Semi-empirical from equivalents	
Max. and min. transmission	1.00000 and 0.60126	
Refinement method	Full-matrix least-squares on F^2	
Data / restraints / parameters	4276 / 0 / 267	
Goodness-of-fit on F^2	1.028	
Final R indices [I>2sigma(I)]	R1 = 0.0340, wR2 = 0.0858	
R indices (all data)	R1 = 0.0357, wR2 = 0.0871	
Extinction coefficient	n/a	
Largest diff. peak and hole	0.557 and -0.533 e.$Å^{-3}$	

Table S17. Atomic coordinates (x 10^4) and equivalent isotropic displacement parameters ($Å^2$x 10^3) for **27**. U(eq) is defined as one third of the trace of the orthogonalized U^{ij} tensor.

	x	y	z	U(eq)
N	5234(1)	6393(1)	4438(1)	20(1)
O(1)	6001(1)	6518(1)	2694(1)	29(1)
O(2)	4443(1)	6624(1)	2378(1)	28(1)
O(3)	3943(1)	7928(1)	4173(1)	27(1)
Cl(1)	7853(1)	6321(1)	1802(1)	44(1)
Cl(2)	6858(1)	6536(1)	-772(1)	45(1)
Cl(3)	6492(1)	4972(1)	844(1)	50(1)
C(1)	6775(1)	6155(1)	844(1)	27(1)
C(2)	6033(1)	6738(1)	1340(1)	30(1)
C(3)	5153(1)	6525(1)	3075(1)	21(1)
C(4)	5906(1)	5848(1)	5144(1)	23(1)
C(5)	5648(1)	5698(1)	6351(1)	24(1)
C(6)	4791(1)	6153(1)	6405(1)	22(1)
C(7)	4537(1)	6572(1)	5221(1)	20(1)
C(8)	3815(1)	7276(1)	4878(1)	21(1)
C(9)	2916(1)	7136(1)	5458(1)	23(1)
C(10)	2296(1)	7985(1)	5231(2)	31(1)
C(11)	2445(1)	6266(1)	4878(1)	23(1)
C(12)	2411(1)	6078(1)	3548(2)	30(1)
C(13)	1997(1)	5280(1)	3008(2)	34(1)
C(14)	1601(1)	4644(1)	3777(2)	33(1)
C(15)	1613(1)	4846(1)	5097(2)	37(1)
C(16)	2034(1)	5638(1)	5648(2)	32(1)
C(17)	1201(1)	3743(1)	3214(2)	45(1)
C(19)	-192(2)	4415(2)	1846(3)	64(1)
C(18)	161(2)	3711(2)	2928(3)	43(1)
C(20)	-152(2)	2730(2)	2534(4)	64(1)
C(18A)	583(6)	3870(7)	1740(11)	46(3)
C(20A)	294(10)	2891(9)	1256(18)	87(6)

Table S18. Bond lengths [Å] for **27**.

N-C(4)	1.3888(18)	C(13)-C(14)	1.386(2)
N-C(7)	1.3991(17)	C(13)-H(13)	0.9500
N-C(3)	1.4052(18)	C(14)-C(15)	1.387(3)
O(1)-C(3)	1.3440(18)	C(14)-C(17)	1.513(2)
O(1)-C(2)	1.4357(17)	C(15)-C(16)	1.387(2)
O(2)-C(3)	1.1916(18)	C(15)-H(15)	0.9500
O(3)-C(8)	1.2181(17)	C(16)-H(16)	0.9500
Cl(1)-C(1)	1.7638(16)	C(17)-C(18)	1.510(3)
Cl(2)-C(1)	1.7697(15)	C(17)-C(18A)	1.673(10)
Cl(3)-C(1)	1.7602(15)	C(17)-H(17A)	0.9900
C(1)-C(2)	1.511(2)	C(17)-H(17B)	0.9900
C(2)-H(2A)	0.9900	C(17)-H(17C)	0.9900
C(2)-H(2B)	0.9900	C(17)-H(17D)	0.9900
C(4)-C(5)	1.359(2)	C(19)-C(18A)	1.395(10)
C(4)-H(4)	0.9500	C(19)-C(18)	1.548(3)
C(5)-C(6)	1.420(2)	C(19)-H(19A)	0.9800
C(5)-H(5)	0.9500	C(19)-H(19B)	0.9800
C(6)-C(7)	1.3693(19)	C(19)-H(19C)	0.9800
C(6)-H(6)	0.9500	C(19)-H(19D)	0.9800
C(7)-C(8)	1.4764(19)	C(19)-H(19E)	0.9800
C(8)-C(9)	1.5214(19)	C(19)-H(19F)	0.9800
C(9)-C(11)	1.520(2)	C(18)-C(20)	1.530(3)
C(9)-C(10)	1.526(2)	C(18)-H(18)	1.0000
C(9)-H(9)	1.0000	C(20)-H(20A)	0.9800
C(10)-H(10A)	0.9800	C(20)-H(20B)	0.9800
C(10)-H(10B)	0.9800	C(20)-H(20C)	0.9800
C(10)-H(10C)	0.9800	C(18A)-C(20A)	1.542(14)
C(11)-C(12)	1.389(2)	C(18A)-H(18A)	1.0000
C(11)-C(16)	1.390(2)	C(20A)-H(20D)	0.9800
C(12)-C(13)	1.386(2)	C(20A)-H(20E)	0.9800
C(12)-H(12)	0.9500	C(20A)-H(20F)	0.9800

Table S19. Bond angles [°] for **27**.

C(4)-N-C(7)	108.68(11)	C(13)-C(14)-C(17)	121.49(16)
C(4)-N-C(3)	124.37(12)	C(15)-C(14)-C(17)	120.83(16)
C(7)-N-C(3)	124.95(12)	C(16)-C(15)-C(14)	121.59(15)
C(3)-O(1)-C(2)	115.17(11)	C(16)-C(15)-H(15)	119.2
C(2)-C(1)-Cl(3)	111.37(11)	C(14)-C(15)-H(15)	119.2
C(2)-C(1)-Cl(1)	111.21(11)	C(15)-C(16)-C(11)	120.55(15)
Cl(3)-C(1)-Cl(1)	109.01(8)	C(15)-C(16)-H(16)	119.7
C(2)-C(1)-Cl(2)	106.23(10)	C(11)-C(16)-H(16)	119.7
Cl(3)-C(1)-Cl(2)	110.28(8)	C(18)-C(17)-C(14)	115.51(17)
Cl(1)-C(1)-Cl(2)	108.69(8)	C(14)-C(17)-C(18A)	112.9(4)
O(1)-C(2)-C(1)	108.44(12)	C(18)-C(17)-H(17A)	108.4
O(1)-C(2)-H(2A)	110.0	C(14)-C(17)-H(17A)	108.4
C(1)-C(2)-H(2A)	110.0	C(18)-C(17)-H(17B)	108.4
O(1)-C(2)-H(2B)	110.0	C(14)-C(17)-H(17B)	108.4
C(1)-C(2)-H(2B)	110.0	H(17A)-C(17)-H(17B)	107.5
H(2A)-C(2)-H(2B)	108.4	C(14)-C(17)-H(17C)	109.0
O(2)-C(3)-O(1)	126.03(13)	C(18A)-C(17)-H(17C)	109.0
O(2)-C(3)-N	125.03(13)	C(14)-C(17)-H(17D)	109.0
O(1)-C(3)-N	108.94(12)	C(18A)-C(17)-H(17D)	109.0
C(5)-C(4)-N	108.05(12)	H(17C)-C(17)-H(17D)	107.8
C(5)-C(4)-H(4)	126.0	C(18)-C(19)-H(19A)	109.5
N-C(4)-H(4)	126.0	C(18)-C(19)-H(19B)	109.5
C(4)-C(5)-C(6)	107.98(12)	H(19A)-C(19)-H(19B)	109.5
C(4)-C(5)-H(5)	126.0	C(18)-C(19)-H(19C)	109.5
C(6)-C(5)-H(5)	126.0	H(19A)-C(19)-H(19C)	109.5
C(7)-C(6)-C(5)	108.09(13)	H(19B)-C(19)-H(19C)	109.5
C(7)-C(6)-H(6)	126.0	C(18A)-C(19)-H(19D)	109.5
C(5)-C(6)-H(6)	126.0	C(18A)-C(19)-H(19E)	109.5
C(6)-C(7)-N	107.19(12)	H(19D)-C(19)-H(19E)	109.5
C(6)-C(7)-C(8)	128.47(13)	C(18A)-C(19)-H(19F)	109.5
N-C(7)-C(8)	122.69(12)	H(19D)-C(19)-H(19F)	109.5
O(3)-C(8)-C(7)	121.15(13)	H(19E)-C(19)-H(19F)	109.5
O(3)-C(8)-C(9)	122.74(12)	C(17)-C(18)-C(20)	109.8(2)
C(7)-C(8)-C(9)	116.10(11)	C(17)-C(18)-C(19)	110.79(19)
C(11)-C(9)-C(8)	109.03(11)	C(20)-C(18)-C(19)	111.0(2)
C(11)-C(9)-C(10)	112.14(12)	C(17)-C(18)-H(18)	108.4
C(8)-C(9)-C(10)	110.89(12)	C(20)-C(18)-H(18)	108.4
C(11)-C(9)-H(9)	108.2	C(19)-C(18)-H(18)	108.4
C(8)-C(9)-H(9)	108.2	C(18)-C(20)-H(20A)	109.5
C(10)-C(9)-H(9)	108.2	C(18)-C(20)-H(20B)	109.5
C(9)-C(10)-H(10A)	109.5	H(20A)-C(20)-H(20B)	109.5
C(9)-C(10)-H(10B)	109.5	C(18)-C(20)-H(20C)	109.5
H(10A)-C(10)-H(10B)	109.5	H(20A)-C(20)-H(20C)	109.5
C(9)-C(10)-H(10C)	109.5	H(20B)-C(20)-H(20C)	109.5
H(10A)-C(10)-H(10C)	109.5	C(19)-C(18A)-C(20A)	110.7(8)
H(10B)-C(10)-H(10C)	109.5	C(19)-C(18A)-C(17)	109.9(6)
C(12)-C(11)-C(16)	117.95(14)	C(20A)-C(18A)-C(17)	106.6(9)
C(12)-C(11)-C(9)	120.44(13)	C(19)-C(18A)-H(18A)	109.9
C(16)-C(11)-C(9)	121.61(13)	C(20A)-C(18A)-H(18A)	109.9
C(13)-C(12)-C(11)	121.10(15)	C(17)-C(18A)-H(18A)	109.9
C(13)-C(12)-H(12)	119.5	C(18A)-C(20A)-H(20D)	109.5
C(11)-C(12)-H(12)	119.5	C(18A)-C(20A)-H(20E)	109.5
C(12)-C(13)-C(14)	121.13(15)	H(20D)-C(20A)-H(20E)	109.5
C(12)-C(13)-H(13)	119.4	C(18A)-C(20A)-H(20F)	109.5
C(14)-C(13)-H(13)	119.4	H(20D)-C(20A)-H(20F)	109.5
C(13)-C(14)-C(15)	117.64(15)	H(20E)-C(20A)-H(20F)	109.5

Table S20. Anisotropic displacement parameters (Å^{2}x 10^3) for **27**. The anisotropic displacement factor exponent takes the form: $-2p^2[h^2 a^{*2}U^{11} + ... + 2 h k a^* b^* U^{12}]$

	U^{11}	U^{22}	U^{33}	U^{23}	U^{13}	U^{12}
N	23(1)	17(1)	18(1)	-1(1)	2(1)	2(1)
O(1)	29(1)	40(1)	19(1)	1(1)	5(1)	8(1)
O(2)	28(1)	33(1)	21(1)	2(1)	0(1)	2(1)
O(3)	34(1)	20(1)	28(1)	5(1)	6(1)	4(1)
Cl(1)	30(1)	56(1)	45(1)	13(1)	2(1)	-2(1)
Cl(2)	56(1)	58(1)	25(1)	6(1)	16(1)	11(1)
Cl(3)	72(1)	24(1)	57(1)	-9(1)	21(1)	-7(1)
C(1)	32(1)	24(1)	25(1)	0(1)	8(1)	2(1)
C(2)	37(1)	32(1)	20(1)	3(1)	8(1)	12(1)
C(3)	28(1)	15(1)	20(1)	-2(1)	3(1)	2(1)
C(4)	24(1)	18(1)	25(1)	-2(1)	-1(1)	4(1)
C(5)	30(1)	18(1)	23(1)	1(1)	-3(1)	1(1)
C(6)	29(1)	18(1)	20(1)	0(1)	2(1)	-2(1)
C(7)	23(1)	16(1)	20(1)	-2(1)	2(1)	-2(1)
C(8)	26(1)	17(1)	17(1)	-2(1)	1(1)	0(1)
C(9)	25(1)	23(1)	20(1)	-1(1)	2(1)	2(1)
C(10)	30(1)	28(1)	34(1)	-2(1)	5(1)	7(1)
C(11)	21(1)	24(1)	25(1)	1(1)	2(1)	2(1)
C(12)	34(1)	32(1)	25(1)	0(1)	5(1)	-7(1)
C(13)	34(1)	38(1)	31(1)	-8(1)	5(1)	-6(1)
C(14)	26(1)	28(1)	45(1)	-3(1)	1(1)	-1(1)
C(15)	36(1)	34(1)	40(1)	9(1)	5(1)	-8(1)
C(16)	33(1)	35(1)	27(1)	3(1)	5(1)	-4(1)
C(17)	39(1)	32(1)	62(1)	-7(1)	2(1)	-6(1)
C(19)	48(1)	57(1)	82(2)	4(1)	-14(1)	-7(1)
C(18)	32(1)	39(1)	56(2)	2(1)	-1(1)	-7(1)
C(20)	49(2)	49(2)	89(2)	4(2)	-15(2)	-22(1)
C(18A)	36(5)	42(5)	58(6)	-25(4)	-3(4)	-9(4)
C(20A)	68(8)	56(8)	123(13)	-51(8)	-39(8)	5(6)

Table S21. Hydrogen coordinates (x 10^4) and isotropic displacement parameters ($Å^2$x 10^3) for **27**.

	x	y	z	U(eq)
H(2A)	6175	7402	1247	35
H(2B)	5428	6608	823	35
H(4)	6450	5620	4838	28
H(5)	5981	5350	7038	29
H(6)	4453	6165	7136	27
H(9)	3068	7044	6424	27
H(10A)	2139	8087	4287	46
H(10B)	2623	8528	5628	46
H(10C)	1730	7886	5631	46
H(12)	2675	6504	2999	36
H(13)	1985	5167	2097	41
H(15)	1326	4431	5638	44
H(16)	2041	5752	6559	38
H(17A)	1463	3615	2391	54
H(17B)	1404	3240	3837	54
H(17C)	801	3474	3825	54
H(17D)	1711	3303	3140	54
H(19A)	11	5038	2125	96
H(19B)	-868	4396	1694	96
H(19C)	58	4257	1034	96
H(19D)	5	5035	2146	96
H(19E)	-558	4135	2478	96
H(19F)	-568	4459	988	96
H(18)	-105	3880	3746	52
H(20A)	-828	2709	2372	97
H(20B)	67	2300	3242	97
H(20C)	105	2552	1734	97
H(18A)	972	4164	1122	56
H(20D)	-163	2936	474	130
H(20E)	24	2560	1947	130
H(20F)	838	2554	1040	130

Table S22. Torsion angles [°] for **27**.

C(3)-O(1)-C(2)-C(1)	-143.86(13)	C(7)-C(8)-C(9)-C(11)	-65.85(15)
Cl(3)-C(1)-C(2)-O(1)	65.42(15)	O(3)-C(8)-C(9)-C(10)	-9.67(19)
Cl(1)-C(1)-C(2)-O(1)	-56.39(15)	C(7)-C(8)-C(9)-C(10)	170.20(12)
Cl(2)-C(1)-C(2)-O(1)	-174.48(10)	C(8)-C(9)-C(11)-C(12)	-42.64(17)
C(2)-O(1)-C(3)-O(2)	6.2(2)	C(10)-C(9)-C(11)-C(12)	80.56(17)
C(2)-O(1)-C(3)-N	-174.01(11)	C(8)-C(9)-C(11)-C(16)	137.61(14)
C(4)-N-C(3)-O(2)	146.10(14)	C(10)-C(9)-C(11)-C(16)	-99.19(16)
C(7)-N-C(3)-O(2)	-16.0(2)	C(16)-C(11)-C(12)-C(13)	-1.3(2)
C(4)-N-C(3)-O(1)	-33.66(17)	C(9)-C(11)-C(12)-C(13)	178.91(14)
C(7)-N-C(3)-O(1)	164.26(12)	C(11)-C(12)-C(13)-C(14)	0.2(3)
C(7)-N-C(4)-C(5)	-0.92(15)	C(12)-C(13)-C(14)-C(15)	1.6(2)
C(3)-N-C(4)-C(5)	-165.48(13)	C(12)-C(13)-C(14)-C(17)	-176.15(16)
N-C(4)-C(5)-C(6)	0.39(16)	C(13)-C(14)-C(15)-C(16)	-2.3(3)
C(4)-C(5)-C(6)-C(7)	0.29(16)	C(17)-C(14)-C(15)-C(16)	175.47(16)
C(5)-C(6)-C(7)-N	-0.85(15)	C(14)-C(15)-C(16)-C(11)	1.2(3)
C(5)-C(6)-C(7)-C(8)	-166.29(13)	C(12)-C(11)-C(16)-C(15)	0.6(2)
C(4)-N-C(7)-C(6)	1.09(15)	C(9)-C(11)-C(16)-C(15)	-179.60(14)
C(3)-N-C(7)-C(6)	165.54(12)	C(13)-C(14)-C(17)-C(18)	-102.4(2)
C(4)-N-C(7)-C(8)	167.58(12)	C(15)-C(14)-C(17)-C(18)	79.9(2)
C(3)-N-C(7)-C(8)	-27.97(19)	C(13)-C(14)-C(17)-C(18A)	-42.7(4)
C(6)-C(7)-C(8)-O(3)	139.01(15)	C(15)-C(14)-C(17)-C(18A)	139.7(4)
N-C(7)-C(8)-O(3)	-24.4(2)	C(14)-C(17)-C(18)-C(20)	-172.8(2)
C(6)-C(7)-C(8)-C(9)	-40.86(19)	C(14)-C(17)-C(18)-C(19)	64.2(3)
N-C(7)-C(8)-C(9)	155.71(12)	C(14)-C(17)-C(18A)-C(19)	-64.5(7)
O(3)-C(8)-C(9)-C(11)	114.27(14)	C(14)-C(17)-C(18A)-C(20A)	175.4(8)

Fig S203: ORTEP diagram for **31**.

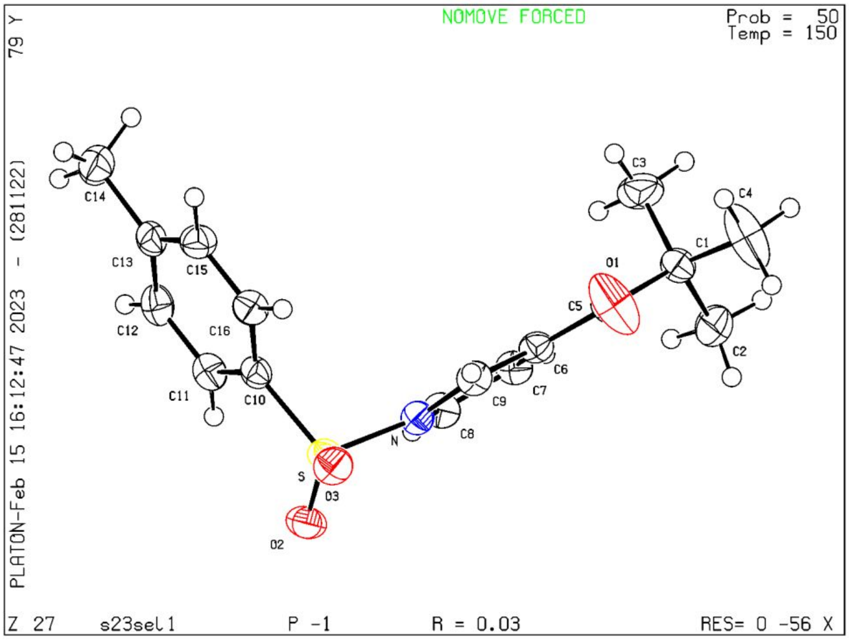

Table S23. Crystal data and structure refinement for **31**.

CCDC Identification code	2242265	
Empirical formula	$C_{16}H_{19}NO_3S$	
Formula weight	305.38	
Temperature	150.00(10) K	
Wavelength	1.54184 Å	
Crystal system	Triclinic	
Space group	P-1	
Unit cell dimensions	a = 6.07652(17) Å	a= 87.332(2)°.
	b = 8.8561(2) Å	b= 83.484(2)°.
	c = 14.7460(4) Å	g = 80.079(2)°.
Volume	776.33(4) $Å^3$	
Z	2	
Density (calculated)	1.306 Mg/m^3	
Absorption coefficient	1.934 mm^{-1}	
F(000)	324	
Crystal size	0.200 x 0.100 x 0.030 mm^3	
Theta range for data collection	5.072 to 72.773°.	
Index ranges	-6<=h<=7, -10<=k<=10, -18<=l<=18	
Reflections collected	12296	
Independent reflections	3075 [R(int) = 0.0195]	
Completeness to theta = 67.684°	100.0 %	
Absorption correction	Semi-empirical from equivalents	
Max. and min. transmission	1.00000 and 0.87144	
Refinement method	Full-matrix least-squares on F^2	
Data / restraints / parameters	3075 / 0 / 194	
Goodness-of-fit on F^2	1.033	
Final R indices [I>2sigma(I)]	R1 = 0.0338, wR2 = 0.0893	
R indices (all data)	R1 = 0.0352, wR2 = 0.0904	
Extinction coefficient	n/a	
Largest diff. peak and hole	0.336 and -0.312 e.$Å^{-3}$	

Table S24. Atomic coordinates (x 10^4) and equivalent isotropic displacement parameters ($Å^2$x 10^3) for **31**. U(eq) is defined as one third of the trace of the orthogonalized U^{ij} tensor.

	x	y	z	U(eq)
S	7115(1)	2533(1)	8488(1)	29(1)
N	7016(2)	2276(1)	7374(1)	28(1)
O(1)	11083(2)	2846(2)	5033(1)	65(1)
O(2)	5937(2)	1426(1)	8962(1)	40(1)
O(3)	9428(2)	2541(1)	8576(1)	37(1)
C(1)	8308(2)	2569(2)	4065(1)	32(1)
C(2)	7342(4)	1104(2)	3970(1)	51(1)
C(3)	6450(4)	3947(2)	3968(1)	52(1)
C(4)	10243(4)	2642(3)	3326(1)	70(1)
C(5)	9175(2)	2632(2)	4993(1)	34(1)
C(6)	7766(2)	2445(2)	5868(1)	28(1)
C(7)	5571(2)	2046(2)	6085(1)	31(1)
C(8)	5144(2)	1955(2)	7004(1)	32(1)
C(9)	8581(2)	2600(2)	6681(1)	30(1)
C(10)	5569(2)	4373(2)	8675(1)	27(1)
C(11)	3330(2)	4546(2)	9023(1)	32(1)
C(12)	2136(3)	6015(2)	9143(1)	35(1)
C(13)	3126(3)	7304(2)	8913(1)	32(1)
C(14)	1799(3)	8894(2)	9018(1)	46(1)
C(15)	5387(3)	7090(2)	8572(1)	35(1)
C(16)	6620(2)	5636(2)	8450(1)	32(1)

Table S25. Bond lengths [Å] for **31**.

S-O(2)	1.4237(11)	C(6)-C(9)	1.370(2)
S-O(3)	1.4276(11)	C(6)-C(7)	1.4361(19)
S-N	1.6770(12)	C(7)-C(8)	1.353(2)
S-C(10)	1.7522(14)	C(7)-H(7)	0.9500
N-C(9)	1.3699(19)	C(8)-H(8)	0.9500
N-C(8)	1.3929(18)	C(9)-H(9)	0.9500
O(1)-C(5)	1.2150(19)	C(10)-C(11)	1.384(2)
C(1)-C(4)	1.517(2)	C(10)-C(16)	1.391(2)
C(1)-C(3)	1.526(2)	C(11)-C(12)	1.385(2)
C(1)-C(5)	1.528(2)	C(11)-H(11)	0.9500
C(1)-C(2)	1.531(2)	C(12)-C(13)	1.391(2)
C(2)-H(2A)	0.9800	C(12)-H(12)	0.9500
C(2)-H(2B)	0.9800	C(13)-C(15)	1.393(2)
C(2)-H(2C)	0.9800	C(13)-C(14)	1.502(2)
C(3)-H(3A)	0.9800	C(14)-H(14A)	0.9800
C(3)-H(3B)	0.9800	C(14)-H(14B)	0.9800
C(3)-H(3C)	0.9800	C(14)-H(14C)	0.9800
C(4)-H(4A)	0.9800	C(15)-C(16)	1.382(2)
C(4)-H(4B)	0.9800	C(15)-H(15)	0.9500
C(4)-H(4C)	0.9800	C(16)-H(16)	0.9500
C(5)-C(6)	1.484(2)		

Table S26. Bond angles [°] for **31**.

O(2)-S-O(3)	121.62(7)	C(9)-C(6)-C(7)	106.78(13)
O(2)-S-N	105.76(6)	C(9)-C(6)-C(5)	120.16(13)
O(3)-S-N	104.98(6)	C(7)-C(6)-C(5)	133.01(13)
O(2)-S-C(10)	109.43(7)	C(8)-C(7)-C(6)	108.14(13)
O(3)-S-C(10)	109.61(7)	C(8)-C(7)-H(7)	125.9
N-S-C(10)	103.82(6)	C(6)-C(7)-H(7)	125.9
C(9)-N-C(8)	109.26(12)	C(7)-C(8)-N	107.54(13)
C(9)-N-S	125.50(10)	C(7)-C(8)-H(8)	126.2
C(8)-N-S	124.40(10)	N-C(8)-H(8)	126.2
C(4)-C(1)-C(3)	110.22(17)	C(6)-C(9)-N	108.25(12)
C(4)-C(1)-C(5)	108.41(13)	C(6)-C(9)-H(9)	125.9
C(3)-C(1)-C(5)	108.22(13)	N-C(9)-H(9)	125.9
C(4)-C(1)-C(2)	109.40(16)	C(11)-C(10)-C(16)	121.36(14)
C(3)-C(1)-C(2)	108.54(15)	C(11)-C(10)-S	119.83(11)
C(5)-C(1)-C(2)	112.05(13)	C(16)-C(10)-S	118.79(11)
C(1)-C(2)-H(2A)	109.5	C(10)-C(11)-C(12)	118.54(14)
C(1)-C(2)-H(2B)	109.5	C(10)-C(11)-H(11)	120.7
H(2A)-C(2)-H(2B)	109.5	C(12)-C(11)-H(11)	120.7
C(1)-C(2)-H(2C)	109.5	C(11)-C(12)-C(13)	121.63(14)
H(2A)-C(2)-H(2C)	109.5	C(11)-C(12)-H(12)	119.2
H(2B)-C(2)-H(2C)	109.5	C(13)-C(12)-H(12)	119.2
C(1)-C(3)-H(3A)	109.5	C(12)-C(13)-C(15)	118.34(14)
C(1)-C(3)-H(3B)	109.5	C(12)-C(13)-C(14)	121.28(15)
H(3A)-C(3)-H(3B)	109.5	C(15)-C(13)-C(14)	120.37(15)
C(1)-C(3)-H(3C)	109.5	C(13)-C(14)-H(14A)	109.5
H(3A)-C(3)-H(3C)	109.5	C(13)-C(14)-H(14B)	109.5
H(3B)-C(3)-H(3C)	109.5	H(14A)-C(14)-H(14B)	109.5
C(1)-C(4)-H(4A)	109.5	C(13)-C(14)-H(14C)	109.5
C(1)-C(4)-H(4B)	109.5	H(14A)-C(14)-H(14C)	109.5
H(4A)-C(4)-H(4B)	109.5	H(14B)-C(14)-H(14C)	109.5
C(1)-C(4)-H(4C)	109.5	C(16)-C(15)-C(13)	121.18(14)
H(4A)-C(4)-H(4C)	109.5	C(16)-C(15)-H(15)	119.4
H(4B)-C(4)-H(4C)	109.5	C(13)-C(15)-H(15)	119.4
O(1)-C(5)-C(6)	117.59(14)	C(15)-C(16)-C(10)	118.93(14)
O(1)-C(5)-C(1)	119.89(14)	C(15)-C(16)-H(16)	120.5
C(6)-C(5)-C(1)	122.52(12)	C(10)-C(16)-H(16)	120.5

Table S27. Anisotropic displacement parameters ($Å^2$x 10^3) for **31**. The anisotropic displacement factor exponent takes the form: $-2p^2[h^2 a^{*2}U^{11} + ... + 2 h k a^* b^* U^{12}]$

	U^{11}	U^{22}	U^{33}	U^{23}	U^{13}	U^{12}
S	32(1)	29(1)	28(1)	1(1)	-11(1)	-4(1)
N	27(1)	30(1)	29(1)	-2(1)	-8(1)	-5(1)
O(1)	31(1)	131(1)	39(1)	9(1)	-9(1)	-28(1)
O(2)	53(1)	34(1)	35(1)	7(1)	-11(1)	-12(1)
O(3)	34(1)	40(1)	40(1)	-2(1)	-17(1)	-1(1)
C(1)	31(1)	35(1)	30(1)	1(1)	-7(1)	-6(1)
C(2)	75(1)	37(1)	44(1)	-9(1)	-4(1)	-15(1)
C(3)	69(1)	38(1)	51(1)	3(1)	-30(1)	3(1)
C(4)	50(1)	135(2)	32(1)	-3(1)	-1(1)	-38(1)
C(5)	26(1)	43(1)	34(1)	3(1)	-8(1)	-4(1)
C(6)	25(1)	27(1)	32(1)	0(1)	-9(1)	-1(1)
C(7)	29(1)	32(1)	34(1)	1(1)	-11(1)	-8(1)
C(8)	29(1)	34(1)	36(1)	0(1)	-9(1)	-11(1)
C(9)	23(1)	33(1)	34(1)	-1(1)	-7(1)	-3(1)
C(10)	29(1)	31(1)	23(1)	0(1)	-7(1)	-6(1)
C(11)	32(1)	38(1)	29(1)	-1(1)	-4(1)	-12(1)
C(12)	29(1)	47(1)	29(1)	-5(1)	-2(1)	-5(1)
C(13)	38(1)	36(1)	22(1)	-3(1)	-8(1)	-1(1)
C(14)	52(1)	42(1)	38(1)	-6(1)	-7(1)	7(1)
C(15)	39(1)	31(1)	35(1)	2(1)	-4(1)	-9(1)
C(16)	29(1)	35(1)	33(1)	1(1)	-3(1)	-8(1)

Table S28. Hydrogen coordinates (x 10^4) and isotropic displacement parameters ($Å^2$x 10^3) for **31**.

	x	y	z	U(eq)
H(2A)	6976	1048	3343	77
H(2B)	5977	1119	4395	77
H(2C)	8454	209	4110	77
H(3A)	5966	3980	3354	78
H(3B)	7020	4891	4063	78
H(3C)	5172	3855	4424	78
H(4A)	9696	2664	2725	105
H(4B)	11405	1738	3385	105
H(4C)	10877	3571	3392	105
H(7)	4589	1875	5657	37
H(8)	3810	1715	7337	38
H(9)	9988	2883	6753	36
H(11)	2627	3675	9176	38
H(12)	602	6145	9389	42
H(14A)	1511	9344	8414	68
H(14B)	2653	9529	9320	68
H(14C)	366	8845	9388	68
H(15)	6095	7958	8420	42
H(16)	8162	5502	8216	38

Table S29. Torsion angles [°] for **31**.

O(2)-S-N-C(9)	-152.08(12)	C(7)-C(6)-C(9)-N	1.54(16)
O(3)-S-N-C(9)	-22.33(14)	C(5)-C(6)-C(9)-N	-176.16(12)
C(10)-S-N-C(9)	92.74(13)	C(8)-N-C(9)-C(6)	-1.80(16)
O(2)-S-N-C(8)	39.61(13)	S-N-C(9)-C(6)	-171.60(10)
O(3)-S-N-C(8)	169.36(11)	O(2)-S-C(10)-C(11)	-15.65(13)
C(10)-S-N-C(8)	-75.57(13)	O(3)-S-C(10)-C(11)	-151.37(11)
C(4)-C(1)-C(5)-O(1)	5.8(2)	N-S-C(10)-C(11)	96.90(12)
C(3)-C(1)-C(5)-O(1)	-113.71(19)	O(2)-S-C(10)-C(16)	165.74(11)
C(2)-C(1)-C(5)-O(1)	126.66(19)	O(3)-S-C(10)-C(16)	30.02(13)
C(4)-C(1)-C(5)-C(6)	-174.40(17)	N-S-C(10)-C(16)	-81.71(12)
C(3)-C(1)-C(5)-C(6)	66.06(19)	C(16)-C(10)-C(11)-C(12)	0.1(2)
C(2)-C(1)-C(5)-C(6)	-53.6(2)	S-C(10)-C(11)-C(12)	-178.44(11)
O(1)-C(5)-C(6)-C(9)	3.6(2)	C(10)-C(11)-C(12)-C(13)	0.8(2)
C(1)-C(5)-C(6)-C(9)	-176.18(14)	C(11)-C(12)-C(13)-C(15)	-1.3(2)
O(1)-C(5)-C(6)-C(7)	-173.39(17)	C(11)-C(12)-C(13)-C(14)	178.02(14)
C(1)-C(5)-C(6)-C(7)	6.8(3)	C(12)-C(13)-C(15)-C(16)	0.9(2)
C(9)-C(6)-C(7)-C(8)	-0.73(17)	C(14)-C(13)-C(15)-C(16)	-178.39(14)
C(5)-C(6)-C(7)-C(8)	176.55(15)	C(13)-C(15)-C(16)-C(10)	-0.1(2)
C(6)-C(7)-C(8)-N	-0.35(17)	C(11)-C(10)-C(16)-C(15)	-0.5(2)
C(9)-N-C(8)-C(7)	1.33(17)	S-C(10)-C(16)-C(15)	178.11(11)
S-N-C(8)-C(7)	171.26(10)		

References

1 - Pollack, S. R.; Dion, A. Metal-Free Stereoselective Synthesis of (*E*)- and (*Z*)-*N*-Monosubstituted β-Aminoacrylates via Condensation Reactions of Carbamates. *J. Org. Chem.* **2021**, *86*, 11748–11762.

2 - Kondasinghe, T. D.; Saraha, H. Y.; Odeesho, S. B.; Stockdill, J. L. Direct Palladium-Mediated on-Resin Disulfide Formation from Allocam Protected Peptides. *Org. Biomol. Chem.* **2017**, *15*, 2914–2918.

3 - Gwon, D.; Hwang, H.; Kim, H. K.; Marder, S. R.; Chang, S. Synthesis of 8-Aminoquinolines by Using Carbamate Reagents: Facile Installation and Deprotection of Practical Amidating Groups. *Chem. Eur. J.* **2015**, *21*, 17200–17204.

4 - Qin, L.; Wang, B.; Zhang, Y.; Chen, L.; Gao, G. Anion Exchange: A Novel Way of Preparing Hierarchical Porous Structure in Poly(Ionic Liquid)s. *Chem. Commun.* **2017**, *53*, 3785–3788.

5 - Zhou, M.-J.; Zhang, L.; Liu, G.; Xu, C.; Huang, Z. Site-Selective Acceptorless Dehydrogenation of Aliphatics Enabled by Organophotoredox/Cobalt Dual Catalysis. *J. Am. Chem. Soc.* **2021**, *143*, 16470–16485.

6 - Davies, H. M.; Saikali, E.; Young, W. B. Synthesis of (±)-Ferruginine and (±)-Anhydroecgonine Methyl-Ester by a Tandem Cyclopropanation/Cope Rearrangement. *J. Org. Chem.* **1991**, *56*, 5696–5700.

7 - Trost, B. M.; Xu, J.; Schmidt, T. Palladium-Catalyzed Decarboxylative Asymmetric Allylic Alkylation of Enol Carbonates. *J. Am. Chem. Soc.* **2009**, *131*, 18343–18357.

8 - Huang, P.-Q.; Huang, Y.-H.; Xiao, K.-J. Metal-Free Intermolecular Coupling of Arenes with Secondary Amides: Chemoselective Synthesis of Aromatic Ketimines and Ketones, and *N*-Deacylation of Secondary Amides. *J. Am. Chem. Soc.* **2016**, *81*, 9020–9027.

9 - Schwalm, C. S.; de Castro, I. B. D.; Ferrari, J.; de Oliveira, F. L.; Aparicio, R.; Correia, C. R. Synthesis of Pentabromopseudilin and Other Arylpyrrole Derivatives via Heck Arylations. *Tetrahedron Lett.* **2012**, *53*, 1660–1663.

10 - Slack, T. J.; Li, W.; Shi, D.; McArthur, J. B.; Zhao, G.; Li, Y.; Xiao, A.; Khedri, Z.; Yu, H.; Liu, Y.; Chen, X. Triazole-Linked Transition State Analogs as Selective Inhibitors against *V. cholerae* Sialidase. *Bioorg. Med. Chem.* **2018**, *26* (21), 5751–5757.

11 - Cooper, G. H. Cyclopropyl 2-Pyrrolyl Ketone. *J. Org. Chem.* **1971**, *36*, 2897–2898.
